# Efficient syntheses of (–)-crinine and (–)-aspidospermidine, and the formal synthesis of (–)-minfiensine by enantioselective intramolecular dearomative cyclization†
†Electronic supplementary information (ESI) available. CCDC 1444649 and 1537084. For ESI and crystallographic data in CIF or other electronic format see DOI: 10.1039/c7sc01859b



**DOI:** 10.1039/c7sc01859b

**Published:** 2017-07-03

**Authors:** Kang Du, He Yang, Pan Guo, Liang Feng, Guangqing Xu, Qinghai Zhou, Lung Wa Chung, Wenjun Tang

**Affiliations:** a State Key Laboratory of Bio-Organic & Natural Products Chemistry , Shanghai Institute of Organic Chemistry , Chinese Academy of Sciences , 345 Lingling Road , Shanghai 200032 , China . Email: tangwenjun@sioc.ac.cn; b Department of Chemistry , South University of Science and Technology of China , Shenzhen 518055 , China; c College of Chemistry , Nankai University , Tianjin 300071 , China

## Abstract

Polycyclic alkaloids bearing all-carbon quaternary centers possess a diversity of biological activities and are challenging targets in natural product synthesis. The development of a general and asymmetric catalytic method applicable to the efficient syntheses of a series of complex polycyclic alkaloids remains highly desirable in synthetic chemistry. Herein we describe an efficient palladium-catalyzed enantioselective dearomative cyclization which is capable of synthesizing two important classes of tricyclic nitrogen-containing skeleton, chiral dihydrophenanthridinone and dihydrocarbazolone derivatives bearing all-carbon quaternary centers, in excellent yields and enantioselectivities. The P-chiral monophosphorus ligand AntPhos is crucial for the reactivity and enantioselectivity, and the choice of the *N*-phosphoramide protecting group is essential for the desired chemoselectivity. This method has enabled the enantioselective total syntheses of three distinctive and challenging biologically important polycyclic alkaloids, specifically a concise and gram-scale synthesis of (–)-crinine, an efficient synthesis of indole alkaloid (–)-aspidospermidine and a formal enantioselective synthesis of (–)-minfiensine.

## Abstract 2

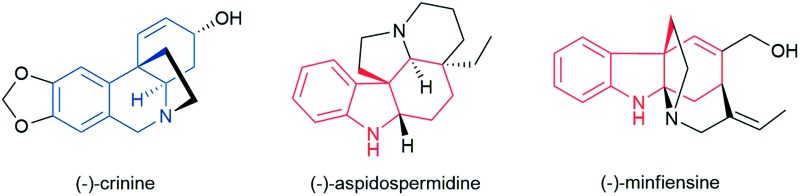
Palladium-catalyzed enantioselective dearomative cyclization has enabled the concise and enantioselective total syntheses of (–)-crinine and (–)-aspidospermidine, as well as a formal total synthesis of (–)-minfiensine.

## Introduction

**Fig. 1:**
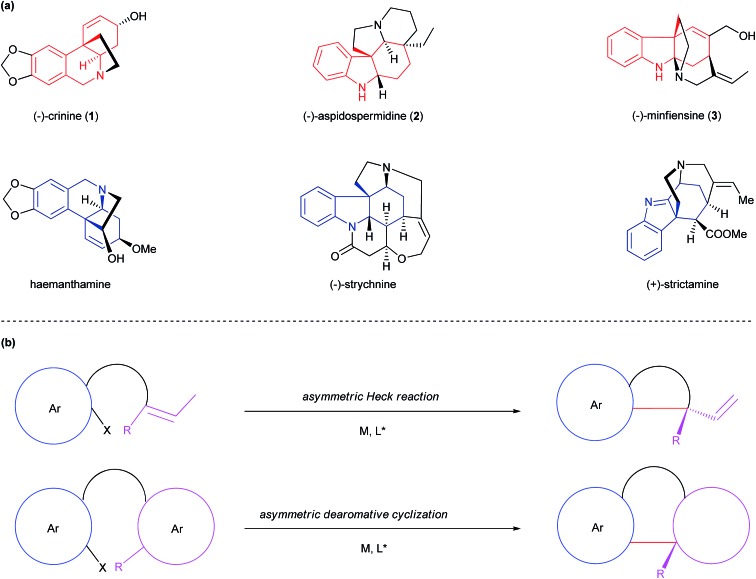
(a) Selected polycyclic alkaloids bearing all-carbon quaternary centers. (b) Asymmetric Heck reaction *vs.* asymmetric dearomative cyclization.

Numerous biologically important natural products are polycyclic alkaloids bearing one or more all-carbon quaternary center. As a representative alkaloid of the *Amaryllidaceae* family with significant biological activity, crinine (**1**) is characterized by a 5,10*b*-ethanophenathridine skeleton bearing an all-carbon quaternary center (Fig. 1a).^1^ Aspidospermidine (**2**) is a representative pentacyclic indole alkaloid of over 250 members of *Aspidosperma* alkaloids that exhibit significant respiratory stimulation and antibiotic activities.^2^ Minfiensine (**3**) is an important member of the *Strychnos* alkaloids possessing potent anticancer activity. Many structurally related alkaloids such as haemanthamine, strychnine and strictamine exhibit a variety of biological properties including potent anticancer, antimalarial and anti-inflammatory activities.^3^ Despite their biological importance, the efficient preparation of these natural products is a significant challenge in synthetic chemistry. A general, efficient and asymmetric catalytic method for the facile preparation of all of these polycyclic alkaloids remains highly desirable for the discovery of new therapeutic agents and drugs.

The development of a general and efficient asymmetric catalytic method for the concise syntheses of polycyclic natural products has become an important subject in organic chemistry. As a result, several elegant catalytic methods for the enantioselective construction of a polycyclic framework possessing all-carbon quaternary centers have been developed.^4^ Among them, the asymmetric intramolecular Heck reaction has become one of the most important methods (Fig. 1b).^5^ Despite its synthetic versatility, the asymmetric Heck cyclization employs an olefinic starting material which often requires multiple synthetic steps to prepare. In addition, the transformation of the olefinic product to a target molecule is not always straightforward. An alternative method is an enantioselective intramolecular dearomative cyclization,^6^ which usually employs a more accessible substrate with an aryl moiety and leads to a multi-cyclic skeleton bearing an all-carbon quaternary center. Because of the closer resemblance of the cyclic product to a variety of chiral natural products, this method offers advantages over the Heck reaction under certain circumstances for the asymmetric synthesis of polycyclic natural products.

We have previously developed an asymmetric palladium-catalyzed dearomative cyclization for the construction of chiral phenanthrenone and spiroenone derivatives bearing all-carbon quaternary centers and applied this method to terpene, steroid and polyketide syntheses.^7^ To accomplish the efficient syntheses of the challenging polycyclic skeletons possessed by crinine, aspidospermidine and minfiensine, a highly enantioselective palladium-catalyzed dearomative cyclization is reported in this paper. By employing this method, two important classes of tricyclic nitrogen-containing skeleton, 6,10*b*-dihydrophenanthridin-3(5*H*)-one and dihydrocarbazolone derivatives bearing all-carbon quaternary centers, are efficiently constructed with excellent enantioselectivities. The employment of a P-chiral monophosphorus ligand AntPhos and the use of a bulky phosphoramide protecting group at nitrogen are critical for the excellent reactivity, chemoselectivity and enantioselectivity of the dearomative cyclization. The advanced cyclization products have enabled us to accomplish the concise and gram-scale synthesis of (–)-crinine, offering a practical synthetic route to a series of crinine-type alkaloids. Although the two complex chiral natural products (–)-aspidospermidine and (–)-minfiensine belong to a different indoline alkaloid family, their distinctive structures can be derived from a common chiral dihydrocarbazolone intermediate which can be efficiently prepared by the enantioselective dearomative cyclization. This strategy has allowed us to accomplish for the first time the efficient synthesis of (–)-aspidospermidine as well as the formal enantioselective synthesis of (–)-minfiensine using the same asymmetric catalytic method. Herein we report our results.

## Results and discussion

### Retrosynthetic analysis of crinine

The synthesis of crinine has gained significant interest, resulting in many elegant synthetic strategies for the construction of the 5,10*b*-ethanophenathridine skeleton bearing an all-carbon quaternary center.^8^ Surprisingly, few asymmetric syntheses of crinine or vittatine have been reported.^9^ Early work by Overman described a beautiful synthesis of crinine by employing a chiral auxiliary.^9*c*^ Chida completed the asymmetric synthesis of vittatine, the antipode of crinine, by utilizing a chiral pool strategy.^9*a*^ A notable enantioselective synthesis of vittatine was developed by Fan using an organocatalytic Michael addition of α-cyanoketones to acrylates with up to 85% ee through a 15-step sequence.^9*b*^ Despite these reported synthetic efforts, a concise and highly enantioselective synthesis of **1** remains highly desirable.

**Fig. 2:**
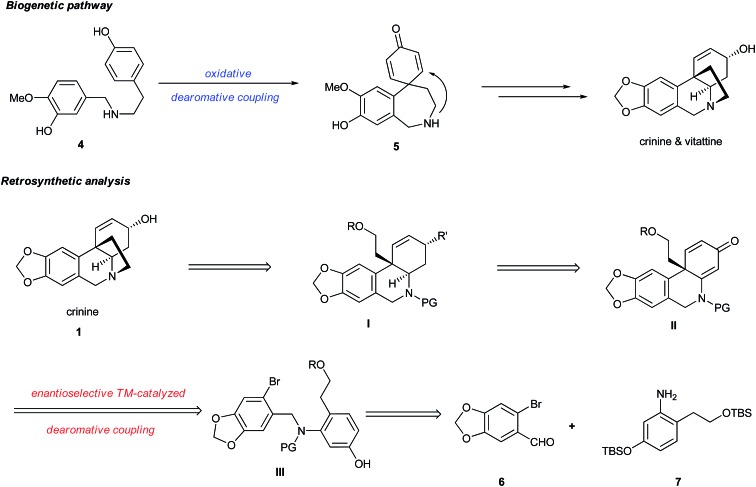
Retrosynthetic analysis of crinine (**1**).

It is regarded that the biogenetic synthesis of crinine (**1**) originates from norbelladine **4** through an intramolecular oxidative dearomative coupling followed by a facile intramolecular aza-Michael addition of **5** (Fig. 2).^10^ However, the asymmetric biomimetic synthesis of crinine remains to be achieved mainly due to two limitations in this pathway: (1) the oxidative dearomative coupling is nonselective and controlling its chemoselectivity is extremely difficult; (2) the intramolecular Michael addition is too facile to develop an enantioselective version, which indicates why both crinine and its antipode vittatine exist in nature. Inspired by the brevity as well as the limitation of this biogenetic pathway, we envisioned that a concise and enantioselective synthesis of crinine could be achieved by employing a transition-metal catalyzed intramolecular dearomative coupling strategy. The advantage of this approach is that it adopts a dearomative coupling reaction bearing resemblance to the biogenetic pathway. More importantly, the palladium-catalyzed intramolecular dearomative coupling could offer excellent chemo- and enantioselectivity that the biogenetic pathway lacks. Thus, crinine could be prepared by the ring closure of structure **I**, which could be synthesized from a chiral 6,10*b*-dihydrophenanthridin-3(5*H*)-one **II** by selective reductions. The key transformation is to construct structure **II** bearing a chiral all-carbon quaternary center from aryl bromide **III** through enantioselective palladium-catalyzed intramolecular dearomative coupling. We proposed that a chiral monophosphorus ligand developed in our laboratory could provide excellent reactivity, chemoselectivity and enantioselectivity for this challenging reaction.^11^ Bromide **III** could be synthesized from the readily available aryl aldehyde **6** and aniline **7** by a reductive amination process.

### Methodology development

**Fig. 3:**
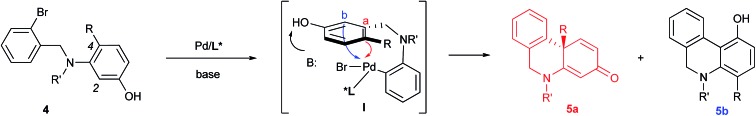
Chemoselectivity in asymmetric dearomative cyclization.

Based on the retrosynthetic analysis of crinine, we considered that its asymmetric synthesis could be efficiently accomplished if dihydrophenanthridine **5a** could be constructed by an efficient enantioselective dearomative cyclization of aniline **4** (Fig. 3). Although we previously reported the synthesis of chiral phenathrenone compounds,^7*b*^ the preparation of dihydrophenanthridine **5a** from aniline **4** could be challenging due to the conformational change caused by the nitrogen atom in the skeleton. Pathway **a** during the nucleophilic substitution of the palladium species **I** would lead to the formation of the desired chiral product **5a** after reductive elimination, while pathway **b** would provide the undesired non-chiral product **5b**. The choice between pathway **a** and **b** could be largely affected by the conformation of the palladium complex **I**.

We reasoned that the conformation of this palladium species could be adjusted by the *N*-R′ protecting group, which could have a significant effect on the chemoselectivity of the transformation. Thus, a series of nitrogen-containing substrates **8a–i** with various R protecting groups were prepared for the study (Table 1). The reactions were performed in toluene at 90 °C for 16 hours with K_2_CO_3_ as the base and a palladium catalyst loading of 2 mol% using (*S*)-**L1** (AntPhos) as the ligand (entries 1–9). The cyclization did not occur when free amine **8a** (R = H) was directly employed (entry 1). Surprisingly, a Piv-protected substrate **8b** proceeded to form solely the undesired cyclization product **10b** (entry 2). In order to alter the chemoselectivity, substrates with bulky sulfonyl protecting groups were tested (entries 3–8). Encouragingly, the Ms-protected substrate **8c** provided the desired cyclization product **9c** in 16% yield with 95% ee (entry 3). A Ts-protected substrate **8d** provided a significantly higher yield of **9d** (61%, entry 4). However, no better results were obtained from substrates with a Tris- or Nos-protecting group (entries 5–6). A slight improvement (yields > 70%) was observed when Tf- or Me_2_NSO_2_-protecting groups were employed (entries 7–8). Finally, when substrate **8i** with a bulky phosphoramide protecting group (Me_2_N)_2_P(O)- was subjected to the cyclization, the desired chiral dihydrophenanthridin-3(5*H*)-one product **9i** was isolated in 96% yield with 96% ee (entry 9). It is important to note that the ligand structure plays a significant role in the reactivity and selectivity of this reaction. (*S*)-**L1** (AntPhos) is responsible for the excellent reactivity, chemoselectivity and enantioselectivity, since the other related monophosphorus ligands **L2**, **L3**, **L4** and **L5** provided either diminished chemoselectivities, yields or ees (entries 10–13).

**Table 1:** Asymmetric dearomative cyclization of **8a–i**

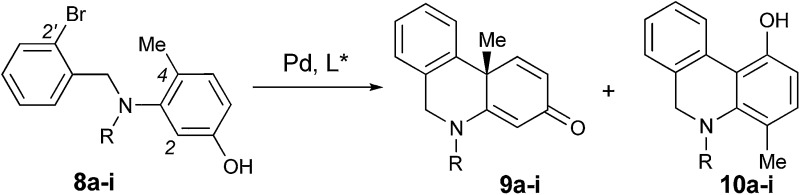					
Entry^*a*^	R (**8a–i**)	L*	Ratio of **9** : **10** ^*b*^	Yield of **9a–i** ^*c*^ (%)	ee of **9a–i** ^*d*^ (%)
1	H (**8a**)	(*S*)-**L1**	—	—	—
2	Piv (**8b**)	(*S*)-**L1**	<1 : 99	—	—
3	Ms (**8c**)	(*S*)-**L1**	21 : 79	16 (**9c**)	95
4	Ts (**8d**)	(*S*)-**L1**	68 : 32	61 (**9d**)	91
5	Tris (**8e**)	(*S*)-**L1**	64 : 36	54 (**9e**)	90
6	Nos (**8f**)	(*S*)-**L1**	95 : 5	37 (**9f**)	86
7	Tf (**8g**)	(*S*)-**L1**	72 : 28	70 (**9g**)	84
8	SO_2_NMe_2_ (**8h**)	(*S*)-**L1**	75 : 25	73 (**9h**)	94
**9**	**P(O)(NMe**_**2**_ **)**_**2**_ **(**8i**)**	**(*S*)-** **L1**	**99 : 1**	**96 (**9i**)**	**96**
10	P(O)(NMe_2_)_2_ (**8i**)	(*S*)-**L2**	99 : 1	90 (**9i**)	56
11	P(O)(NMe_2_)_2_ (**8i**)	(*S*)-**L3**	78 : 22	24 (**9i**)	70
12	P(O)(NMe_2_)_2_ (**8i**)	(*S*)-**L4**	88 : 12	83 (**9i**)	55
13	P(O)(NMe_2_)_2_ (**8i**)	(*S*)-**L5**	96 : 4	68 (**9i**)	89
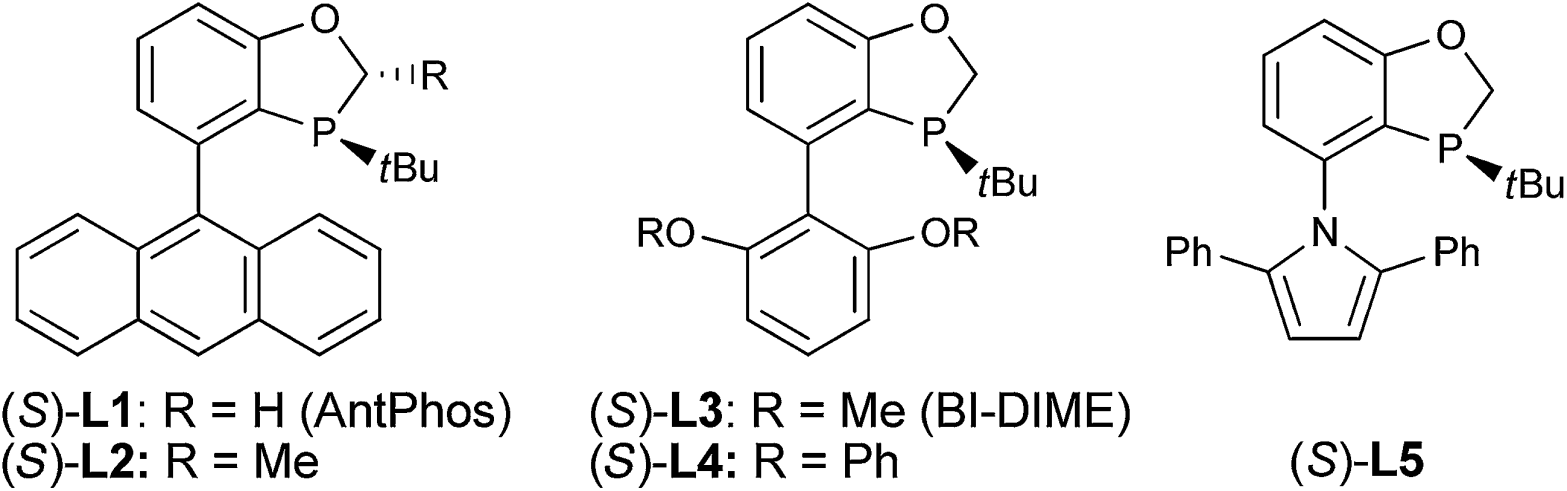					

^*a*^Unless otherwise specified, the reactions were performed in toluene at 90 °C under nitrogen for 16 h with K_2_CO_3_ (2 equiv.) as the base in the presence of 1 mol% [Pd(cinnamyl)Cl]_2_ and 2 mol% L* at a 0.1 mmol scale of **8a–i**. The absolute configurations of **9c–i** were assigned by analogy on the basis of compound **14**.

^*b*^The **9c–i** : **10c–i** ratios were determined by HPLC.

^*c*^Isolated yields of **9c–i**.

^*d*^Determined by chiral HPLC on a Chiralcel AD-H or OD-H column.

**Fig. 4:**
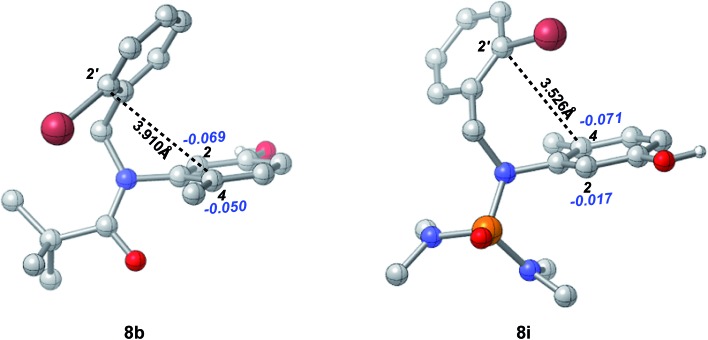
The optimized structures of substrates **8b** and **8i** at the B3LYP/6-31+G(d,p) level (the black bold numbers are the distances between C-2′ and C-4. The blue italic numbers are the NPA charges on the carbon atoms and their attached hydrogen atoms. Hydrogens (except those in the OH groups) have been omitted for clarity).

The effects of the *N*-R protecting groups on the chemoselectivity observed in our experiments were in accordance with the DFT calculations of the optimized structures of substrates **8b** and **8i** (Fig. 4). The C-2′ position is in closer proximity to the C-4 position in substrate **8i** (3.526 Å) than to that in substrate **8b** (3.910 Å). In addition, according to the NBO analysis, the charge on the C-4 position in **8i** is –0.071, which is more negative than that in **8b** (–0.050) and that on the C-2 position in **8i** (–0.017), indicating the higher nucleophilicity of the C-4 position in substrate **8i** which participates smoothly in the intramolecular dearomative cyclization.

### Synthesis of (–)-crinine

**Fig. 5:**
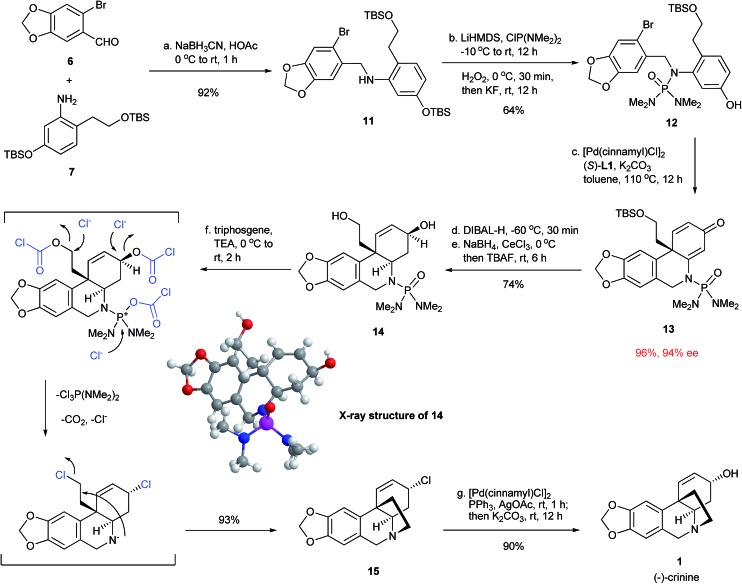
Enantioselective synthesis of (–)-crinine.

With the successful development of an efficient enantioselective dearomative cyclization, we turned our attention to complete the synthesis of crinine (Fig. 5). Thus, the reductive amination between 6-bromopiperonal **6** and readily accessible aniline **7** using NaBH_3_CN/HOAc provided secondary amine **11** in 92% yield. This was followed by the installation of the phosphoramide group at the nitrogen with LiHMDS/ClP(NMe_2_)_2_/H_2_O_2_ and subsequent treatment with KF/tetraethylene glycol to selectively deprotect the TBS aryl ether.^12^ The next step was the key intramolecular dearomative cyclization of bromo phenol **12**. Gratifyingly, the cyclization proceeded smoothly in the presence of 1 mol% [Pd(cinnamyl)Cl]_2_ and 2 mol% (*S*)-**L1** with potassium carbonate as the base to form compound **13** bearing an all-carbon quaternary center in 96% yield with 94% ee. This result further demonstrated the generality and excellent functional group compatibility of the enantioselective dearomative cross-coupling. Treatment of **13** with DIBAL-H at –78 °C selectively reduced the enamide double bond, which was followed by a Luche reduction and treatment with TBAF to stereospecifically give the allylic alcohol **14**, whose absolute structure and relative stereochemistry were confirmed by X-ray crystallographic analysis.^13^ The final tetrahydropyrrole ring formation required the deprotection of the phosphoramide moiety, as well as the activation of the primary alcohol in **14**. This was accomplished effectively in a single step by the treatment of **14** with triphosgene/Et_3_N to afford the cyclization product **15** in 93% yield. It is noteworthy that the allylic alcohol moiety in **14** was stereospecifically transformed into the allylic chloride functionality in **15**. The final task was the transformation of the allylic chloride to an allylic alcohol with the retention of its stereochemistry to give the final product crinine. A number of reaction conditions such as using H_2_O, H_2_O/AcOH and AgOAc/AcOH ^14^ were studied and they all provided mixtures of stereoisomers along with a diene side-product. We were delighted that the employment of [Pd(cinnamyl)Cl]_2_, PPh_3_ and AgOAc ^15^ stereoselectively afforded an allylic acetate with the desired stereochemistry, which, after basic hydrolysis, led to (–)-crinine (**1**) in 90% yield and a 35% overall yield from 6-bromopiperonal **6**. Over 1 g of (–)-crinine was successfully prepared, demonstrating the practicality of this synthetic route. The work constitutes the most efficient enantioselective synthesis of (–)-crinine to date.

**Fig. 6:**
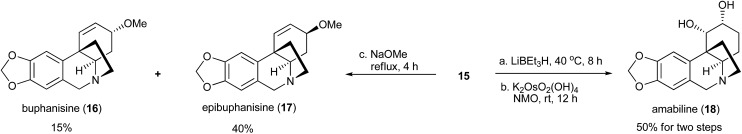
Preparation of buphanisine, amabiline and epibuphanisine.

The chiral allylic chloride **15** can also be used for the synthesis of other crinine-type alkaloids (Fig. 6). Methanolysis of **15** led to the formation of two natural products, buphanisine (**16**)^8h,9b^ and epibuphanisine (**17**).^16^ Alternatively, the reduction of **15** with LiEt_3_BH followed by dihydroxylation led to the formation of amabiline (**18**)^8c,17^ in 50% overall yield. Thus, a series of crinine-type alkaloids were conveniently synthesized using this synthetic route.

### Retrosynthetic analysis of aspidospermidine and minfiensine

**Fig. 7:**
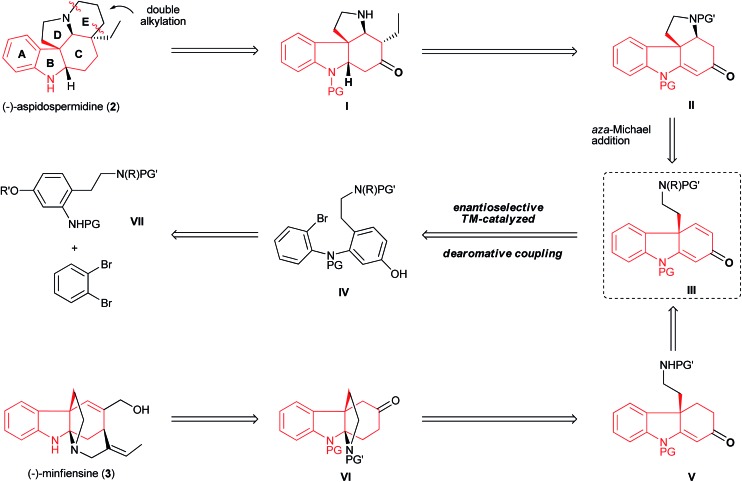
Retrosynthetic analysis of aspidospermidine (**2**) and minfiensine (**3**).

The unique structures of aspidospermidine and minfiensine have attracted considerable synthetic efforts. Although a number of beautiful total syntheses have been reported, the asymmetric syntheses of aspidospermidine^18^ and minfiensine^19^ using enantioselective catalytic methods remain scarce and highly desirable. Despite having different biological origins, both aspidospermidine and minfiensine share a common chiral hydrocarbazole skeleton bearing an all-carbon quaternary stereocenter. We envisioned that a dearomative cyclization of bromo phenol **IV** could lead to the formation of dihydrocarbazolone **III** bearing an all-carbon quaternary center, which could be followed by two ring closures *via* structures **II** and **I** to form (–)-aspidospermidine (**2**) in a concise manner (Fig. 7). The advanced intermediate **VI** in the minfiensine synthesis could be readily afforded from the dearomative cyclization of product **III** *via* intermediate **V**. The bromo phenol substrate **IV** could be readily synthesized from the Buchwald–Hartwig amination of 1,2-dibromobenzene with aniline **VII**.

### Total synthesis of (–)-aspidospermidine

**Fig. 8:**
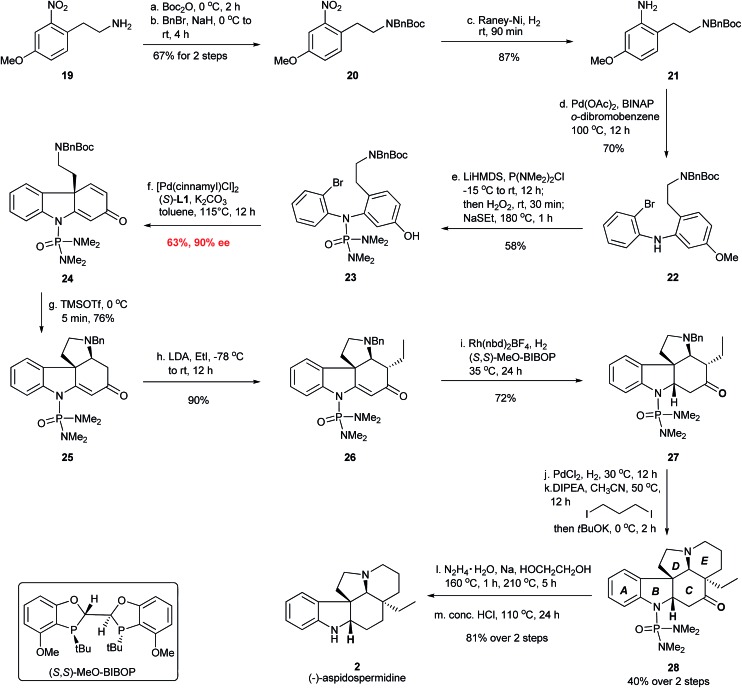
Enantioselective synthesis of (–)-aspidospermidine (**2**).

The total synthesis of (–)-aspidospermidine commenced from the construction of diaryl amine structure **IV** in Fig. 7 for the asymmetric dearomative cyclization. Thus, the installation of the *N*-Boc and *N*-Bn protecting group on the commercially available material **19** formed compound **20**, which was hydrogenated over RANEY® nickel to provide aniline **21** in 58% yield over three steps (Fig. 8). A Pd-catalyzed Buchwald–Hartwig amination of 1,2-dibromobenzene with **21** furnished bromo aniline **22** in 70% yield. The installation of the *N*-P(O)(NMe_2_)_2_ group using LiHMDS/P(NMe_2_)_2_Cl/H_2_O_2_ followed by the deprotection of the MeO group using NaSEt as the reagent afforded the cyclization substrate **23** in 58% yield, which was subjected to the Pd-catalyzed dearomative cyclization. To our delight, the chiral carbazolone **24** possessing an all-carbon quaternary stereocenter was successfully afforded in 63% yield and 90% ee with the Pd-(*S*)-AntPhos catalyst. The *N*-phosphoramide protecting group in **23** again proved to be highly important for the success of the dearomative cyclization.^20^ Treatment of **24** with TMSOTf provided tetracyclic compound **25** in 76% yield through a Boc deprotection and an intramolecular aza-Michael addition. This was followed by the stereoselective installation of the ethyl group at the α-position of the carbonyl group using EtI/LDA to give **26** as a single diastereoisomer in 90% yield. A homogeneous hydrogenation with the Rh-(*S*,*S*)-MeO-BIBOP catalyst^21^ was employed and the reduction of the double bond in **26** took place exclusively at the Re face to afford **27** in 72% yield. After debenzylation of **27** by hydrogenolysis using PdCl_2_ as the catalyst, the construction of the *E* ring in **28** was accomplished through a double-alkylation protocol using I(CH_2_)_3_I/DIPEA/*t*BuOK.^22^ A Wolff–Kishner–Huang Minlon reduction and subsequent acidic hydrolysis successfully delivered (–)-aspidospermidine in 7 steps and 10% overall yield from the key chiral intermediate **24**.

### Formal synthesis of (–)-minfiensine

**Fig. 9:**
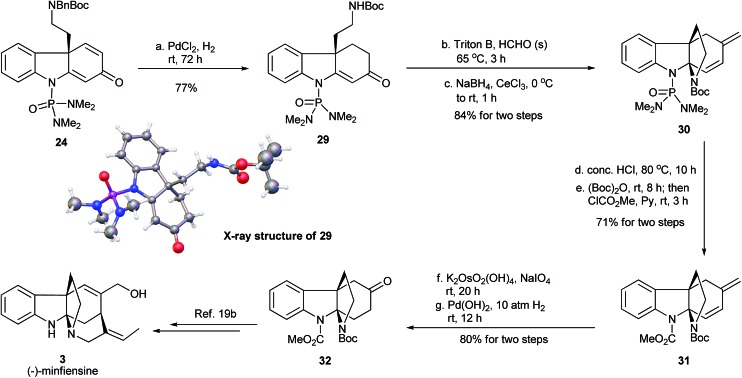
Formal synthesis of (–)-minfiensine.

We envisioned that the enantioenriched dienone **24** could be used for the synthesis of (–)-minfiensine. Thus, the transformation of **24** to the reported key chiral intermediate **32** in the minfiensine synthesis^19*b*^ was conducted (Fig. 9). In order to avoid the intramolecular reaction described in Fig. 8, compound **24** was subjected to hydrogenation over PdCl_2_ to reduce the less-hindered carbon–carbon double bond. The concomitant removal of the *N*-benzyl group led to the formation of compound **29** in 77% yield, whose absolute configuration was confirmed by its X-ray crystal structure.^23^ Next, the cyclization of enone **29** *via* an intramolecular aza-Michael addition was attempted under various acidic or basic conditions and all attempts led to the formation of complex mixtures. Interestingly, the installation of a methylene group at the α position of the carbonyl group using Triton B/paraformaldehyde followed by a Luche reduction cleanly formed the tetracyclic diene **30** in 84% yield. Exchange of the phosphoramide protecting group in **30** to form the methyl carbamate **31** was successfully accomplished by acidic hydrolysis followed by sequential treatment with Boc_2_O and methyl chloroformate. Finally, the Lemieux–Johnson oxidation followed by hydrogenation afforded the chiral ketone **32**, a key advanced intermediate for the synthesis of (–)-minfiensine.^19*b*^ Thus, we have accomplished the formal synthesis of (–)-minfiensine using the enantioselective dearomative cyclization protocol.

## Conclusions

We have established a highly efficient and enantioselective Pd-catalyzed intramolecular dearomative cyclization for the synthesis of two important classes of tricyclic nitrogen-containing skeleton, dihydrophenanthridinone and dihydrocarbazolone derivatives bearing an all-carbon quaternary center. It has been demonstrated that the choice of an *N*-phosphoramide protecting group is essential for the excellent chemoselectivity and the P-chiral monophosphorus ligand AntPhos is crucial for the high reactivity and enantioselectivity. This synthetic method has enabled the enantioselective total syntheses of three distinctive and challenging biologically important polycyclic alkaloids, specifically a concise and gram-scale synthesis of (–)-crinine, an efficient synthesis of indole alkaloid (–)-aspidospermidine and a formal enantioselective synthesis of (–)-minfiensine. This enantioselective dearomative cyclization is expected to be a powerful asymmetric catalytic method for the efficient and scalable syntheses of a number of biologically important natural products, which will certainly facilitate the research and discovery of new therapeutic agents and drugs.
