# Supplementary material for: Efficient syntheses of (–)-crinine and (–)-aspidospermidine, and the formal synthesis of (–)-minfiensine by enantioselective intramolecular dearomative cyclization
Source: Chem Sci. 2017 Jul 3;8(9):6247–56. doi: 10.1039/c7sc01859b (PMC5628388; doi:10.1039/c7sc01859b)
Supplement: Supplementary file 1 [file SC-008-C7SC01859B-s001.pdf]

Electronic Supplementary Material (ESI) for Chemical Science.
This journal is © The Royal Society of Chemistry 2017

Electronic Supporting Information

Efficient Syntheses of (-)-Crinine and (-)-Aspidospermidine, and Formal Synthesis of (-)-Minfiensine by Enantioselective Intramolecular Dearomative Cyclization

Kang Du,[a,+] He Yang,[a,+] Pan Guo, [a,+] Liang Feng,[a] Guangqing Xu,[a] Qinghai Zhou,[b,c] Lung Wa Chung[b] and Wenjun Tang[a,*]

[a] State Key Laboratory of Bio-Organic & Natural Products Chemistry, Shanghai Institute of Organic Chemistry, University of Chinese Academy of Sciences

[b] Department of Chemistry, South University of Science and Technology of China, Shenzhen 518055, China

[c] College of Chemistry, Nankai University, Tianjin 300071, China

Table of Contents

1. **General information……………………………………………………………………….……………….S2**
2. **General procedures for the preparation of cyclization substrates 8a-i………………………….……….......S3**
3. **General procedures for Pd-catalyzed asymmetric dearomative cyclization…………………….….……S11**
4. **Total synthesis of (-)-crinine (1)...…………………………………………………………..…….……S16**
5. **Synthesis of buphanisine (16), epibuphanisine (17) and amabiline (18)…...….…………………...…..……..S25**
6. **Model reactions using diarylamine substrates………………………………………….……..…...…S28**
7. **Total synthesis of aspidospermidine (2)………………...…………………………………………….………S29**
8. **Formal synthesis of minfiensine (3)……………………………………………………………………S41**
9. **Computational results……………………………………………………………………………… S45**
10. **References………………………………………………………………………………………….....S49**
11. **NMR spectra……………………………………………………………………………….…………....S52**

1. General information

All reactions were set-up on the bench top and carried out under nitrogen atmosphere in Schlenk tubes unless otherwise noted. Compounds were purified by flash chromatography using silica gel (200-400 mesh). Anhydrous THF and toluene were distilled from sodium/benzophenone immediately prior to use. All other solvents and reagents were used as received from commercial sources, unless otherwise specified. Known chrial monophosphorus ligands **L1**, **L2, L3, L4** and **L5** from our laboratory were synthesized according to our reported procedures.[1]

^{1}H NMR, ^{31}P NMR, ^{19}F NMR and ^{13}C NMR data were recorded on a Bruker-Ultrashield PLUS 400 NMR or a 500 MHz Agilent spectrometer with $CDCl_3$ or C_6D_6 as the solvent. ^{1}H chemical shifts were referenced to $CDCl_3$ at 7.26 ppm or C_6D_6 at 7.16 ppm. ^{13}C chemical shifts were referenced to $CDCl_3$ at 77 ppm or C_6D_6 at 128 ppm, and obtained with ^{1}H decoupling. ^{31}P chemical shifts were referenced to 85% H_3PO_4 in D_2O at 0.0 ppm as external standard and obtained with ^{1}H decoupling. Multiplicities are abbreviated as follows: singlet (s), doublet (d), triplet (t), quartet (q), doublet-doublet (dd), multiplet (m), and broad (br). MS was measured on Agilent 7890A/5975C Series GC/MSD mass spectrometer or Agilent 1100 Series LC/MSD mass spectrometer. HPLC yields were determined on an Agilent 1200 Infinity Series. Chiral HPLC analyses were performed on an Agilent 1100 Series using a Daicel Chiralpak (AD-H, OD-H) column with hexane/*i*PrOH as the eluent. Optical rotations were measured on a Jacsco P-1010 polarimeter. Melting points were measured with a X-4 microscope melting point apparatus and are uncorrected.

2. General procedures for the preparation of cyclization substrates 8a-i

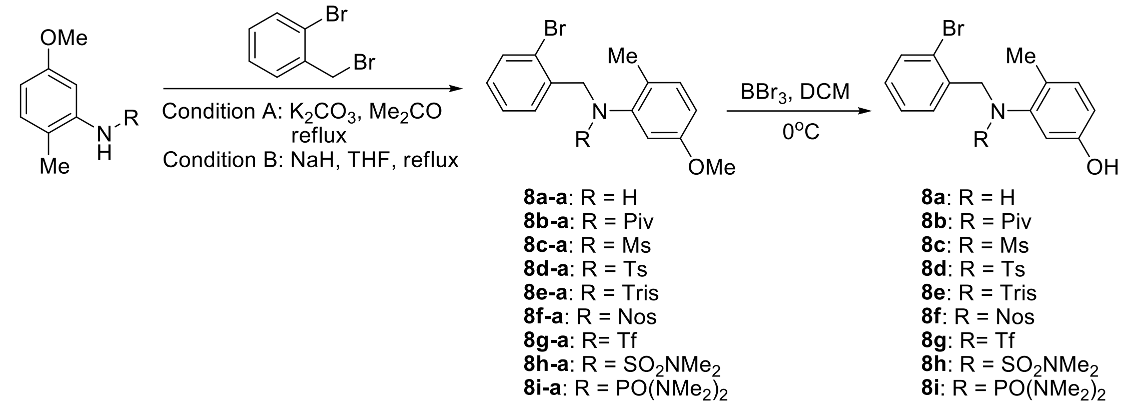

2.1 General procedures for the preparation of compound 8(a-i)-a

Condition A: To a solution of 2-bromobenzyl bromide (1.1 equiv) and *N*-R substituted 5-methoxy-2-methylaniline (1.0 equiv) in acetone at rt was added potassium carbonate (1.2 equiv). The mixture was heated at reflux for 6 h and then concentrated under reduced pressure. Water and EtOAc was added and the organic layer was separated. The aqueous layer was extracted with EtOAc for three times. The combined organic layer was washed with water, brine, dried over sodium sulfate, and concentrated under reduced pressure. The residue was purified by silica gel flash chromatography to afford the desired product.

Condition B: To a solution of 2-bromobenzyl bromide (1.1 equiv) and *N*-R substituted 5-methoxy-2-methylaniline (1.0 equiv) in THF at 0 ^{o}C was added NaH (1.2 equiv) in portions. The resulting mixture was stirred at 0 ^{o}C for 30 min and then heated to reflux for additional 6 h. The reaction mixture was quenched by addition of saturated aqueous NH_4Cl solution and extracted with EtOAc for three times. The combined organic layer was washed with water, brine, dried over sodium sulfate, and concentrated under reduced pressure. The residue was purified by silica gel flash chromatography to afford the desired product.

2.2 General procedures for preparation of compound 8(a-i)

To a solution (0.2 M) of compound **8(a-i)-a** (1.0 equiv) in CH_2Cl_2 at -78 ℃ under nitrogen was added BBr_3 (1.0 equiv) dropwise. The resulting mixture was stirred at rt until the reaction was complete, and then quenched by addition of saturated $NaHCO_3$ solution. The organic layer was separated and the aqueous layer was extracted twice with CH_2Cl_2. The combined organic layer was washed with brine, dried over sodium sulfate and concentrated. The crude product was purified by silica gel flash chromatography to afford the desired phenol **8(a-i)**.

2.3 Characterization data of compounds 8(a-i)-a and 8(a-i)

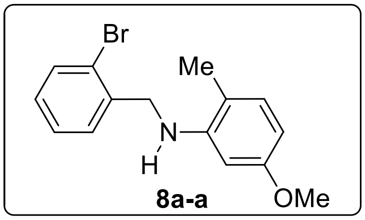

***N*-(2-Bromobenzyl)-5-methoxy-2-methylaniline (8a-a)** The title compound was synthesized according to condition A described in **2.1**. **8a-a:** Colorless oil; ^{1}H NMR (500 MHz, $CDCl_3$) δ 7.66 (d, J = 8.0 Hz, 1H), 7.46 (d, J = 7.7 Hz, 1H), 7.33 (t, J = 7.5 Hz, 1H), 7.20 (t, J = 7.6 Hz, 1H), 7.09 (d, J = 8.1 Hz, 2H), 6.33 (d, J = 8.5 Hz, 1H), 6.23 (s, 1H), 4.50 (s, 2H), 4.18(br s, 1H), 3.79 (s, 3H), 2.23 (s, 3H); ^{13}C NMR (126 MHz, $CDCl_3$) δ 159.5, 146.7, 138.1, 132.9, 130.6, 129.3, 128.8, 127.7, 123.4, 114.7, 110.2, 97.8, 55.2, 48.4, 16.8; ESI-MS: *m/z* 306.2 $[M+H]^+$; HMRS (ESI) calculated for $[M+H, C_{15}H_{17}ONBr]^+$: 306.0489; found: 306.0494.

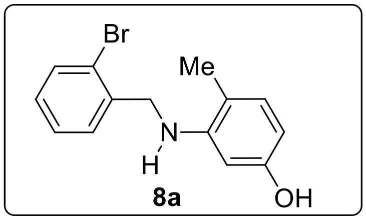

3-((2-Bromobenzyl)amino)-4-methylphenol (8a) The title compound was synthesized according to the general procedure described in **2.2** from **8a-a**. **8a**: White solid; ^{1}H NMR (500 MHz, $CDCl_3$) δ 7.59 (d, J = 7.8 Hz, 1H), 7.36 (d, J = 7.8 Hz, 1H), 7.25 (t, J = 7.5 Hz, 1H), 7.14 (t, J = 7.5 Hz, 1H), 6.92 (d, J = 8.0 Hz, 1H), 6.17 (dd, J = 8.0, 2.5 Hz, 1H), 6.09 (s, 1H), 5.63 (br s, 1H), 4.38 (s, 2H), 2.13 (s, 3H); ^{13}C NMR (126 MHz, $CDCl_3$) δ 155.1, 146.7, 137.9, 132.8, 130.8, 129.4, 128.8, 127.6, 123.4, 114.4,

103.9, 98.4, 48.4, 16.7; ESI-MS: m/z 292.0 $[M+H]^+$; HMRS (ESI) calculated for $[M+H, C_{14}H_{15}ONBr]^+$: 292.0332; found: 292.0337.

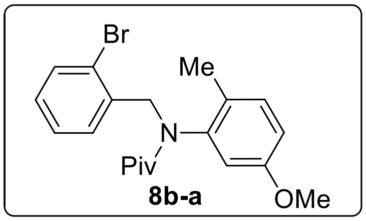

***N*-(2-Bromobenzyl)-*N*-(5-methoxy-2-methylphenyl)pivalamide (8b-a)** The title compound was synthesized according to condition B described in **2.1** from the corresponding starting material. **8b-a:** Colorless oil; ^{1}H NMR (500 MHz, $CDCl_3$) δ 7.41 (d, *J* = 8.0 Hz, 1H), 7.36 (d, *J* = 7.8 Hz, 1H), 7.21 (t, *J* = 7.5 Hz, 1H), 7.09 (d, *J* = 8.5 Hz, 1H), 7.04 (t, *J* = 7.5 Hz, 1H), 6.31 (d, *J* = 2.7 Hz, 1H), 5.57 (d, *J* = 14.8 Hz, 1H), 4.20 (d, *J* = 14.8 Hz, 1H), 3.55 (s, 3H), 2.17 (s, 3H), 1.06 (s, 9H); ^{13}C NMR (126 MHz, $CDCl_3$) δ 177.8, 157.5, 141.9, 136.7, 132.4, 131.5, 130.4, 128.6, 128.3, 127.4, 124.3, 116.0, 114.1, 55.2, 53.5, 41.2, 28.9, 17.1; ESI-MS: m/z 390.3 $[M+H]^+$; HMRS (ESI) calculated for $[M+H, C_{20}H_{25}O_2NBr]^+$: 390.1064; found: 390.1069.

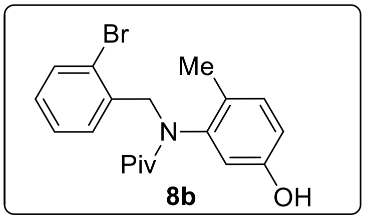

***N*-(2-Bromobenzyl)-*N*-(5-hydroxy-2-methylphenyl)pivalamide (8b)** The title compound was synthesized according to a general procedure described in **2.2** from **8b-a. 8b:** White solid; ^{1}H NMR (500 MHz, $CDCl_3$) δ 7.44 (d, *J* = 8.2 Hz, 1H), 7.35 (d, *J* = 7.8 Hz, 1H), 7.21 (t, *J* = 7.6 Hz, 1H), 7.02-7.08 (m, 2H), 6.70 (dd, *J* = 8.2, 2.6 Hz, 1H), 6.38 (d, *J* = 2.6 Hz, 1H), 5.75 (s, 1H), 5.49 (d, *J* = 14.8 Hz, 1H), 4.24 (d, *J* = 14.8 Hz, 1H), 2.15 (s, 3H), 1.08 (s, 9H); ^{13}C NMR (126 MHz, $CDCl_3$) δ 178.2, 153.9, 142.0, 136.4, 132.5, 131.7, 130.0, 128.6, 128.0, 127.4, 121.2, 117.3, 115.5, 53.9, 41.2, 28.9, 17.1; ESI-MS: m/z 376.1 $[M+H]^+$; HMRS (ESI) calculated for $[M+H, C_{19}H_{23}O_2NBr]^+$: 376.0908; found: 376.0906.

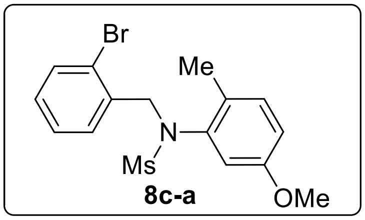

***N*-(2-Bromobenzyl)-*N*-(5-methoxy-2-methylphenyl)methanesulfonamide (8c-a)** The title compound was synthesized according to condition B

described in **2.1** from the corresponding starting material. **8c-a**: Colorless oil; ^{1}H NMR (500 MHz, $CDCl_3$) δ 7.43(d, J = 8.0 Hz, 1H), 7.41 (d, J = 7.8 Hz, 1H), 7.20 (t, J = 7.5 Hz, 1H), 7.07 (t, J = 7.5 Hz, 1H), 7.04 (d, J = 9.0 Hz, 1H), 6.77-6.73 (m, 2H), 4.90 (s, 2H), 3.70 (s, 3H), 3.02 (s, 3H), 2.10 (s, 3H); ^{13}C NMR (126 MHz, $CDCl_3$) δ 158.0, 137.9, 135.0, 132.8, 132.0, 131.8, 131.1, 129.6, 127.5, 124.5, 115.0, 114.2, 55.4, 54.4, 38.8, 17.4; ESI-MS: m/z 383.3 $[M+H]^+$; HMRS (ESI) calculated for $[M+H, C_{16}H_{18}O_3NBrS]^+$: 383.0221; found: 383.0223.

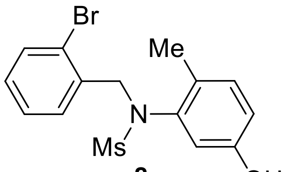

N-(2-Bromobenzyl)-N-(5-hydroxy-2-methylphenyl)methanesulfonamide (8c) The title compound was synthesized according to a general procedure described in **2.2** from **8c-a**. **8c:** White solid; ^{1}H NMR (500 MHz, $CDCl_3$) δ 7.46 (d, J = 8.0 Hz, 1H), 7.40 (d, J = 8.0 Hz, 1H), 7.22 (t, J = 7.5 Hz, 1H), 7.10 (t, J = 7.6 Hz, 1H), 7.01 (d, J = 8.2 Hz, 1H), 6.73-6.67 (m, 2H), 4.90 (br s, 2H), 4.79 (s, 1H), 3.04 (s, 3H), 2.09 (s, 3H); ^{13}C NMR (126 MHz, $CDCl_3$) δ 154.1, 137.8, 134.8, 132.8, 132.0, 131.0, 129.6, 127.4, 124.5, 116.2, 54.4, 39.0, 17.3; ESI-MS: m/z 392.0 $[M+Na]^+$; HMRS (ESI) calculated for $[M+H, C_{15}H_{17}O_3NBrS]^+$: 370.0107; found: 370.0113.

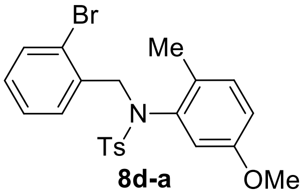

N-(2-Bromobenzyl)-N-(5-methoxy-2-methylphenyl)-4-methylbenzenesulfonamide (8d-a) The title compound was synthesized according to condition B described in **2.1** from the corresponding starting material. **8d-a**: Colorless oil; ^{1}H NMR (500 MHz, $CDCl_3$) δ 7.64 (d, J = 8.5 Hz, 2H), 7.46 (d, J = 7.8 Hz, 1H), 7.39 (d, J = 8.0 Hz, 1H), 7.31 (d, J = 8.0 Hz, 2H), 7.19 (t, J = 7.5 Hz, 1H), 7.05 (t, J = 7.5 Hz, 1H), 6.98 (d, J = 8.5 Hz, 1H), 6.70 (dd, J = 8.4 , 2.7 Hz, 1H), 6.23 (d, J = 2.6 Hz, 1H), 5.04 (br s, 1H), 4.58 (br s, 1H), 3.57 (s, 3H), 2.45 (s, 3H), 1.96 (s, 3H); ^{13}C NMR (126 MHz, $CDCl_3$) δ 157.4, 143.6, 138.0, 135.8, 135.0, 132.6, 131.9, 131.8, 131.5, 129.5, 129.3, 128.1, 127.3, 124.3, 55.2, 54.7, 21.5, 17.3;

ESI-MS: m/z 460.3 [M+H]$^+$; HMRS (ESI) calculated for [M+H, $C_{22}H_{23}O_3NBrS$]$^+$: 460.0577; found: 460.0582.

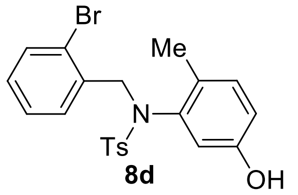

8d

***N*-(2-Bromobenzyl)-*N*-(5-hydroxy-2-methylphenyl)-4-methylbenzenesulfonamide (8d)** The title compound was synthesized according to a general procedure described in **2.2** from **8d-a. 8d:** White solid; ^{1}H NMR (500 MHz, $CDCl_3$) δ 7.64 (d, *J* = 8.2 Hz, 2H), 7.41 (d, *J* = 7.7 Hz, 1H), 7.39 (d, *J* = 8.0 Hz, 1H), 7.30 (d, *J* = 8.5 Hz, 2H), 7.17 (t, *J* = 7.5 Hz, 1H), 7.05 (t, *J* = 7.5 Hz, 1H), 6.91(d, *J* = 8.2 Hz, 1H), 6.63 (dd, *J* = 8.2, 2.7 Hz, 1H), 6.31(d, *J* = 2.7 Hz, 1H), 5.25 (br s, 1H), 5.01 (br s, 1H), 4.55 (br s, 1H), 2.44(s, 3H), 1.90(s, 3H); ^{13}C NMR (126 MHz, $CDCl_3$) δ 153.6, 143.7, 138.0, 135.6, 134.9, 132.6, 131.8, 131.7, 131.5, 129.5, 129.3, 128.0, 127.3, 124.2, 115.9, 115.8, 54.7, 21.6, 17.2; ESI-MS: m/z 468.0 [M+Na]$^+$; HMRS (ESI) calculated for [M+H, $C_{21}H_{21}O_3NBrS$]$^+$: 446.0422; found: 446.0426.

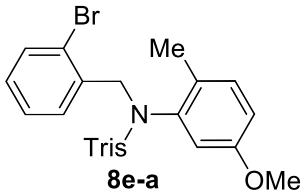

8e-a

***N*-(2-Bromobenzyl)-2,4,6-triisopropyl-*N*-(5-methoxy-2-methylphenyl)benzenesulfonamide (8e-a)** The title compound was synthesized according to condition B described in **2.1** from the corresponding starting material. **8e-a**: Colorless oil; ^{1}H NMR (500 MHz, $CDCl_3$) δ 7.42-7.39 (m, 2H), 7.18 (t, *J* = 7.5 Hz, 1H), 7.12 (s, 2H), 7.05 (t, *J* = 7.6 Hz, 1H), 6.92 (d, *J* = 8.5 Hz, 1H), 6.70 (dd, *J* = 8.5, 2.7 Hz, 1H), 6.58 (d, *J* = 2.7 Hz, 1H), 5.20-4.88 (m, 2H), 3.84-3.78 (m, 2H), 3.52 (s, 3H), 2.93-2.84 (m, 1H), 1.82 (s, 3H), 1.24 (d, *J* = 7.0 Hz, 6H), 1.12 (br s, 12H); ^{13}C NMR (126 MHz, $CDCl_3$) δ 157.7, 152.9, 151.1, 137.0, 135.5, 133.2, 132.6, 132.2, 131.5, 131.4, 129.3, 127.3, 124.9, 123.8, 116.4, 115.6, 55.2, 54.3, 34.1, 30.3, 24.9, 23.6, 17.0; ESI-MS: m/z 594.2 [M+Na]$^+$; HMRS (ESI) calculated for [M+H, $C_{30}H_{39}O_3NBrS$]$^+$: 572.1824; found: 572.1829.

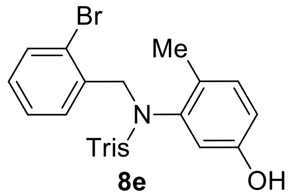

N-(2-Bromobenzyl)-2,4,6-triisopropyl-N-(5-hydroxy-2-methylphenyl)benzenesulfonamide (8e) The title compound was synthesized according to a general procedure described in **2.2** from **8e-a. 8e:** White solid; ^{1}H NMR (500 MHz, $CDCl_3$) δ 7.41 (t, J = 8.0 Hz, 2H), 7.18 (t, J = 7.5 Hz, 1H), 7.10 (s, 2H), 7.06 (t, J = 7.6 Hz, 1H), 6.86 (d, J = 8.5 Hz, 1H), 6.73 (s, 1H), 6.67 (d, J = 2.5 Hz, 1H), 5.15-4.83 (m, 3H), 3.83-3.75 (m, 2H), 2.92-2.84 (m, 1H), 1.69 (s, 3H), 1.23 (d, J = 6.9 Hz, 6H), 1.20-1.10 (m, 12H); ^{13}C NMR (126 MHz, $CDCl_3$) δ 153.8, 152.9, 151.1, 137.0, 135.3, 133.0, 132.6, 132.1, 131.7, 131.2, 129.3, 127.3, 124.8, 123.8, 118.4, 116.1, 54.2, 34.0, 30.4, 24.9, 23.5, 16.8; ESI-MS: m/z 580.1 $[M+Na]^+$; HMRS (ESI) calculated for $[M+H, C_{29}H_{37}O_3NBrS]^+$: 558.1674; found: 558.1678.

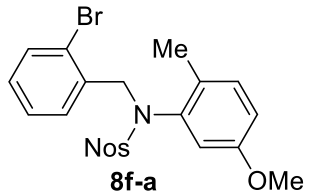

N-(2-Bromobenzyl)-N-(5-methoxy-2-methylphenyl)-4-nitrobenzenesulfonamide(8f-a) The title compound was synthesized according to condition B described in **2.1** from the corresponding starting material. **8f-a**: Light yellow solid; ^{1}H NMR (500 MHz, $CDCl_3$) δ 8.35 (d, J = 8.0 Hz, 2H), 7.93 (d, J = 8.5 Hz, 2H), 7.41 (d, J = 8.0 Hz, 2H), 7.21 (t, J = 7.5 Hz, 1H), 7.09 (t, J = 7.5 Hz, 1H), 7.02 (d, J = 8.5 Hz, 1H), 6.75 (d, J = 8.5 Hz, 1H), 6.22 (d, J = 2.5 Hz, 1H), 5.05 (br s, 1H), 4.69 (br s, 1H), 3.60 (s, 3H), 1.91 (s, 3H); ^{13}C NMR (126 MHz, $CDCl_3$) δ 157.7, 150.1, 144.7, 137.0, 134.2, 132.8, 131.9, 131.3, 129.7, 129.2, 127.4, 124.5, 124.1, 114.7, 114.6, 55.3, 54.9, 17.2; ESI-MS: m/z 515.0 $[M+Na]^+$; HMRS (ESI) calculated for $[M+H, C_{21}H_{20}O_5N_2BrS]^+$: 491.0274; found: 491.0276.

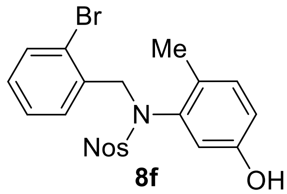

N-(2-Bromobenzyl)-N-(5-hydroxy-2-methylphenyl)-4-nitrobenzenesulfonamide (8f) The title compound was synthesized according to a general procedure described in **2.2** from **8f-a. 8f:** Yellow solid; ^{1}H NMR (500 MHz, $CDCl_3$) δ 8.35 (d, J = 8.5 Hz, 2H), 7.93 (d, J = 8.5 Hz, 2H), 7.41 (t, J = 8.4

Hz, 1H), 7.21 (t, *J* = 7.8 Hz, 1H), 7.10 (t, *J* = 15.0 Hz, 1H), 6.97 (d, *J* = 8.3 Hz, 1H), 6.68 (dd, *J* = 8.3, 2.6 Hz, 1H), 6.24 (d, *J* = 2.7 Hz, 1H), 5.04 (br s, 1H), 4.67 (br s, 1H), 1.88 (s, 3H); ^{13}C NMR (126 MHz, $CDCl_3$) δ 153.7, 150.1, 144.6, 137.1, 134.1, 132.9, 132.1, 131.8, 131.4, 129.8, 129.1, 127.5, 124.4, 124.1, 116.3, 115.9, 54.9, 17.1; ESI-MS: m/z 499.2 $[M+Na]^+$; HMRS (ESI) calculated for $[M+H, C_{20}H_{17}O_2N_5BrS]^+$: 477.2932; found: 477.2928.

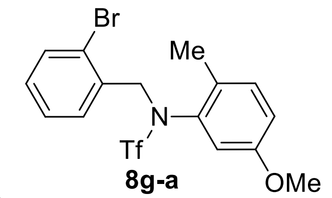

8g-a

***N*-(2-Bromobenzyl)-1,1,1-trifluoro-*N*-(5-methoxy-2-methylphenyl)methanesulfonamide (8g-a)** The title compound was synthesized according to condition A described in **2.1** from the corresponding starting material. **8g-a**: Colorless oil; ^{1}H NMR (400 MHz, $CDCl_3$) δ 7.50 (d, *J* = 8.0 Hz, 1H), 7.40 (d, *J* = 8.0 Hz, 1H), 7.25 (t, *J* = 7.5 Hz, 1H), 7.11 (t, *J* = 7.4 Hz, 1H), 7.01 (d, *J* = 8.5 Hz, 1H), 6.79 (dd, *J* =8.5, 2.7, 1H), 6.67 (s, 1H), 5.13 (d, *J* = 14.2 Hz, 1H), 4.96 (d, *J* = 14.2 Hz, 1H), 3.69 (s, 3H), 1.95 (s, 3H); ^{19}F NMR (376 MHz, $CDCl_3$) δ -73.3; ^{13}C NMR (126 MHz, $CDCl_3$) δ 157.9, 135.3, 133.3, 133.0, 132.4, 131.9, 130.8, 130.2, 127.7, 125.1, 120.3 (q, *J* =325.2 Hz), 115.6, 115.2, 55.8, 55.4, 17.0; ESI-MS: m/z 460.0 $[M+Na]^+$; HMRS (ESI) calculated for $[M+H, C_{16}H_{16}O_3NBrF_3S]^+$: 437.9979; found: 437.9981.

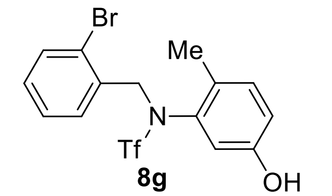

8g

***N*-(2-Bromobenzyl)-1,1,1-trifluoro-*N*-(5-hydroxy-2-methylphenyl)methanesulfonamide (8g)** The title compound was synthesized according to a general procedure described in **2.1** from **8g-a. 8g:** White solid; ^{1}H NMR (500 MHz, $CDCl_3$) δ 7.49 (d, *J* = 7.7 Hz, 1H), 7.43 (d, *J* = 8.0 Hz, 1H), 7.28 (t, *J* = 7.4 Hz, 1H), 7.15 (t, *J* = 7.5 Hz, 1H), 6.98 (d, *J* = 8.2 Hz, 1H), 6.73-6.67 (m, 2H), 5.14 (d, *J* = 14.2 Hz, 1H), 5.08 (s, 1H), 4.95 (d, *J* = 14.2 Hz, 1H), 1.96 (s, 3H); ^{13}C NMR (126 MHz, $CDCl_3$) δ 153.9, 135.4, 133.2, 133.0, 132.3, 132.1, 131.0, 130.3, 127.7, 125.0, 120.3 (q, *J* = 319.2 Hz), 117.0, 116.7, 55.9, 17.0; ^{19}F NMR(376 MHz, $CDCl_3$) δ -73.3; ESI-MS: m/z 422.9 $[M]^+$; HMRS (ESI) calculated for [M+H,

$C_{15}H_{14}O_3NBrF_3S]^+$: 423.9828; found: 423.9830.

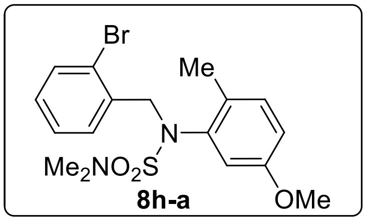

***N*-(2-Bromobenzyl)-*N*-(5-methoxy-2-methylphenyl)dimethylaminosulfonamide (8h-a)** The title compound was synthesized according to condition B described in **2.1** from the corresponding starting material. **8h-a**: Colorless oil; ^{1}H NMR (500 MHz, $CDCl_3$) δ 7.44 (d, J = 8.0 Hz, 2H), 7.30 (d, J = 8.2 Hz, 1H), 7.17 (t, J = 7.6 Hz, 1H), 7.07 (t, J = 7.6 Hz, 1H), 7.00 (d, J = 8.5 Hz, 1H), 6.92 (d, J = 2.7 Hz, 2H), 6.73 (dd, J = 8.2, 2.7 Hz, 1H), 4.83 (br s, 2H), 3.73 (s, 3H), 2.87 (s, 6H), 2.07 (s, 3H); ^{13}C NMR (126 MHz, $CDCl_3$) δ 157.8, 139.1, 135.1, 132.7, 132.1, 131.5, 130.6, 129.4, 127.2, 124.7, 114.8, 114.1, 55.4, 38.5, 17.2; ESI-MS: m/z 413.3 $[M+H]^+$; HMRS (ESI) calculated for $[M+H, C_{17}H_{22}O_3N_2BrS]^+$: 413.0531; found: 413.0528.

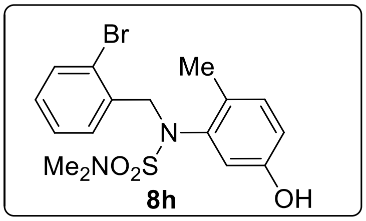

***N*-(2-Bromobenzyl)-*N*-(5-hydroxy-2-methylphenyl)dimethylaminosulfonamide (8h)** The title compound was synthesized according to a general procedure described in **2.2** from **8h-a. 8h:** White solid; ^{1}H NMR (500 MHz, $CDCl_3$) δ 7.44 (d, J = 8.0 Hz, 1H), 7.22 (d, J = 8.0 Hz, 1H), 7.13 (t, J = 7.5 Hz, 1H), 7.06 (t, J = 7.5 Hz, 1H), 6.95 (d, J = 2.7 Hz, 1H), 6.92 (d, J = 8.4 Hz, 1H), 6.67 (dd, J = 8.3, 2.6 Hz, 1H), 5.78 (br s, 1H), 4.80 (br s, 2H), 2.88 (s, 6H), 2.00 (s, 3H) ; ^{13}C NMR (126 MHz, $CDCl_3$) δ 154.2, 138.9, 134.9, 132.7, 132.2, 131.6, 130.3, 129.4, 127.2, 124.7, 116.0, 115.8, 55.5, 38.5, 17.1; ESI-MS: m/z 399.0 $[M+H]^+$; HMRS (ESI) calculated for $[M+H, C_{16}H_{20}O_3N_2BrS]^+$: 399.0373; found: 399.0378.

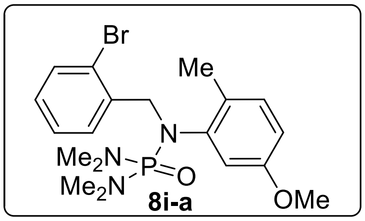

***N*-(2-Bromobenzyl)-*N*-(5-methoxy-2-methylphenyl)-*N,N,N',N'*-tetramethyl-1-oxophosphoranetriamine(8i-a)** The title compound was synthesized according to condition B described in **2.1** from the corresponding starting

material. **8i-a**: Colorless oil; ^{1}H NMR (500 MHz, $CDCl_3$) δ 7.89 (d, J = 7.7 Hz, 1H), 7.31 (d, J = 7.8 Hz, 1H), 7.22 (t, J = 7.4 Hz, 1H), 7.02-6.92 (m, 2H), 6.64-6.57 (m, 2H), 4.66 (d, J = 10.5 Hz, 2H), 3.60 (s, 3H), 2.53 (d, J = 9.1 Hz, 12H), 2.24 (s, 3H) ; ^{13}C NMR (126 MHz, $CDCl_3$) δ 157.4, 141.5, 137.0, 132.9, 132.2, 131.2, 129.0, 128.6, 127.2, 124.7, 116.0 (d, J = 2.5 Hz), 112.7, 55.3, 52.9, 37.3 (d, J = 15.1 Hz), 17.7; ^{31}P NMR (162 MHz, $CDCl_3$) δ 22.1; ESI-MS: m/z 440.1 $[M+H]^+$; HMRS (ESI) calculated for $[M+H, C_{19}H_{28}O_2N_3BrP]^+$: 440.1098; found: 440.1097.

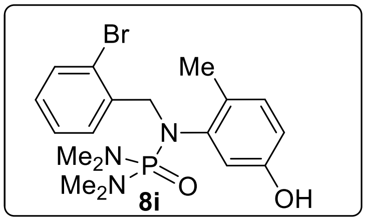

***N*-(2-Bromobenzyl)-*N*-(5-hydroxy-2-methylphenyl)-*N,N,N',N'*-tetramethyl-1-oxophosphoranetriamine (8i)** The title compound was synthesized according to a general procedure described in **2.2** from **8i-a. 8i:** White solid; ^{1}H NMR (500 MHz, $CDCl_3$) δ 7.67 (d, J = 7.7 Hz, 1H), 7.32 (d, J = 7.7 Hz, 1H), 7.21 (s, 1H), 7.00-6.94 (m, 2H), 6.88 (t, J = 7.5 Hz, 1H), 6.64 (dd, J = 8.3, 2.6 Hz, 1H), 4.60 (d, J = 11.5 Hz, 2H), 2.57 (s, 12H), 2.23 (s, 3H); ^{13}C NMR (126 MHz, $CDCl_3$) δ 156.3, 140.8, 136.4, 132.5, 132.2, 131.4, 128.5, 127.0, 125.4 (d, J = 6.6 Hz), 124.4, 116.8, 114.2, 53.2, 37.6 (d, J = 15.1 Hz), 17.8; ^{31}P NMR (162 MHz, $CDCl_3$) δ 22.0; ESI-MS: m/z 426.1 $[M+H]^+$; HMRS (ESI) calculated for $[M+H, C_{18}H_{26}O_2N_3BrP]^+$: 426.0928; found: 426.0936.

3. General procedures for Pd-catalyzed asymmetric dearomative cyclization

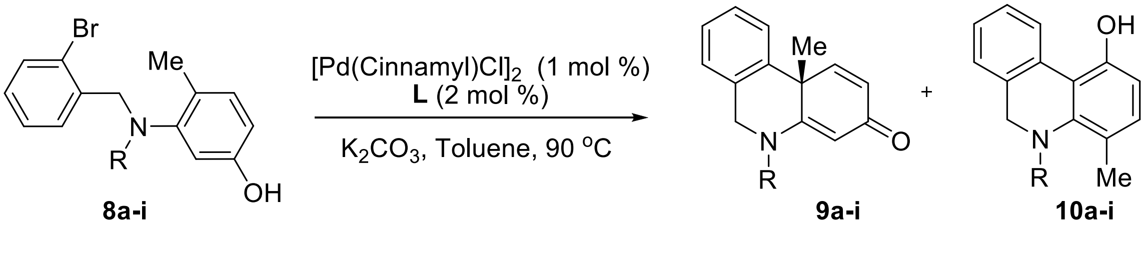

To a flame-dried Schlenk tube equipped with a magnetic stirring bar was charged substrate **8** (0.10

mmol, 1.0 equiv), [Pd(cinnamyl)Cl]$_2$ (0.001 mmol, 0.01 equiv), **L** (0.002 mmol, 0.02 equiv), K_2CO_3 (0.15 mmol, 1.5 equiv). The mixture was evacuated and back-filled with nitrogen for three times. Toluene (10 mL) was added via syringe and the resulting mixture was stirred at 90 ℃ for 16 h, and then quenched with EtOAc (5 mL). The mixture was filtered through a pad of Celite and concentrated. The product was purified by silica gel flash chromatography. The enantiomeric excess was determined by chiral HPLC on a Chiralcel OD-H or Chiralpak AD-H column.

Characterization data of compounds 9c-i

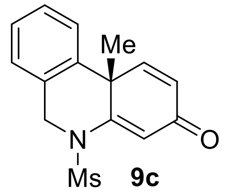

9c

(*R*)-10b-Methyl-5-(methylsulfonyl)-5,6-dihydrophenanthridin-3(10*bH*)-one (9c): white solid (16 %); m.p. 74-77 ºC; 95% ee; Enantiomeric excess was determined by chiral HPLC (Chiralcel OD-H, 25 ºC, flow rate: 1 mL/min, hexane/isopropanol: 70/30, 254 nm, 21.2 min (*S*), 22.7 min (*R*); the absolute configurations of **9c** was assigned by analogy on the basis of compound **13**. $[\alpha]_D^{25}$ = -65.3 (c = 1.2, $CHCl_3$); ^{1}H NMR (500 MHz, $CDCl_3$) δ 7.43 (d, *J* = 10.2 Hz, 1H), 7.40-7.31 (m , 3H), 7.27 (d, *J* = 10.2 Hz, 1H), 6.47 (d, *J* = 1.6 Hz, 1H), 6.41 (dd, *J* = 10.2, 1.6 Hz, 1H), 5.00 (d, *J* = 16.0 Hz, 1H), 4.94 (d, *J* = 16.0 Hz, 1H), 3.16 (s, 3H), 1.57 (s, 3H); ^{13}C NMR (126 MHz, $CDCl_3$) δ 185.2, 156.6, 147.7, 136.9, 130.3, 128.7, 128.1, 127.3, 126.9, 123.9, 112.7, 49.7, 43.2, 38.6, 26.9; ESI-MS: m/z 290.1 $[M+H]^+$; HMRS (ESI) calculated for [M+H, $C_{15}H_{16}O_3NS]^+$: 290.0845; found: 290.0845.

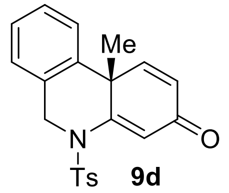

9d

(*R*)-10b-Methyl-5-tosyl-5,6-dihydrophenanthridin-3(10*bH*)-one (9d): white solid (61%); m.p. 102-104 ºC; 91% ee; Enantiomeric excess was determined by chiral HPLC (Chiralcel OD-H, 25 ºC, flow rate: 1 mL/min, hexane/isopropanol: 70/30, 254 nm, 13.00 min (*S*), 16.62 min (*R*)); the

absolute configurations of **9d** was assigned by analogy on the basis of compound **13**. $[\alpha]_D^{26}$ = -129.6 (c = 1.5, $CHCl_3$); ^{1}H NMR (500 MHz, $CDCl_3$) δ 7.80 (d, *J* = 8.3 Hz, 2H), 7.33-7.26 (m, 7H), 6.61 (s, 1H), 6.31 (dd, *J* = 10.2, 1.5 Hz, 1H), 5.14 (d, *J* = 15.6 Hz, 2H), 4.92 (d, *J* = 15.6 Hz, 2H), 2.41 (s, 3H), 1.10 (s, 3H); ^{13}C NMR (126 MHz, $CDCl_3$) δ 187.6, 158.0, 150.0, 147.2, 139.3, 136.9, 132.6, 132.1, 130.8, 130.2, 129.9, 129.5, 129.2, 126.0, 116.6, 52.3, 45.3, 28.2, 23.8; ESI-MS: m/z 366.2 $[M+H]^+$; HMRS (ESI) calculated for $[M+H, C_{21}H_{20}O_3NS]^+$: 366.1157; found: 366.1158.

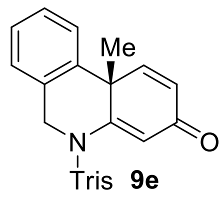

(*R*)-10b-Methyl-5-((2,4,6-triisopropylphenyl)sulfonyl)-5,6-dihydrophenanthridin-3(10*bH*)-one (9e): white solid (54%); m.p. 131-133 °C; 90% ee; Enantiomeric excess was determined by chiral HPLC (Chiralcel OD-H, 25 °C, flow rate: 1 mL/min, hexane/isopropanol: 70/30, 254 nm, 8.77 min (*S*), 9.70 min (*R*); the absolute configurations of **9e** was assigned by analogy on the basis of compound **13**. $[\alpha]_D^{23}$ = -93.2 (c = 0.5, $CHCl_3$); ^{1}H NMR (500 MHz, $CDCl_3$) δ 7.38 (d, *J* = 10.2 Hz, 1H), 7.36-7.28 (m, 3H), 7.25 (d, *J* = 10.2 Hz, 1H), 7.20 (s, 2H), 6.32 (d, *J* = 10.2 Hz, 1H), 6.05 (s, 1H), 5.03 (d, *J* = 15.6 Hz, 1H), 4.88 (d, *J* = 15.5 Hz, 1H), 4.06-3.97 (m, 2H), 2.95-2.86 (m, 1H), 1.56 (s, 3H), 1.28 (d, *J* = 8.6 Hz, 6H), 1.26 (d, *J* = 6.9 Hz, 6H), 1.14 (d, *J* = 6.8 Hz, 6H); ^{13}C NMR (126 MHz, $CDCl_3$) δ 185.0, 157.0, 154.4, 151.0, 147.8, 137.4, 132.3, 130.9, 128.6, 127.8, 127.2, 126.9, 124.5, 123.9, 113.7, 48.8, 43.3, 34.1, 29.5, 26.8, 24.8, 24.4, 23.4 (d, *J* = 5.7 Hz); ESI-MS: m/z 478.2 $[M+H]^+$; HMRS (ESI) calculated for $[M+H, C_{29}H_{36}O_3NS]^+$: 478.2406; found: 478.2410.

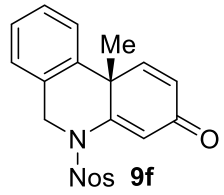

(*R*)-10b-Methyl-5-((4-nitrophenyl)sulfonyl)-5,6-dihydrophenanthridin-3(10*bH*)-one (9f): white film (37%); 86% ee; Enantiomeric excess was

determined by chiral HPLC (Chiralpak AD-H, 25 °C, flow rate: 1 mL/min, hexane/isopropanol: 90/10, 254 nm, 8.02 min (*S*), 9.98 min (*R*). the absolute configurations of **9f** was assigned by analogy on the basis of compound **13**. $[\alpha]_D^{25}$ = -104.5 (c = 1.0, $CHCl_3$); ^1H NMR (500 MHz, $CDCl_3$) δ 8.37 (d, *J* = 8.6 Hz, 2H), 8.11 (d, *J* = 9.0 Hz, 2H), 7.36-7.26 (m, 5H), 6.59 (s, 1H), 6.34 (dd, *J* = 10.2, 1.6 Hz, 1H), 5.16 (d, *J* = 15.5 Hz, 1H), 4.97 (d, *J* = 15.5 Hz, 1H), 1.12 (s, 3H) ; ^{13}C NMR (126 MHz, $CDCl_3$) δ 185.0, 155.0, 150.6, 148.0, 143.2, 136.8, 129.6, 128.9, 128.8, 128.2, 127.3, 127.0, 124.5, 123.9, 115.6, 50.3, 43.1, 26.1; ESI-MS: m/z 397.0 $[M+H]^+$; HMRS (ESI) calculated for $[M+H, C_{20}H_{17}O_5N_2S]^+$: 397.0853; found: 397.0853.

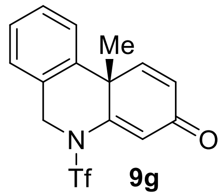

9g

(*R*)-10b-Methyl-5-((trifluoromethyl)sulfonyl)-5,6-dihydrophenanthridin-3(10*bH*)-one (9g): white foam (70%); 84% ee; Enantiomeric excess was determined by chiral HPLC (Chiralcel OD-H, 25 °C, flow rate: 1 mL/min, hexane/isopropanol: 80/20, 254 nm, 21.23 min (*S*), 22.69 min (*R*). the absolute configurations of **9g** was assigned by analogy on the basis of compound **13**. $[\alpha]_D^{25}$ = -67.5 (c = 1.2, $CHCl_3$); ^1H NMR (400 MHz, $CDCl_3$) δ 7.48 (d, *J* = 10.2 Hz, 1H), 7.42-7.30 (m, 3H), 7.21 (d, *J* = 10.2 Hz, 1H), 6.51 (s, 1H), 6.43 (d, *J* = 1.6 Hz, 2H), 5.15 (d, *J* = 15.5 Hz, 1H), 4.93 (d, *J* = 15.5 Hz, 1H), 1.62 (s, 3H), 1.59 (s, 3H); ^{13}C NMR (126 MHz, $CDCl_3$) δ 185.0, 153.8, 149.2, 136.9, 129.0, 128.8, 128.2, 127.5, 126.7, 124.3, 120.6, 120.0 (q, *J* = 325.3), 51.7, 43.1, 27.7; ^{19}F NMR (376 MHz, $CDCl_3$) δ -73.9; ESI-MS: m/z 366.0 $[M+Na]^+$; HMRS (ESI) calculated for $[M+H, C_{15}H_{13}O_3NF_3S]^+$: 344.0563; found: 344.0563.

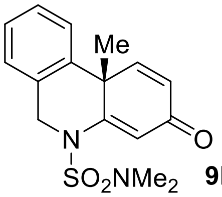

9h

(*R*)-10b-Methyl-5-(*N*,*N*-dimethysulfonyl)-5,6-dihydrophenanthridin-3(10*bH*)-one (9h): white film (73%); 94% ee; Enantiomeric excess was

determined by chiral HPLC (Chiralcel OD-H, 25 °C, flow rate: 1 mL/min, hexane/isopropanol: 80/20, 254 nm, 10.35 min (*S*), 15.38 min (*R*); the absolute configurations of **9c** was assigned by analogy on the basis of compound **13**. $[\alpha]_D^{25}$ = -90.0 (c = 0.65, $CHCl_3$); ^1H NMR (500 MHz, $CDCl_3$) δ 7.40 (d, J = 10.0 Hz, 1H), 7.38-7.28 (m, 3H), 7.23 (d, J = 7.2 Hz, 1H), 6.38 (d, J = 10.6 Hz, 1H), 6.35 (s, 1H), 5.00 (d, J = 16.0 Hz, 1H), 4.90 (d, J = 16.0 Hz, 1H), 2.93 (s, 6H), 1.56 (s, 3H); ^{13}C NMR (126 MHz, $CDCl_3$) δ 185.4, 157.0, 147.6, 137.0, 130.8, 128.6, 127.9, 127.3, 126.8, 123.8, 112.4, 51.5, 43.2, 38.2, 27.3; ESI-MS: m/z 319.0 $[M+H]^+$; HMRS (ESI) calculated for $[M+H, C_{16}H_{19}O_3N_2S]^+$: 319.1110; found: 319.1111.

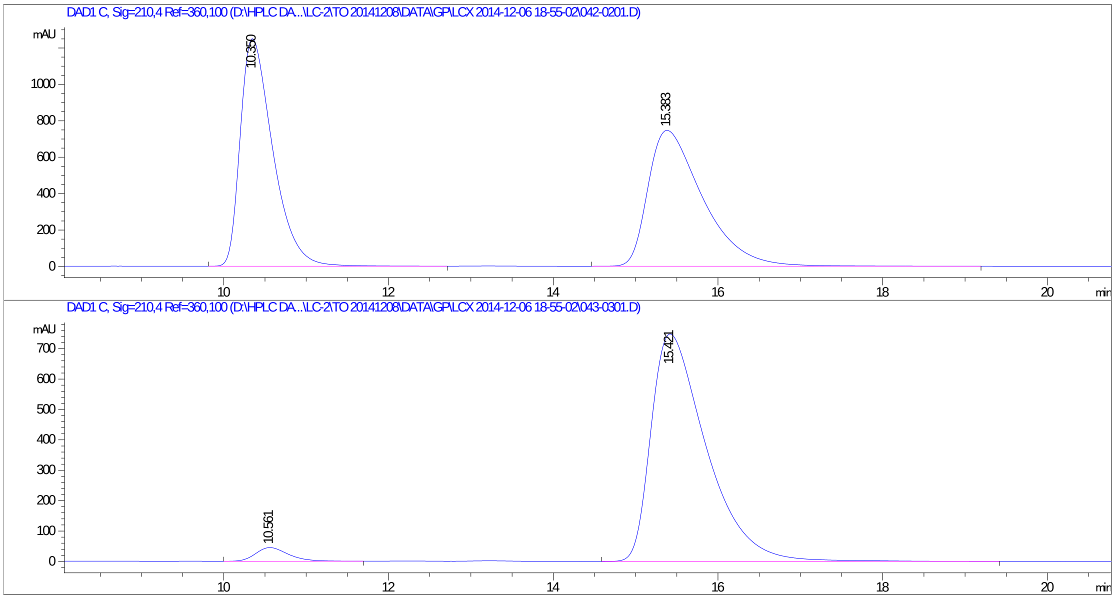

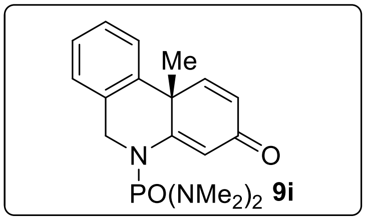

(*R*)-10b-Methyl-5-(*N*,*N*,*N'*,*N'*-tetramethyl-1-oxophosphyl)-5,6-dihydrophenanthridin-3(10*bH*)-one (9i): white film (96%); 96% ee; Enantiomeric excess was determined by chiral HPLC (Chiralpak AD-H, 25 °C, flow rate: 1 mL/min, hexane/isopropanol: 70/30, 254 nm, 5.64 min (*R*), 9.28 min (*S*); the absolute configurations of **9i** was assigned by analogy on the basis of compound **13**. $[\alpha]_D^{25}$ = -88.9 (c = 0.5, $CHCl_3$); ^1H NMR (500

MHz, $CDCl_3$) δ 7.38 (d, J = 10.2 Hz, 1H), 7.34 (t, J = 16.8 Hz, 1H), 7.31-7.26 (m ,2H), 7.22 (d, J = 7.1 Hz, 1H), 6.36 (dd, J = 10.2, 1.5 Hz, 1H), 5.91 (s, 1H), 4.90 (d, J = 16.7 Hz, 1H), 4.70 (dd, J = 16.7, 4.3 Hz, 1H), 2.74 (d, J = 9.4 Hz, 6H), 2.68 (d, J = 10.5 Hz, 6H), 1.53 (s, 3H); ^{31}P NMR (162 MHz, $CDCl_3$) δ 18.2; ^{13}C NMR (126 MHz, $CDCl_3$) δ 185.7, 162.0, 147.5, 137.5, 132.1 (d, J = 6.1 Hz), 128.2, 127.7, 127.3, 126.6, 123.6, 110.1 (d, J = 2.5 Hz), 50.3 (d, J = 4.3Hz), 43.2, 37.7 (d, J = 4.7Hz), 36.4 (d, J = 4.0 Hz), 26.3; ESI-MS: m/z 346.1 $[M+H]^+$; HMRS (ESI) calculated for $[M+H, C_{18}H_{25}O_2N_3P]^+$: 346.1679; found: 346.1679.

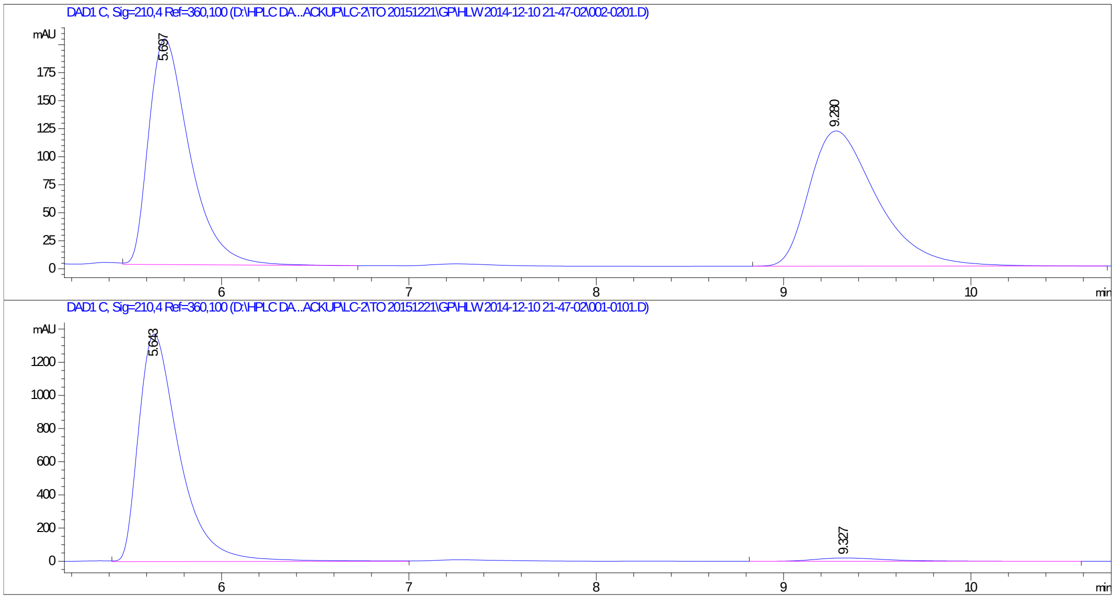

4. Total synthesis of (-)-Crinine

4.1 Synthesis of aniline 7

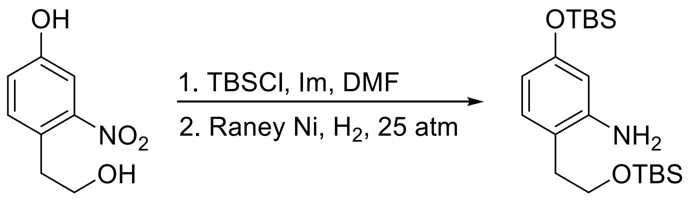

7

5-((*tert*-Butyldimethylsilyl)oxy)-2-(2-((*tert*-butyldimethylsilyl)oxy)ethyl)aniline (7): To a stirred solution of 4-(2-hydroxyethyl)-3-nitrophenol (30 g, 0.16 mol, 1.0 equiv) (reference: H. M. Woodburn, C. F. Stuntz, *J. Am. Chem. Soc.* **1950**, *72*, 1361) and imidazole (33 g, 0.48 mol, 3.0 equiv) in DMF (200 mL) at 0 °C was added TBSCl (74 g, 0.48 mol, 3.0 equiv) over 10 min. The resulting mixture was stirred at rt for 2 h and then concentrated under reduced pressure. Water (200 mL) and EtOAc (200 mL) was added and the organic layer was separated. The aqueous layer was extracted with EtOAc (200 mL × 3). The combined organic layer was washed with water (200 mL × 3), brine (200 mL), dried over sodium sulfate, and concentrated to afford the crude TBS-protected product as light yellow oil (65 g), which was used for the next step without further purification.

To a solution of the above-obtained oil in MeOH (500 mL) was added Raney Ni (6.5 g) at room temperature. The mixture was stirred at 40 °C under 25 atm of hydrogen pressure in a Parr apparatus for 6 h and then filtered through a pad of Celite. The Celite pad was washed with MeOH (50 mL × 3). The combined filtrate was concentrated under reduced pressure to give a colorless oil. The residue was purified by silica gel column chromatography (eluent: *n*-Hexane/EtOAc = 30/1) to afford aniline **7** (50.0 g, 0.13 mol, 79% yield over two steps) as colorless oil. ^{1}H NMR (500 MHz, $CDCl_3$) δ 6.85 (d, *J* = 8.1 Hz, 1H), 6.24 (dd, *J* = 8.1, 2.4 Hz, 1H), 6.21 (d, *J* = 2.3 Hz, 1H), 3.94 (br s, 2H), 3.84 (t, *J* = 12.2 Hz, 2H), 2.72 (t, *J* = 12.2 Hz, 2H), 1.01 (s, 9H), 0.90 (s, 9H), 0.21 (s, 6H), 0.00 (s, 6H); ^{13}C NMR (126 MHz, $CDCl_3$) δ 155.0,

146.6, 131.1, 118.4, 110.4, 107.6, 64.7, 34.6, 26.0, 25.8, 18.3, 18.2, -4.3, -5.5; ESI-MS: *m/z* 382.5 $[M+H]^+$; HMRS (ESI) calculated for $[M+H, C_{20}H_{40}NO_2Si_2]^+$: 382.2598; found: 382.2586.

4.2 Total synthesis of crinine from aniline 7 and aldehyde 6

Preparation of aniline 11:

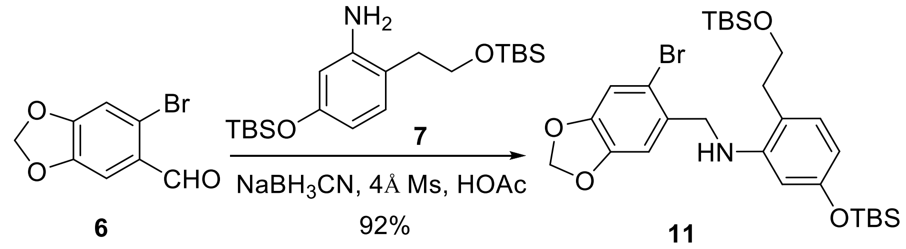

To a stirred solution of aniline **7** (50.0 g, 131 mmol), 6-bromopiperonal **6** (33.0 g, 144 mmol), 4Å MS (5.0 g) and AcOH (7.5 mL, 131 mmol) in DCM (500 mL) at 0 ºC was added in portions $NaBH_3CN$ (10.7 g, 17.0 mmol) over 10 min. The resulting mixture was stirred at 0 ºC for 30 min and was then allowed to stir at rt for 1 h. After aniline **7** was completely consumed, the reaction mixture was quenched by addition of saturated NH_4Cl solution (200 mL). The organic layer was separated and the aqueous layer was extracted with DCM (200 mL×3). The combined organic layer was washed with water (200 mL×3), brine (200 mL), dried over sodium sulfate, concentrated, and purified by silica gel column chromatography (eluent: *n*-Hexane/EtOAc = 30/1) to afford aniline **11** (71.0 g, 92% yield) as a colorless oil. ^1H NMR (500 MHz, $CDCl_3$) δ 7.03 (s, 1H), 6.92 (s, 1H), 6.87 (d, *J* = 8.0 Hz, 1H), 6.21-6.17 (m, 1H), 5.95-5.91 (m, 3H), 5.06 (br s, 1H), 4.28 (s, 2H), 3.87 (t, *J* = 6.1 Hz, 2H), 2.78 (t, *J* = 6.1 Hz, 2H), 0.94 (s, 9H), 0.89 (s, 9H), 0.10 (s, 6H), 0.02 (s, 6H); ^{13}C NMR (126 MHz, $CDCl_3$) δ 155.3, 147.2, 147.6, 147.1, 131.7, 130.9, 118.3, 113.0, 113.7, 108.9, 103.8, 101.6, 64.9, 47.9, 35.0, 26.0, 25.8, 18.4, 18.3, -4.5, -5.4; ESI-MS: *m/z* 594.5 $[M+H]^+$; HMRS (ESI) calculated for $[M+H, C_{28}H_{45}BrNO_4Si_2]^+$: 594.2070; found: 594.2050.

Preparation of phenol 12

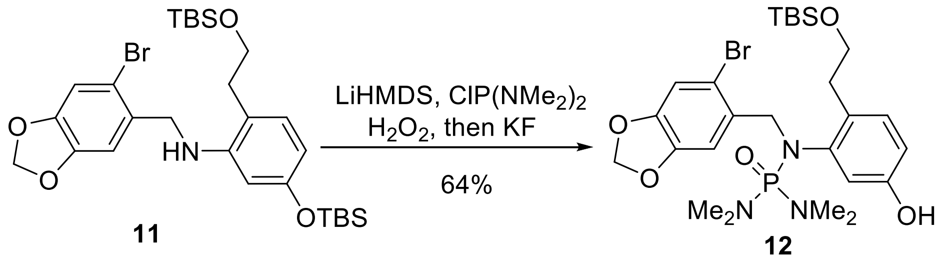

To a flame-dried Schlenk flask equipped with a magnetic stirring bar was added aniline **11** (30.0 g, 50 mmol) and THF (300 mL). LiHMDS (75 mL, 75 mmol, 1.5 equiv, 1.0 M in *n*-hexane) was added dropwise over 30 min at -10 °C and the resulting mixture was stirred at 0 °C for 2 h. $ClP(NMe_2)_2$ (11.0 mL, 75 mmol, 1.5 equiv) was added dropwise and the resulting mixture was allowed to warm to rt and stirred overnight. To the mixture at 0 °C was added 30% H_2O_2 (20 mL) and the resulting mixture was further stirred at the same temperature for 30 min. A saturated $NaHSO_3$ solution (100 mL) was added carefully to quench the excess H_2O_2. The organic layer was separated and the aqueous layer was extracted with EtOAc (100 mL×3). The combined organic phases were washed with brine, dried over sodium sulfate, filtered and concentrated under reduced pressure to afford light yellow oil, which was used for the next operation without further purification.

To a flame-dried Schlenk flask equipped with a magnetic stirring bar was added potassium fluoride (4.4 g, 75 mmol, 1.5 equiv) and tetraethylene glycol (300 mL). The reaction mixture was stirred at room temperature for 15 min. To the mixture was added the aforementioned yellow oil in EtOAc (5 mL) and the resulting mixture was stirred at rt overnight, and then quenched with water (300 mL) and EtOAc (400 mL×3). The organic phase was separated and the aqueous phase was washed with EtOAc (200 mL×3). The combined organic phase was washed with water (200 mL×3), brine (200 mL), dried over sodium sulfate, and concentrated under reduced pressure to afford crude phenol **12** (27.4 g) as a yellow solid, which was further recrystallized from *n*-hexane/DCM to afford pure **12** as white crystalline solid (19.7 g, 64%). m.p.

111-113 °C; ^{1}H NMR (500 MHz, $CDCl_3$) δ 10.41 (br s, 1H), 7.27 (s, 1H), 7.10 (s, 1H), 7.07 (d, *J* = 8.5 Hz, 1H), 6.79 (s, 1H), 6.69 (dd, *J* = 8.4, 2.4 Hz, 1H), 5.70 (s, 2H), 4.51 (d, *J* = 11.0 Hz, 2H), 3.80 (t, *J* = 7.3 Hz, 2H), 2.85 (t, *J* = 6.2 Hz, 2H), 2.58 (br s, 12H), 0.89 (s, 9H), 0.04 (s, 6H); ^{31}P NMR (162 MHz, $CDCl_3$) δ 21.3; ^{13}C NMR (126 MHz, $CDCl_3$) δ 156.6, 147.4, 147.0, 141.4, 129.8, 126.3, 116.8, 114.8, 114.3, 112.0, 111.9, 101.4, 63.3, 54.5, 37.7, 33.2, 26.0, 18.3, -5.3; ESI-MS: *m/z* 614.5 $[M+H]^+$; HMRS (ESI) calculated for $[M+H, C_{26}H_{42}BrN_3O_5PSi_2]^+$: 614.1815; found: 614.1810.

Preparation of cyclization product 13

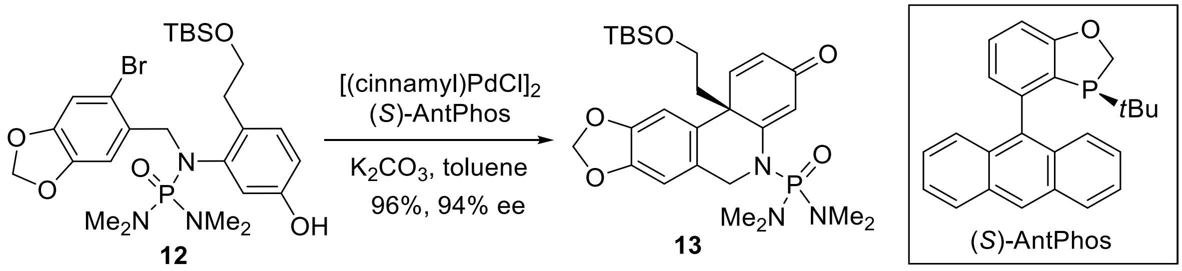

To a flame-dried Schlenk flask equipped with a magnetic stirring bar was charged phenol **12** (922 mg, 1.5 mmol), $[Pd(cinnamyl)Cl]_2$ (7.8 mg, 0.015 mmol, 1 mol %), (*S*)-AntPhos (11.1 mg, 0.030 mmol, 2 mol %), and K_2CO_3 (414 mg, 3.0 mmol, 2 equiv). The mixture was pumped and back-filled with nitrogen for three times. Toluene (15 mL) was added via syringe and the resulting mixture was stirred at 110 ℃ for 12 h and then diluted with EtOAc (30 mL). The mixture was filtered over a pad of Celite and the filtrate was concentrated. The residue was purified by silica gel column chromatography (eluent: Hexane/EtOAc = 1/1) to afford the compound **13** (745 mg, 96%, 94% ee) as a light yellow film. $[\alpha]_D^{28}$ = -109.0 (c = 0.26, $CHCl_3$); Enantiomeric excess was determined by chiral HPLC (Chiralpak AD-H, 25 °C, flow rate: 1 mL/min, hexane/isopropanol: 70/30, 254 nm, 4.49 min (*R*), 7.17 min (*S*); ^{1}H NMR (500 MHz, $CDCl_3$) δ 7.40 (dd, *J* = 10.3, 1.3 Hz, 1H), 6.79 (s, 1H), 6.64 (s, 1H), 6.31 (dd, *J* = 10.2, 1.6 Hz, 1H), 5.93 (d, *J* = 1.3 Hz, 1H), 5.91 (s, 2H), 4.67 (d, *J* = 16.6 Hz, 1H), 4.54 (dd, *J* = 16.7, 4.7 Hz, 1H), 3.50-3.45 (m, 1H), 3.45-3.38 (m, 1H), 2.70 (d, *J* = 9.3 Hz, 6H), 2.63 (d, *J* = 10.5 Hz, 6H), 2.07-2.00 (m, 1H), 1.99-1.92 (m,

1H), 0.80 (s, 9H), -0.07 (s, 6H); ^{13}C NMR (126 MHz, $CDCl_3$) δ 185.7, 160.8, 160.7, 147.6, 147.1, 146.8, 129.5, 127.6, 125.7, 125.6, 110.9, 110.8, 106.8, 105.7, 101.4, 59.2, 50.3, 50.2, 45.9, 45.9, 40.2, 37.8, 36.4, 25.8, 18.0, -5.5. ^{31}P NMR (162 MHz, $CDCl_3$) δ 19.3; ESI-MS: *m/z* 534.6 $[M+H]^+$; HMRS (ESI) calculated for $[M+H, C_{26}H_{40}N_3O_5PSi]^+$: 534.2553; found: 534.2540.

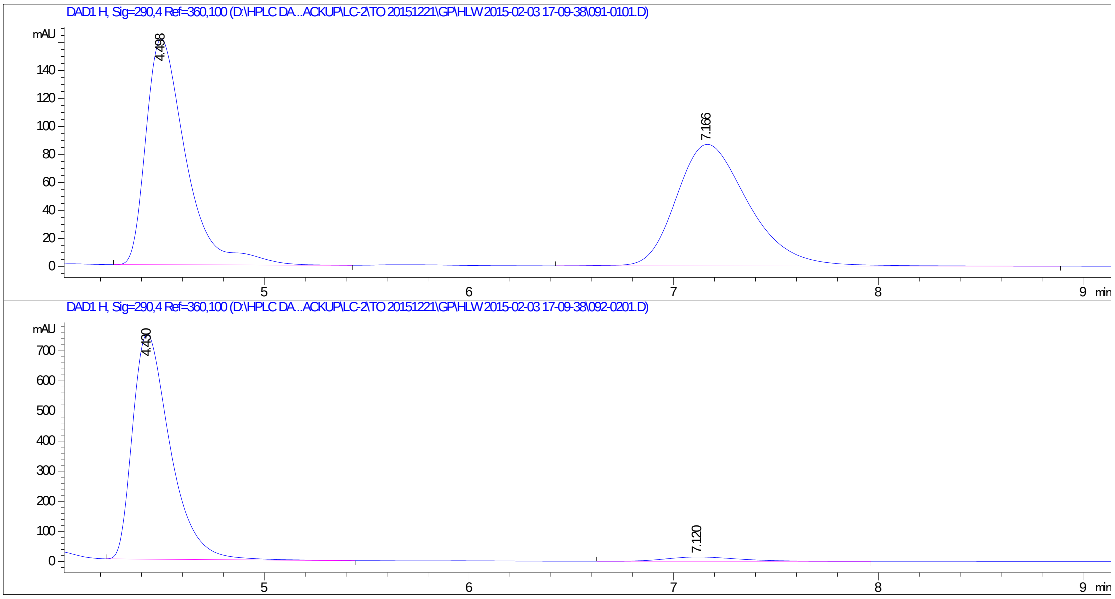

Preparation of diol 14

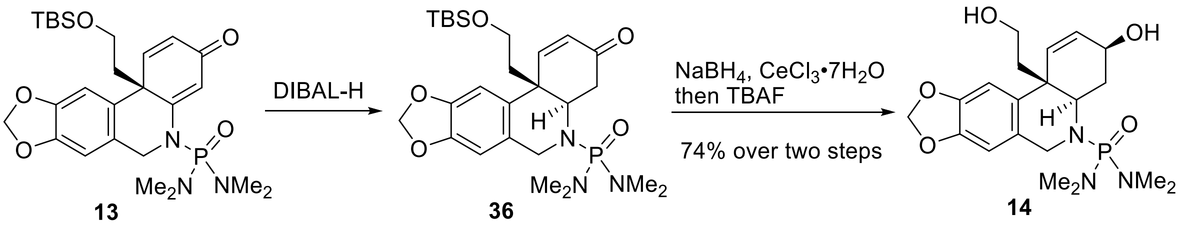

To a stirred solution of compound **13** (10.7 g, 20.0 mmol) in THF (150 mL) at -60 °C was added dropwise DIBAL-H (26.0 mL, 26.0 mmol, 1.3 equiv, 1.0 M in *n*-hexane). After completion of addition, the reaction mixture was stirred at -60 °C for 30 min and then quenched by addition of saturated potassium sodium tartaric acid solution (200 mL). The resulting mixture was allowed to warm to rt and stirred for 1 h. The organic layer was separated and the aqueous layer was extracted with EtOAc (100 mL ×3). The combined

organic layer was washed with brine (150 mL), dried over sodium sulfate, filtered and concentrated under reduced pressure. The residue was purified by silica gel column chromatography (eluent: EtOAc/actone = 5:1) to afford ketone **36** (8.3 g) as a light yellow solid. $[\alpha]_D^{28}$ = -102.6 (c = 0.27, $CHCl_3$); ^{1}H NMR (500 MHz, $CDCl_3$) δ 7.49 (d, *J* = 10.3 Hz, 1H), 7.15 (s, 1H), 6.48 (s, 1H), 5.94 (d, *J* = 1.1 Hz, 1H), 5.91-5.87 (m, 2H), 4.22 (d, *J* = 15.6 Hz, 1H), 4.15 (dd, *J* = 15.8, 6.9 Hz, 1H), 3.81-3.75 (m, 1H), 3.71-3.63 (m, 1H), 3.63-3.54 (m, 2H), 2.65 (d, *J* = 9.2 Hz, 6H), 2.57 (d, *J* = 9.5 Hz, 6H), 2.28-2.20 (m, 2H), 0.88 (s, 9H), 0.03 (s, 3H), 0.00 (s, 3H); ^{13}C NMR (126 MHz, $CDCl_3$) δ 198.3, 158.2, 146.4, 146.3, 131.1, 127.4 (d, *J* = 6.0 Hz), 126.1, 107.7, 105.8, 101.1, 61.1 (d, *J* = 2.8 Hz), 59.6, 51.6 (d, *J* = 4.0 Hz), 43.7, 40.8, 39.2 , 36.8 (d, *J* = 4.2 Hz), 36.6 (d, *J* = 4.9 Hz), 25.9, 18.1, -5.33, -5.52; ESI-MS: *m/z* 536.6 $[M+H]^+$; HMRS (ESI) calculated for $[M+H, C_{26}H_{43}N_3O_5PSi]^+$: 536.2710; found: 536.2691.

To a stirred solution of the above product (6.0 g, 11.2 mmol) in MeOH (110 mL) was added $CeCl_3$•$7H_2O$ (8.3 g, 22.4 mmol) at 0 °C. After 10 min, $NaBH_4$ (560 mg, 14.6 mmol) was added in small portions and the resulting mixture was stirred at 0 °C for 30 min. The reaction was quenched by addition of acetone (50 mL) and the resulting mixture was stirred at rt for additional 15 min, and then poured into H_2O (100 mL) and stirred for 30 min. The mixture was concentrated to remove most of organic solvent and the residue was further treated with DCM (100 mL). The aqueous layer was extracted with DCM (50 mL×4). The combined organic layer was washed with brine (100 mL), dried over sodium sulfate, and concentrated to afford a light yellow oil, which was used for the next operation without further purification.

To the aforementioned yellow oil in THF (50 mL) at 0 °C was added TBAF (22.4 mL, 22.4 mmol 1.0 M in THF) over 10 min. The resulting mixture was allowed to warm to rt and stirred for 6 h and then concentrated. The residue was purified by silica gel column chromatography (eluent: DCM/MeOH = 10:1) to afford the desired diol **14** (4.5 g, 74% over two steps) as a white solid. **14**: m.p. 63-66 °C; $[\alpha]_D^{28}$ = -116.6 (c = 0.25, $CHCl_3$); ^{1}H NMR (500 MHz, $CDCl_3$) *δ* 6.87 (s, 1H), 6.45 (s, 1H), 6.14 (d, *J* = 10.3 Hz,

1H), 5.90 (dd, J = 6.0, 1.4 Hz, 2H), 5.73 (d, J = 10.2 Hz, 1H), 4.27 (t, J = 7.3 Hz, 1H), 4.22 (d, J = 15.6 Hz, 1H), 4.10 (dd, J = 15.6, 6.8 Hz, 1H), 3.73-3.66 (m, 1H), 3.64-3.57 (m, 1H), 3.27-3.20 (m, 1H), 2.68 (d, J = 9.3 Hz, 6H), 2.63 (d, J = 9.3 Hz, 6H), 2.54-2.47 (m, 1H), 2.33-2.25 (m ,1H), 2.08-2.01 (m, 1H), 1.81-1.73 (m, 1H); ^{13}C NMR (126 MHz, $CDCl_3$) δ 146.2, 145.8, 135.1, 132.5, 130.8, 125.9, 106.3, 105.7, 100.9, 68.3, 60.2, 52.1, 51.0, 42.1, 41.8, 36.9 (d, J = 4.6 Hz), 36.5 (d, J = 5.2 Hz), 33.2; ^{31}P NMR (162 MHz, $CDCl_3$) δ 20.8; ESI-MS: *m/z* 423.4 $[M+H]^+$; HMRS (ESI) calculated for $[M+H, C_{20}H_{30}N_3O_5P]^+$: 423.1910; found: 423.1922.

Preparation of Compound 15

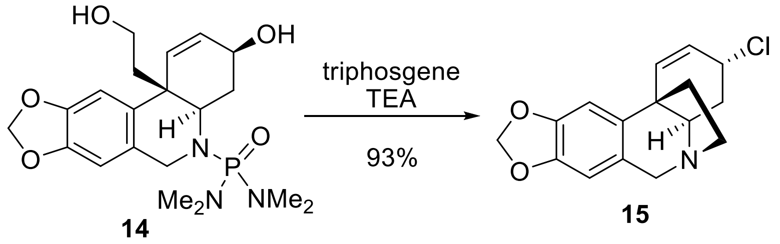

To a stirred solution of diol **14** (3.2 g, 7.6 mmol) in DCM (50 mL) was added TEA (5.5 mL, 38 mmol) and triphosgene (3.4 g, 11.4 mmol) at 0 °C. The reaction mixture was allowed to warm to room temperature and stirred for 2 h, then quenched by addition of H_2O (25 mL). The organic phase was separated and the aqueous layer was extracted with DCM (100 mL ×4). The combined organic layer was washed with brine (100 mL), dried over sodium sulfate, concentrated purified by silica gel column chromatography (eluent: DCM/MeOH = 20:1) to afford compound **15** (1.6 g, 93%) as a white foam. **15**: $[\alpha]_D^{28}$ = 75.6 (c = 0.25, $CHCl_3$); ^{1}H NMR (500 MHz, $CDCl_3$) δ 6.80 (s, 1H), 6.56 (d, J = 9.9 Hz, 1H), 6.49 (s, 1H), 5.93 (dd, J = 9.8, 5.1 Hz), 5.87 (dd, J = 5.8, 1.2 Hz, 2H), 4.71 (t, J = 3.6 Hz, 1H), 4.43 (d, J = 16.8 Hz, 1H), 3.78 (d, J = 16.8 Hz, 1H), 3.56 (dd, J = 12.8, 4.0 Hz, 1H), 3.31 (ddd, J = 13.5, 9.1, 4.5 Hz, 1H), 2.94-2.86 (m, 1H), 2.22-2.15 (m, 2H), 2.01 (ddd, J = 12.8, 8.4, 4.4 Hz, 1H), 1.94-1.85 (m, 1H); ^{13}C NMR (126 MHz, $CDCl_3$)

δ 146.2, 145.8, 137.8, 132.3, 126.5, 126.1, 107.0, 102.7, 100.8, 62.5, 62.3, 53.8, 53.4, 44.6, 44.1, 33.0; ESI-MS: *m/z* 290.1 $[M+H]^+$; HMRS (ESI) calculated for $[M+H, C_{16}H_{16}NO_2Cl]^+$: 290.1825; found: 290.1823.

Preparation of (-)-Crinine

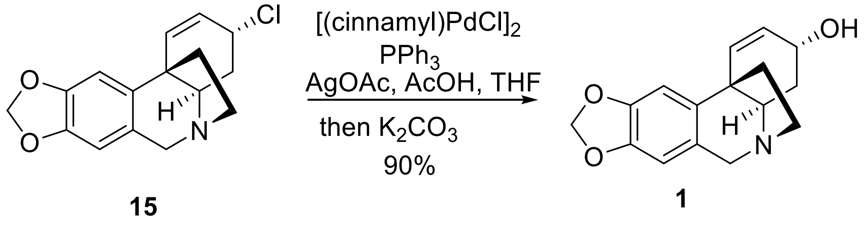

To a flame-dried Schlenk flask equipped with a magnetic stirring bar was charged **15** (1.3 g, 4.5 mmol), $[Pd(cinnamyl)Cl]_2$ (118.8 mg, 0.23 mmol, 5 mol %), PPh_3 (118.0 mg, 0.45 mmol, 10 mol %), and AgOAc (901.3 mg, 5.4 mmol, 1.2 equiv). The mixture was pumped and back-filled with nitrogen for three times. THF/AcOH (10/1, v/v, 20 mL) was added via syringe and the resulting mixture was stirred at rt for 1 h. The reaction mixture was filtered over a pad of Celite and the filtrate was concentrated. The residue was dissolved with DCM (100 mL), washed with saturated $NaHCO_3$ solution, dried over sodium sulfate, filtered and concentrated under reduced pressure to afford a yellow oil, which was used directly for next operation without further purification.

To a stirred solution of the aforementioned oil in MeOH (20 mL) was added K_2CO_3 (3.4 g, 24.8 mmol, 5.5 equiv). The reaction mixture was stirred at rt overnight and then filtered through a pad of Celite. The Celite pad was washed with MeOH (20 mL x 3) and the filtrated was concentrated under reduced pressure. The residue was diluted with $CHCl_3$ (50 mL), washed with saturated $NaHCO_3$ solution (50 mL), dried over sodium sulfate, filtered and concentrated under reduced pressure. The residue was purified by silica gel column chromatography (eluent: DCM/MeOH = 4:1) to afford **1** (1.1 g, 90 %) as a light yellow solid. $[\alpha]_D^{28}$ = -25.8 (c = 0.25, $CHCl_3$) (literature: $[\alpha]_D^{23}$ = -25.8 (c = 0.24, $CHCl_3$), natural;[2] $[\alpha]_D^{23}$ = -26.1 (c =

0.26, $CHCl_3$), synthetic[2]); ^1H NMR (400 MHz, $CDCl_3$) δ 6.84 (s, 1H), 6.58 (d, J = 10.0 Hz, 1H), 6.47(s, 1H), 5.95 (dd, J = 10.0, 5.2 Hz, 1H), 5.88 (dd, J = 6.0, 1.2 Hz, 2H), 4.39 (d, J = 16.8 Hz, 1H), 4.36-4.31(m, 1H), 3.76 (d, J = 16.8 Hz, 1H), 3.41-3.30 (m , 2H), 2.88 (ddd, J = 14.7, 8.8, 5.9 Hz, 1H), 2.17 (ddd, J = 14.7, 8.5, 4.2 Hz, 1H), 2.01-1.88 (m, 2H), 1.73 (td, J = 13.6, 4.0 Hz, 1H); ^{13}C NMR (126 MHz, $CDCl_3$) δ 146.1, 145.7, 138.3, 132.3, 127.4, 126.4, 107.0, 102.8, 100.7, 64.1, 62.8, 62.4, 53.6, 44.2, 32.7; ESI-MS: *m/z* 272.1 $[M+H]^+$; HMRS (ESI) calculated for $[M+H, C_{16}H_{18}NO_3]^+$: 272.1287; found: 272.1284. The ^1H and ^{13}C NMR were in full agreement with reported data.[3]

5. Synthesis of buphanisine (16), epibuphanisine (17) and amabiline (18)

5.1 Synthesis of amabiline (18)

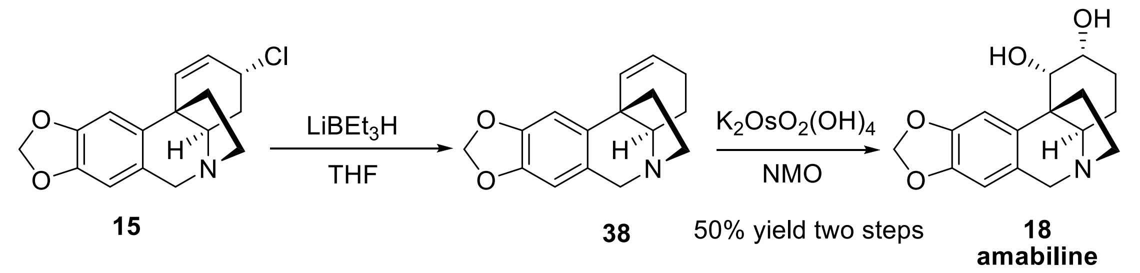

To a solution of compound **15** (66 mg, 0.228 mmol, 1 equiv) in THF (2 mL) at 0 ^{o}C was added dropwise a solution of $LiBEt_3H$ in THF (0.456 mmol, 1.0 M, 2 equiv) over 2 minutes. The desired mixture was stirred at 40 ^{o}C for 8 h. After cooled to rt, the reaction was quenched by addition of 1 *N* NaOH (2 mL). Dichloromethane (5 mL) was added and the organic layer was separated and the aqueous layer was extracted with dichloromethane (5 mL×2). The combined organic layer was washed with brine, concentrated, and purified by silica gel flash column chromatography (Eluents: EtOAc/MeOH/NEt_3 = 100:5:0.5) to give compound **38** (56 mg) as a white foam. **38**: $[\alpha]_D^{28}$ = -61.1 (c = 0.56, EtOH); ^{1}H NMR (500 MHz, $CDCl_3$) δ 6.82 (s, 1H), 6.47 (s, 1H), 6.36 (d, *J* = 9.8 Hz, 1H), 5.86 (d, *J* = 8.4 Hz, 2H), 5.77-5.75 (m, 1H), 4.38 (d, *J* = 16.8 Hz, 1H), 3.76 (d, *J* = 16.8 Hz, 1H), 3.38-3.32 (m, 1H), 3.08 (dd, *J* = 13.0, 3.2 Hz, 1H), 2.87-2.82 (m, 1H), 2.21-2.10 (m, 3H), 2.02-1.96 (m, 1H), 1.77-1.75 (m, 1H), 1.62-1.54 (m, 1H) ; ^{13}C NMR (126 MHz, $CDCl_3$) δ 146.1, 145.6, 139.6, 128.4, 126.9, 126.4, 106.9, 103.1, 100.8, 67.3, 62.6, 52.8, 45.4, 44.3, 25.0, 24.6; ESI-MS: 256.2 $[M+H]^+$; HRMS (ESI) calculated for [M+H, $C_{16}H_{20}O_2N]^+$: 256.1332; found:256.1330.

To a solution of compound **38** (23 mg, 0.090 mmol, 1 equiv) and citric acid (34 mg, 0.180 mmol, 2 equiv) in *t*BuOH (1.2 mL) at rt was added NMO (21 mg, 0.180 mmol, 2 equiv) in water (0.3 mL) and $K_2OsO_2(OH)_4$ (1.6 mg, 0.0045 mmol, 0.05 equiv). The resulting mixture was stirred at rt overnight. The

reaction was quenched by addition of saturated Na_2SO_3 solution (5 mL) and dichloromethane (5 mL). The organic layer was separated and the aqueous layer was extracted with dichloromethane (5 mL×2). The combined organic layer was washed with brine, concentrated, and purified by silica gel flash column chromatography (Eluents: EtOAc/MeOH/NEt_3 = 100:5:0.5) to give amabiline **18** (13 mg, 50% yield for two steps) as a colorless oil. **18**: $[\alpha]_D^{28}$ = -31.8 (c = 0.053, EtOH), literature $[\alpha]_D^{28}$ = -31.6 (c = 0.28, EtOH)[3]; ^{1}H NMR (400 MHz, $CDCl_3$) δ 6.80 (s, 1H), 6.46 (s, 1H), 5.92-5.91 (m, 2H), 4.57 (d, *J* = 6.6 Hz, 1H), 4.38 (d, *J* = 16.5 Hz, 1H), 4.11-4.07 (m, 1H), 3.72 (d, *J* = 16.5 Hz, 1H), 3.42-3.31 (m, 2H), 2.85-2.78 (m, 1H), 2.10 (ddd, *J* = 14.0, 8.1, 6.4 Hz, 1H), 1.93-1.83 (m, 2H), 1.81-1.77 (m, 2H), 1.37 (ddd, *J* = 26.1, 12.6, 5.3 Hz, 1H); ^{13}C NMR (100 MHz, $CDCl_3$) δ 146.8, 145.7, 137.2, 127.2, 107.1, 103.8, 101.0, 69.7, 69.1, 63.3, 62.1, 51.0, 49.8, 37.9, 27.0, 25.8; ESI-MS: 290.0 $[M+H]^+$; HRMS (ESI) calculated for $[M+H, C_{16}H_{20}O_4N]^+$: 290.1387; found: 290.1387. The ^{1}H and ^{13}C NMR were in full agreement with reported data.[3]

5.2 Synthesis of Buphanisine (16) and Epibuphanisine (17)

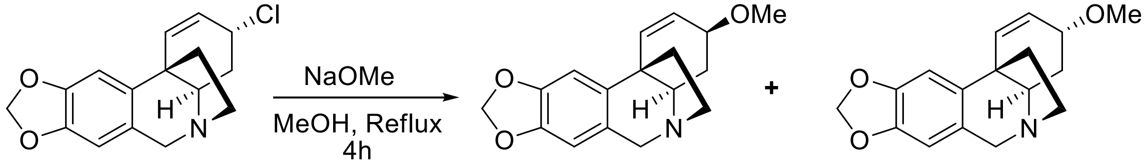

15

17
Epibuphanisine
40%

16
Buphanisine
15%

To a solution of compound **15** (47 mg, 0.163 mmol, 1 equiv) in MeOH at rt was added MeONa (10.5 mg, 0.195 mmol, 1.2 equiv). The desired mixture was stirred at reflux for 4 h. After cooled to rt, the mixture was quenched by addition of saturated NH_4Cl aqueous solution (2 mL) and dichloromethane (5 mL). The organic layer was separated and the aqueous layer was extracted with dichloromethane (5 mL×2). The

combined organic layer was washed with brine. The residue was purified by silica gel flash column chromatography (Eluents: EtOAc/MeOH/NEt_3 = 100:5:0.5) to give compound **17** (epibuphanisine, 18.6 mg, 40%) and compound **16** (buphanisine, 7.0 mg, 15%), respectively, as oils. **Epibuphanisine** (**17**): $[\alpha]_D^{28}$ = -112.9 (c = 0.47, EtOH) (literature: $[\alpha]_D^{22}$ = 129 (c = 0.224, EtOH) for (+)-epibuphanisine) [4]; ^{1}H NMR (400 MHz, $CDCl_3$) δ 6.82 (s), 6.51 (s), 6.40 (dd, *J* = 10.3, 2.2 Hz, 1H), 5.92 (d, *J* = 1.3 Hz, 1H), 5.91 (d, *J* = 1.4 Hz, 1H), 5.85 (d, *J* = 10.3 Hz, 1H), 4.52 (d, *J* = 20.8 Hz, 1H), 4.02-3.97 (m, 1H), 3.88 (d, *J* = 20.8 Hz, 1H), 3.63-3.56 (m, *J* = 1H), 3.42 (s, 3H), 3.34 (dd, *J* = 13.4, 3.5 Hz, 1H), 3.00 (ddd, *J* = 13.2, 7.8, 5.9 Hz, 1H), 2.44 (br s, 1H), 2.27-2.12 (m, 2H), 1.60 (dd, *J* = 24.9, 11.7 Hz, 1H); ^{13}C NMR (100 MHz, $CDCl_3$) δ 146.8, 146.4, 137.9, 129.5, 128.4, 107.2, 103.1, 101.2, 76.0, 67.0, 61.7, 56.3, 53.2, 45.0, 44.3, 30.4; ESI-MS:286.1 $[M+H]^+$; HRMS (ESI) calculated for $[M+H, C_{17}H_{20}O_3N]^+$: 286.1438; found:286.1439. The ^{1}H and ^{13}C NMR were in full agreement with reported data.[4]

Buphanisine (**16**): $[\alpha]_D^{28}$ = -24.8 (c = 0.25, $CHCl_3$) (literature: $[\alpha]_D^{25}$ = +23.0 (c = 0.8, $CHCl_3$)[5]; $[\alpha]_D$ = +18.0 (c = 2.1, $CHCl_3$) for (+)-buphanisine) [6]; ^{1}H NMR (400 MHz, $CDCl_3$) δ 6.84 (s, 1H), 6.61 (d, *J* = 10.0 Hz, 1H), 6.48 (s, 1H), 5.97 (dd, *J* = 10.0, 5.2 Hz, 1H), 5.88 (d, *J* = 5.0 Hz, 1H), 4.42 (d, *J* = 16.8 Hz, 1H), 3.83-3.77 (m, 2H), 3.42-3.33 (m, 2H), 3.36 (s, 3H), 2.94-2.87 (m, 1H), 2.17 (ddd, *J* = 12.6, 10.4, 4.2 Hz, 1H), 2.11 (d, *J* = 13.8 Hz, 1H), 1.96-1.89 (m, 1H), 1.61 (td, *J* = 13.8, 3.8 Hz, 1H); ^{13}C NMR (100 MHz, $CDCl_3$) δ 146.0, 145.6, 138.5, 133.0, 126.4, 125.3, 106.9, 102.9, 100.7, 72.7, 63.1, 62.4, 56.5, 53.6, 44.3, 44.3, 28.9; ESI-MS:286.1 $[M+H]^+$; HRMS (ESI) calculated for $[M+H, C_{17}H_{20}O_3N]^+$: 286.1438; found:286.1440. The ^{1}H and ^{13}C NMR were in full agreement with reported data.[5]

6. Model reactions using diarylamine substrates

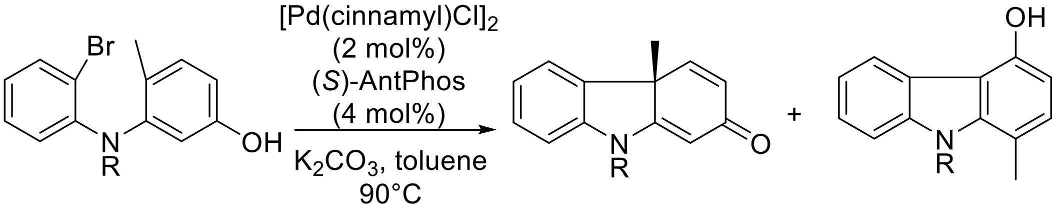

19a: R = P(O)Ph$_2$

19b: R = P(O)(NMe$_2$)$_2$

20a: R = P(O)Ph$_2$ 0% yield

20b: R = P(O)(NMe$_2$)$_2$ 71% yield, 89% ee

21a: R = P(O)Ph$_2$ 90% yield

21b: R = P(O)(NMe$_2$)$_2$ 5% yield

To demonstrate whether an asymmetric dearomative cyclization could be applied to the enantioselective synthesis of aspidospermidine, model reactions were conducted initially to investigate the influence of N-R protecting groups on enantioselective dearomative cyclization for the synthesis of chiral carbazolone products. Bromo aniline substrates **19a** and **19b** were prepared and subjected to dearomative cyclization conditions employed in crinine synthesis with a Pd-(*S*)-AntPhos catalyst. We were pleased to find that substrate **19b** led to the formation of the desired chiral carbazolone **20b** in 71% yield and 89% ee. The *N*-P(O)(NMe$_2$)$_2$ protecting group in **19b** again proved to be critical for excellent chemoselectivity. In contrast, substrate **19a** bearing an *N*-P(O)Ph$_2$ group did not provide the desired product **20a**.

20b: $[\alpha]_D^{28}$ = -368.2 (c = 0.21, CHCl$_3$); ^{1}H NMR (500 MHz, CDCl$_3$) δ 7.61 (d, *J* = 8.1 Hz, 1H), 7.33 (dd, *J* = 9.6, 1.0 Hz, 1H), 7.28-7.23 (m, 2H), 7.06 (t, *J* = 7.3 Hz, 1H), 6.24 (dd, *J* = 9.6, 1.4 Hz, 1H), 5.95 (d, *J* = 1.4 Hz, 1H), 2.78 (s, 3H), 2.76 (s, 3H), 2.76 (s, 3H), 2.74 (s, 3H), 1.62 (s, 3H); ^{13}C NMR (126 MHz, CDCl$_3$) δ 187.2, 173.2, 145.1, 144.0, 133.2, 128.8, 128.7, 123.2, 120.9, 115.5, 106.7, 50.7, 37.9, 36.7, 36.6; ESI-MS: 332.2 [M+H]$^+$; HRMS (ESI) calculated for [M+Na, $C_{17}H_{22}N_3NaO_2P$]$^+$: 354.1342; found:

354.1345. Enantiomeric excess was determined by chiral HPLC (Chiralpak AD-H, 25 °C, flow rate: 1 mL/min, hexanes/isopropanol: 80/20, 254 nm, 8.95 min (*S*), 12.69 min (*R*)).

7. Total synthesis of aspidospermidine (2)

Preparation of phenol ether 20

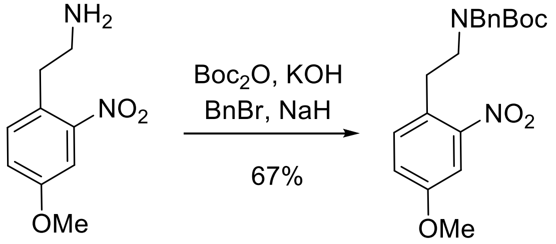

19 **20**

To a solution of compound **19** (5.0 g, 25.5 mol, 1 equiv) in dioxane (40 mL) was added NaOH (1.22 g, 30.5 mmol, 1.2 equiv) in water (20 mL) and dropwise $(Boc)_2O$ (5.78 g, 26 mmol, 1 equiv) over 5 min at 0 °C.The solution was stirred for 2 h and was quenched by addition of H_2O (20 mL) and DCM (40 mL). The organic layer was separated and the aqueous layer was extracted with DCM (30 mL×3), the combined organic layer was washed with brine, dried over sodium sulfate and concentrated under reduced pressure. To the above residue (3.0 g, 10.1 mmol, 1 equiv) in dry DMF (20 mL) was added NaH (811 mg, 20.2 mmol, 2 equiv, 60 wt% in mineral oil) at 0 °C. The desired mixture was stirred at 40 °C for 40 min, and then BnBr (3.4 g, 20.2 mmol, 2 equiv) was added at 0 °C. The mixture was stirred at rt for 4 h and the reaction was quenched by addition of saturated NH_4Cl (20 mL) and heaxane/EtOAc (100 mL, 1/1). The organic layer was separated and the aqueous layer was extracted with hexane/EtOAc (1/1) (20 mL×3), the combined organic layer was washed with brine, dried over sodium sulfate and concentrated under reduced pressure. The residue was purified by silica gel flash column chromatography (Eluents: Hexane/ EtOAc = 10:1) to give the title compound **20** (6.6 g, 67%) as an oil. **20**: ^1H NMR (400 MHz, $CDCl_3$) δ 7.44 (br s,

1H), 7.32-7.22 (m, 5H), 7.10-7.05 (m, 2H), 7.44-7.38 (m, 2H), 3.84 (s, 3H), 3.47-3.40 (m, 2H), 3.07-2.96 (m, 2H), 1.46 (s, 9H); ^{13}C NMR (126 MHz, $CDCl_3$) δ 158.6, 155.9 (155.6), 149.8, 138.6 (138.4), 133.8 (133.7), 128.6, 128.1, 127.6, 127.4, 126.4, 120.1, 109.4 (109.4), 79.9, 55.9, 51.1 (50.1), 47.7 (47.3), 31.6 (31.1), 28.5; ESI-MS: 409.2 $[M+Na]^+$; HRMS (EI) calculated for $[M+Na, C_{21}H_{26}N_2NaO_5]^+$: 409.1743; found: 409.1734.

Preparation of amine 21

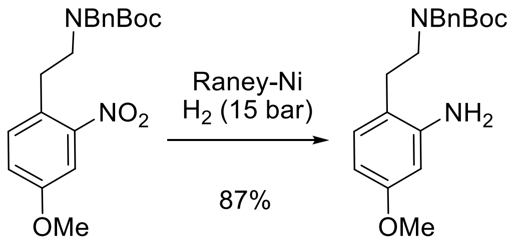

20 **21**

To a solution of compound **20** (3.3 g, 8.5 mmol, 1 equiv) in EtOAc /MeOH (60 mL, 1/1) was added Raney nickle (800 mg) and the desired mixture was stirred for 90 min under 15 atm hydrogen atmosphere at rt . The mixture was filtered through a pad of Celite and the catalyst was washed with EtOAc (20 mL). The filtrate was concentrated under reduced pressure to afford the desired compound **21** (2.6 g, 87%) as a white solid. m.p. 75-77 °C; ^{1}H NMR (400 MHz, $CDCl_3$) δ 7.36-7.33 (m, 2H), 7.30-7.26 (m, 4H), 6.82 (d, J = 8.5 Hz, 1H), 6.26-6.23 (m, 2H), 4.44 (br s, 2H), 4.27 (s, 1H), 3.74 (s, 3H), 3.61 (br s, 1H), 3.29-3.27 (m, 2H), 2.67-2.58 (m, 2H), 1.55-1.50 (m, 9H); ^{13}C NMR (101 MHz, $CDCl_3$) δ 159.5, 155.9, 146.5 (145.6), 138.7 (138.4), 131.1 (131.0), 128.6, 128.0, 127.4, 115.7 (115.4), 104.0 (103.2), 101.3 (101.0), 80.0, 55.1, 51.9 (50.7), 47.4 (46.3), 30.3, 28.5; ESI-MS: 357.5 $[M+H]^+$; HRMS (EI) calculated for $[M+H, C_{21}H_{29}N_2N_2O_3]^+$: 357.2173; found: 357.2177.

Preparation of diaryl amine 22

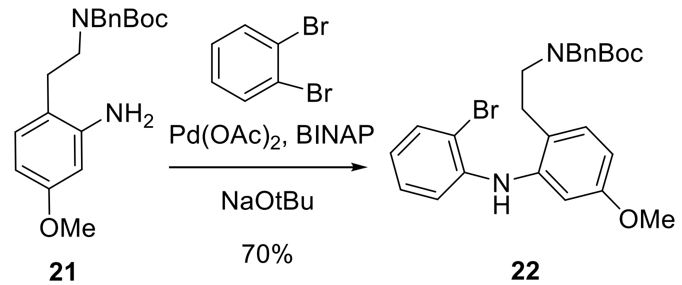

To a flame dried Schlenk tube was added compound **21** (520 mg, 1.46 mmol, 1 equiv), $Pd(OAc)_2$ (32 mg, 0.146 mmol, 0.1 equiv), BINAP (21 mg, 0.146 mmol, 0.1 equiv), *t*BuONa (210 mg, 2.19 mmol, 1.5 equiv). The mixture was pumped and backfilled with nitrogen for three times. Dibromobenzene (414 mg, 1.75 mmol, 1.2 equiv) was added via syringe followed by addition of toluene (5 mL). The mixture was stirred at 100 °C for 12 h. After cooled to rt, EtOAc (5 mL) was added and the mixture was filtered through a pad of Celite and concentrated under reduced pressure. The residue was purified by silica gel flash column chromatography (Eluents: Hexane/ EtOAc = 10:1) to give the title compound **22** (520 mg, 70%) as an oil. ^{1}H NMR (400 MHz, $CDCl_3$) δ 7.53 (dd, *J* = 8.0, 1.4 Hz, 2H), 7.28-7.21 (m, 3H), 7.20-7.06 (m, 4H), 6.92-6.84 (m, 1H), 6.76-6.71 (m, 2H), 6.64 (br s, 1H), 6.11-5.84 (m, 1H), 4.39-4.31 (m, 2H), 3.75 (s, 3H), 3.37-3.30 (m, 2H), 2.79-2.73 (m, 2H), 1.45 (s, 9H); ^{13}C NMR (101 MHz, $CDCl_3$) δ 159.2, 155.9 (155.6), 142.5, 140.9 (140.7), 138.6 (138.4), 132.9, 131.7, 128.5, 128.3, 128.0 (127.4), 127.2, 125.1 (124.5), 120.7, 116.5 (115.9), 112.3 (118.3), 110.2 (109.6), 108.9 (108.4), 79.8, 55.4, 51.2 (50.2), 47.5 (47.1), 30.1 (29.7), 28.5; ESI-MS: 511.4 $[M+H]^+$; HRMS (ESI) calculated for $[M+H, C_{27}H_{32}BrN_2O_3]^+$: 511.1591; found: 511.1592.

Preparation of phenol 23

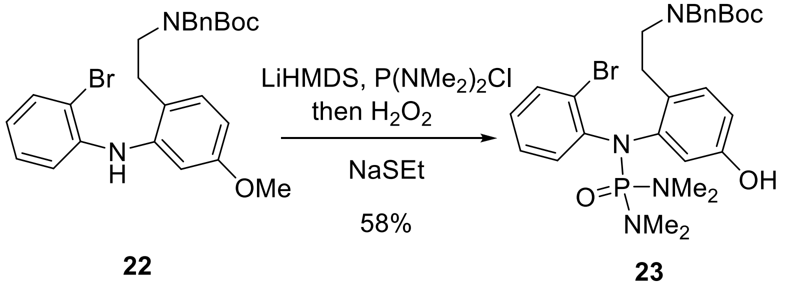

To a solution of compound **22** (550 mg, 1.08 mmol, 1.0 equiv) in THF (15 mL) was added dropwise LiHMDS (1.0 M, 1.40 mmol, 1.3 equiv) at -15℃ and the mixture was stirred at -15 ℃ for 1 h. To the solution was added dropwise $P(NMe_2)_2Cl$ (211 mg, 1.40 mmol, 1.3 equiv) at -15℃ and the mixture was stirred at rt overnight. To the mixture at 0℃ was added 30% aqueous H_2O_2 (158 mg, 1.4 mmol, 1.3 equiv) and the mixture was stirred at rt for 0.5 h .The reaction was quenched by addition of saturated aqueous Na_2SO_3 and EtOAc (20 mL). The organic layer was separated and the aqueous layer was washed with EtOAc (20 mL×3). The combined organic layer was washed with brine, dried with sodium sulfate and concentrated under reduced pressure. The crude product was used in the next step without further purification.

To a solution of NaH (87 mg, 2.18 mmol, 2 equiv, 60 wt% in mineral oil) in DMF (2 mL) was added dropwise HSEt (161 μL, 2.18 mmol, 2 equiv) at 0 ℃ and the mixture was stirred at rt for 10 min. To the solution was added a solution of the above crude compound in DMF (700 mg, 1.09 mmol, 1.0 equiv) at rt and the mixture was stirred at 180 ℃ for 1 h. After being cooled to rt, the reaction was quenched by addition of MeOH and the solution was concentrated under reduced pressure. Saturated NH_4Cl (10 mL) and DCM (20mL) was added. The organic layer was separated and the aqueous layer was extracted with DCM (20 mL×3). The combined organic layer was washed with brine, dried with sodium sulfate and concentrated under reduced pressure. The residue was purified by silica gel flash chromatography (eluents:

DCM/MeOH = 20/1) to afford compound **23** (400 mg, 58%) as a white solid. m.p. 162-165 °C; ^{1}H NMR (500 MHz, $CDCl_3$) δ 9.80 (s, 1H), 8.08 (s, 1H), 7.65 (d, *J* = 8.0 Hz, 1H), 7.36 (br s, 1H), 7.24-7.16 (m, 3H), 7.11 (t, *J* = 7.2 Hz, 1H), 6.99-6.88 (m, 3H), 6.81 (br s, 1H), 6.66 (dd, *J* = 8.3, 2.4 Hz, 1H), 4.12-4.07 (m, 2H), 2.84 (br s, 2H), 2.75 (br s, 1H), 2.64 (br s, 1H), 2.51 (s, 6H), 2.50 (s, 6H), 1.47-1.40 (m, 9H); ^{13}C NMR (101 MHz, $CDCl_3$) δ 156.2, 155.9, 142.6, 138.5, 134.5, 132.7, 131.6, 128.3, 127.9, 127.1, 126.8, 126.6, 123.2, 117.6, 114.1, 79.7, 50.9, 47.4, 37.4, 29.4, 28.6; ESI-MS: 631.6 $[M+H]^+$; HRMS (ESI) calculated for $[M+H, C_{30}H_{41}BrN_4O_4P]^+$: 631.2043; found: 631.2024.

Synthesis of tricyclic skeleton (24)

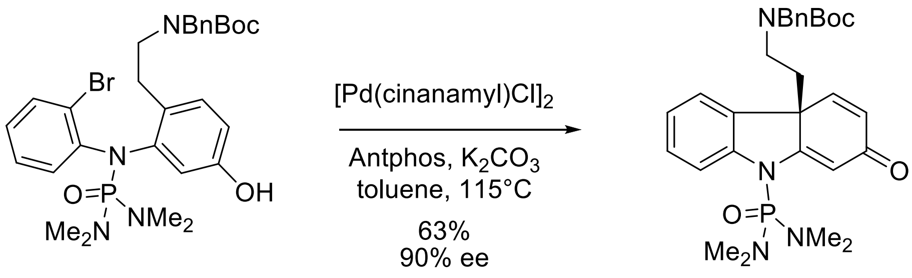

23 **24**

To a flame-dried Schlenk tube was added compound **23** (500 mg, 0.79 mmol, 1 equiv), $[Pd(cinanmyl)Cl]_2$ (8.22 mg, 0.016 mmol, 0.02 equiv), AntPhos (11.7 mg, 0.032 mmol, 0.04 equiv) and K_2CO_3 (164 mg, 1.19 mmol, 1.5 equiv). The mixture was evacuated and back-filled with nitrogen for three times. Toluene (8 mL) was added via syringe and the mixture was stirred at 115 °C overnight. After cooled to rt, EtOAc (20 mL) was added and the mixture was through a pad of Celite and concentrated under reduced pressure. The residue was purified by silica gel flash column chromatography (Eluents: EtOAc/MeOH = 20:1) to give the title compound **24** (274 mg, 63%) as a white solid. m.p. 98-99 °C; 90% ee; Enantiomeric excess was determined by chiral HPLC (Chiralpak AD-H, 25 °C, flow rate: 1 mL/min, hexane/isopropanol: 80/20, 254 nm, 8.32 min (*S*), 12.99 min (*R*)); The absolute configuration was determined according to the X-ray

crystal structure of **29;** $[\alpha]_D^{23}$ = -255.4 (c = 0.53, $CHCl_3$); ^{1}H NMR (500 MHz, $CDCl_3$) δ 7.58 (s, 1H), 7.33-7.19 (m, 6H), 7.07-7.04 (m, 3H), 6.26 (d, *J* = 9.2 Hz, 1H), 5.93 (s, 1H), 4.30-4.21 (m, 2H), 3.37-3.07 (m, 2H), 2.70-2.68 (m, 12H), 1.95-1.87 (m, 2H), 1.38 (m, 9H); ^{13}C NMR (126 MHz, $CDCl_3$) δ 186.8, 171.1, 155.4, 144.3, 143.4, 137.9, 129.8, 128.9, 128.7, 128.0, 127.5, 127.3, 123.0, 121.9 (d, *J* = 25.2 Hz), 115.4 (d, *J* = 7.6 Hz), 107.2 (d, *J* = 73.1 Hz), 80.3, 50.4 (d, *J* = 47.9 Hz), 46.9 (d, *J* = 228 Hz), 42.4 (d, *J* = 52.9 Hz), 36.69, 36.66, 36.58, 36.54, 28.5; ESI-MS: 573.6 $[M+Na]^+$; HRMS (ESI) calculated for $[M+Na, C_{30}H_{39}N_4NaO_4P]^+$: 573.2608; found: 573.2608.

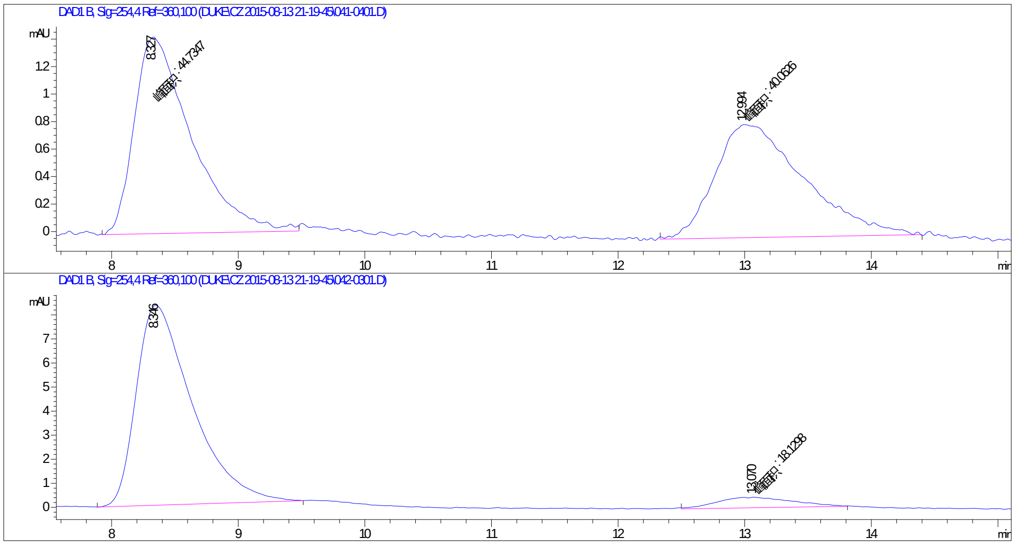

Preparation of compound 25

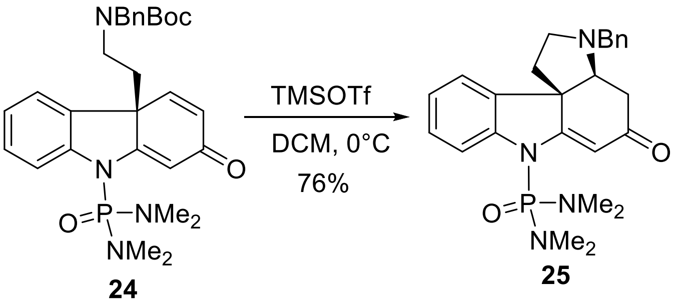

To a solution of compound **24** (280 mg, 0.509 mmol, 1 equiv) in DCM (12 mL) at 0℃ was added TMSOTf (564 mg, 2.54 mmol, 5 equiv) and the resulting mixture was stirred at 0 ℃ for 5 min. The reaction was quenched by adding saturated $NaHCO_3$ (20 mL) until PH > 7. The organic layer was separated and the aqueous layer was extracted with DCM (3×10 mL). The combined organic layer was washed with saturated NH_4Cl (aq), dried over sodium sulfate, concentrated and the residue was purified by silica gel flash column chromatography (Eluent: DCM/MeOH = 20:1) to give the title compound **25** (174 mg, 76%). **25**: $[\alpha]_D^{27}$ = -252.1 (c = 0.47, $CHCl_3$); ^{1}H NMR (500 MHz, $CDCl_3$) δ 7.88 (d, *J* = 7.6 Hz, 1H), 7.52 (d, *J* = 7.7 Hz, 1H), 7.44 (d, *J* = 7.7 Hz, 2H), 7.33 (t, *J* = 7.6 Hz, 2H), 7.26-7.22 (m, 2H), 7.09 (t, *J* = 7.5 Hz, 1H), 5.79 (s, 1H), 3.87 (s, 2H), 3.67 (dd, *J* = 9.3, 7.8 Hz, 1H), 3.32-3.28 (m, 1H), 2.87-2.82 (m, 1H), 2.72-2.70 (m, 9H), 2.60 (dd, *J* = 17.5, 6.4 Hz, 1H), 2.55 (d, *J* = 17.1, 9.4 Hz, 1H), 2.41-2.36 (m, 1H), 2.11-2.08 (m, 1H); ^{13}C NMR (126 MHz, $CDCl_3$) δ 197.3, 171.0 (d, *J* = 2.5 Hz), 142.9 (d, *J* = 6.3 Hz), 138.6, 137.0 (d, *J* = 6.3 Hz), 128.6, 128.4, 128.2, 127.2, 123.5 (d, *J* = 8.8 Hz), 114.0, 106.0, 64.0, 54.8, 54.0 (d, *J* = 5.0 Hz), 49.0, 41.7, 36.6, 36.5, 36.43, 36.4, 34.0; ESI-MS: 451.6 $[M+H]^+$; HRMS (ESI) calculated for $[M+H, C_{25}H_{32}N_4O_2P]^+$: 451.2257.

Preparation of compound 26

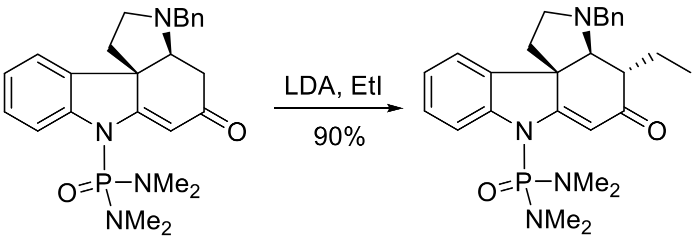

25 26

To a solution of compound **25** (150 mg, 0.33 mmol, 1 equiv) in THF (5 mL) was added dropwise LDA (0.66 mL, 1.0 M in THF, 0.66 mmol, 2 equiv) at -40 ℃ over 3 min and the mixture was stirred at -78 ℃

for 60 min, followed by dropwise addition of EtI (103 mg, 0.66 mmol, 2 equiv). The reaction solution was stirred at -78 °C for 60 min and then at rt overnight. The reaction was quenched by addition of saturated NH_4Cl (aq) (10 mL) and DCM (10 mL). The organic layer was separated and the aqueous layer was extracted with DCM (3×10 mL). The combined organic layer was washed with saturated NH_4Cl (aq), dried over sodium sulfate, concentrated and the residue was purified by silica gel flash column chromatography (Eluent: EA/MeOH = 30: 1) to give the title compound **26** (143 mg, 90%). **26** : $[\alpha]_D^{26}$ = -169.8 (c = 0.37, $CHCl_3$); ^{1}H NMR (500 MHz, $CDCl_3$) δ 7.64 (d, *J* = 8.1 Hz, 1H), 7.40 (d, *J* = 7.1 Hz, 2 H), 7.34 (t, *J* = 7.5 Hz, 3H), 7.28-7.23 (m, 2H), 7.06 (t, *J* = 7.3 Hz, 1H), 5.77 (s, 1H), 4.07 (d, *J* = 13.2 Hz, 1H), 3.84 (d, *J* = 13.2 Hz, 1H), 3.28 (d, *J* = 2.8 Hz, 1 H), 2.81-2.69 (m, 1H), 2.75 (s, 3H), 2.74 (s, 3H), 2.72 (s, 3H), 2.71 (s, 3H), 2.56 (td, *J* = 6.4, 2.8 Hz, 1H), 2.11-1.99 (m, 2H), 1.63-1.55 (m, 2H), 1.42-1.32 (m , 1H), 0.79 (t, *J* = 7.4 Hz, 3H); ^{13}C NMR (101 MHz, $CDCl_3$) δ 201.3, 170.2, 143.1 (d, *J* = 6.1 Hz), 138.8, 137.2 (d, *J* = 7.0 Hz), 128.9, 128.5, 128.3, 127.3, 123.2, 122.2, 114.6, 103.7, 55.6, 57.3, 54.8 (d, *J* = 6.1 Hz), 50.7, 49.2, 47.9, 36.6, 36.5, 27.8, 11.1; ESI-MS: 479.4 $[M+H]^+$; HRMS (ESI) calculated for $[M+H, C_{27}H_{36}N_4O_2P]^+$: 479.2670; found: 479.2569.

Preparation of compound 27

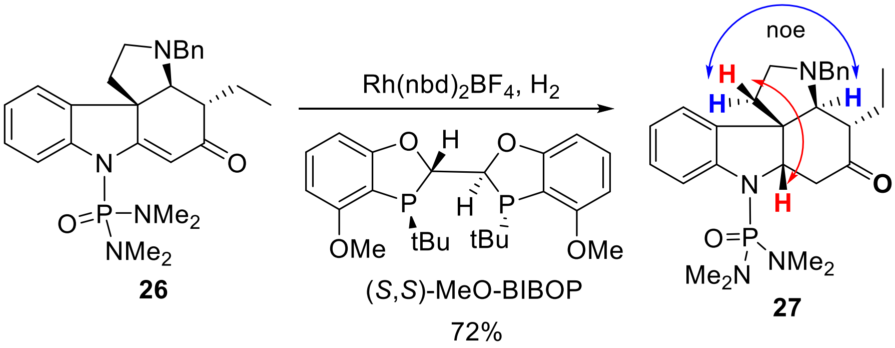

To a 5 mL dry tube in glove box was added $Rh(nbd)_2BF_4$ (3.13 mg, 8.4 μmol, 0.05 equiv) and (*S*,*S*)-MeO-BIBOP (5.04 mg, 8.4 μmol, 0.05 equiv) followed by addition of THF (1 mL) .The mixture was stirred at rt for 15 min. the solution of compound **26** (80 mg, 0.167 mmol, 1 equiv) in THF (2 mL) was added. The solution was stirred at 35 °C under 600 psi H_2 atmosphere for 24 h. The reaction was stopped by releasing of H_2.The solution was concentrated and the residue was purified by silica gel flash column chromatography (Eluents: EA /MeOH = 30:1) to give the title compound **27** (58 mg, 72%). **27**: $[\alpha]_D^{26}$ = 26.8 (c = 0.7, $CHCl_3$); ^1H NMR (500 MHz, $CDCl_3$) δ 7.32 -7.31 (m , 5H), 7.15 (t, *J* = 7.8 Hz, 1H), 7.12 (d, *J* = 7.5 Hz, 1H), 6.94 (d, *J* = 7.7 Hz, 1H), 6.91 (t, *J* = 8.1 Hz, 1H), 4.47-4.72 (m, 1H), 4.21 (d, *J* = 13.1 Hz, 1H), 3.43 (d, *J* = 13.1 Hz, 1H), 3.30 (d, *J* = 4.8 Hz, 1H), 3.06-3.00 (m, 1H), 2.87 (dd, *J* = 11.2, 6.2 Hz, 1H), 2.74 (s, 3H), 2.72 (s, 3H), 2.65 (s, 3H), 2.62 (s, 3H), 2.57-2.45 (m, 3H), 2.19-2.12 (m, 1H), 1.84-1.77 (m, 1H), 1.61-1.53 (m, 2H), 0.87 (t, *J* = 7.4 Hz, 3H); ^{13}C NMR (101 MHz, $CDCl_3$) δ 210.9, 143.9 (d, *J* = 4.0 Hz), 139.0, 136.5 (d, *J* = 8.0 Hz), 128.5, 128.4, 128.3, 127.0, 123.5, 121.2, 113.4, 69.1, 67.2 (d, *J* = 5.0 Hz), 58.4, 54.4, 53.8 (d, *J* = 5.0 Hz), 50.6, 42.1, 38.5, 36.7, 36.7, 36.6, 36.5, 21.8, 12.2; ESI-MS: 481.5 $[M+H]^+$; HRMS (ESI) calculated for $[M+H, C_{27}H_{38}N_4O_2P]^+$: 481.2727; found: 481.2722.

Preparation of compound 28

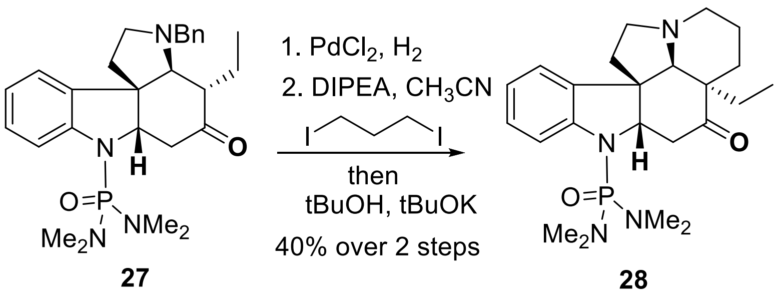

27 **28**

A mixture of **27** (42 mg, 0.087 mmol, 1 equiv), $PdCl_2$ (4.6 mg, 0.026 μmol, 0.3 equiv) and MeOH (4 mL) was stirred at 30 °C under 1 atm hydrogen atmosphere for 12 h. The mixture was filtrated through a pad of

Celite. The filtrate was concentrated and the residue was directly used for next step without further purification.

To a solution of the above compound (24 mg, 0.0613 mmol, 1 equiv) in CH_3CN (0.3 mL) under nitrogen was added DIPEA (79 mg, 0.613 mmol, 10 equiv) and 1,3-diiodopropane (296 mg, 0.613 mmol, 10 equiv). The mixture was stirred at 50℃ overnight. After being cooled to rt, *t*-BuOH (0.2 mL) and *t*-BuOK (13.7 mg, 0.12 mmol, 2 equiv) was added to the reaction system at 0 ℃ and the resulting mixture was stirred at 0 ℃ for 2 h. The reaction was quenched by addition of DCM (4 mL) and H_2O (2 mL). The organic layer was separated and the aqueous layer was extracted with DCM (3×3 mL). The combined organic layer was washed with saturated NH_4Cl (aq), dried over sodium sulfate, concentrated. and the residue was purified by silica gel flash column chromatography (Eluent: DCM/MeOH/NEt_3 = 95:5:1) to give the title compound **28** (10.5 mg, 40 %). **28**: $[\alpha]_D^{26}$ = 32.1 (c = 0.47, $CHCl_3$); ^{1}H NMR (500 MHz, $CDCl_3$) δ 7.21 (d, *J* = 7.4 Hz, 1H), 7.14 (t, *J* = 7.8 Hz, 1H), 7.01 (d, *J* = 8.0 Hz, 1H), 6.92 (t, *J* = 9.4 Hz, 1H), 4.35-4.31 (m, 1H), 3.13 (td, *J* = 9.0, 4.6 Hz, 1H), 3.00 (d, *J* = 10.8 Hz, 1H), 2.78 (dd, *J* = 10.5, 8.4 Hz, 1H), 2.74 (s, 3H), 2.72 (s, 3H), 2.68 (s, 3H), 2.66 (s, 3H), 2.58 (dd, *J* = 14.6, 9.7 Hz, 1H), 2.42-2.39 (m, 1H), 2.35-2.28 (m, 1H), 2.26-2.20 (m, 1H), 2.02 (td, *J* = 11.9, 2.8 Hz, 1H), 1.75-1.60 (m, 4H), 1.50-1.48 (m, 1H), 1.24-1.18 (m, 1H), 0.78 (td, *J* = 13.4, 4.5 Hz, 1H), 0.56 (t, *J* = 7.6 Hz, 1H); ^{13}C NMR (126 MHz, $CDCl_3$) δ 210.6, 144.4 (d, *J* = 3.8 Hz, 1H), 137.0 (d, *J* = 8.8 Hz, 1H), 128.2, 123.5, 121.6, 114.1, 73.2, 69.1 (d, *J* = 3.8 Hz), 53.7 (d, *J* = 5.0 Hz, 1H), 53.6, 52.0, 51.6, 42.9, 38.0, 37.0, 37.0, 37.0, 36.9, 30.8, 29.3, 22.3, 7.8; ESI-MS: 431.4 $[M+H]^+$; HRMS (ESI) calculated for $[M+H, C_{23}H_{36}N_4O_2P]^+$: 431.2570; found: 431.2569.

Preparation of compound 29

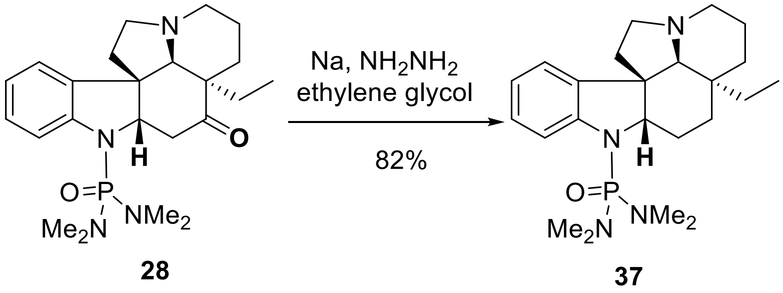

To a solution compound **28** (3 mg, 7.0 μmol, 1 equiv) in ethylene glycol (0.2 mL) under nitrogen was added Na (28 mg, 1.21 mmol, 175 equiv) and $NH_2NH_2•H_2O$ (83 mg, 1.7 mmol, 240 equiv). The mixture was stirred at 160 °C for 1 h and then at 210 °C for 5 h. After being cooled to rt, the reaction was quenched by addition of DCM (4 mL) and H_2O (2 mL). The organic layer was separated and the aqueous layer was extracted with DCM (3×3 mL). The combined organic layer was washed with saturated NH_4Cl (aq), dried over sodium sulfate, concentrated. and the residue was purified by silica gel flash column chromatography (Eluent: DCM/MeOH/NEt_3 = 95:5:1) to give the title compound **37** (2.4 mg, 82 %). **37**：$[\alpha]_D^{26}$ = 24.1 (c = 0.1, $CHCl_3$); ^{1}H NMR (500 MHz, $CDCl_3$) δ 7.12 (d, *J* = 7.4 Hz, 1H), 7.07 (t, *J* = 7.9 Hz, 1H), 6.94 (d, *J* = 7.8 Hz, 1H), 6.84 (t, *J* = 7.4 Hz, 1H), 3.96-3.92 (m, 1H), 3.11 (td, *J* = 7.0, 2.8 Hz, 1H), 3.02 (d, *J* = 10.8 Hz, 1H), 2.74 (s, 3H), 2.73 (s, 3H), 2.69 (s, 3H), 2.67 (s, 3H), 2.26-2.19 (m, 3H), 2.01-1.89 (m, 3H), 1.77-1.69 (m, 1H), 1.64-1.62 (m, 1H), 1.52-1.40 (m, 4H), 1.12-1.03 (m, 2H), 0.92-0.82 (m, 1H), 0.65 (t, *J* = 7.5Hz, 3H); ^{13}C NMR (126 MHz, $CDCl_3$) δ 144.2, 130.1, 127.3, 123.0, 120.9, 113.5, 71.1, 68.7, 53.9, 53.3, 52.8, 39.4, 37.2, 37.1, 37.0, 36.9, 35.7, 34.5, 30.3, 27.1, 23.3, 21.2, 7.0; ESI-MS: 417.4 $[M+H]^+$; HRMS (ESI) calculated for $[M+H, C_{23}H_{38}N_4O_2P]^+$: 417.2778; found: 417.2775 .

Preparation of Aspidospermidine (2)

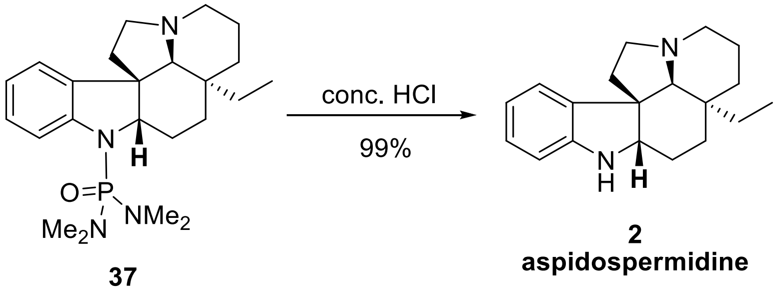

37

A solution of compound **37** (3 mg, 7.0 μmol, 1 equiv) in EtOH (0.1 mL) and 4 M HCl (0.2 mL) was stirred at 110 ℃ under nitrogen for 24 h. After being cooled to rt, the reaction was quenched by addition of saturated $NaHCO_3$ (aq) until PH > 7, followed by addition of DCM (5mL). The organic layer was separated and the aqueous layer was extracted with DCM (3×3 mL). The combined organic layer was washed with saturated NH_4Cl (aq), dried over sodium sulfate, concentrated and the residue was purified by silica gel flash column chromatography (Eluent: DCM/MeOH/NEt_3 = 95:5:1) to give (-)-**aspidospermidine** (**2**, 2.0 mg, 99%). **2**: $[\alpha]_D^{26}$ = -18.5 (c = 0.067, $CHCl_3$); Literature of (+)-aspidospermidine: $[\alpha]_D^{23}$ = +20.8 (c = 1.00, $CHCl_3$) (ref: S. B. Jones, B. Simmons, A. Mastracchio, D. W. C. MacMillan, *Nature* **2011**, *475*, 183). ^{1}H NMR (500 MHz, $CDCl_3$) δ 7.08 (d, *J* = 7.4 Hz, 1H), 7.01 (td, *J* = 7.6, 1.2 Hz, 1H), 6.72 (td, *J* = 7.4, 1.0 Hz, 1H), 6.64 (d, *J* = 7.8 Hz, 1H), 3.51 (dd, *J* = 11.0, 6.2 Hz, 1H), 3.13-3.10 (m, 1H), 3.05 (d, *J* = 10.8 Hz, 1H), 2.32-2.21 (m, 2H), 2.22 (s, 3H), 1.98-1.91 (m, 2H), 1.74 (qt, *J* = 14.5, 4.4 Hz, 1H), 1.66-1.61 (m, 2H), 1.52-1.45 (m, 3H), 1.43-1.34 (m, 1H), 1.11 (td, *J* = 13.6, 4.6 Hz, 1H), 1.05 (dt, *J* = 13.5, 3.2 Hz, 1H), 0.90-0.83 (m, 1H), 0.64 (t, *J* = 7.5 Hz, 3H); ^{13}C NMR (126 MHz, $CDCl_3$) δ 149.5, 135.9, 127.2, 123.0, 119.1, 110.5, 71.4, 65.8, 54.0, 53.5, 53.2, 39.0, 35.8, 34.6, 30.1, 28.3, 23.2, 21.9, 7.0; ESI-MS: 283.3 $[M+H]^+$. HRMS (ESI) calculated for $[M+H, C_{19}H_{27}N_2]^+$: 283.2169; found: 238.2169.

8. Formal Synthesis of Minfiensine (3)

Preparation of compound 29

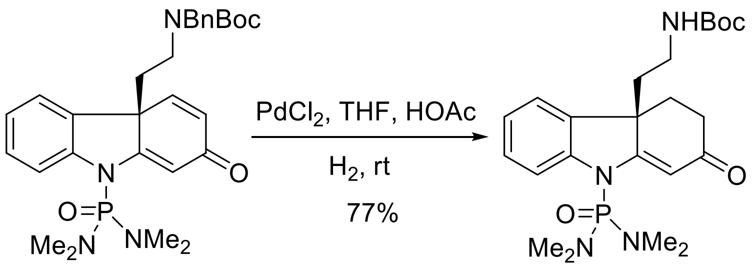

To a 25 mL round-bottom flask containing enone **24** (0.30 g, 0.54 mmol) was added 0.10 g (30 wt%) $PdCl_2$, THF (4 mL) and one drop of HOAc. The mixture was stirred under 1 atm H_2 at room temperature for 72 h. The reaction mixture was filtered through a layer of Celite and the catalyst was washed with MeOH (5 mL×3). The filtrates were combined and concentrated under reduced pressure. The crude product **29** was purified by column chromatography (eluent: ethyl acetate/THF = 1/1) to give a white solid (0.19 g, 77% yield). m.p. 136-138 °C; $[\alpha]_D^{23}$= -320.3 (c = 0.18, $CHCl_3$); ^{1}H NMR (500 MHz, $CDCl_3$) δ 7.57 (d, *J* = 8.1 Hz, 1H), 7.26 (t, *J* = 8.1 Hz, 1H), 7.21 (d, *J* = 7.4 Hz, 1H), 7.08 (t, *J* = 7.4 Hz, 1H), 5.79 (s, 1H), 4.47 (br s, 1H), 3.23-3.11 (m, 1H), 2.99-2.89 (m, 1H), 2.76 (s, 3H), 2.74 (s, 3H), 2.73 (s, 3H), 2.71 (s, 3H), 2.70-2.64 (m, 1H), 2.55 (dd, *J* = 12.9, 5.2 Hz, 1H), 2.52 (dd, *J* = 18.3, 5.2 Hz, 1H), 2.10-2.03 (m, 1H), 1.98 (td, *J* = 13.4, 5.8 Hz, 1H), 1.93-1.83 (m, 1H), 1.39 (s, 9H); ^{13}C NMR (126 MHz, $CDCl_3$) δ 197.9, 173.1, 155.7, 144.1, 134.4, 128.7, 123.2, 122.6, 114.5, 105.6, 79.4, 56.6, 48.1, 39.3, 36.6, 36.5, 36.4, 36.3, 32.6, 29.8, 28.4; ESI-MS: 463.1 $[M+H]^+$; HRMS (ESI) calculated for $[M+H, C_{23}H_{36}N_4O_4P]^+$: 463.2474; found: 463.2466.

Preparation of compound 30

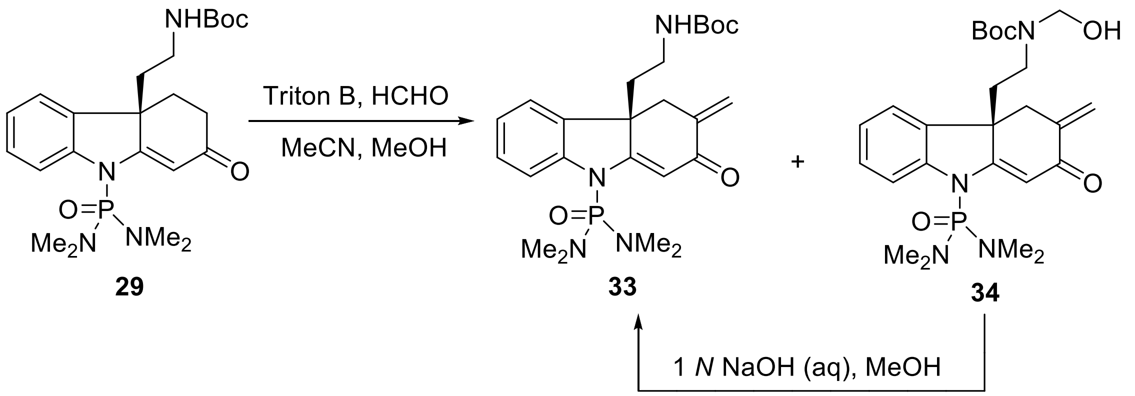

To a suspension of paraformaldehyde (4 mg, 0.13 mmol) in MeCN (0.3 mL) was added a 40% solution of Triton B in MeOH (6 μL, 0.013 mmol) at room temperature. The resulting mixture was stirred at rt for 10 min and became a clear solution. To the obtained solution was added a solution of enone **4** (20 mg, 0.043 mmol) in MeCN (0.5 mL). The reaction mixture was warmed to 65 °C and stirred for 3 h. Upon fully consumption of starting material, the mixture was partitioned between EtOAc (15 mL) and brine (5 mL). The organic phase was separated and the aqueous layer was extracted with EtOAc (10 mL × 3). The organic phases were combined, dried over Na_2SO_4, filtered and concentrated under reduced pressure to give a mixture of **33** and **34**.

The crude mixture was treated with 1 *N* NaOH (aq) (0.2 mL) in MeOH (3 mL) for 2 h at rt to give product **33** which was used in the next step without further purification.

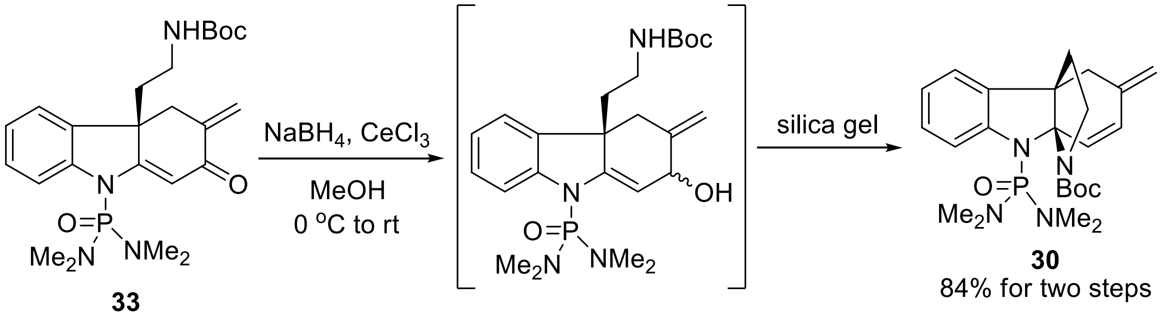

To a solution of the above dienone **33** (12 mg, 0.025 mmol) in MeOH (0.8 mL) was added $CeCl_3$•$7H_2O$ (28 mg, 0.075 mmol). The mixture was stirred at room temperature for 10 min and then cooled to 0 °C. $NaBH_4$ (2 mg, 0.050 mmol) was added and the reaction system was stirred for 30 min at 0 °C and 1 h at rt. Upon completion, the reaction was quenched by addition of sat. NH_4Cl (aq) (3 mL) and the mixture was extracted with EA (10 mL × 3). The organic layers were combined, washed with brine (5 mL), dried over Na_2SO_4, filtered and concentrated under reduced pressure. Upon subjection of the crude product to silica gel column chromatography (eluent: EA/PE = 1/1), alcohol intermediate fully cyclized to give diene **30** (16.7 mg, 84% yield over two steps). $[\alpha]_D^{23}$= -32.3 (c = 0.52, $CHCl_3$); ^1H NMR (500 MHz, $CDCl_3$) δ 7.14 (t, *J* = 8.0 Hz, 1H), 7.11 (d, *J* = 7.4 Hz, 1H), 6.97 (d, *J* = 8.0 Hz, 1H), 6.94 (t, *J* = 7.4 Hz, 1H), 6.80 (d, *J* = 10.4 Hz, 1H), 6.09 (d, *J* = 10.4 Hz, 1H), 5.00 (s, 1H), 4.93 (s, 1H), 3.60-3.47 (m, 1H), 3.10 (dd, *J* = 17.7, 10.2 Hz, 1H), 2.73 (s, 3H), 2.71 (s, 3H), 2.57 (br s, 6H), 2.47 (br s, 2H), 2.03-1.93 (m, 2H), 1.44 (s, 9H); ^{13}C NMR (126 MHz, $CDCl_3$) δ 152.9, 144.9, 139.1, 136.3, 128.0, 127.7, 126.9, 125.3, 121.7, 115.8, 114.1, 87.7, 79.2, 60.4, 55.6, 46.2, 37.4, 37.4, 37.1, 29.8, 28.5; ESI-MS: 481.2 $[M+Na]^+$; HRMS (ESI) calculated for $[M+Na, C_{24}H_{35}N_4NaO_3P]^+$: 481.2344; found: 481.2342.

Preparation of compound 31

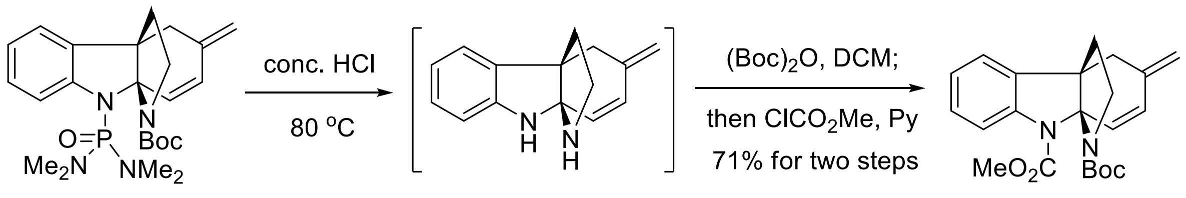

30 35 31

A mixture of diene **30** (30 mg, 0.066 mmol) and conc. HCl (aq) (3 mL) in a sealed tube was heated at 80 °C for 10 h. The reaction solution was cooled to 0 °C and neutralized to pH = 9 with 2 *N* NaOH (aq). The

mixture was extracted with EA (10 mL × 3). The organic phases were combined, washed with brine (10 mL), dried over Na_2SO_4, filtered and concentrated under reduced pressure to give diamine **35**.

To a solution of crude diamine **35** in anhydrous DCM (5 mL) was added $(Boc)_2O$ (17 mg, 0.079 mmol) and the resulting mixture was stirred at rt for 8 h. Pyridine (11 μL, 0.13 mmol) and methyl chloroformate (10 μL, 0.13 mmol) were added to the reaction solution. The mixture was stirred for 3 h. Upon completion, water (5 mL) was added to quench the reaction. The organic phase was separated and the aqueous layer was extracted with DCM (6 mL × 3). The organic phases were combined, washed once with brine (4 mL), dried over Na_2SO_4, filtered and concentrated under reduced pressure. The crude product was purified by column chromatography (eluent: EA/PE = 1/4) to give diene **31** (17.8 mg, 71% yield). $[\alpha]_D^{23}$= -18.0 (c = 0.50, $CHCl_3$); ^{1}H NMR (500 MHz, $CDCl_3$) δ 7.67 (s, 1H), 7.27-7.21 (m, 1H), 7.16 (t, *J* = 6.4 Hz, 1H), 7.06 (dd, *J* = 9.5, 5.3 Hz, 1H), 6.61 (s, 1H), 6.15 (d, *J* = 10.2 Hz, 1H), 5.06 (s, 1H), 4.99 (s, 1H), 3.84 (s, 3H), 3.51 (ddd, *J* = 10.6, 8.5, 1.9 Hz, 1H), 3.08 (dd, *J* = 17.8, 9.5 Hz, 1H), 2.52-2.42 (m, 2H), 2.08-1.95 (m, 2H), 1.46 (s, 9H); ^{13}C NMR (126 MHz, $CDCl_3$) δ 153.6, 141.1, 138.7, 128.3, 128.0, 126.3, 125.7, 124.3, 123.6, 121.7, 117.8, 115.2, 80.4, 70.2, 52.4, 45.9, 37.7, 37.0, 29.7, 28.6, 20.7; ESI-MS: 405.3 $[M+Na]^+$; HRMS (ESI) calculated for $[M+Na, C_{22}H_{26}N_2NaO_4]^+$: 405.1790; found: 405.1785;

Preparation of compound 32

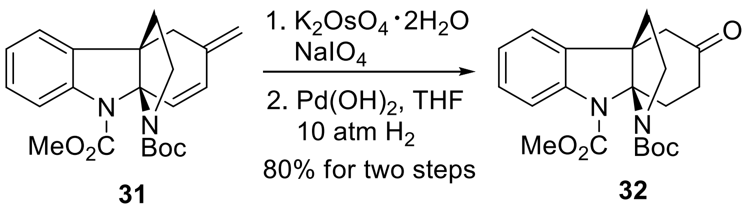

To a solution of diene **31** (25 mg, 0.065 mmol) in THF (3 mL) and water (1 mL) was added potassium osmate dihydrate (5 mg, 0.013 mmol) and $NaIO_4$ (34 mg, 0.16 mmol). The resulting mixture was stirred at

rt for 20 h. Upon fully consumption of diene **31**, saturated sodium thiosulfate solution (5 mL) was added. After being stirred for 10 min, the mixture was extracted with DCM (6 mL × 3). The organic phases were combined, washed once with brine (4 mL), dried over Na_2SO_4, filtered and concentrated under reduced pressure. The residue was dissolved in THF (5 mL) and stirred in the presence of $Pd(OH)_2$ (6 mg) under 10 atm H_2 for 12 h. The reaction mixture was filtered through a layer of Celite. The catalyst was washed with MeOH (5 mL × 3) and the filtrates were combined and concentrated under reduced pressure. Ketone **32** was purified by column chromatography (eluent: EA/PE = 1/8) (20 mg, 80% yield). $[\alpha]_D^{23}$= 55.9 (c = 0.30, $CHCl_3$), literature $[\alpha]_D$ = -157 (c = 0.52, $CHCl_3$) for antipode of **32**[7]; ^{1}H NMR (500 MHz, C_6D_6, 60 °C) δ 8.08 (d, *J* = 8.3 Hz, 1H), 7.02 (t, *J* = 7.8 Hz, 1H), 6.76 (t, *J* = 7.5 Hz, 1H), 6.62 (d, *J* = 7.0 Hz, 1H), 3.56 (s, 3H), 3.59-3.53 (m, 1H), 3.11 (ddd, *J* = 14.3, 7.9, 4.5 Hz, 1H), 2.76 (td, *J* = 11.3, 6.4 Hz, 1H), 2.55-2.48 (m, 1H), 2.30 (q, *J* = 15.2 Hz, 2H), 2.15 (ddd, *J* = 18.6, 9.1, 4.4 Hz, 1H), 1.96 (ddd, *J* = 18.7, 8.0, 4.5 Hz, 1H), 1.39 (s, 9H), 1.44-1.31 (m, 2H); ^{13}C NMR (126 MHz, C_6D_6) δ 206.6, 153.2, 152.3, 142.3, 133.4, 128.7, 123.3, 122.4, 115.2, 88.7, 79.3, 56.9, 51.7, 47.8, 45.2, 38.7, 34.6, 28.9, 28.0; ESI-MS: 409.3 $[M+Na]^+$; HRMS (ESI) calculated for $[M+Na, C_{21}H_{26}N_2NaO_5]^+$: 409.1739; found: 409.1738. The ^{1}H and ^{13}C NMR were in full agreement with reported data.[7]

9. Computation results

To rationalize the distinct chemoselectivity observed with different *N*-R groups, the substrate structures of **8b** and **8i** were analyzed with the aid of DFT calculations. As shown in Figure 1, the distance between CBr and CMe in substrate **8i** (3.526 Å) is much shorter than that in substrate **8b** (3.910 Å). This is in accordance with the observed formation of dearomative cyclization product **9i** via a shorter CBr--CMe distance for substrate **8i**. NBO analysis demonstrates that the charge on CMe of **8i** is -0.071, more negative

than that of **8b** (-0.050) and that for the C2 of **8i** (-0.017). More nucleophilicity of CMe in substrate **8i** could be another driving force for preference of the dearomatization pathway for substrate **8i**.

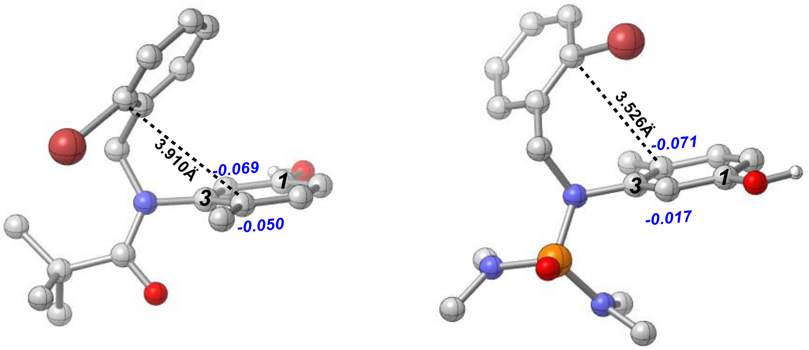

8b **8i**

Figure 1. Optimized structure of substrates **8b** and **8i** at the B3LYP/6-31+G(d,p) level.1 Black bold numbers are distances between CBr and CMe. Blue italic numbers are NPA charges on the carbon atom plus the attached hydrogen. Hydrogens (except the OH) are omitted for clarity.

First, the conformation searches of the substrate structures **8b** and **8i** were performed using Macromodel[8] (Mixed MCMM/Low-mode sampling method[9] and OPLS_2005 force field[10]) with the default settings. Then, the conformation of the lowest energy was optimized at B3LYP[11]/6-31+G(d,p) level (ultrafine integral grid, 5d), with SMD solvation model (solvent=toluene), and Grimme's dispersion (GD3),[12] by using Gaussian 09 software package.[13] NBO population analysis was performed with NBO module[14] in Gaussian 09. The 3D model was generated with CYL view.[15]

Cartesian coordinates and energies of all the optimized structures 8b and 8i

8b

Opt @ B3LYP-GD3/6-31+G(d,p)/5d in toluene (SMD model)
SCF Done: E(RB3LYP) = -3514.01219296 A.U.
Zero-point correction= 0.371438 Hartree/Particle
Sum of electronic and thermal Free Energies= -3513.693824 A.U.
N,-0.1029534167,-1.2683525255,-0.3011891152
C,-1.3664123423,-1.142026445,0.3967904394
C,-1.4559531977,-0.5468199217,1.6641172886
C,-2.7413365389,-0.3960665762,2.2078262238
C,-3.8913198055,-0.8140164805,1.5397095146
C,-3.7718988512,-1.4179691005,0.2854254496

C,-2.5077358506,-1.5784344375,-0.2849678008
O,-4.9234886532,-1.8265684333,-0.3391387546
C,0.0448536413,-0.3342469271,-1.4360199188
C,-0.2162537997,1.1049060801,-1.0325469634
C,-1.4824396219,1.6707853554,-1.2481253277
C,-1.7785412432,2.9787223432,-0.8654010451
C,-0.7971772716,3.7587789692,-0.2495744223
C,0.4760586935,3.22949405,-0.0296243541
C,0.7482754349,1.9183620695,-0.4219207856
Br,2.5314665787,1.2570192872,-0.1045427396
C,0.730960942,-2.2898183058,0.1207833729
O,0.4380147708,-2.8797946205,1.1643274837
C,-0.2361501606,-0.1267506062,2.4396571068
C,1.9897716607,-2.8226121032,-0.6376615131
C,3.1893469342,-2.649159151,0.3212431983
C,1.7156933955,-4.3365676731,-0.8369331541
C,2.3712999479,-2.2467465456,-2.014536213
H,-2.8429066962,0.0643905554,3.1873532947
H,-4.87578797,-0.6855239472,1.9786968052
H,-2.3992683624,-2.0489880702,-1.2597362369
H,-4.7105082087,-2.2773652226,-1.1681449227
H,1.0435930554,-0.4161160747,-1.8388615109
H,-0.6591061207,-0.6096904975,-2.2299456806
H,-2.2506154331,1.057073081,-1.709407276
H,-2.7713848151,3.3819305252,-1.0404815836
H,-1.0131377992,4.7778182727,0.0577847325
H,1.2478132458,3.828684152,0.4410308844
H,-0.4822771776,0.6477197486,3.1725797541
H,0.5489331412,0.2582730595,1.7868405684
H,0.1885879052,-0.9869985892,2.969419096
H,2.982996633,-3.112172542,1.2892338048
H,4.0798835892,-3.1205410559,-0.1101089634
H,3.4145229381,-1.5907018916,0.4871316406
H,2.595181457,-4.8131530059,-1.2841489883
H,1.4964282285,-4.8252799284,0.1139959396
H,0.8658557342,-4.5002275411,-1.5109754472
H,2.7405313402,-1.2190746568,-1.9610245843
H,3.1923517251,-2.8494275465,-2.4193792804
H,1.550232344,-2.3027471263,-2.7365500166

8i

Opt @ B3LYP-GD3/6-31+G(d,p)/5d in toluene (SMD model)
SCF Done: E(RB3LYP) = -3928.61340133 A.U.
Zero-point correction = 0.411262 Hartree/Particle
Sum of electronic and thermal Free Energies= -3928.259786 A.U.

N,-0.3565717309,-0.2841055129,-0.3929839484
C,-1.6002093925,0.412911898,-0.1398995247
C,-1.7235238752,1.2549413415,0.9822369643
C,-2.9409595555,1.9321113829,1.1485808346
C,-4.0028528167,1.7876916688,0.2567579379
C,-3.8602666522,0.9378959874,-0.8457040177
C,-2.6599544526,0.2579564854,-1.0430909036
O,-4.858879259,0.7351983392,-1.7635157742
C,0.5382709662,0.3920682504,-1.3755615195
C,1.1678723152,1.6474954556,-0.8093397161
C,2.3192770967,1.5214586203,-0.0132482169
C,2.9262000793,2.6194540864,0.5925001847
C,2.3843940784,3.8950237864,0.4088130784
C,1.2540280861,4.0582580936,-0.3920701828
C,0.6610262798,2.9426045176,-0.9915003784
Br,-0.8768784778,3.2750583209,-2.1013995266
C,-0.6135558472,1.4600727757,1.9840311451
P,-0.4423435153,-1.9830038014,-0.5410339309
N,-1.5552329544,-2.4784014715,0.6185520286
N,1.1311528702,-2.4376623851,-0.1256290736
C,-2.8554924792,-3.0055737308,0.2038348731
C,-1.5189356895,-2.041844408,2.0112285422
C,1.7772853736,-2.0128678838,1.1131499145
C,1.5605601206,-3.7687640811,-0.5651600789
O,-0.8045683474,-2.5281251655,-1.891498363
H,-3.0611100588,2.5914019913,2.0049567506
H,-4.9326000025,2.329061028,0.4180204947
H,-2.5472379119,-0.3904069185,-1.9058904434
H,-5.6321509242,1.2696758301,-1.5351989752
H,1.3310831993,-0.3149057208,-1.6285039408
H,-0.0154792065,0.6107536562,-2.2941029831
H,2.7388023747,0.5299512603,0.1274053817
H,3.8147305012,2.4826164716,1.2019368293
H,2.8417674823,4.763207893,0.8744295501

H,0.8337193352,5.0451220453,-0.5519578135
H,0.129896831,0.6656443415,1.92318939
H,-0.0895900331,2.4068060347,1.8075616149
H,-1.0109846703,1.4875931753,3.0046815475
H,-2.8085886302,-3.3424245839,-0.8317542091
H,-3.1235060488,-3.8584612663,0.8410186908
H,-3.6422619338,-2.2413823356,0.2931850101
H,-2.308721041,-1.3052051685,2.2153864822
H,-1.6671996989,-2.9023597244,2.6770963859
H,-0.5645727556,-1.5813014377,2.2615788728
H,1.4590206661,-1.0060721408,1.3837391156
H,1.5625245335,-2.6940453593,1.9518997182
H,2.8646430078,-1.9976477668,0.9658025175
H,2.6510634342,-3.7725953241,-0.6808836572
H,1.2880158444,-4.5511989908,0.1613164577
H,1.1068934855,-4.0066795598,-1.5279631353

10. References

1. (a) W. Tang, A. G. Capacci, X. Wei, W. Li, A. White, N. D. Patel, J. Savoie, J. J. Gao, S. Rodriguez, B. Qu, N. Haddad, B. Z. Lu, D. Krishnamurthy, N. K. Yee, C. H. Senanayake, *Angew. Chem. Int. Ed.* **2010**, *49*, 5879; *Angew. Chem.* **2010**, *122*, 6015. (b) Q. Zhao, C. Li, C. H. Senanayake, W. Tang, *Chem. Eur. J.* **2013**, *19*, 2261. (c) G. Xu, W. Fu, G. Liu, C. H. Senanayanke, W. Tang, *J. Am. Chem. Soc.* **2014**, *136*, 570. (d) W. Fu, M. Nie, A. Wang, Z. Cao, W. Tang, *Angew. Chem. Int. Ed.* **2015**, *54*, 2520; *Angew. Chem.* **2015**, *127*, 2550. (e) K. Li, N. Hu, R. Luo, W. Yuan, W. Tang, *J. Org. Chem.* **2013**, *78*, 6350.

2. L. E. Overman, S. Sugai, *Helv. Chim. Acta* **1985**, *68*, 745.

3. W. H. Pearson, F. E. Lovering, *J. Org. Chem.* **1998**, *63*, 3607.

4. F. Viladomat, J. Bastida, C. Codina, W. E. Campbell, S. Mathee, *Phytochemistry* **1995**, *40*, 307.

5. M.-X. Wei, C.-T. Wang, J.-Y. Du, H. Qu, P.-R. Yin, X. Bao, X.-Y. Ma, X.-H. Zhao, G.-B. Zhang, C.-A. Fan, *Chem. Asian J.* **2013**, *8*, 1966

6. V. Pabuccuoglu, P. Richomme, T. Gozler, B. Kivcak, A. J. Freyer, M. Shamma, *J. Nat. Prod.* **1989**, *52*, 785.

7. A. B. Dounay, P. G. Humphreys, L. E. Overman, A. D. Wrobleski, *J. Am. Chem. Soc.* **2008,** *130*, 5368.

8. MacroModel, *version 11.3*, Schrödinger, LLC, New York, NY, **2016**.

9. (a) G. Chang, W. C. Guida, W. C. Still, *J. Am. Chem. Soc.* **1989**, *111*, 4379; (b) P. Labute, *J. Chem. Inf. Model.* **2010**, *50*, 792.

10. J. L. Banks, H. S. Beard, Y. Cao, A. E. Cho, W. Damm, R. Farid, A. K. Felts, T. A. Halgren, D. T. Mainz, J. R. Maple, R. Murphy, D. M. Philipp, M. P. Repasky, L. Y. Zhang, B. J. Berne, R. A. Friesner, E. Gallicchio, R. M. Levy, *J. Comput. Chem.* **2005**, *26*, 1752.

11. (a) A. D. Becke, *J. Chem. Phys.* **1993**, *98*, 5648; (b) C. Lee, W. Yang, R. G. Parr, *Phys. Rev. B* **1988**, *37*, 785.

12. S. Grimme, J. Antony, S. Ehrlich, H. Krieg, *J. Chem. Phys.* **2010**, *132*, 154104.

13. M. J. Frisch, G. W. T., H. B. Schlegel, G. E. Scuseria, M. A. Robb, J. R. Cheeseman, G. Scalmani, V. Barone, B. Mennucci, G. A. Petersson, H. Nakatsuji, M. Caricato, X. Li, H. P. Hratchian, A. F. Izmaylov, J. Bloino, G. Zheng, J. L. Sonnenberg, M. Hada, M. Ehara, K. Toyota, R. Fukuda, J. Hasegawa, M. Ishida, T. Nakajima, Y. Honda, O. Kitao, H. Nakai, T. Vreven, J. A. Montgomery, Jr., J. E. Peralta, F. Ogliaro, M. Bearpark, J. J. Heyd, E. Brothers, K. N. Kudin, V. N. Staroverov, R. Kobayashi, J. Normand, K. Raghavachari, A. Rendell, J. C. Burant, S. S. Iyengar, J. Tomasi, M. Cossi, N. Rega, J. M. Millam, M. Klene, J. E. Knox, J. B. Cross, V. Bakken, C. Adamo, J. Jaramillo, R.

Gomperts, R. E. Stratmann, O. Yazyev, A. J. Austin, R. Cammi, C. Pomelli, J. W. Ochterski, R. L. Martin, K. Morokuma, V. G. Zakrzewski, G. A. Voth, P. Salvador, J. J. Dannenberg, S. Dapprich, A. D. Daniels, Ö. Farkas, J. B. Foresman, J. V. Ortiz, J. Cioslowski, and D. J. Fox. *Gaussian 09*, revision D.01; Gaussian, Inc. Wallingford, CT: 2009.

14. NBO Version 3.1, E. D. Glendening, A. E. Reed, J. E. Carpenter, and F. Weinhold.

15. CYLview, 1.0b; Legault, C. Y., Université de Sherbrooke, 2009 (http://www.cylview.org).

11. NMR spectra

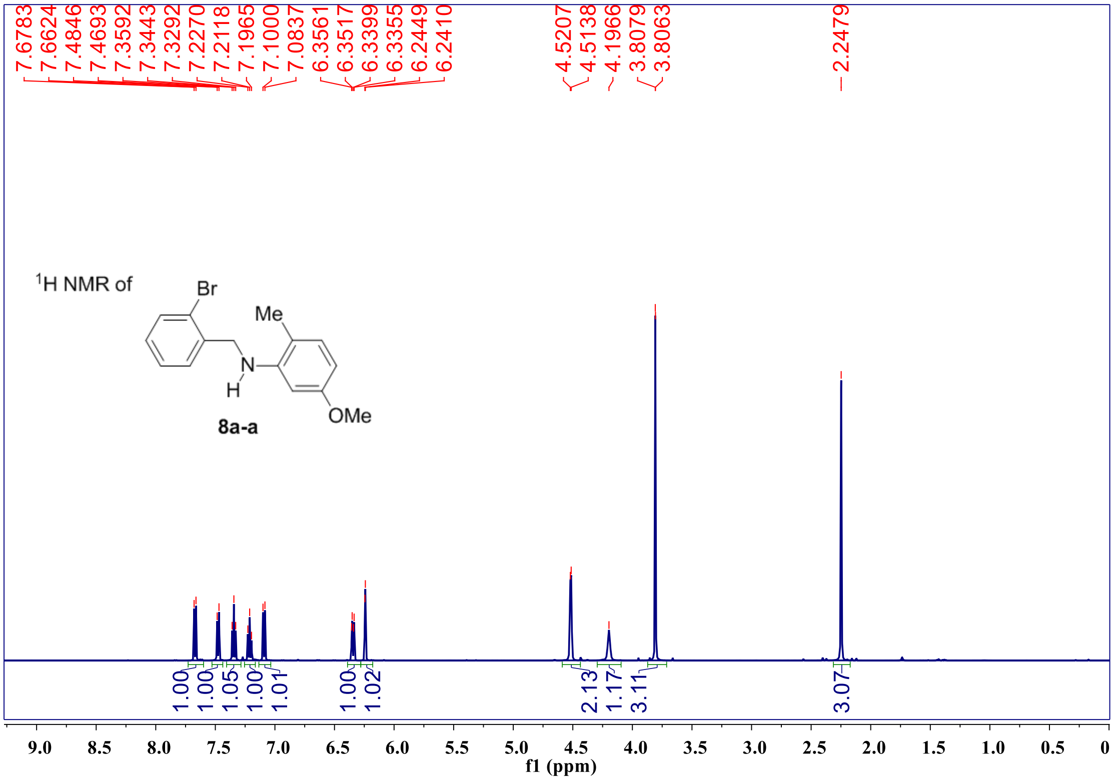

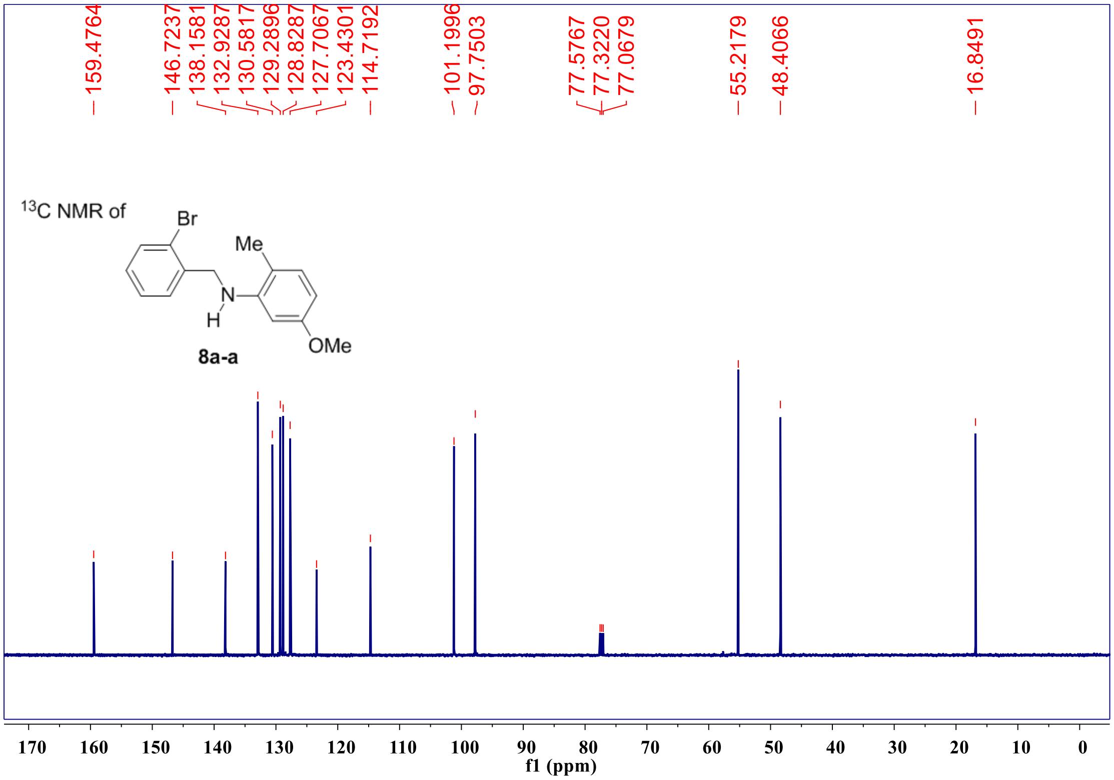

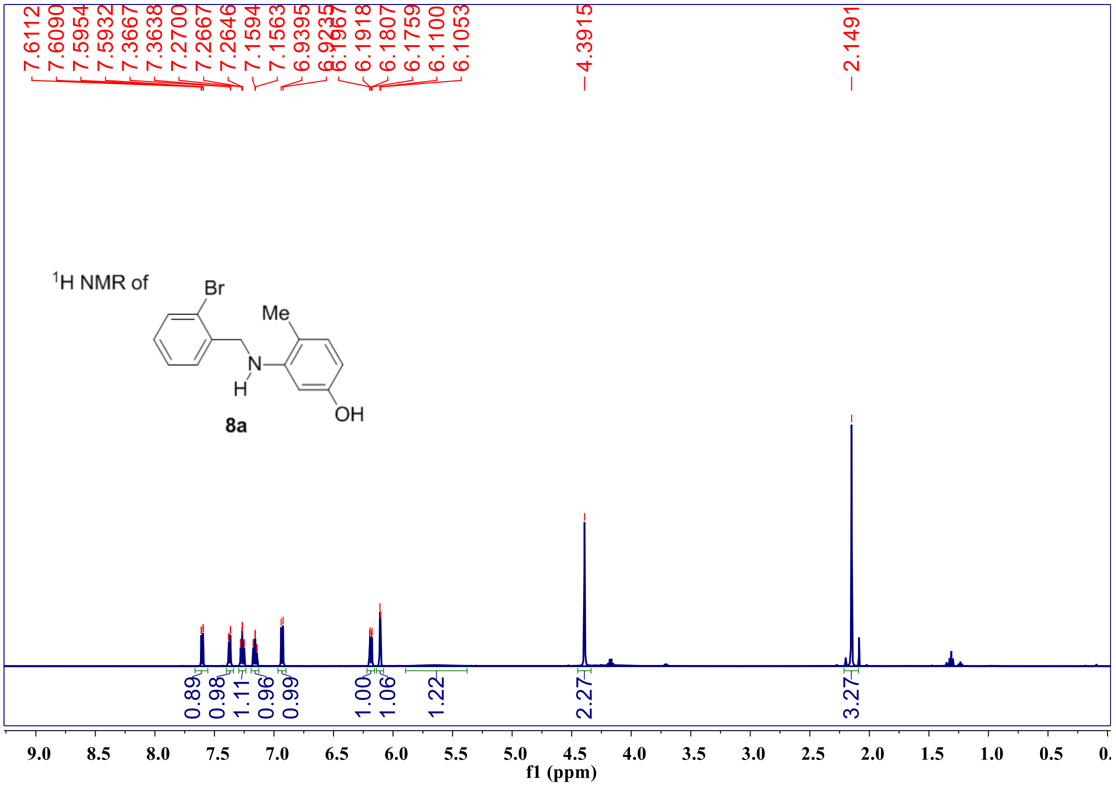
7.6112
7.6090
7.5954
7.5932
7.3667
7.3638
7.2700
7.2667
7.2646
7.1594
7.1563
6.9395
6.1918
6.1807
6.1759
6.1100
6.1053
4.3915
2.1491
1H NMR of
Br
Me
N
H
OH
8a
0.89
0.98
1.11
0.96
0.99
1.00
1.06
1.22
2.27
3.27
f1 (ppm)

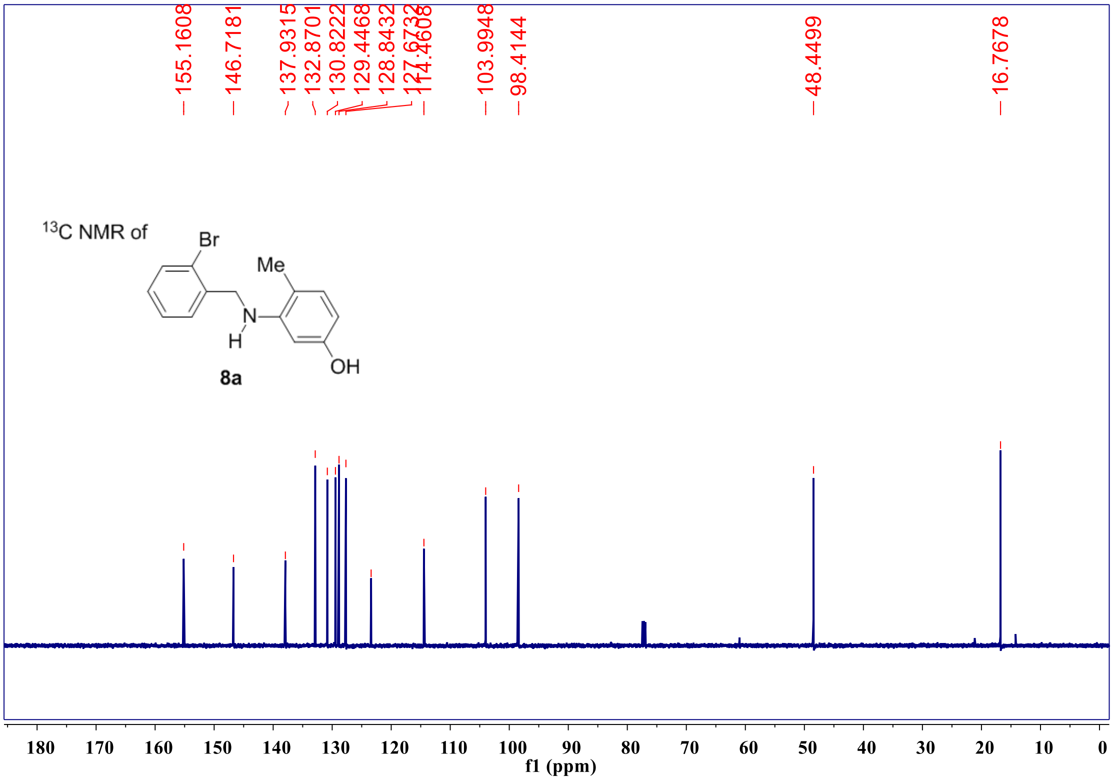
155.1608
146.7181
137.9315
132.8701
130.8222
129.4468
128.8432
114.4608
103.9948
98.4144
48.4499
16.7678
13C NMR of
Br
Me
N
H
OH
8a
f1 (ppm)

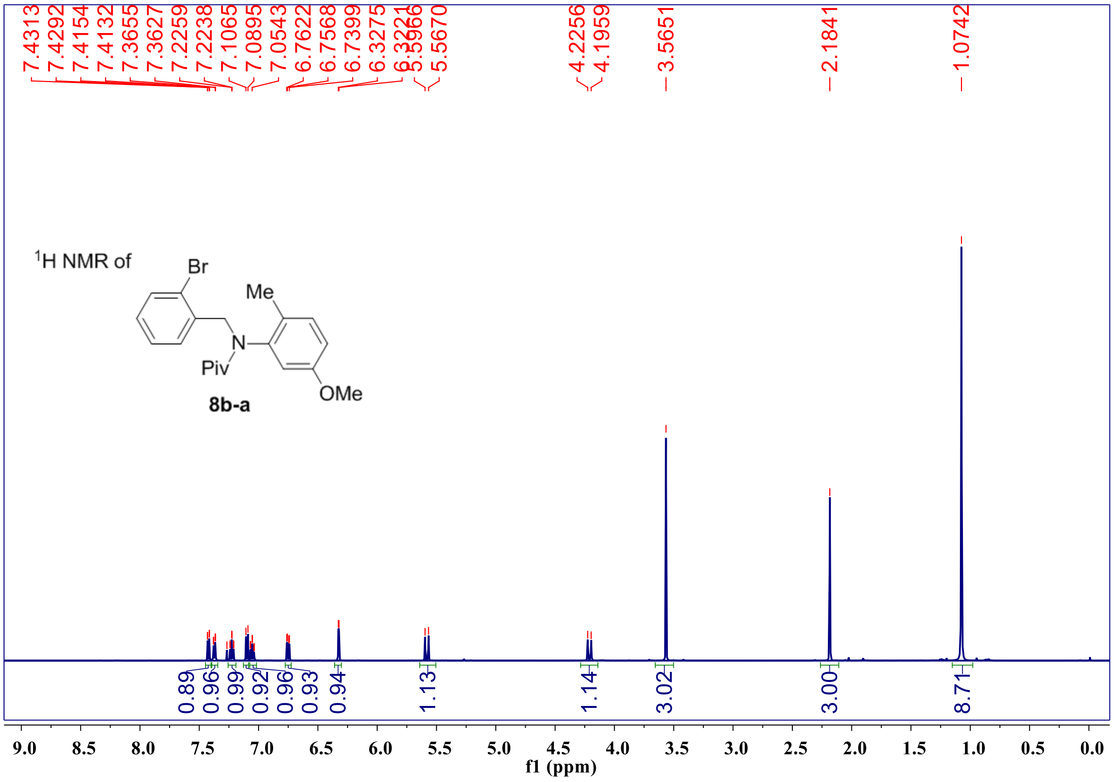

7.4313
7.4292
7.4154
7.4132
7.3655
7.3627
7.2259
7.2238
7.1065
7.0895
7.0543
6.7622
6.7568
6.7399
6.3275
6.3221
5.5966
5.5670
4.2256
4.1959
3.5651
2.1841
1.0742
^{1}H NMR of
Br
Me
N
Piv
OMe
8b-a
0.89
0.96
0.99
0.92
0.96
0.93
0.94
1.13
1.14
3.02
3.00
8.71
9.0 8.5 8.0 7.5 7.0 6.5 6.0 5.5 5.0 4.5 4.0 3.5 3.0 2.5 2.0 1.5 1.0 0.5 0.0
f1 (ppm)

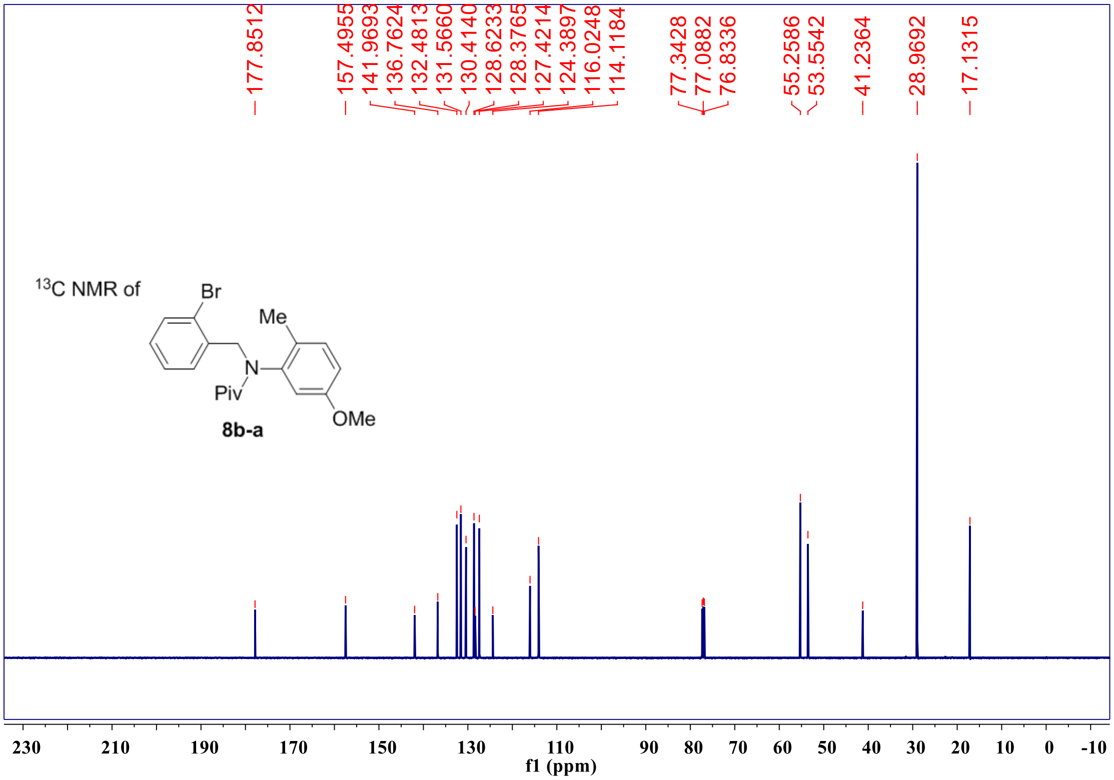

177.8512
157.4955
141.9693
136.7624
132.4813
131.5660
130.4140
128.6233
128.3765
127.4214
124.3897
116.0248
114.1184
77.3428
77.0882
76.8336
55.2586
53.5542
41.2364
28.9692
17.1315
^{13}C NMR of
Br
Me
N
Piv
OMe
8b-a
230 210 190 170 150 130 110 90 80 70 60 50 40 30 20 10 0 -10
f1 (ppm)

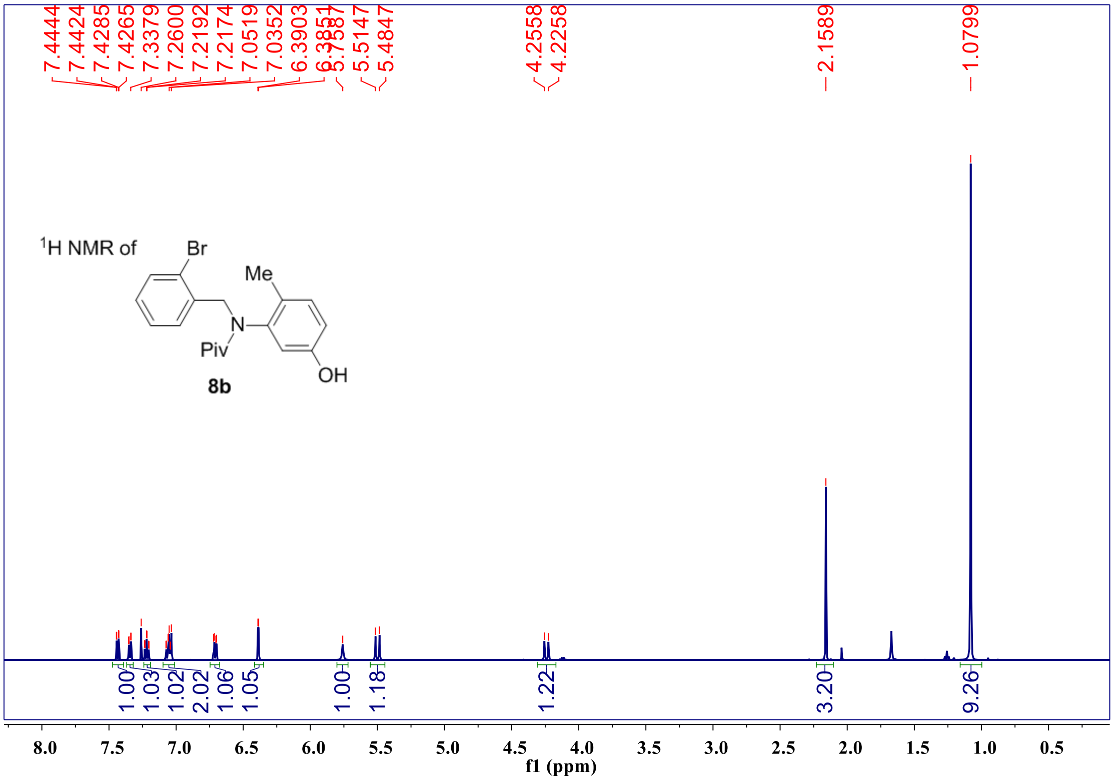
1H NMR of
8b
f1 (ppm)

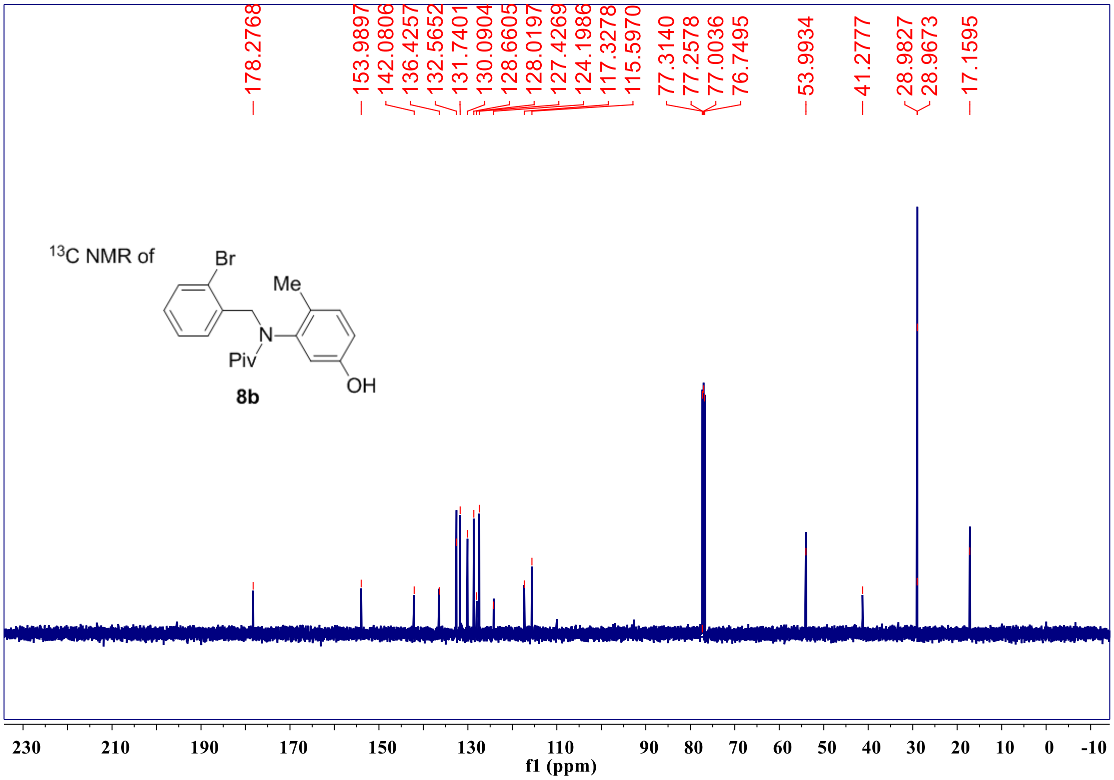
13C NMR of
8b
f1 (ppm)

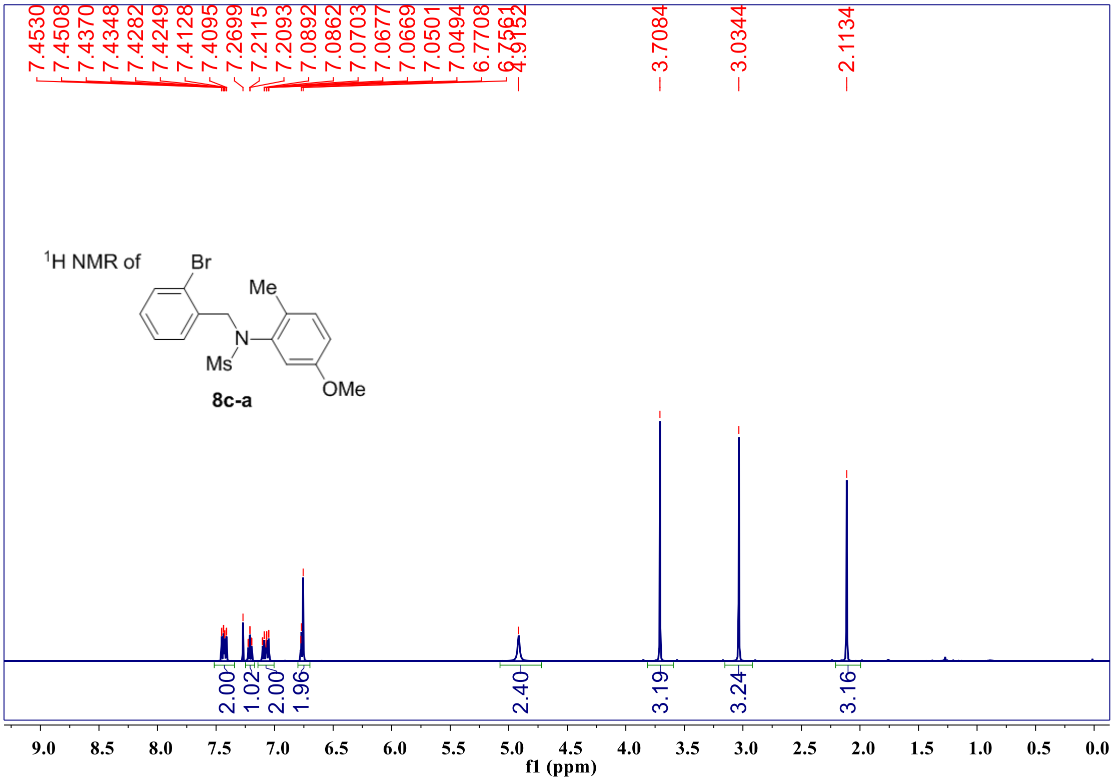
7.4530
7.4508
7.4370
7.4348
7.4282
7.4249
7.4128
7.4095
7.2699
7.2115
7.2093
7.0892
7.0862
7.0703
7.0677
7.0669
7.0501
7.0494
6.7708
6.7561
4.9152
3.7084
3.0344
2.1134
1H NMR of
Br
Me
N
Ms
OMe
8c-a
2.00
1.02
2.00
1.96
2.40
3.19
3.24
3.16
f1 (ppm)

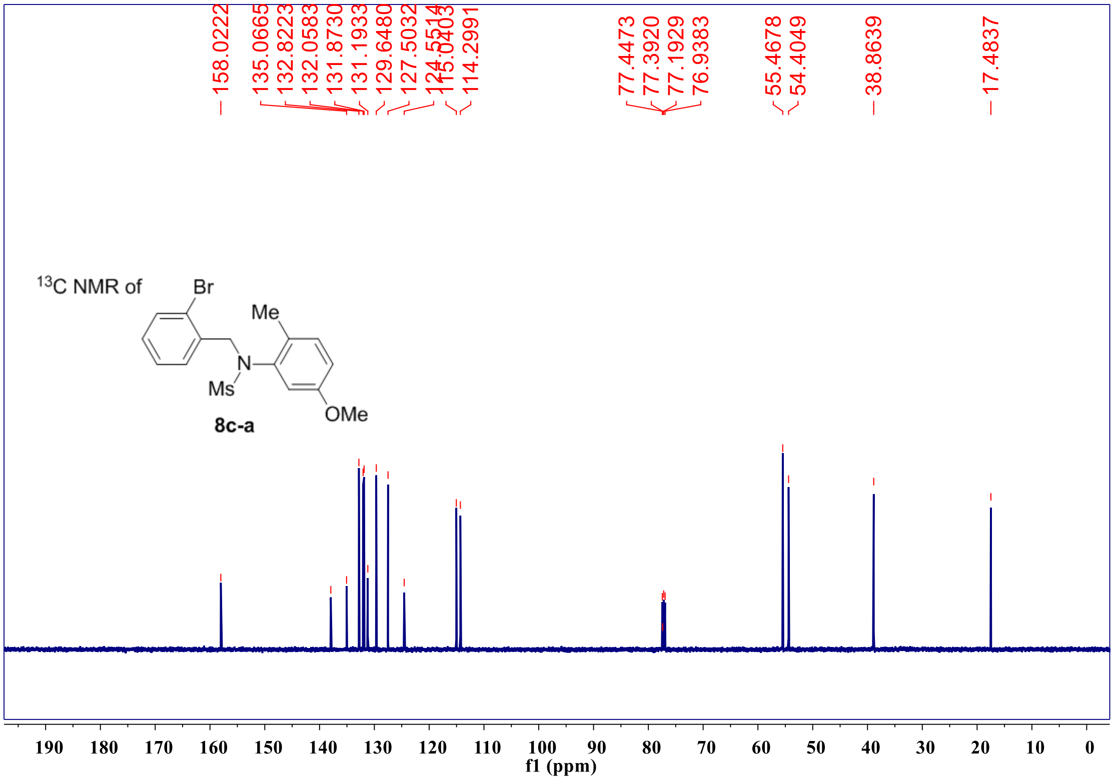
158.0222
135.0665
132.8223
132.0583
131.8730
131.1933
129.6480
127.5032
124.5514
115.0403
114.2991
77.4473
77.3920
77.1929
76.9383
55.4678
54.4049
38.8639
17.4837
13C NMR of
Br
Me
N
Ms
OMe
8c-a
f1 (ppm)

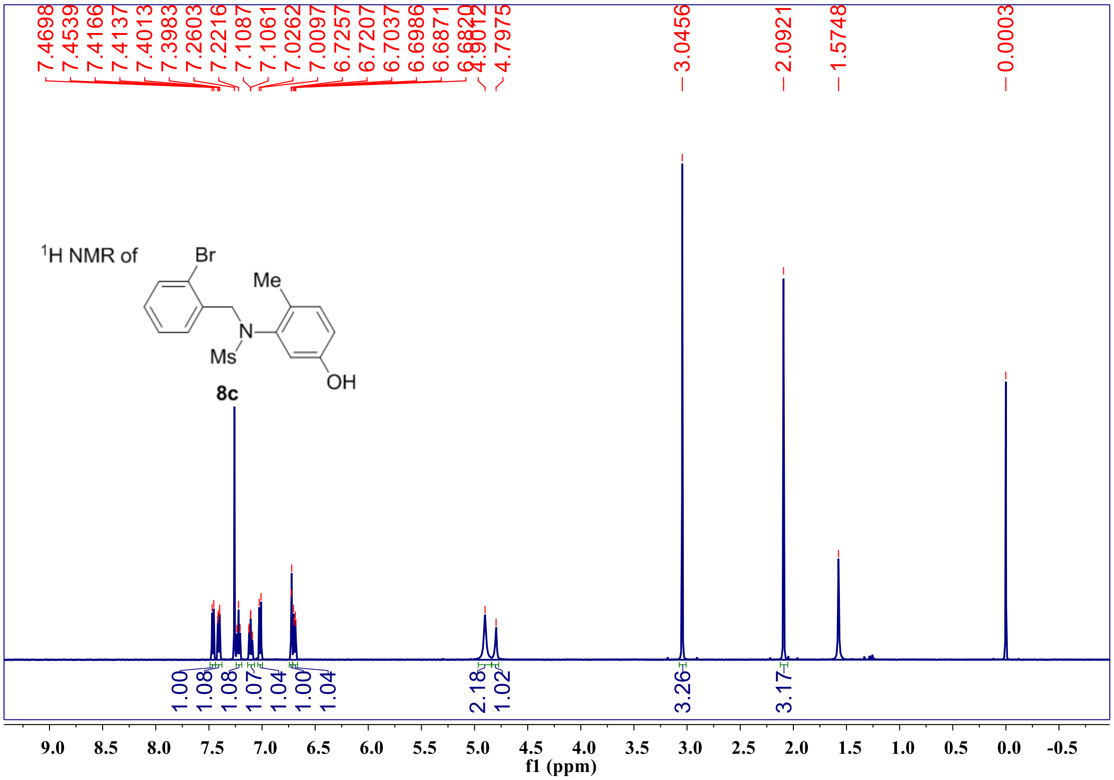

7.4698
7.4539
7.4166
7.4137
7.4013
7.3983
7.2603
7.2216
7.1087
7.1061
7.0262
7.0097
6.7257
6.7207
6.7037
6.6986
6.6871
6.6820
4.9012
4.7975
3.0456
2.0921
1.5748
0.0003
^{1}H NMR of
Br
Me
N
Ms
OH
8c
1.00
1.08
1.08
1.07
1.04
1.00
1.04
2.18
1.02
3.26
3.17
f1 (ppm)

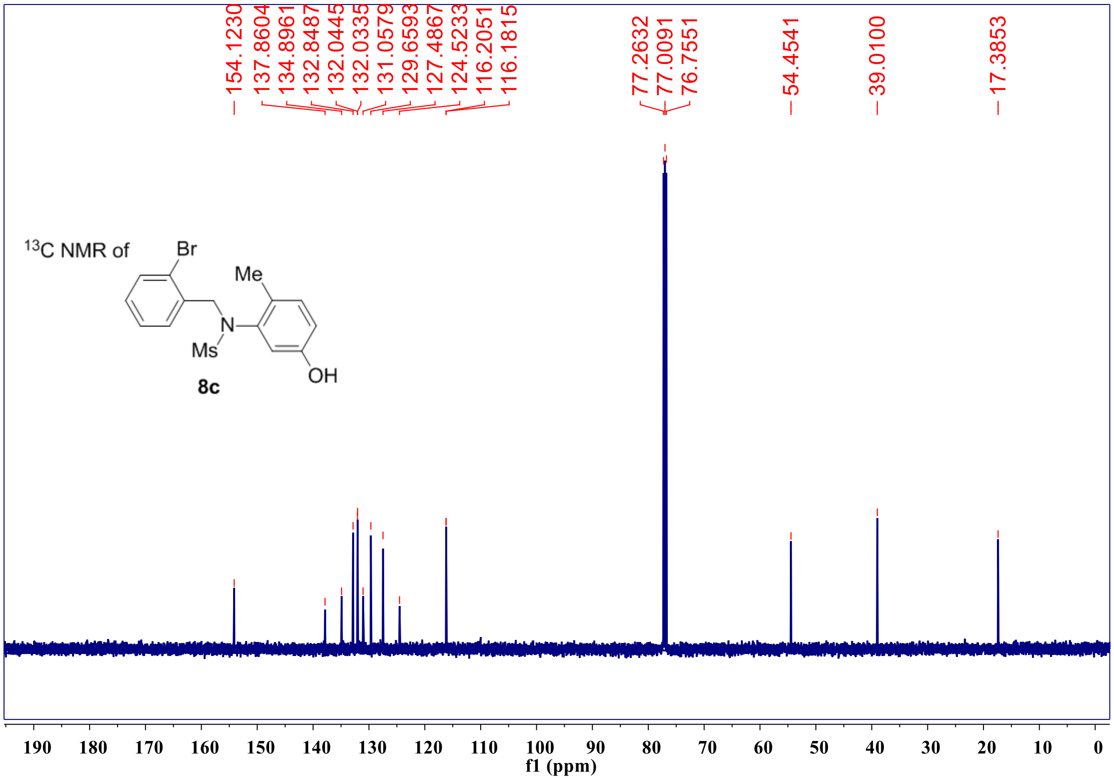

154.1230
137.8604
134.8961
132.8487
132.0445
132.0335
131.0579
129.6593
127.4867
124.5233
116.2051
116.1815
77.2632
77.0091
76.7551
54.4541
39.0100
17.3853
^{13}C NMR of
Br
Me
N
Ms
OH
8c
f1 (ppm)

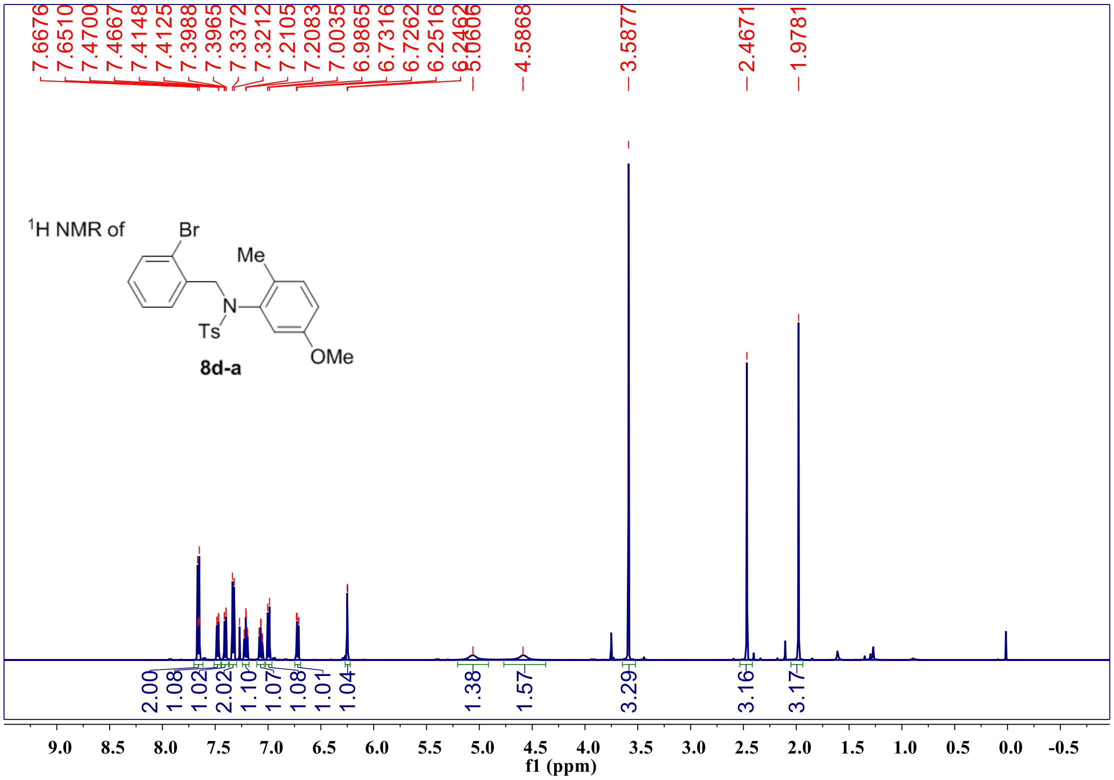

7.6676
7.6510
7.4700
7.4667
7.4148
7.4125
7.3988
7.3965
7.3372
7.3212
7.2105
7.2083
7.0035
6.9865
6.7316
6.7262
6.2516
6.2462
5.0606
4.5868
3.5877
2.4671
1.9781
^{1}H NMR of
Br
Me
N
Ts
OMe
8d-a
2.00
1.08
1.02
2.02
1.10
1.07
1.08
1.01
1.04
1.38
1.57
3.29
3.16
3.17
9.0 8.5 8.0 7.5 7.0 6.5 6.0 5.5 5.0 4.5 4.0 3.5 3.0 2.5 2.0 1.5 1.0 0.5 0.0 -0.5
f1 (ppm)

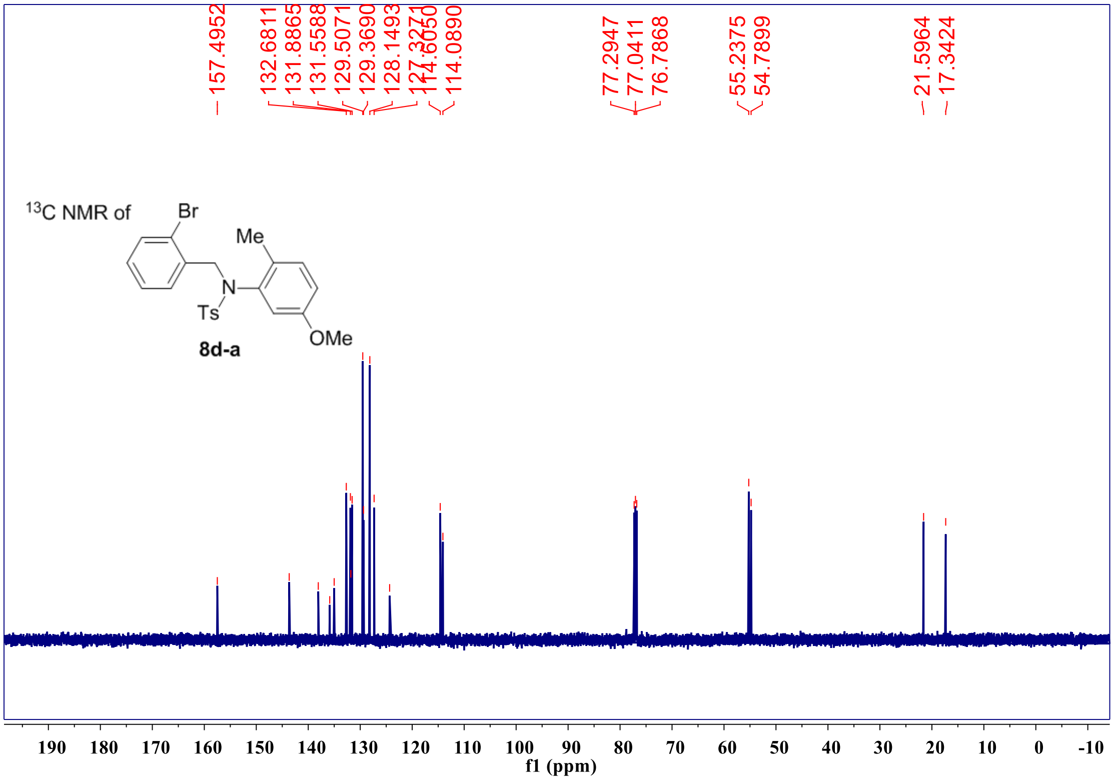

157.4952
132.6811
131.8865
131.5588
129.5071
129.3690
128.1493
127.3271
114.6050
114.0890
77.2947
77.0411
76.7868
55.2375
54.7899
21.5964
17.3424
^{13}C NMR of
Br
Me
N
Ts
OMe
8d-a
190 180 170 160 150 140 130 120 110 100 90 80 70 60 50 40 30 20 10 0 -10
f1 (ppm)

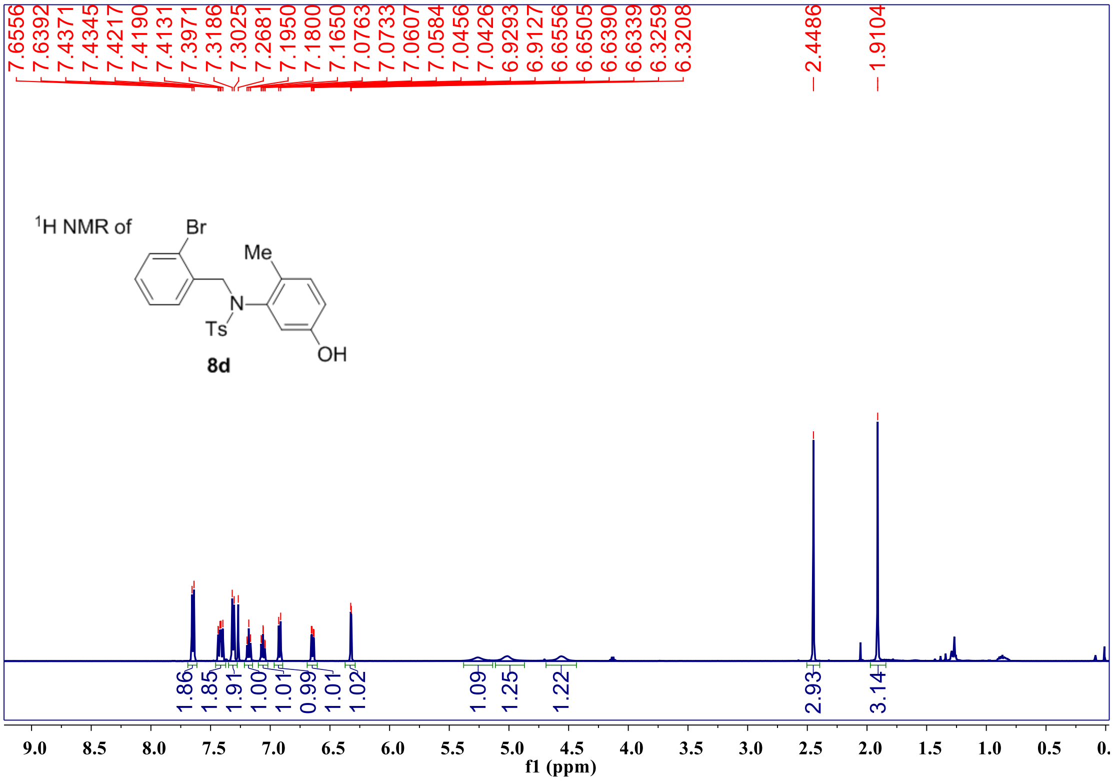

7.6556
7.6392
7.4371
7.4345
7.4217
7.4190
7.4131
7.3971
7.3186
7.3025
7.2681
7.1950
7.1800
7.1650
7.0763
7.0733
7.0607
7.0584
7.0456
7.0426
6.9293
6.9127
6.6556
6.6505
6.6390
6.6339
6.3259
6.3208
2.4486
1.9104
^{1}H NMR of
Br
Me
N
Ts
OH
8d
1.86
1.85
1.91
1.00
1.01
0.99
1.01
1.02
1.09
1.25
1.22
2.93
3.14
f1 (ppm)

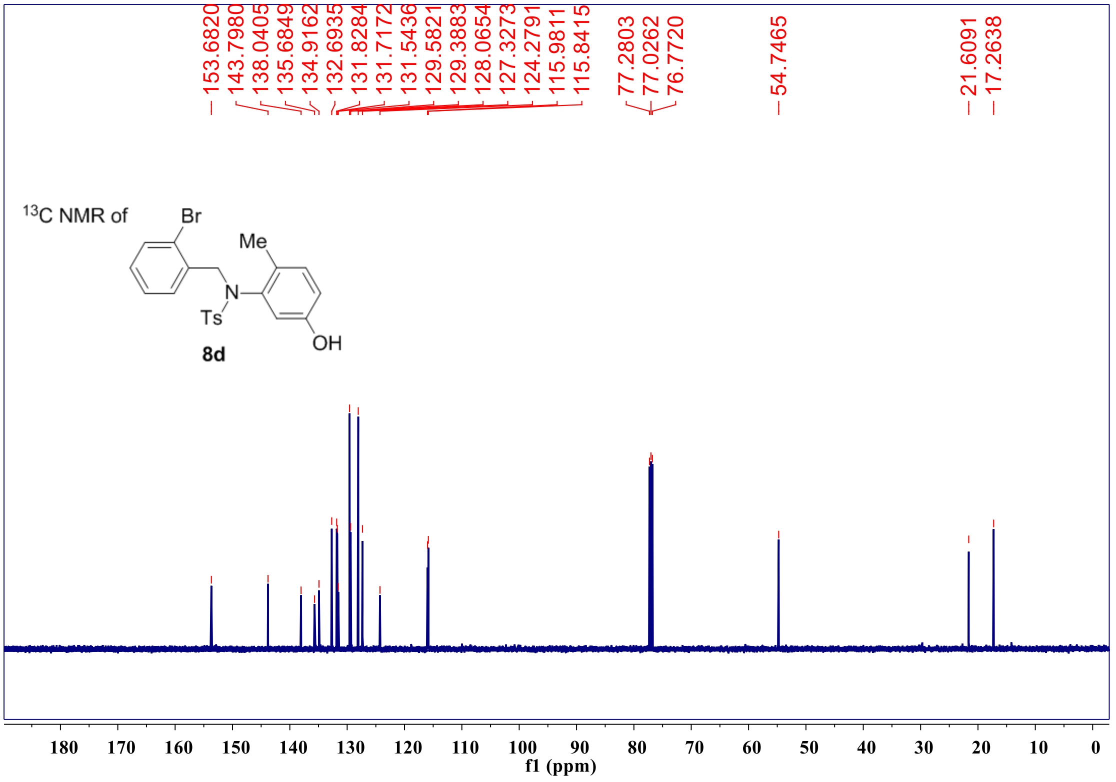

153.6820
143.7980
138.0405
135.6849
134.9162
132.6935
131.8284
131.7172
131.5436
129.5821
129.3883
128.0654
127.3273
124.2791
115.9811
115.8415
77.2803
77.0262
76.7720
54.7465
21.6091
17.2638
^{13}C NMR of
Br
Me
N
Ts
OH
8d
f1 (ppm)

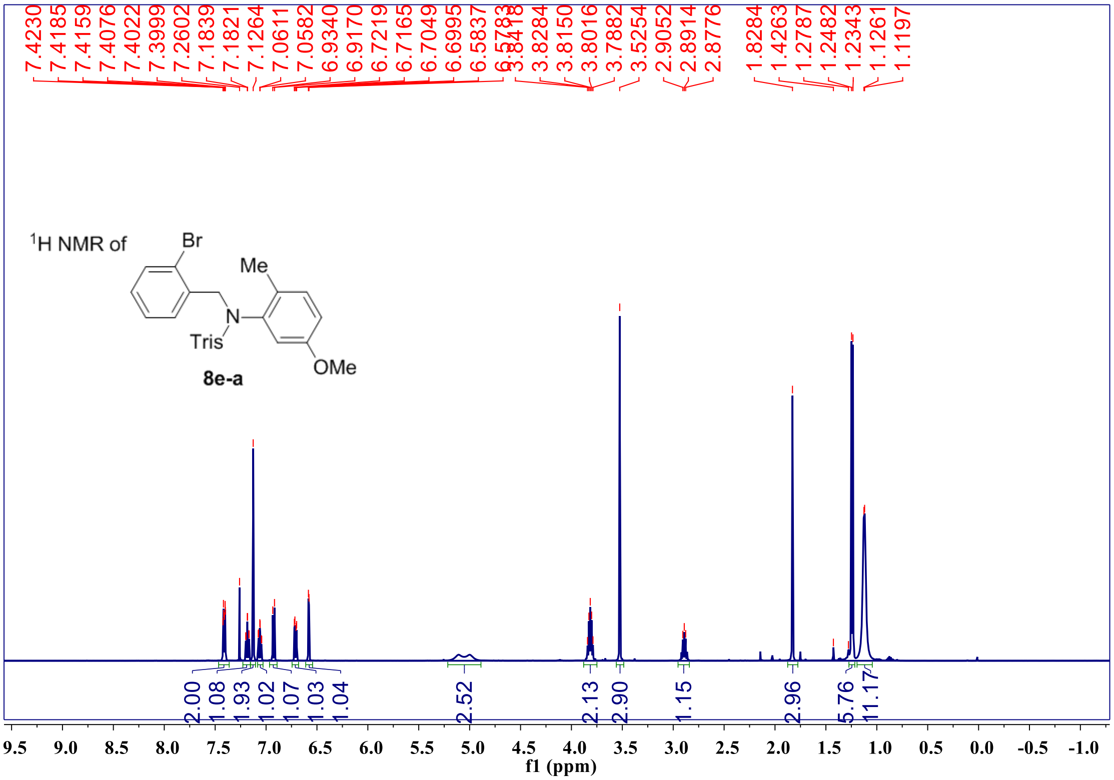

7.4230
7.4185
7.4159
7.4076
7.4022
7.3999
7.2602
7.1839
7.1821
7.1264
7.0611
7.0582
6.9340
6.9170
6.7219
6.7165
6.7049
6.6995
6.5837
6.5783
3.8418
3.8284
3.8150
3.8016
3.7882
3.5254
2.9052
2.8914
2.8776
1.8284
1.4263
1.2787
1.2482
1.2343
1.1261
1.1197
¹H NMR of
Br
Me
N
Tris
OMe
8e-a
2.00
1.08
1.93
1.02
1.07
1.03
1.04
2.52
2.13
2.90
1.15
2.96
5.76
11.17
9.5 9.0 8.5 8.0 7.5 7.0 6.5 6.0 5.5 5.0 4.5 4.0 3.5 3.0 2.5 2.0 1.5 1.0 0.5 0.0 -0.5 -1.0
f1 (ppm)

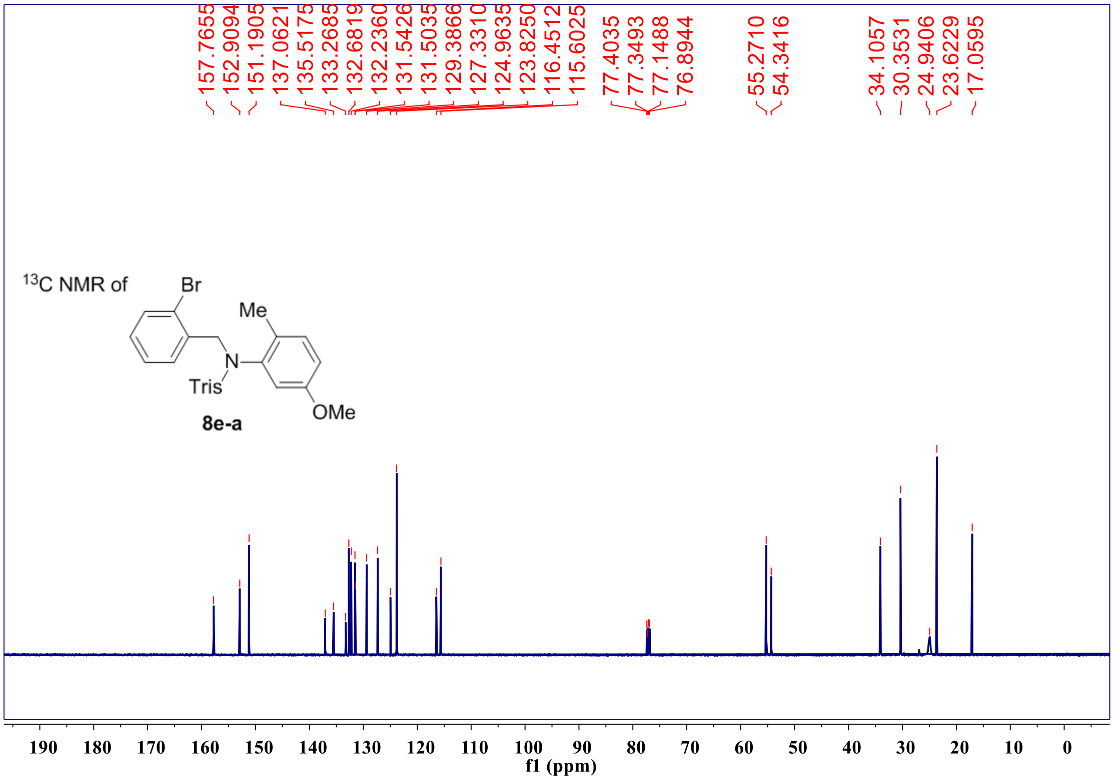

157.7655
152.9094
151.1905
137.0621
135.5175
133.2685
132.6819
132.2360
131.5426
131.5035
129.3866
127.3310
124.9635
123.8250
116.4512
115.6025
77.4035
77.3493
77.1488
76.8944
55.2710
54.3416
34.1057
30.3531
24.9406
23.6229
17.0595
¹³C NMR of
Br
Me
N
Tris
OMe
8e-a
190 180 170 160 150 140 130 120 110 100 90 80 70 60 50 40 30 20 10 0
f1 (ppm)

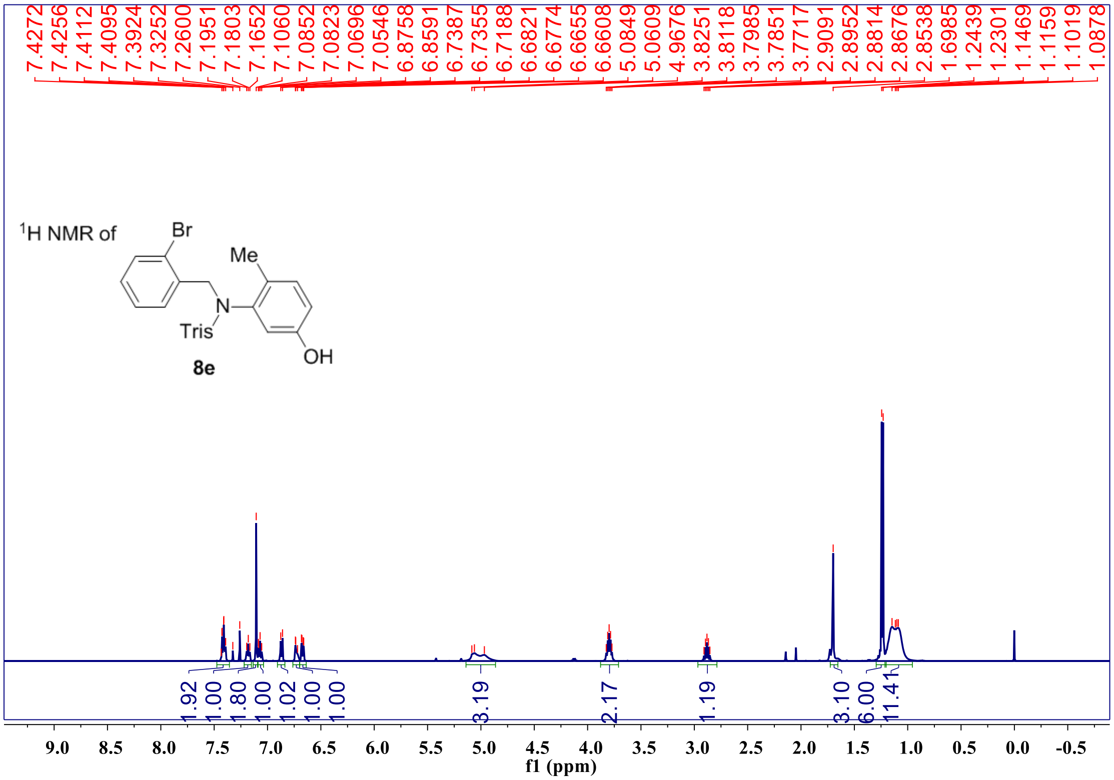
7.4272 7.4256 7.4112 7.4095 7.3924 7.3252 7.2600 7.1951 7.1803 7.1652 7.1060 7.0852 7.0823 7.0696 7.0546 6.8758 6.8591 6.7387 6.7355 6.7188 6.6821 6.6774 6.6655 6.6608 5.0849 5.0609 4.9676 3.8251 3.8118 3.7985 3.7851 3.7717 2.9091 2.8952 2.8814 2.8676 2.8538 1.6985 1.2439 1.2301 1.1469 1.1159 1.1019 1.0878
1H NMR of
Br
Me
N
Tris
OH
8e
1.92 1.00 1.80 1.00 1.02 1.00 1.00 3.19 2.17 1.19 3.10 6.00 11.41
f1 (ppm)

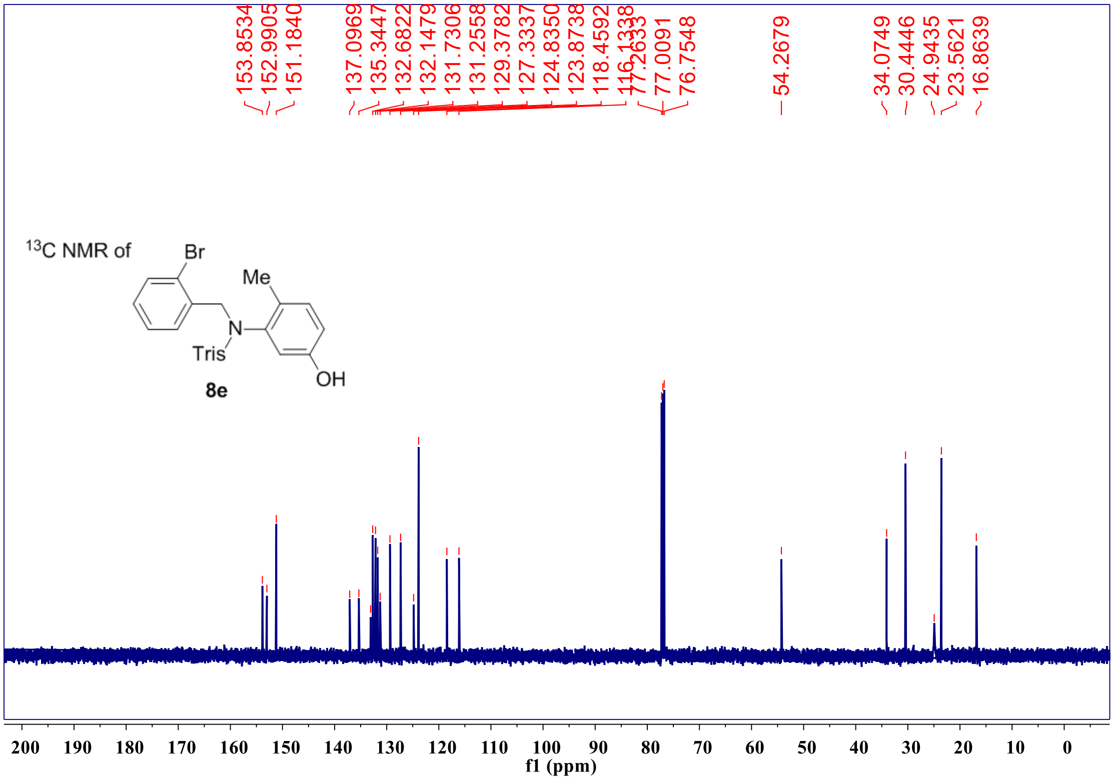
153.8534 152.9905 151.1840 137.0969 135.3447 132.6822 132.1479 131.7306 131.2558 129.3782 127.3337 124.8350 123.8738 118.4592 116.1338 77.2633 77.0091 76.7548 54.2679 34.0749 30.4446 24.9435 23.5621 16.8639
13C NMR of
Br
Me
N
Tris
OH
8e
f1 (ppm)

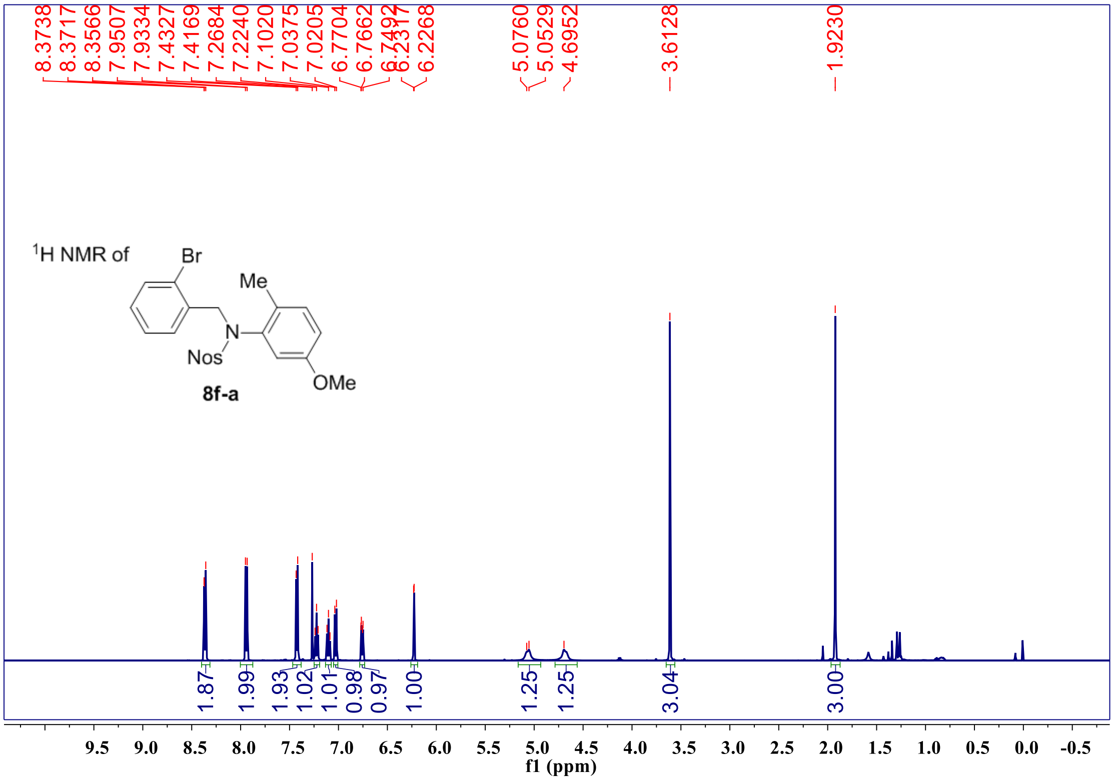
8.3738
8.3717
8.3566
7.9507
7.9334
7.4327
7.4169
7.2684
7.2240
7.1020
7.0375
7.0205
6.7704
6.7662
6.7492
6.2317
6.2268
5.0760
5.0529
4.6952
3.6128
1.9230
1H NMR of
Br
Me
N
Nos
OMe
8f-a
1.87
1.99
1.93
1.02
1.01
0.98
0.97
1.00
1.25
1.25
3.04
3.00
f1 (ppm)

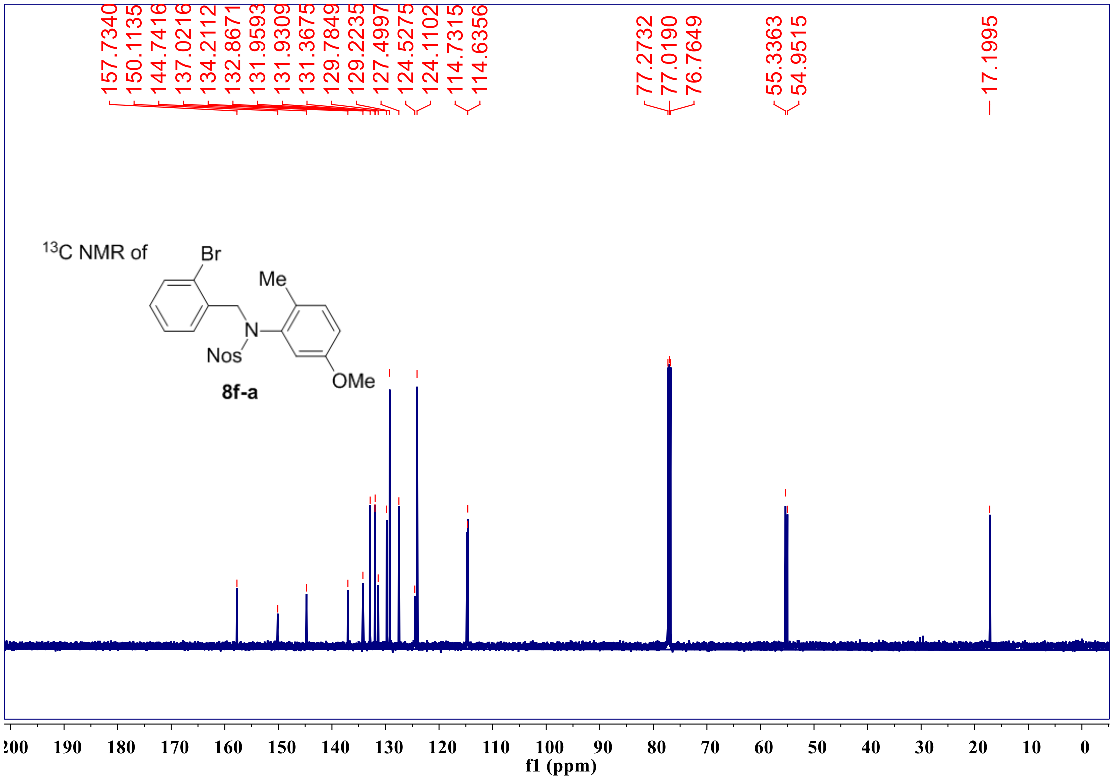
157.7340
150.1135
144.7416
137.0216
134.2112
132.8671
131.9593
131.9309
131.3675
129.7849
129.2235
127.4997
124.5275
124.1102
114.7315
114.6356
77.2732
77.0190
76.7649
55.3363
54.9515
17.1995
13C NMR of
Br
Me
N
Nos
OMe
8f-a
f1 (ppm)

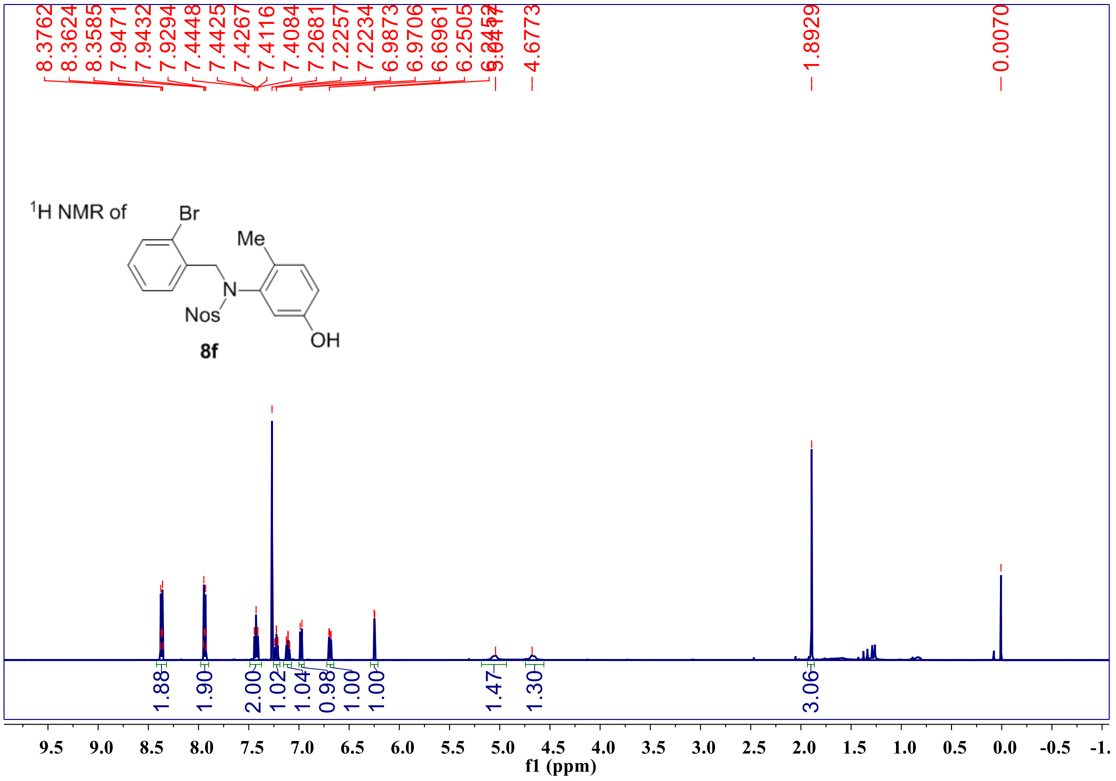

^{1}H NMR of
Br
Me
N
Nos
OH
8f
f1 (ppm)

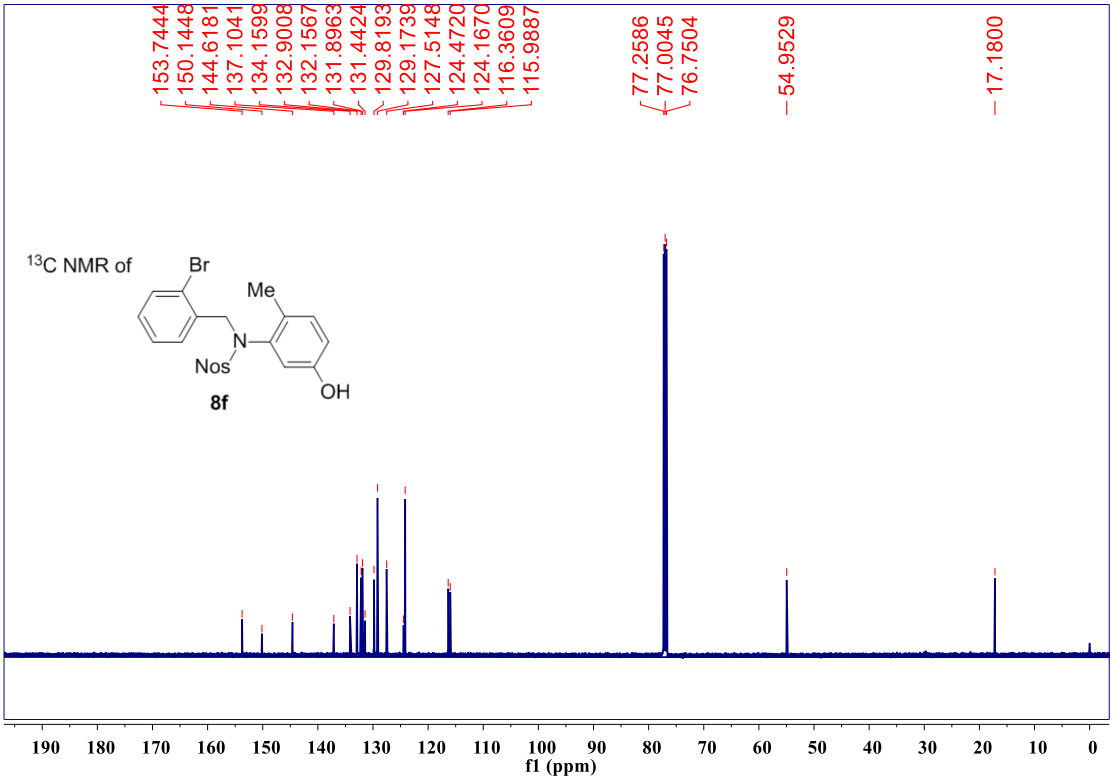

^{13}C NMR of
Br
Me
N
Nos
OH
8f
f1 (ppm)

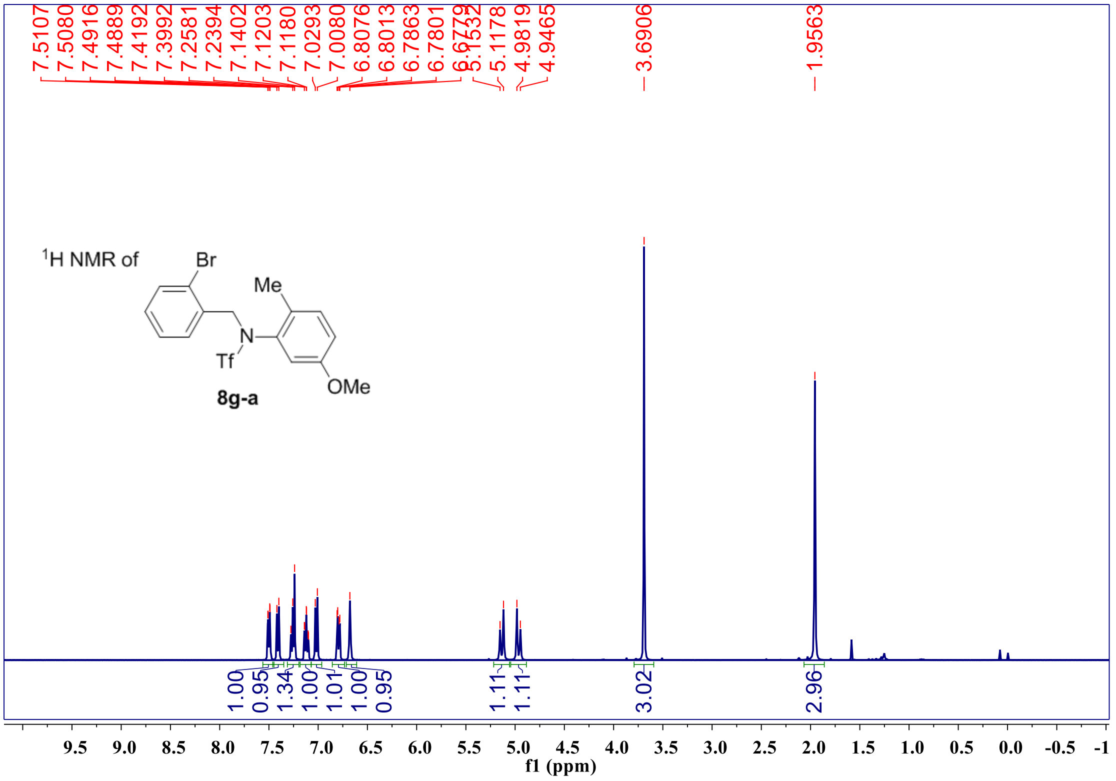

^{1}H NMR of
Br
Me
N
Tf
OMe
8g-a
7.5107
7.5080
7.4916
7.4889
7.4192
7.3992
7.2581
7.2394
7.1402
7.1203
7.1180
7.0293
7.0080
6.8076
6.8013
6.7863
6.7801
6.6779
5.1532
5.1178
4.9819
4.9465
3.6906
1.9563
1.00
0.95
1.34
1.00
1.01
1.00
0.95
1.11
1.11
3.02
2.96
f1 (ppm)

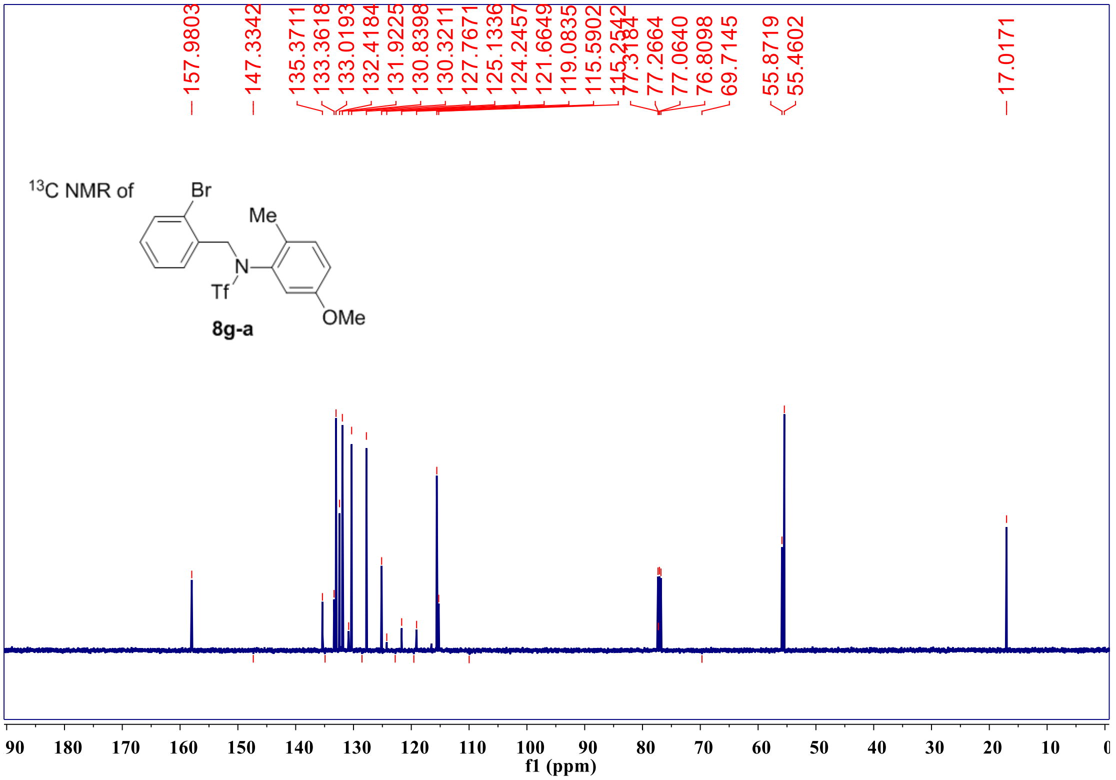

^{13}C NMR of
Br
Me
N
Tf
OMe
8g-a
157.9803
147.3342
135.3711
133.3618
133.0193
132.4184
131.9225
130.8398
130.3211
127.7671
125.1336
124.2457
121.6649
119.0835
115.5902
115.2542
77.3184
77.2664
77.0640
76.8098
69.7145
55.8719
55.4602
17.0171
f1 (ppm)

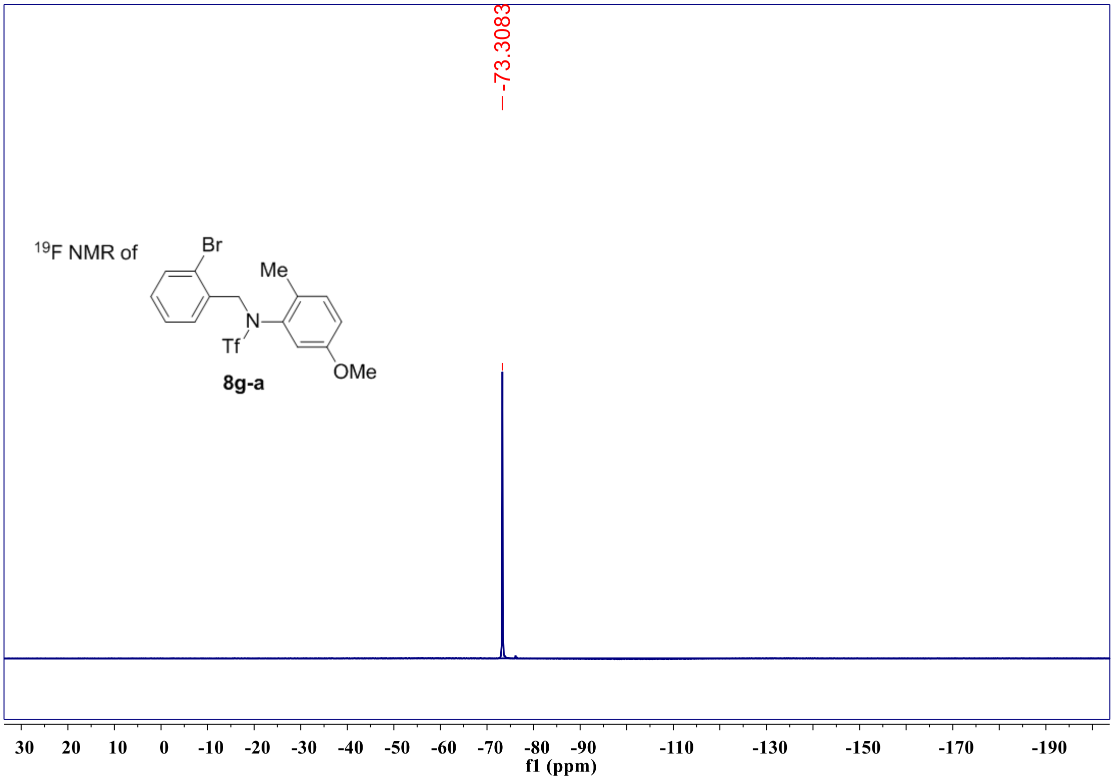
-73.3083
19F NMR of
Br
Me
N
Tf
OMe
8g-a
30 20 10 0 -10 -20 -30 -40 -50 -60 -70 -80 -90 -110 -130 -150 -170 -190
f1 (ppm)

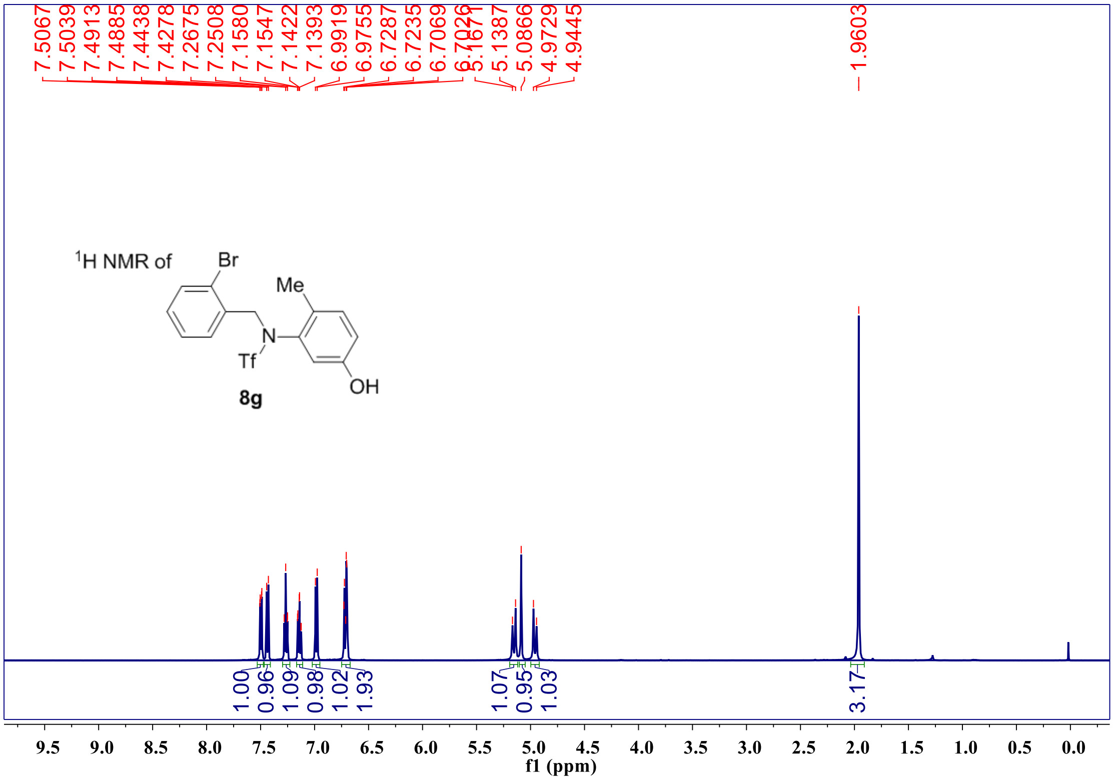
7.5067
7.5039
7.4913
7.4885
7.4438
7.4278
7.2675
7.2508
7.1580
7.1547
7.1422
7.1393
6.9919
6.9755
6.7287
6.7235
6.7069
5.1387
5.0866
4.9729
4.9445
1.9603
1H NMR of
Br
Me
N
Tf
OH
8g
1.00
0.96
1.09
0.98
1.02
1.93
1.07
0.95
1.03
3.17
9.5 9.0 8.5 8.0 7.5 7.0 6.5 6.0 5.5 5.0 4.5 4.0 3.5 3.0 2.5 2.0 1.5 1.0 0.5 0.0
f1 (ppm)

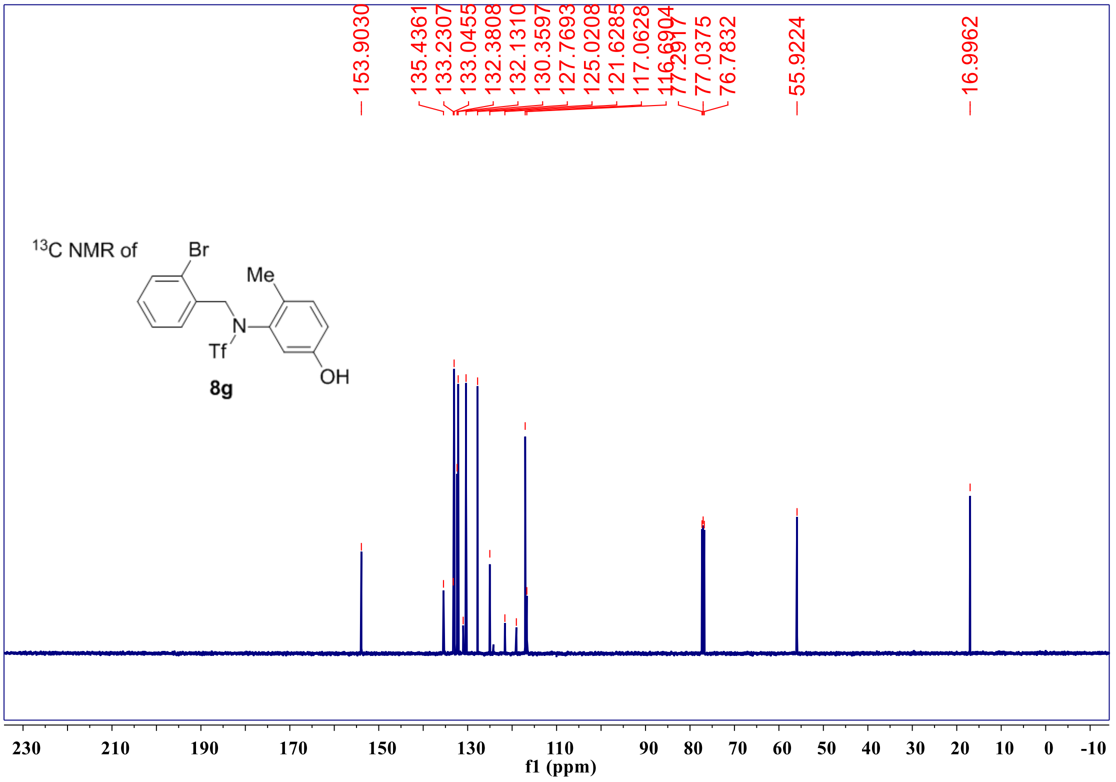

153.9030
135.4361
133.2307
133.0455
132.3808
132.1310
130.3597
127.7693
125.0208
121.6285
117.0628
116.6904
77.2917
77.0375
76.7832
55.9224
16.9962
^{13}C NMR of
Br
Me
N
Tf
OH
8g
f1 (ppm)

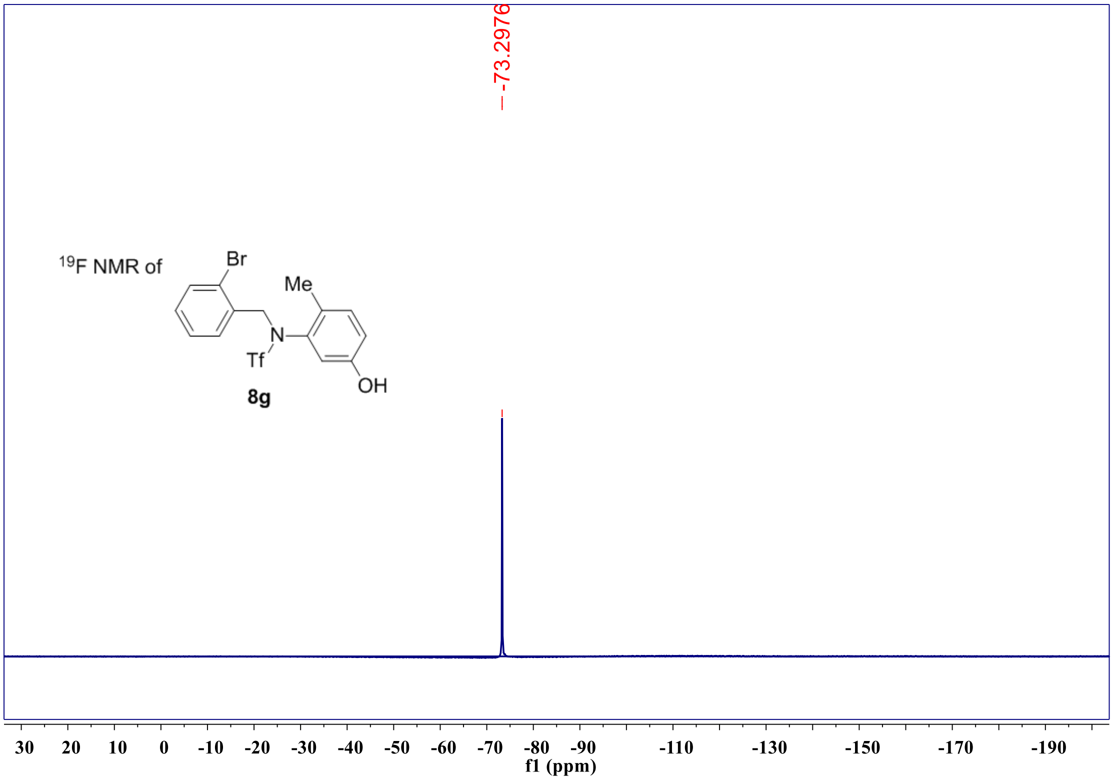

-73.2976
^{19}F NMR of
Br
Me
N
Tf
OH
8g
f1 (ppm)

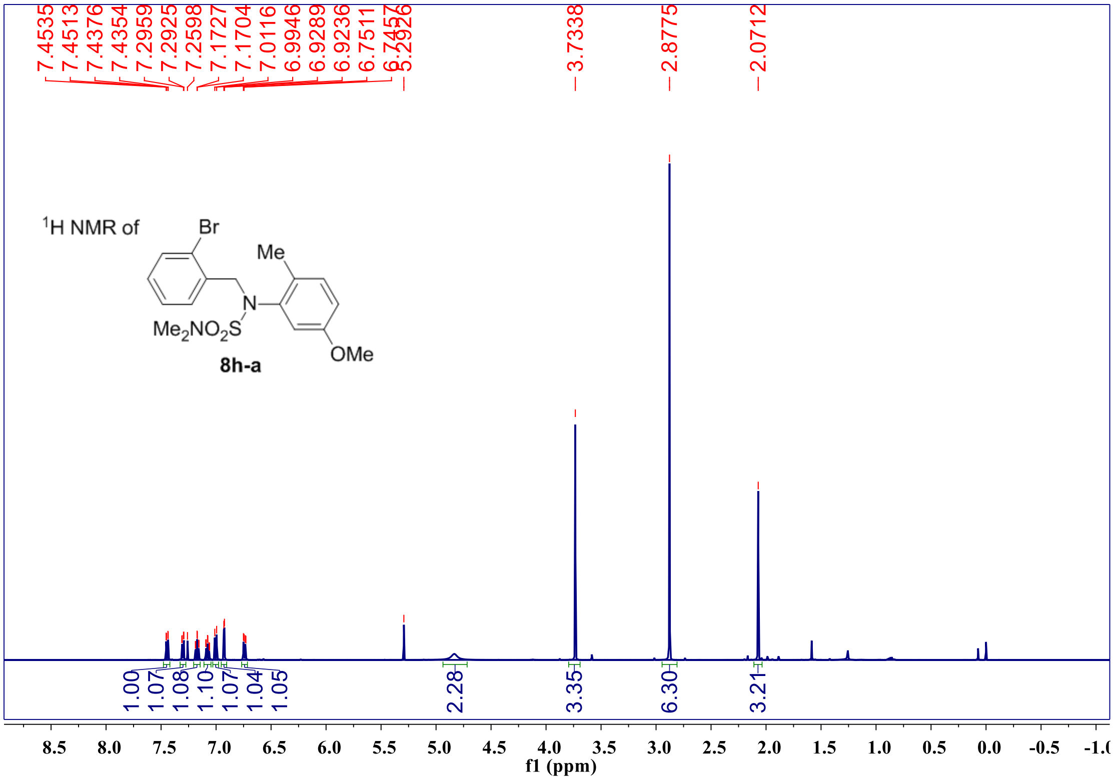
7.4535
7.4513
7.4376
7.4354
7.2959
7.2925
7.2598
7.1727
7.1704
7.0116
6.9946
6.9289
6.9236
6.7511
6.7457
5.2926
3.7338
2.8775
2.0712
1H NMR of
Br
Me
N
Me2NO2S
OMe
8h-a
1.00
1.07
1.08
1.10
1.07
1.04
1.05
2.28
3.35
6.30
3.21
f1 (ppm)

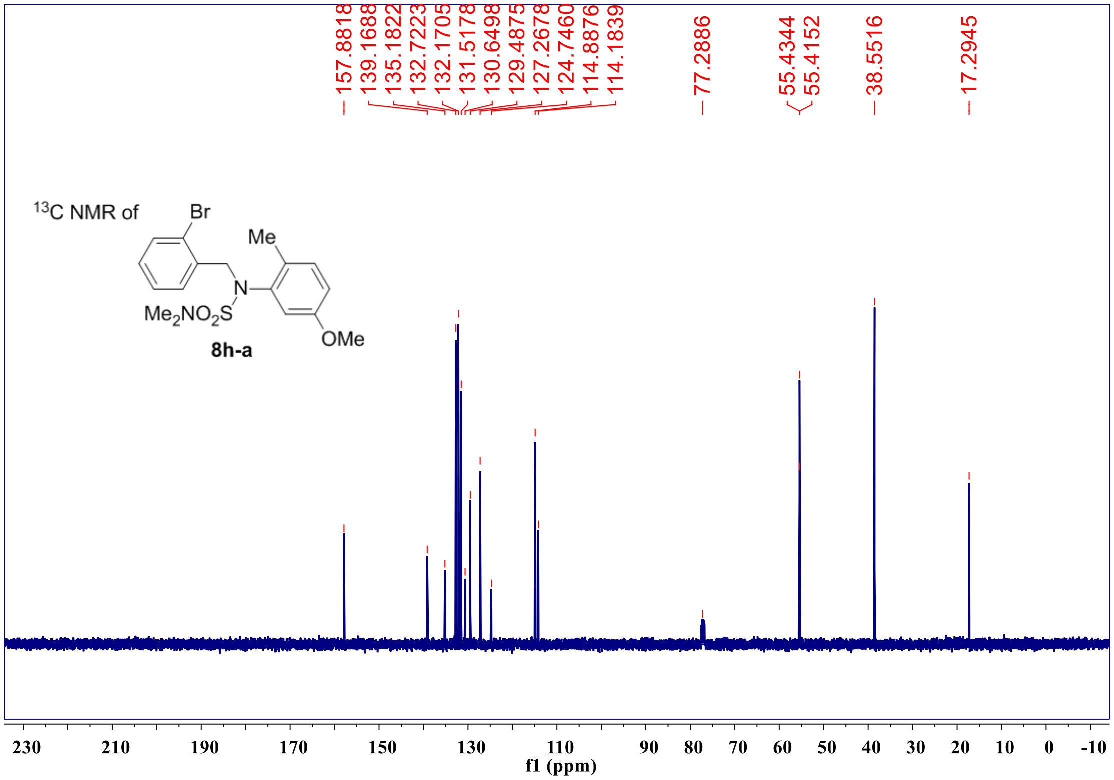
157.8818
139.1688
135.1822
132.7223
132.1705
131.5178
130.6498
129.4875
127.2678
124.7460
114.8876
114.1839
77.2886
55.4344
55.4152
38.5516
17.2945
13C NMR of
Br
Me
N
Me2NO2S
OMe
8h-a
f1 (ppm)

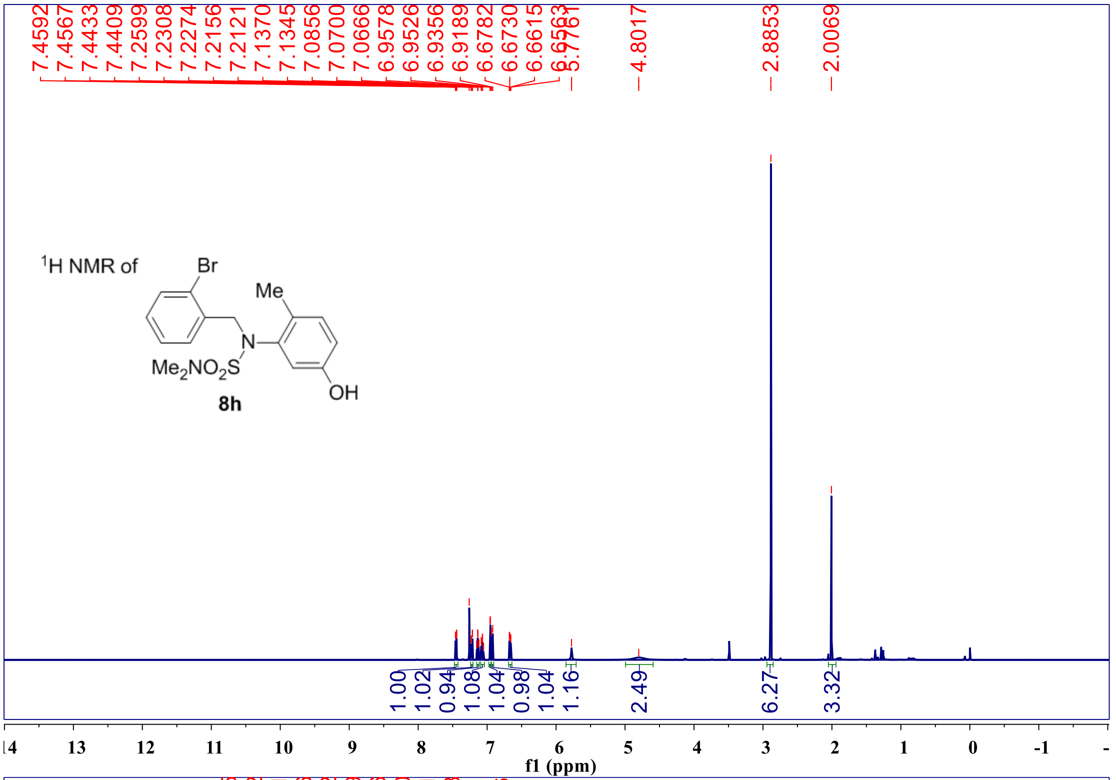
7.4592
7.4567
7.4433
7.4409
7.2599
7.2308
7.2274
7.2156
7.2121
7.1370
7.1345
7.0856
7.0700
7.0666
6.9578
6.9526
6.9356
6.9189
6.6782
6.6730
6.6615
6.6563
5.7761
4.8017
2.8853
2.0069
^{1}H NMR of
Br
Me
N
Me_2NO_2S
OH
8h
1.00
1.02
0.94
1.08
1.04
0.98
1.04
1.16
2.49
6.27
3.32
f1 (ppm)

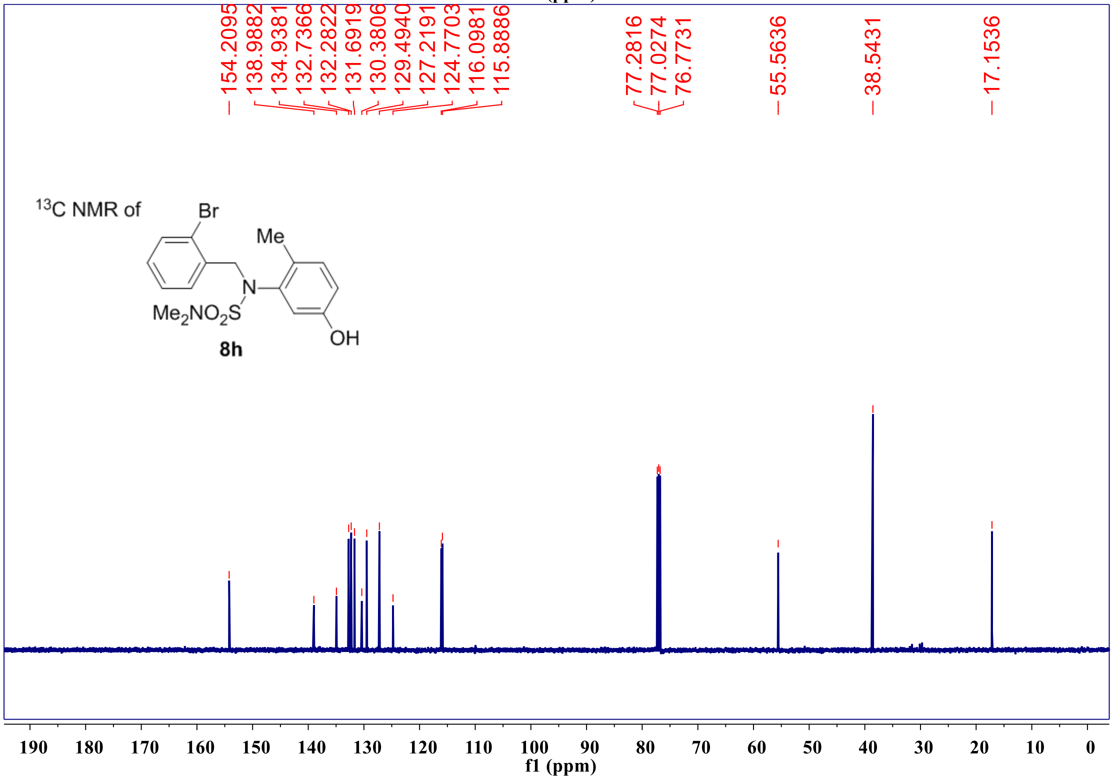
154.2095
138.9882
134.9381
132.7366
132.2822
131.6919
130.3806
129.4940
127.2191
124.7703
116.0981
115.8886
77.2816
77.0274
76.7731
55.5636
38.5431
17.1536
^{13}C NMR of
Br
Me
N
Me_2NO_2S
OH
8h
f1 (ppm)

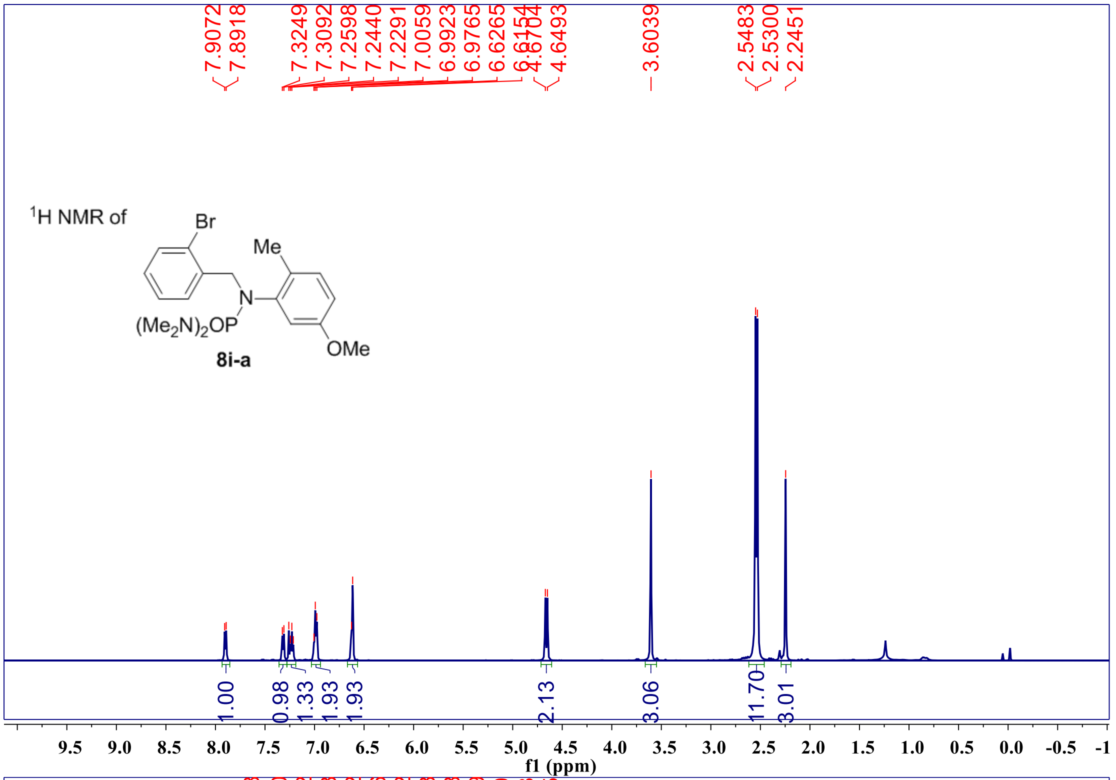

7.9072
7.8918
7.3249
7.3092
7.2598
7.2440
7.2291
7.0059
6.9923
6.9765
6.6265
6.6154
4.6704
4.6493
3.6039
2.5483
2.5300
2.2451
^{1}H NMR of
Br
Me
N
$(Me_2N)_2OP$
OMe
8i-a
1.00
0.98
1.33
1.93
1.93
2.13
3.06
11.70
3.01
f1 (ppm)

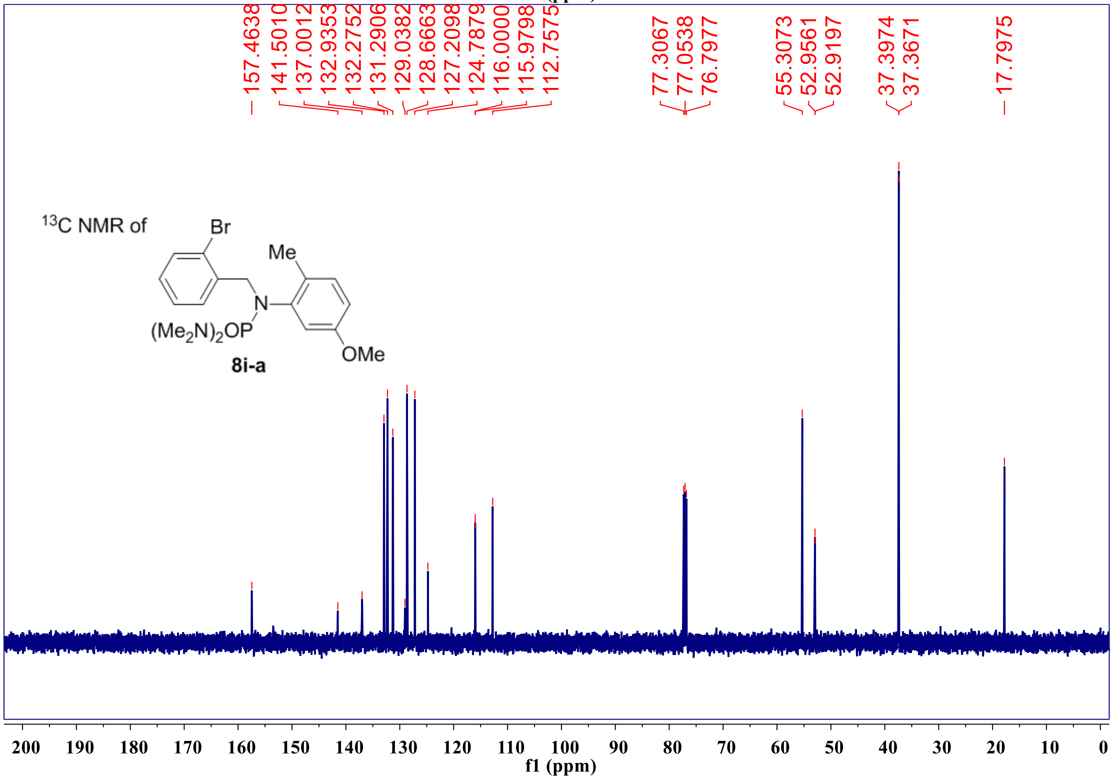

157.4638
141.5010
137.0012
132.9353
132.2752
131.2906
129.0382
128.6663
127.2098
124.7879
116.0000
115.9798
112.7575
77.3067
77.0538
76.7977
55.3073
52.9561
52.9197
37.3974
37.3671
17.7975
^{13}C NMR of
Br
Me
N
$(Me_2N)_2OP$
OMe
8i-a
f1 (ppm)

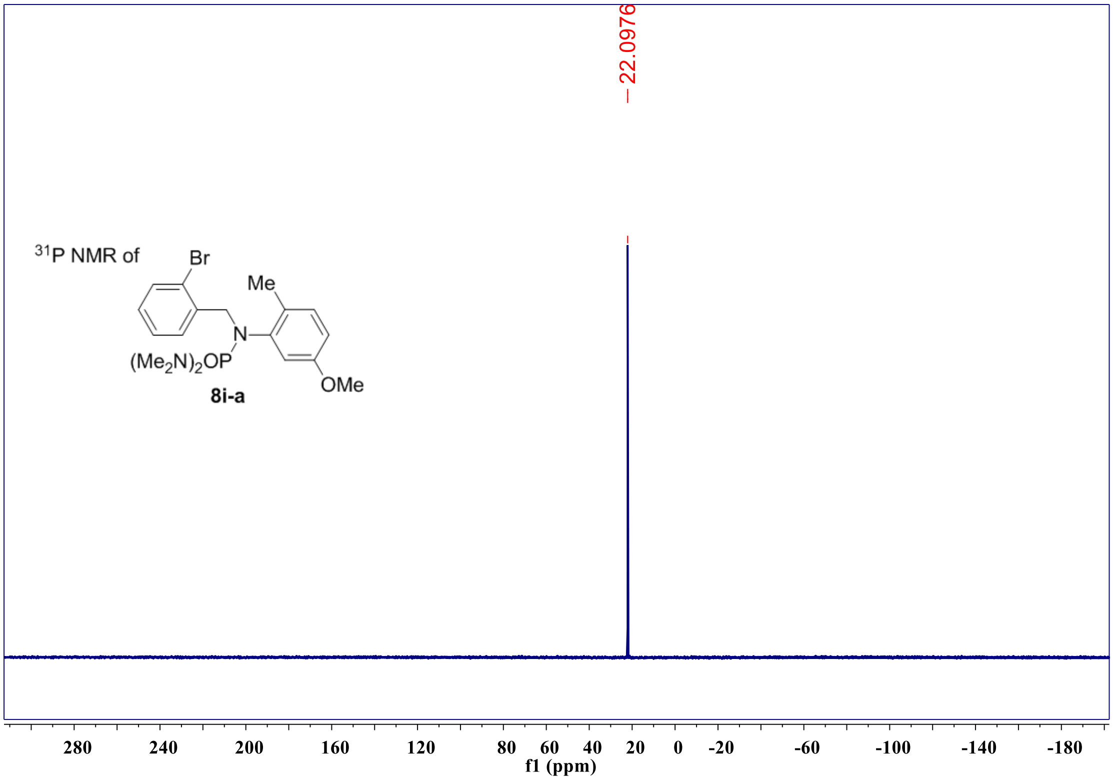
22.0976
31P NMR of
Br
Me
N
(Me2N)2OP
8i-a
OMe
280 240 200 160 120 80 60 40 20 0 -20 -60 -100 -140 -180
f1 (ppm)

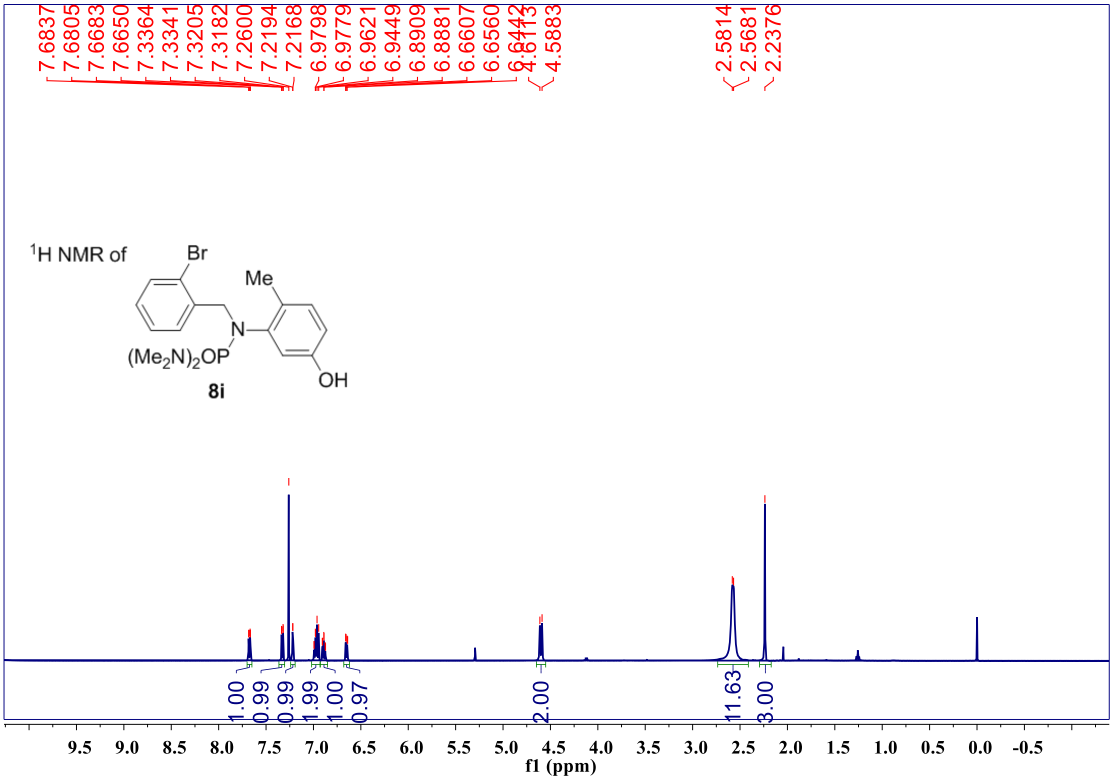
7.6837
7.6805
7.6683
7.6650
7.3364
7.3341
7.3205
7.3182
7.2600
7.2194
7.2168
6.9798
6.9779
6.9621
6.9449
6.8909
6.8881
6.6607
6.6560
6.6442
4.6113
4.5883
2.5814
2.5681
2.2376
1H NMR of
Br
Me
N
(Me2N)2OP
8i
OH
1.00
0.99
0.99
1.99
1.00
0.97
2.00
11.63
3.00
9.5 9.0 8.5 8.0 7.5 7.0 6.5 6.0 5.5 5.0 4.5 4.0 3.5 3.0 2.5 2.0 1.5 1.0 0.5 0.0 -0.5
f1 (ppm)

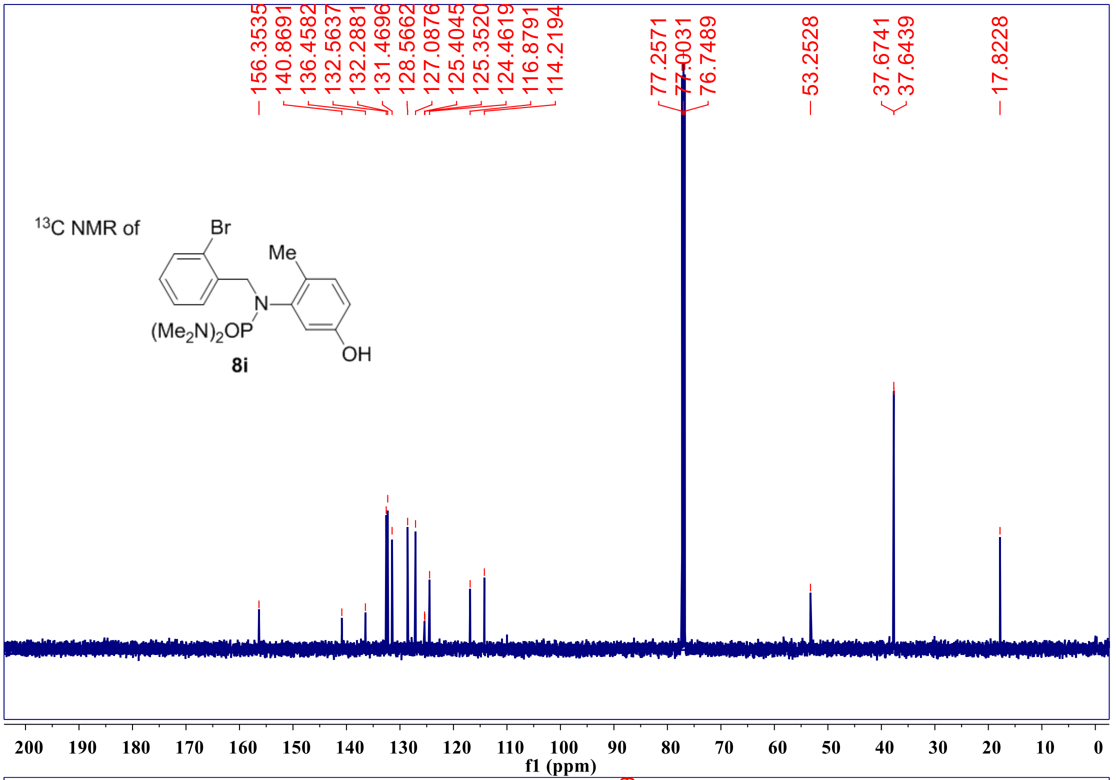
156.3535
140.8691
136.4582
132.5637
132.2881
131.4696
128.5662
127.0876
125.4045
125.3520
124.4619
116.8791
114.2194
77.2571
77.0031
76.7489
53.2528
37.6741
37.6439
17.8228
13C NMR of
Br
Me
N
(Me2N)2OP
OH
8i
f1 (ppm)

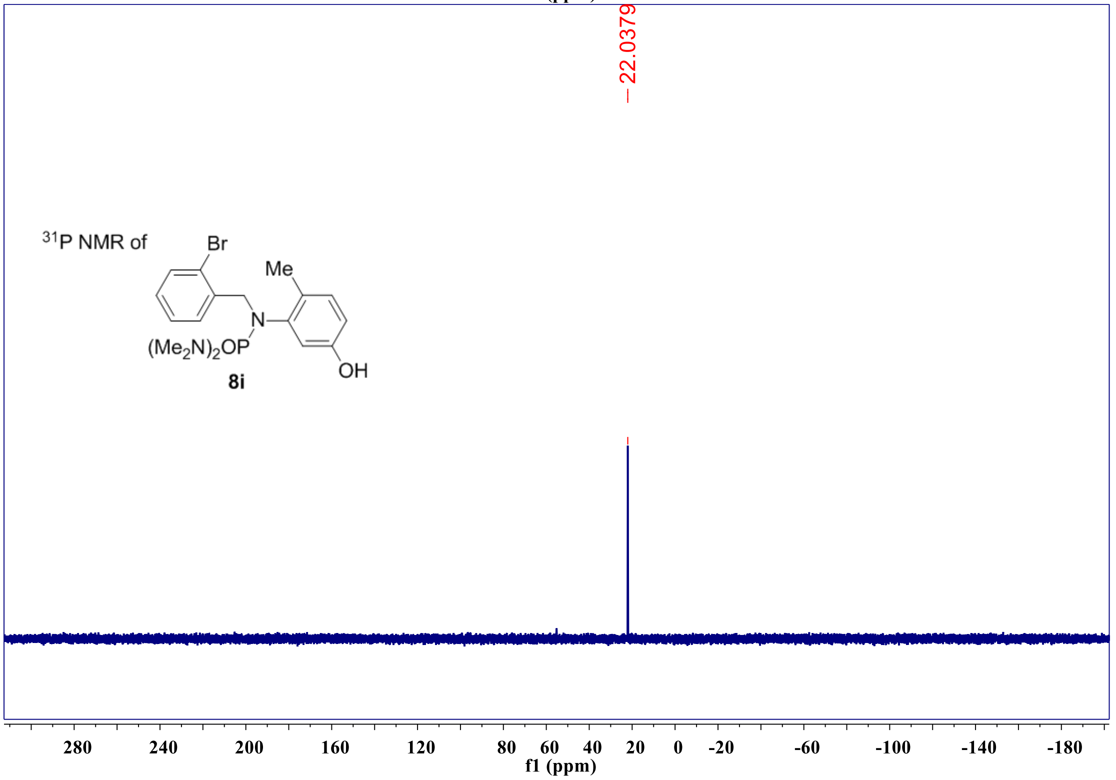
22.0379
31P NMR of
Br
Me
N
(Me2N)2OP
OH
8i
f1 (ppm)

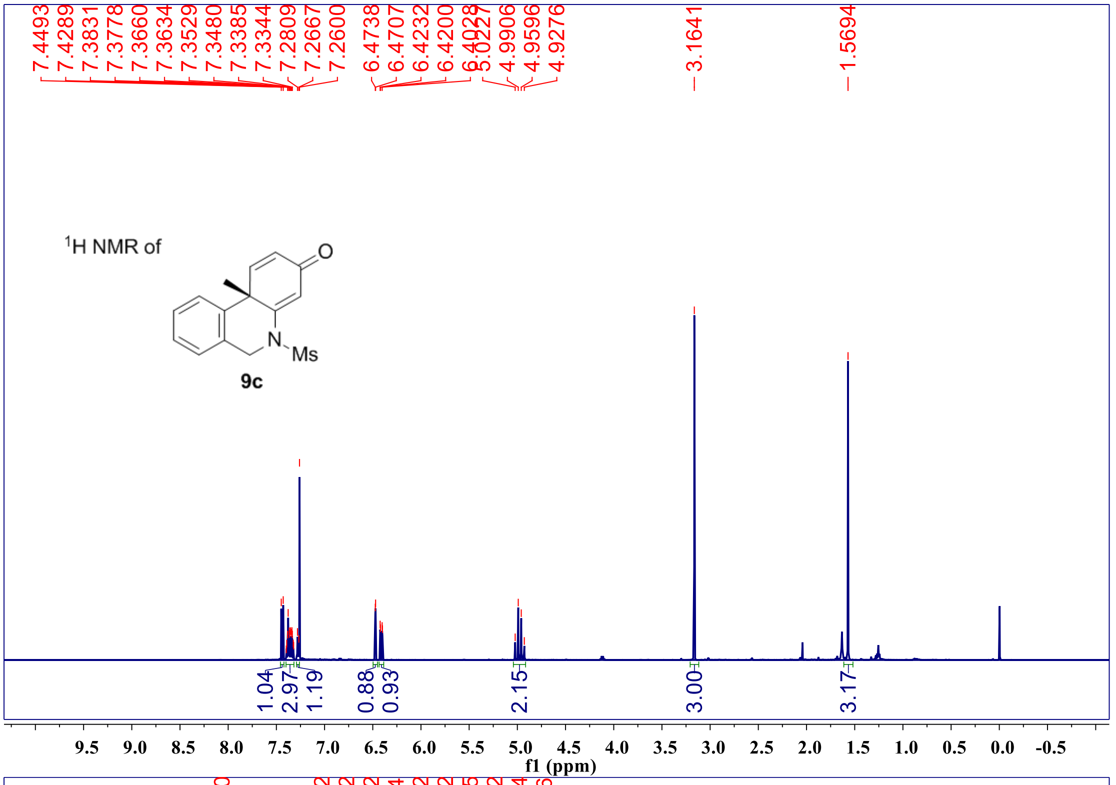

1H NMR of
9c
Ms
O
N
f1 (ppm)

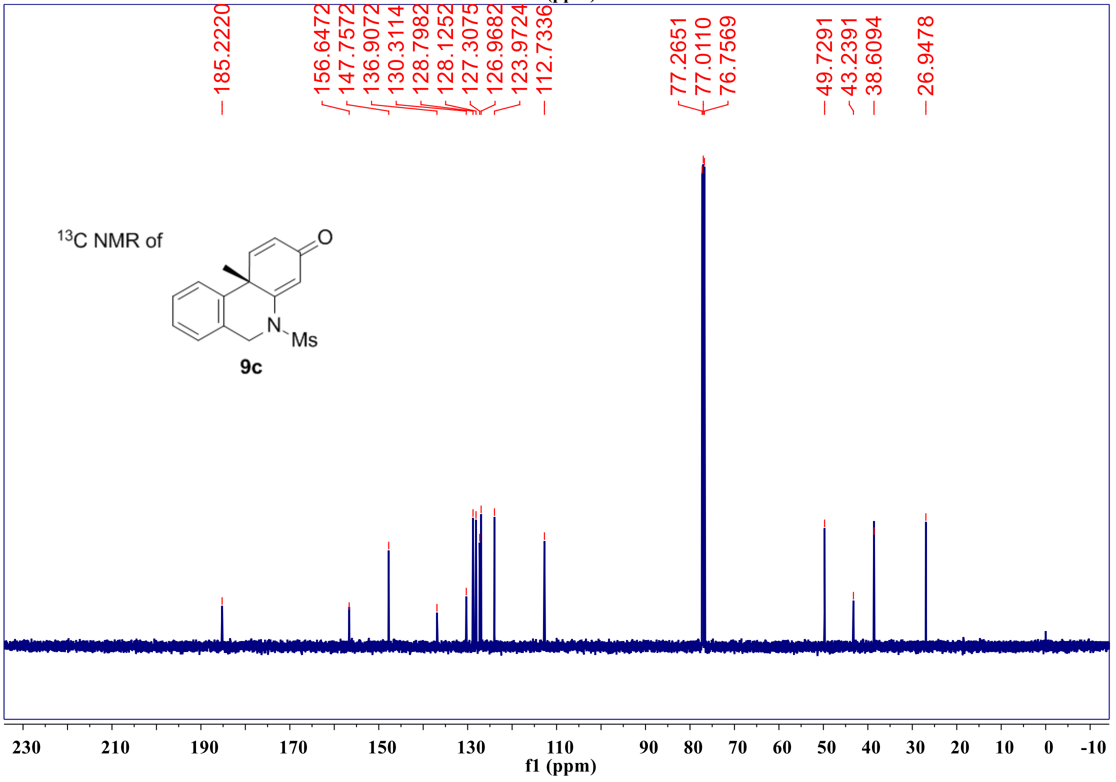

13C NMR of
9c
Ms
O
N
f1 (ppm)

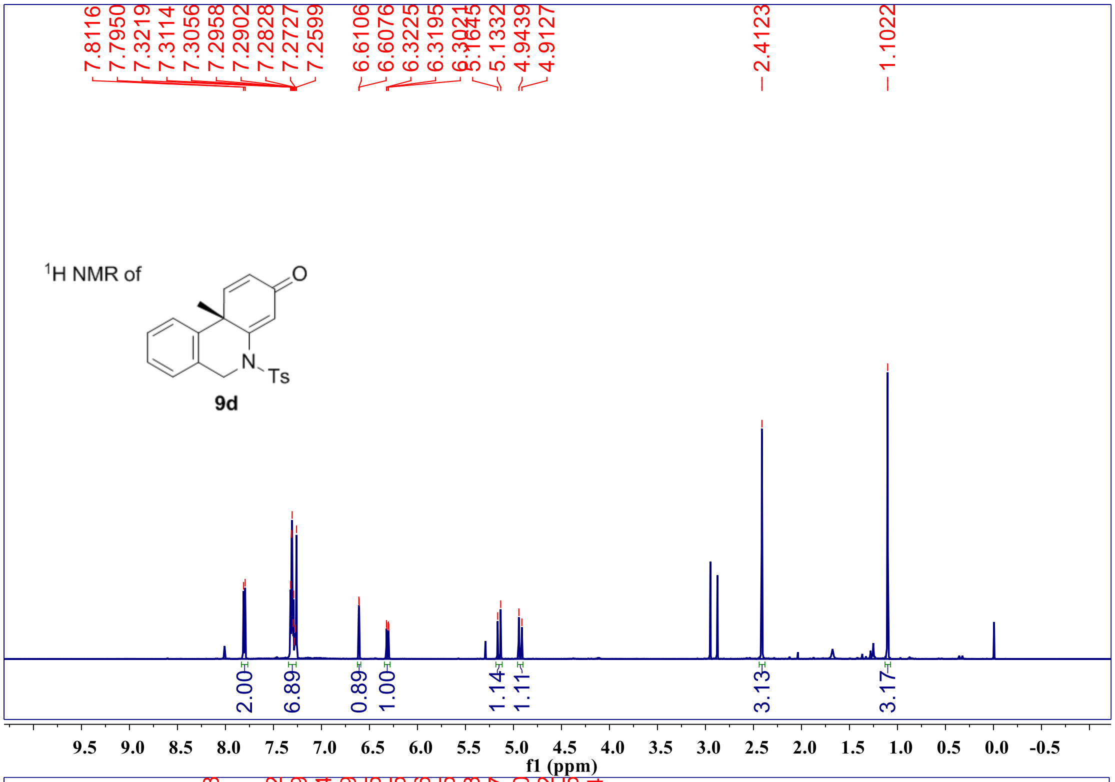

7.8116
7.7950
7.3219
7.3114
7.3056
7.2958
7.2902
7.2828
7.2727
7.2599
6.6106
6.6076
6.3225
6.3195
6.3021
5.1645
5.1332
4.9439
4.9127
2.4123
1.1022
^{1}H NMR of
O
N
Ts
9d
2.00
6.89
0.89
1.00
1.14
1.11
3.13
3.17
9.5 9.0 8.5 8.0 7.5 7.0 6.5 6.0 5.5 5.0 4.5 4.0 3.5 3.0 2.5 2.0 1.5 1.0 0.5 0.0 -0.5
f1 (ppm)

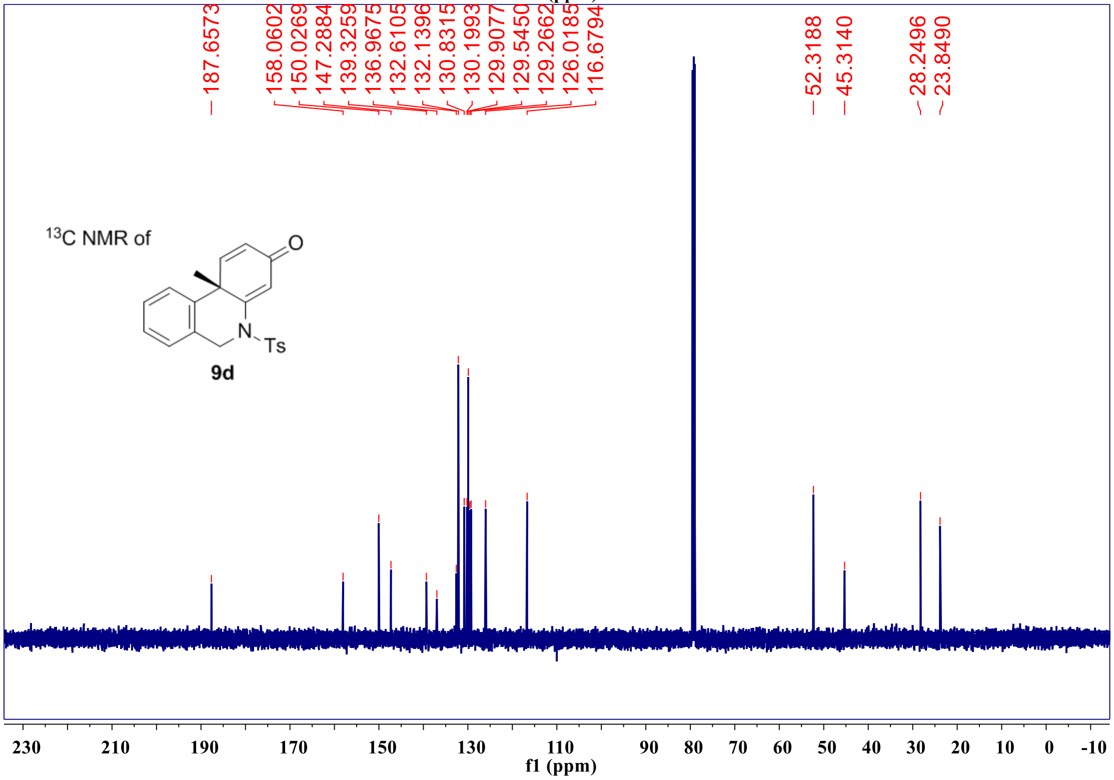

187.6573
158.0602
150.0269
147.2884
139.3259
136.9675
132.6105
132.1396
130.8315
130.1993
129.9077
129.5450
129.2662
126.0185
116.6794
52.3188
45.3140
28.2496
23.8490
^{13}C NMR of
O
N
Ts
9d
230 210 190 170 150 130 110 90 80 70 60 50 40 30 20 10 0 -10
f1 (ppm)

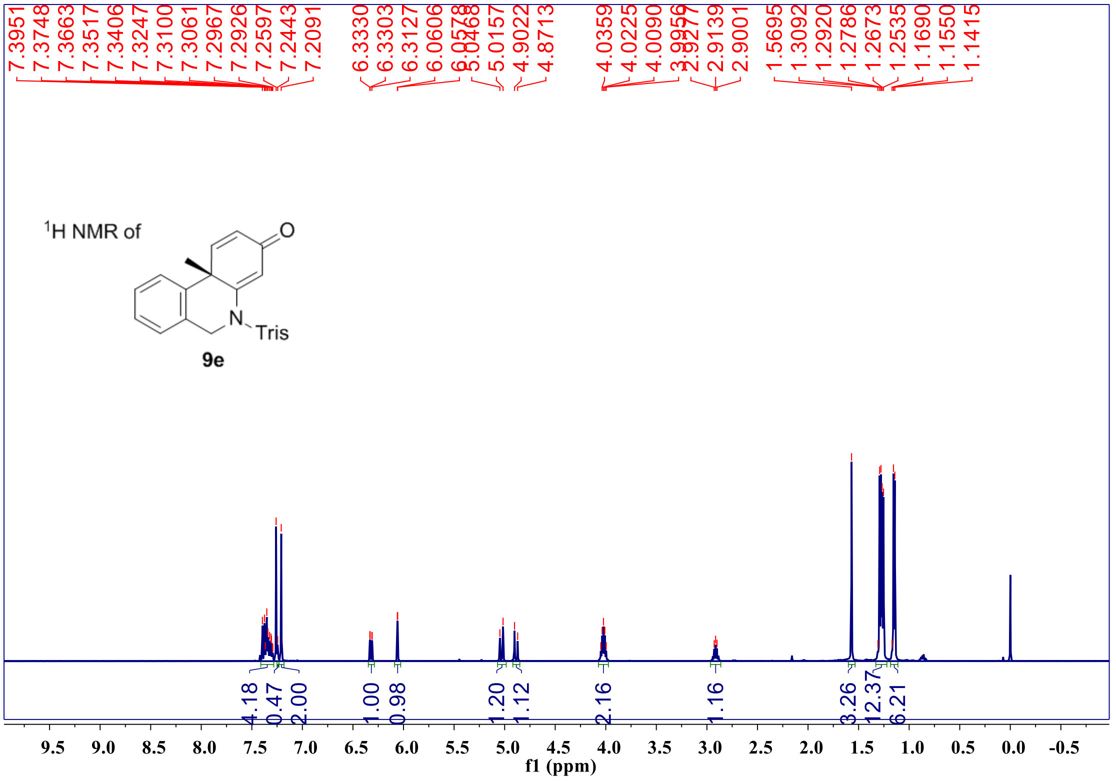

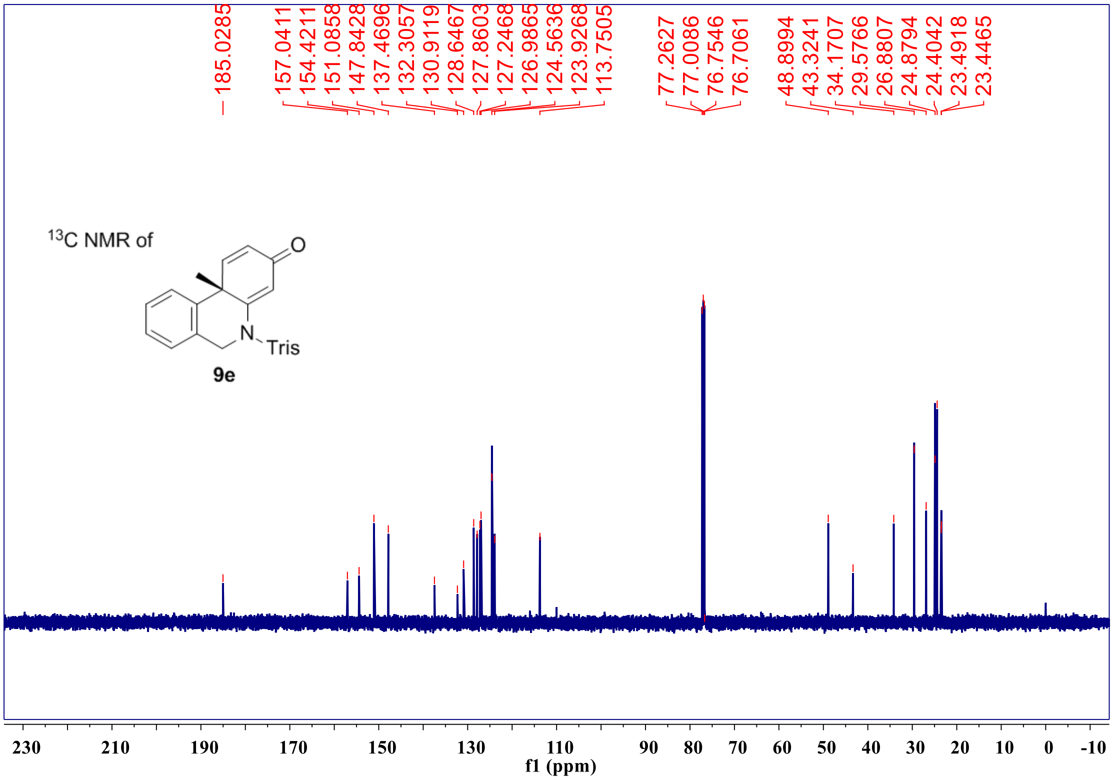

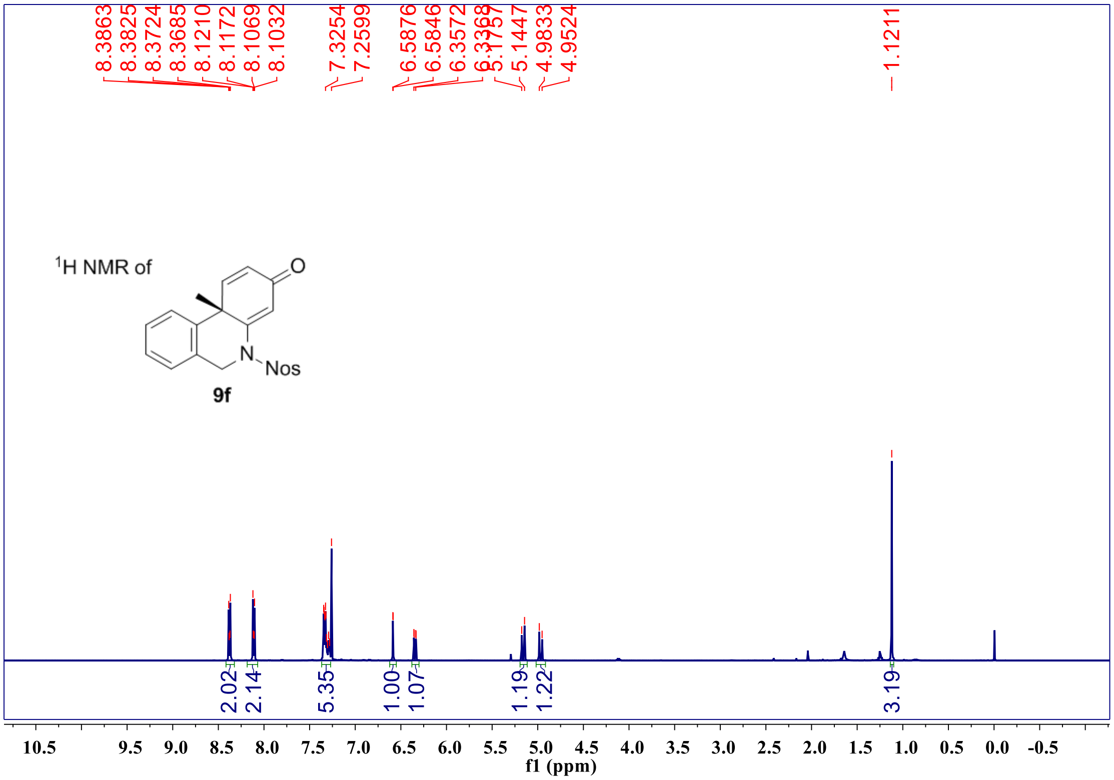

8.3863
8.3825
8.3724
8.3685
8.1210
8.1172
8.1069
8.1032
7.3254
7.2599
6.5876
6.5846
6.3572
6.3368
5.1757
5.1447
4.9833
4.9524
1.1211
1H NMR of
O
N
Nos
9f
2.02
2.14
5.35
1.00
1.07
1.19
1.22
3.19
10.5 9.5 9.0 8.5 8.0 7.5 7.0 6.5 6.0 5.5 5.0 4.5 4.0 3.5 3.0 2.5 2.0 1.5 1.0 0.5 0.0 -0.5
f1 (ppm)

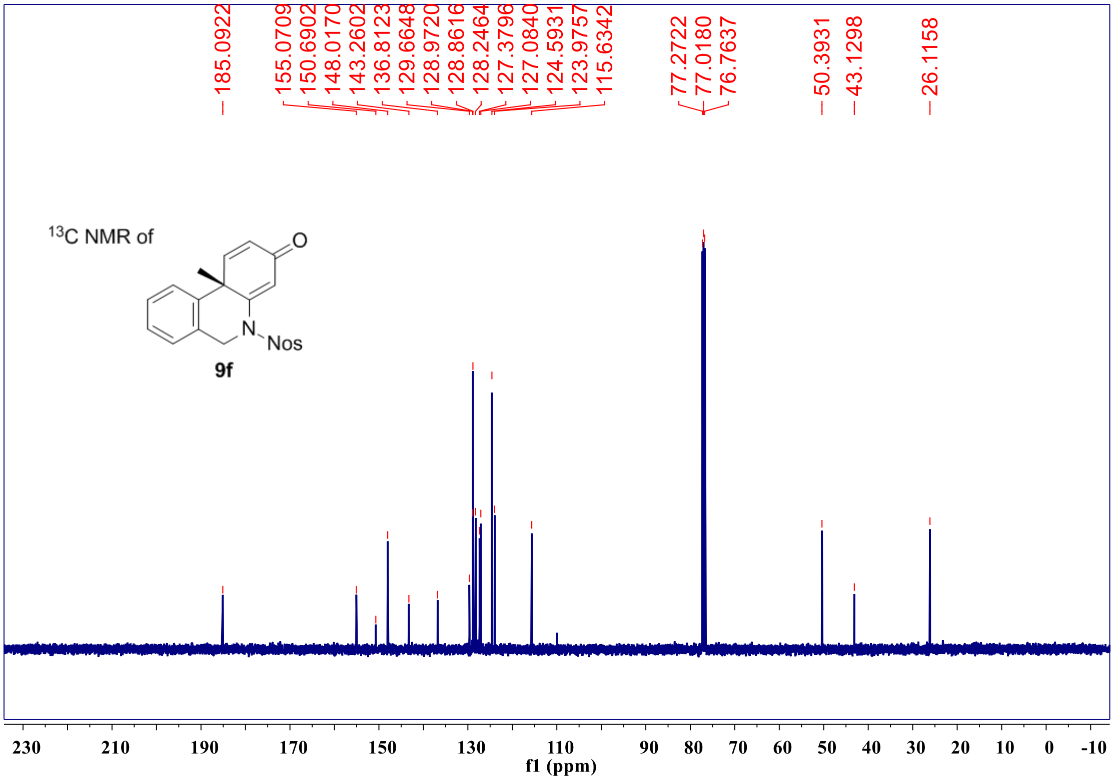

185.0922
155.0709
150.6902
148.0170
143.2602
136.8123
129.6648
128.9720
128.8616
128.2464
127.3796
127.0840
124.5931
123.9757
115.6342
77.2722
77.0180
76.7637
50.3931
43.1298
26.1158
13C NMR of
O
N
Nos
9f
230 210 190 170 150 130 110 90 80 70 60 50 40 30 20 10 0 -10
f1 (ppm)

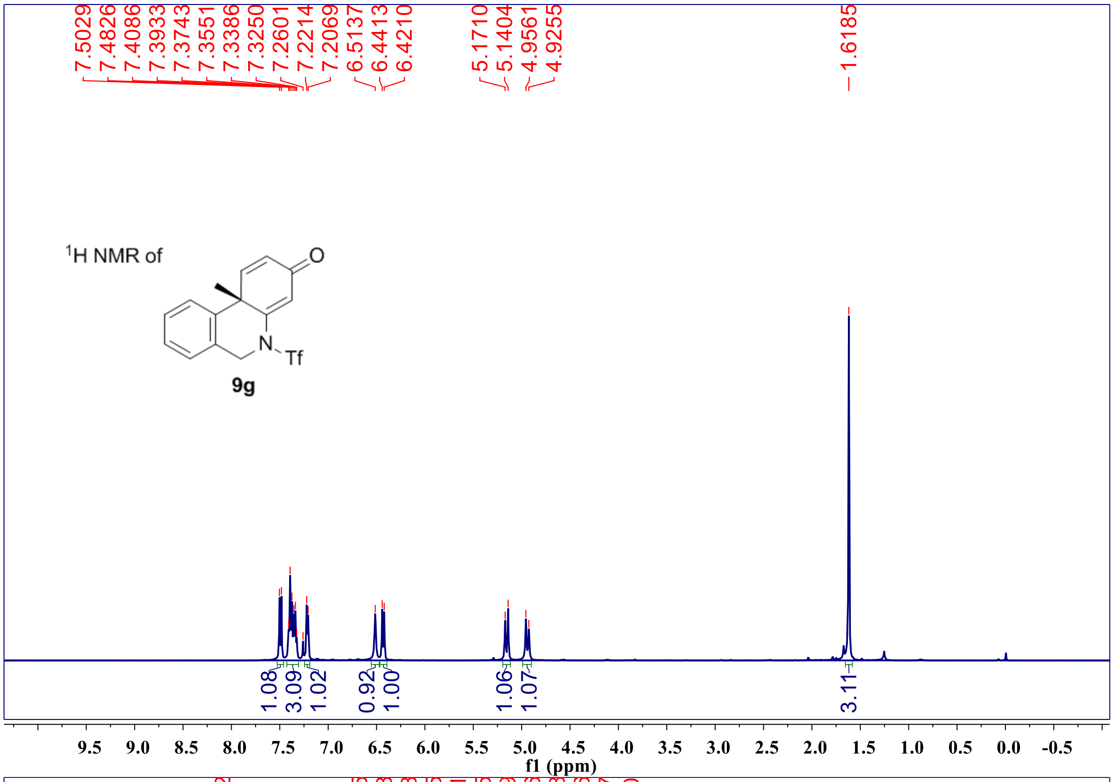
7.5029
7.4826
7.4086
7.3933
7.3743
7.3551
7.3386
7.3250
7.2601
7.2214
7.2069
6.5137
6.4413
6.4210
5.1710
5.1404
4.9561
4.9255
1.6185
1H NMR of
9g
1.08
3.09
1.02
0.92
1.00
1.06
1.07
3.11
f1 (ppm)

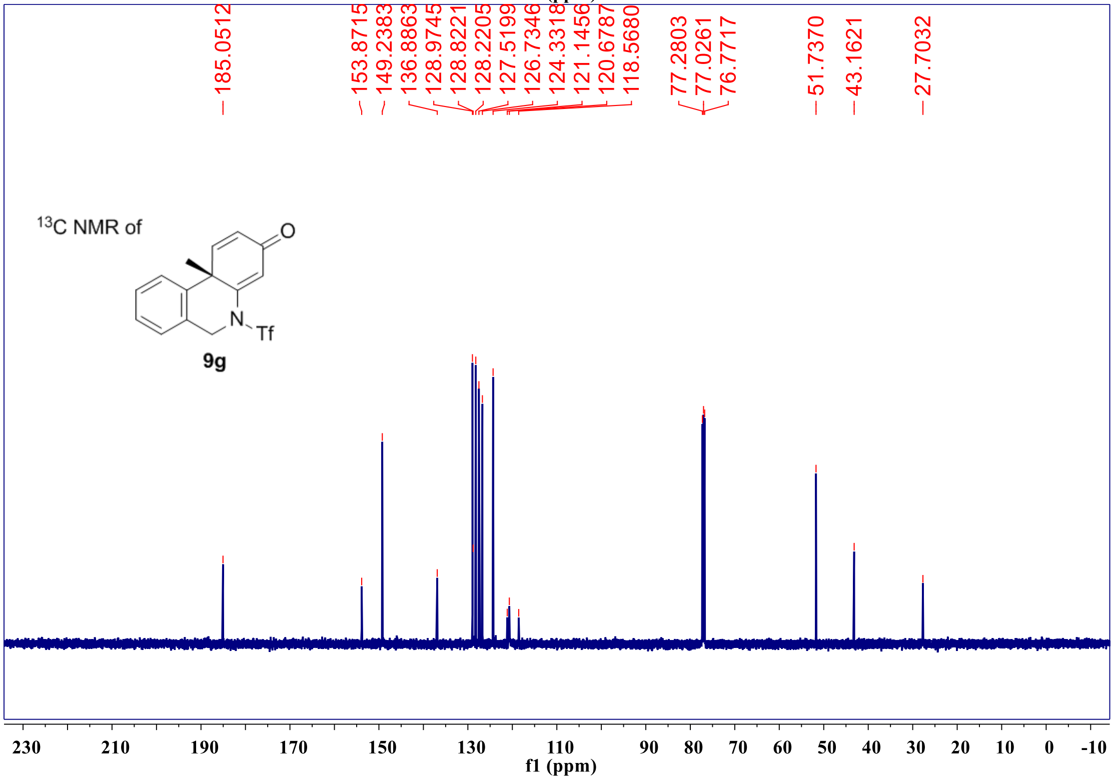
185.0512
153.8715
149.2383
136.8863
128.9745
128.8221
128.2205
127.5199
126.7346
124.3318
121.1456
120.6787
118.5680
77.2803
77.0261
76.7717
51.7370
43.1621
27.7032
13C NMR of
9g
f1 (ppm)

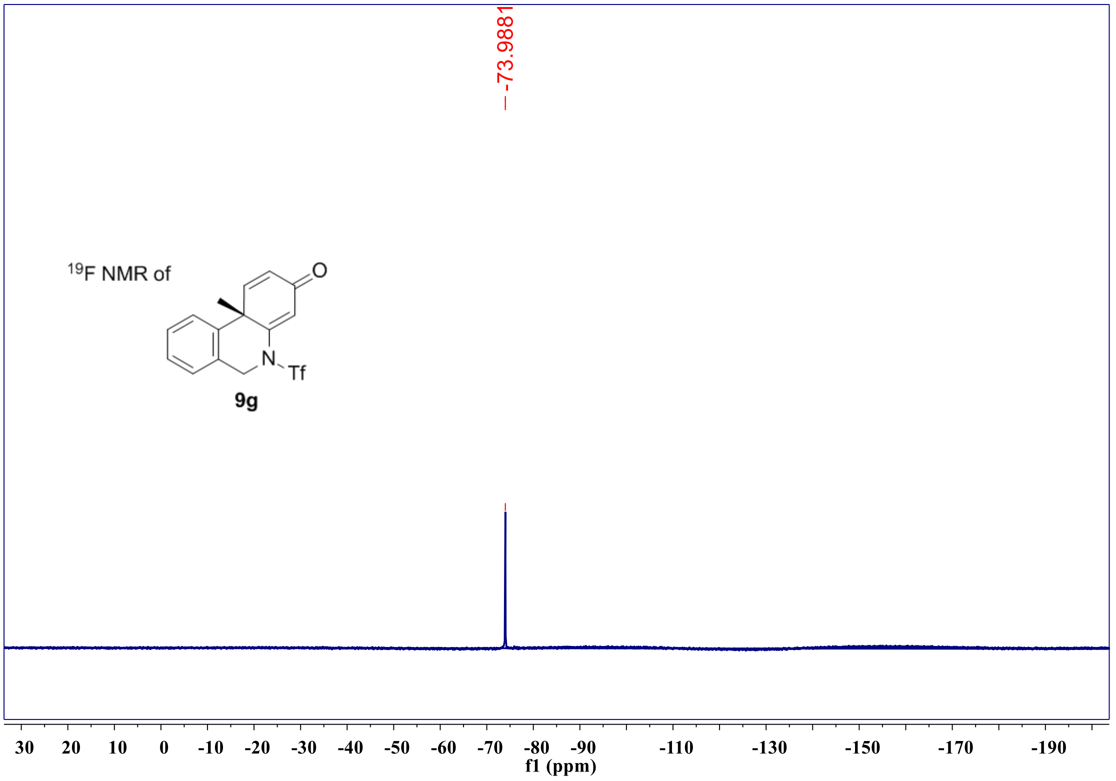
-73.9881
19F NMR of
O
N
Tf
9g
30 20 10 0 -10 -20 -30 -40 -50 -60 -70 -80 -90 -110 -130 -150 -170 -190
f1 (ppm)

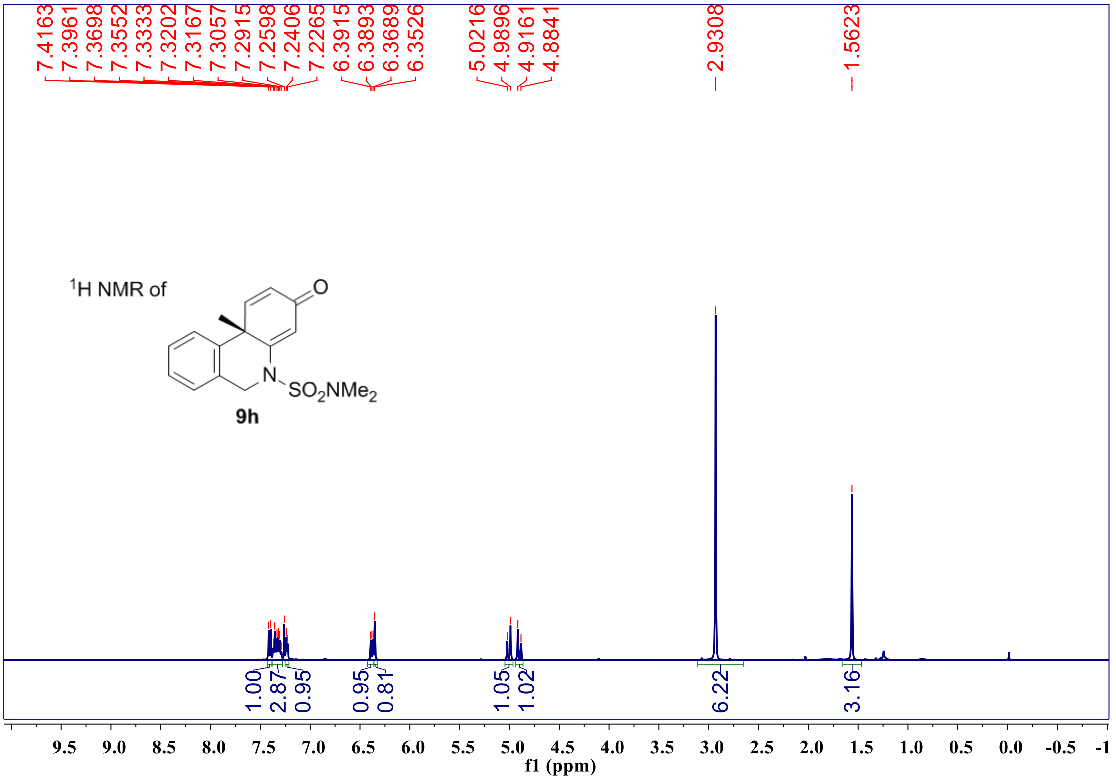
7.4163 7.3961 7.3698 7.3552 7.3333 7.3202 7.3167 7.3057 7.2915 7.2598 7.2406 7.2265 6.3915 6.3893 6.3689 6.3526 5.0216 4.9896 4.9161 4.8841 2.9308 1.5623
1H NMR of
O
N
SO2NMe2
9h
1.00 2.87 0.95 0.95 0.81 1.05 1.02 6.22 3.16
9.5 9.0 8.5 8.0 7.5 7.0 6.5 6.0 5.5 5.0 4.5 4.0 3.5 3.0 2.5 2.0 1.5 1.0 0.5 0.0 -0.5 -1
f1 (ppm)

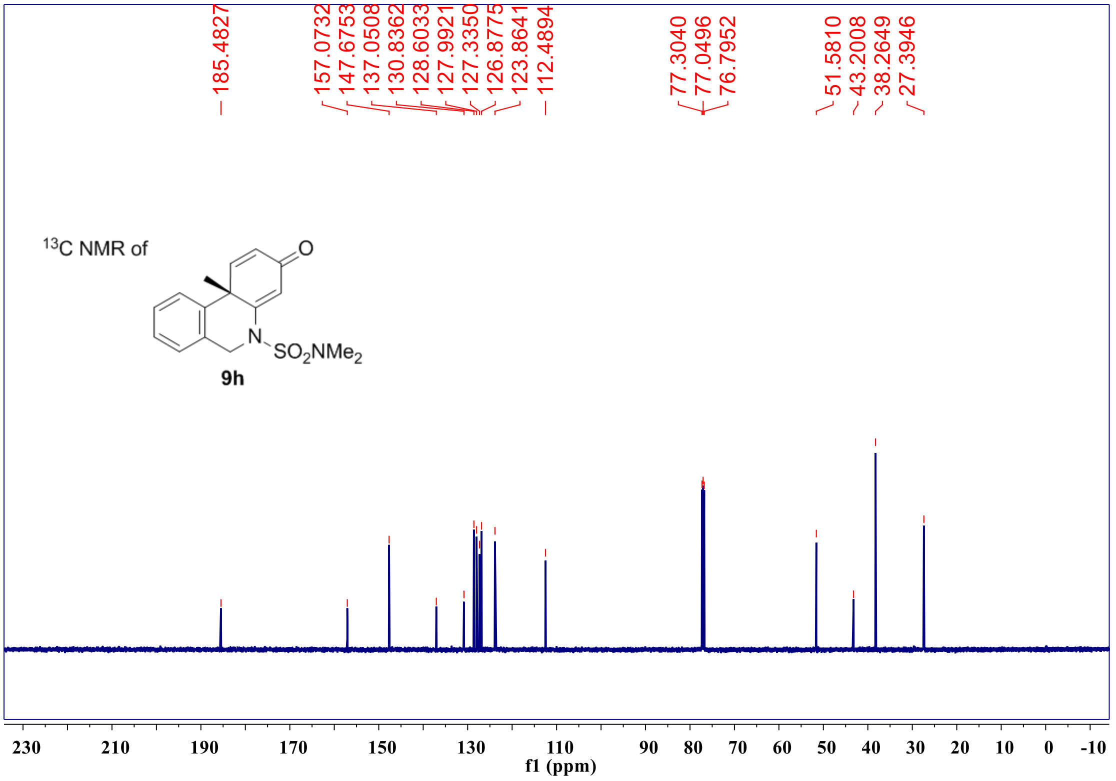
185.4827
157.0732
147.6753
137.0508
130.8362
128.6033
127.9921
127.3350
126.8775
123.8641
112.4894
77.3040
77.0496
76.7952
51.5810
43.2008
38.2649
27.3946
13C NMR of
O
N
SO2NMe2
9h
230 210 190 170 150 130 110 90 80 70 60 50 40 30 20 10 0 -10
f1 (ppm)

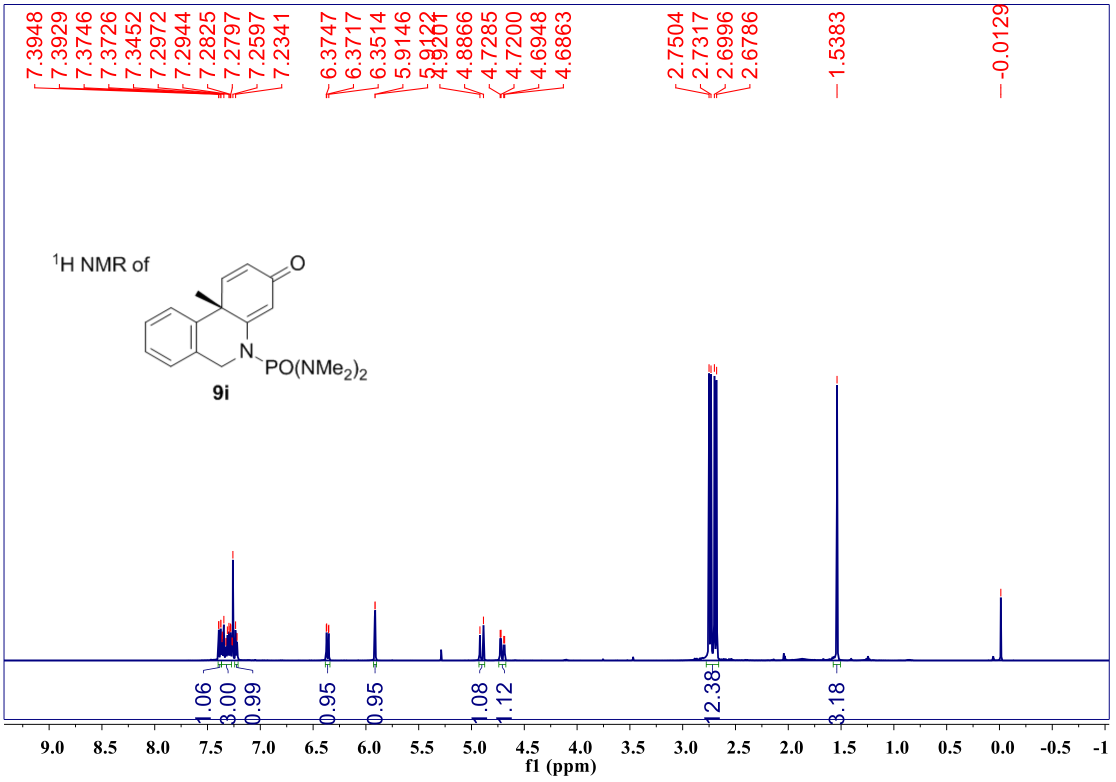
7.3948
7.3929
7.3746
7.3726
7.3452
7.2972
7.2944
7.2825
7.2797
7.2597
7.2341
6.3747
6.3717
6.3514
5.9146
5.9122
4.9201
4.8866
4.7285
4.7200
4.6948
4.6863
2.7504
2.7317
2.6996
2.6786
1.5383
-0.0129
1H NMR of
O
N
PO(NMe2)2
9i
1.06
3.00
0.99
0.95
0.95
1.08
1.12
12.38
3.18
9.0 8.5 8.0 7.5 7.0 6.5 6.0 5.5 5.0 4.5 4.0 3.5 3.0 2.5 2.0 1.5 1.0 0.5 0.0 -0.5 -1
f1 (ppm)

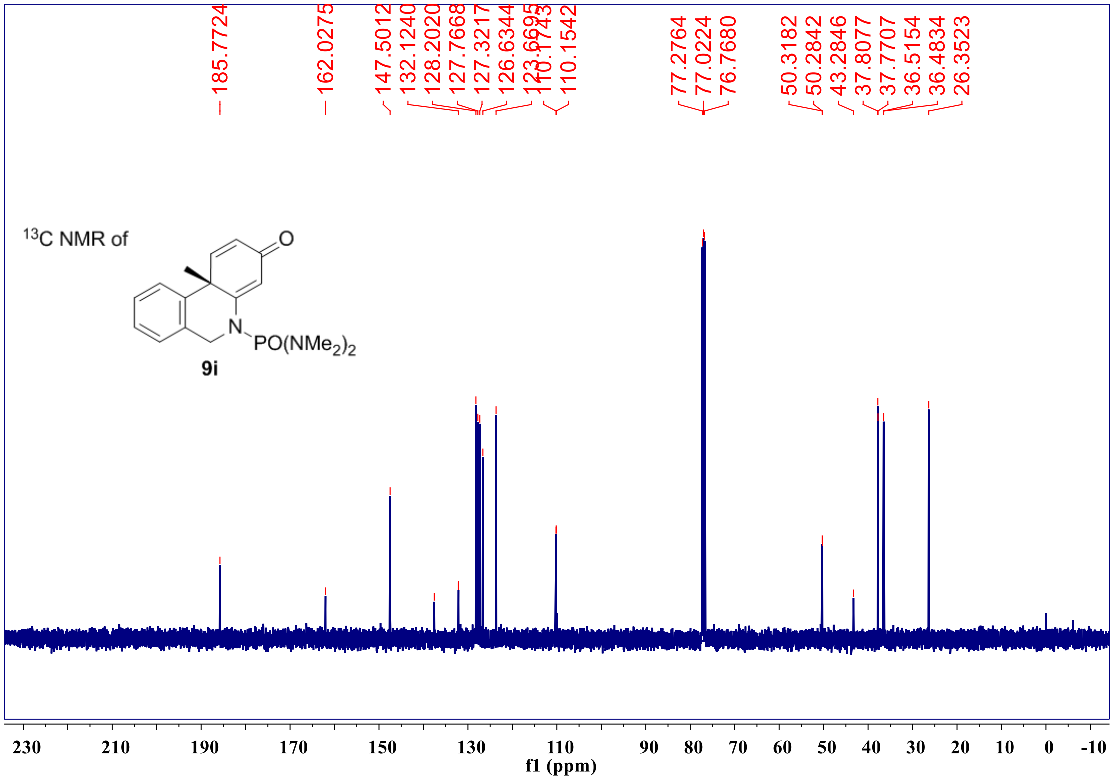
185.7724
162.0275
147.5012
132.1240
128.2020
127.7668
127.3217
126.6344
110.1542
77.2764
77.0224
76.7680
50.3182
50.2842
43.2846
37.8077
37.7707
36.5154
36.4834
26.3523
13C NMR of
O
N
PO(NMe2)2
9i
230 210 190 170 150 130 110 90 80 70 60 50 40 30 20 10 0 -10
f1 (ppm)

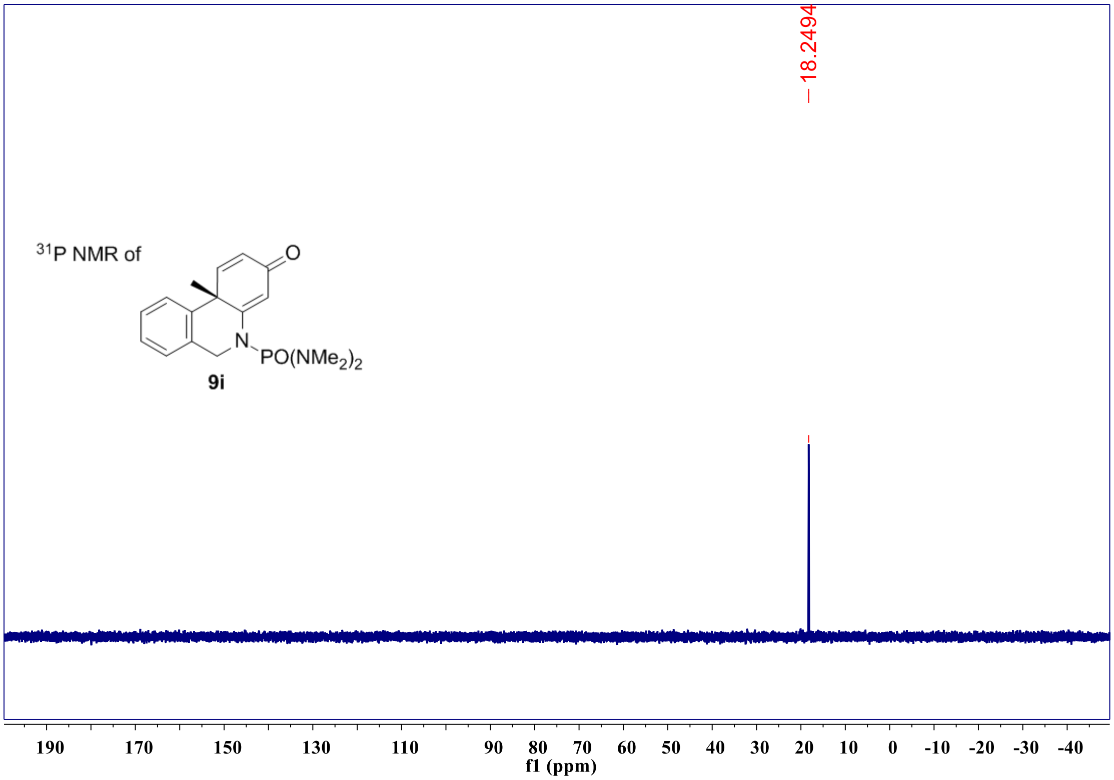
18.2494
31P NMR of
O
N
PO(NMe2)2
9i
190 170 150 130 110 90 80 70 60 50 40 30 20 10 0 -10 -20 -30 -40
f1 (ppm)

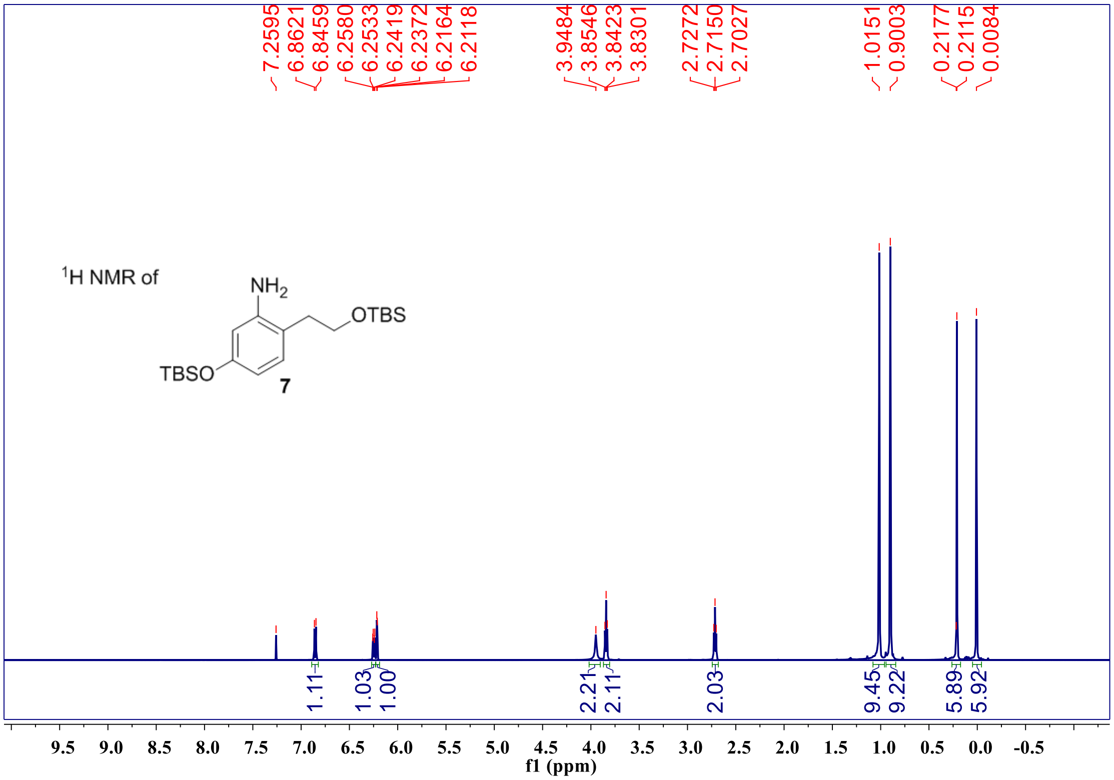

7.2595
6.8621
6.8459
6.2580
6.2533
6.2419
6.2372
6.2164
6.2118
3.9484
3.8546
3.8423
3.8301
2.7272
2.7150
2.7027
1.0151
0.9003
0.2177
0.2115
0.0084
1H NMR of
NH2
OTBS
TBSO
7
1.11
1.03
1.00
2.21
2.11
2.03
9.45
9.22
5.89
5.92
f1 (ppm)

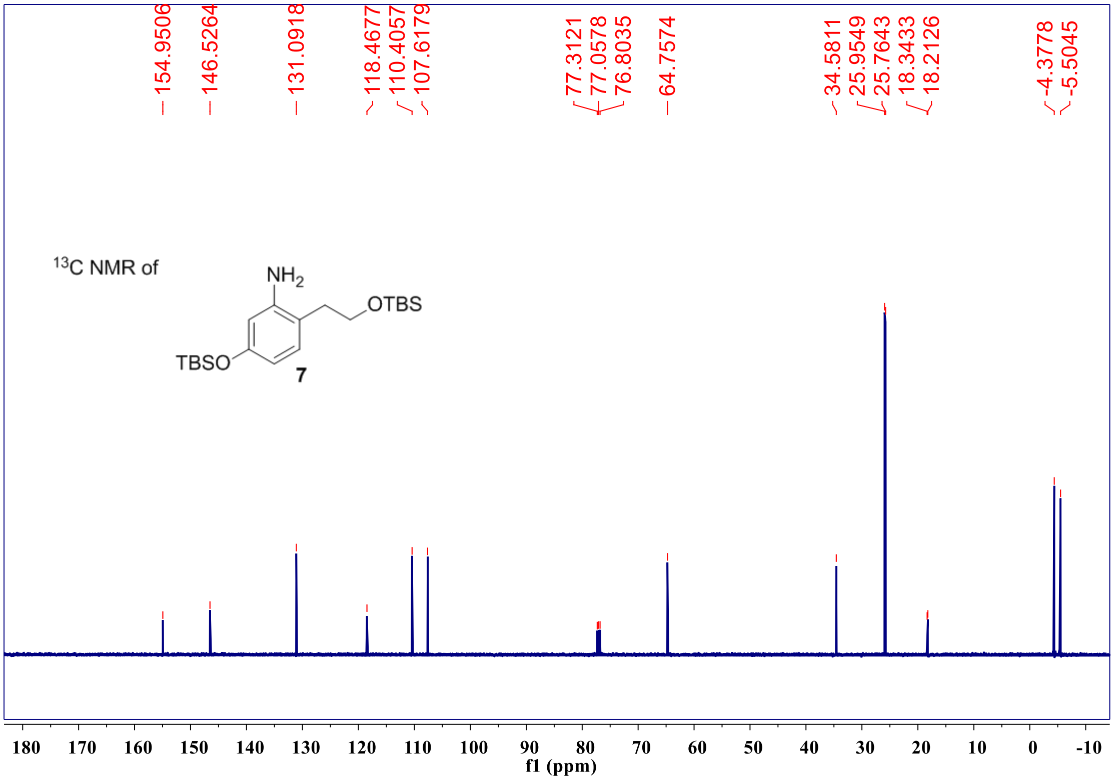

154.9506
146.5264
131.0918
118.4677
110.4057
107.6179
77.3121
77.0578
76.8035
64.7574
34.5811
25.9549
25.7643
18.3433
18.2126
-4.3778
-5.5045
13C NMR of
NH2
OTBS
TBSO
7
f1 (ppm)

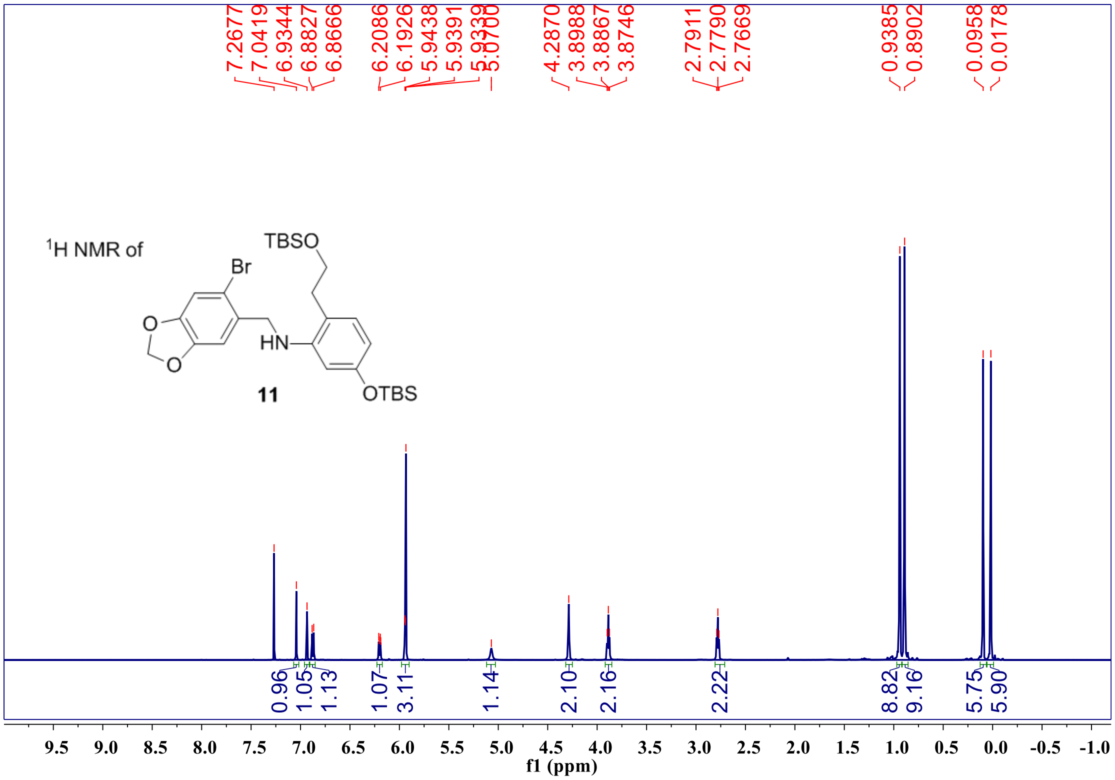

7.2677
7.0419
6.9344
6.8827
6.8666
6.2086
6.1926
5.9438
5.9391
5.9339
5.0700
4.2870
3.8988
3.8867
3.8746
2.7911
2.7790
2.7669
0.9385
0.8902
0.0958
0.0178
^{1}H NMR of
TBSO
Br
O
O
HN
11
OTBS
0.96
1.05
1.13
1.07
3.11
1.14
2.10
2.16
2.22
8.82
9.16
5.75
5.90
9.5 9.0 8.5 8.0 7.5 7.0 6.5 6.0 5.5 5.0 4.5 4.0 3.5 3.0 2.5 2.0 1.5 1.0 0.5 0.0 -0.5 -1.0
f1 (ppm)

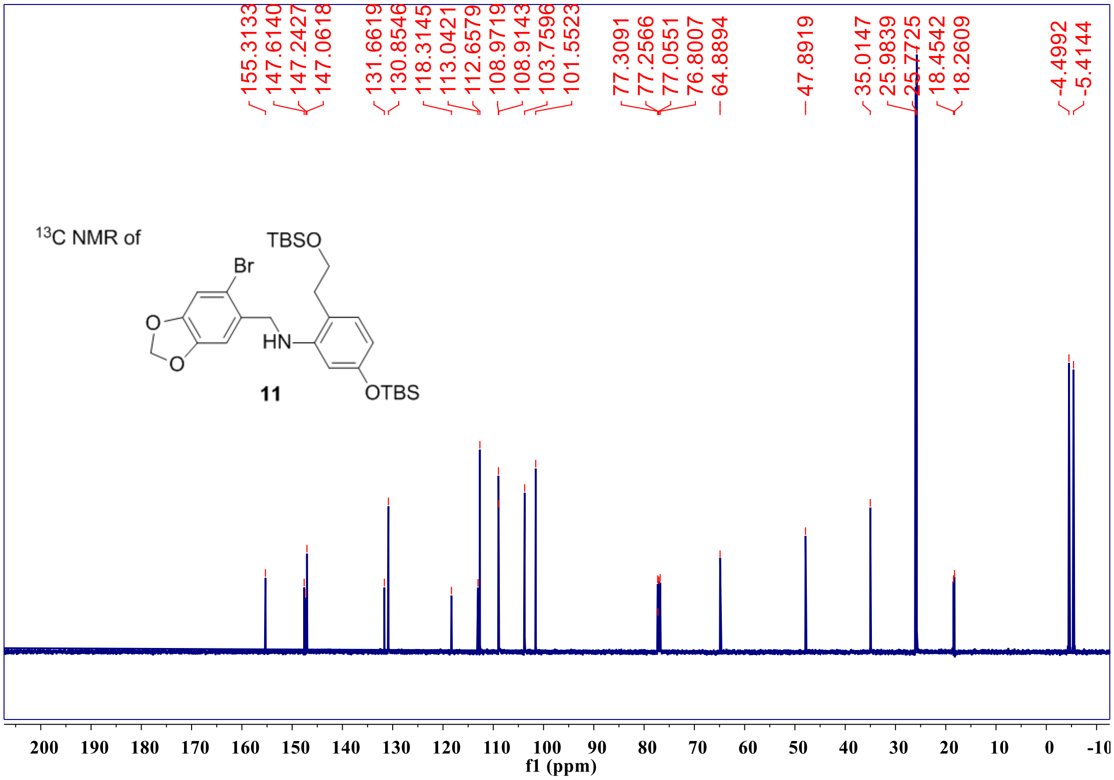

155.3133
147.6140
147.2427
147.0618
131.6619
130.8546
118.3145
113.0421
112.6579
108.9719
108.9143
103.7596
101.5523
77.3091
77.2566
77.0551
76.8007
64.8894
47.8919
35.0147
25.9839
25.7725
18.4542
18.2609
-4.4992
-5.4144
^{13}C NMR of
TBSO
Br
O
O
HN
11
OTBS
200 190 180 170 160 150 140 130 120 110 100 90 80 70 60 50 40 30 20 10 0 -10
f1 (ppm)

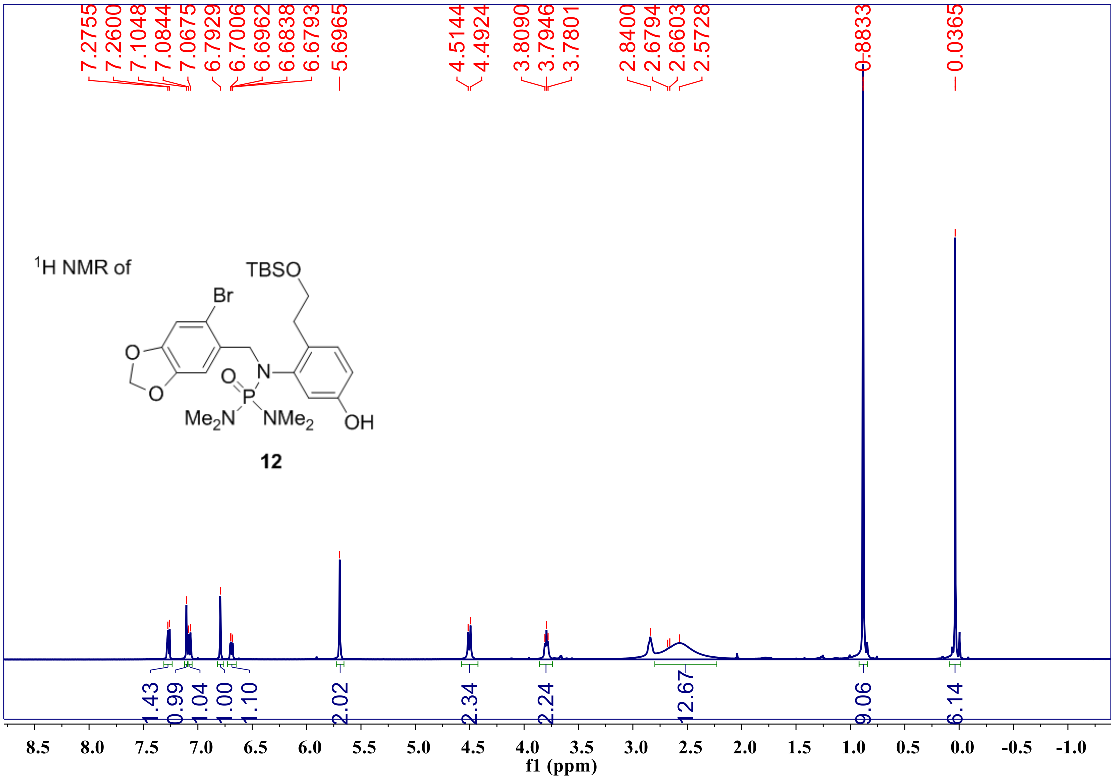

7.2755
7.2600
7.1048
7.0844
7.0675
6.7929
6.7006
6.6962
6.6838
6.6793
5.6965
4.5144
4.4924
3.8090
3.7946
3.7801
2.8400
2.6794
2.6603
2.5728
0.8833
0.0365
^{1}H NMR of
TBSO
Br
O
O
O
P
N
Me$_2$N
NMe$_2$
OH
12
1.43
0.99
1.04
1.00
1.10
2.02
2.34
2.24
12.67
9.06
6.14
f1 (ppm)

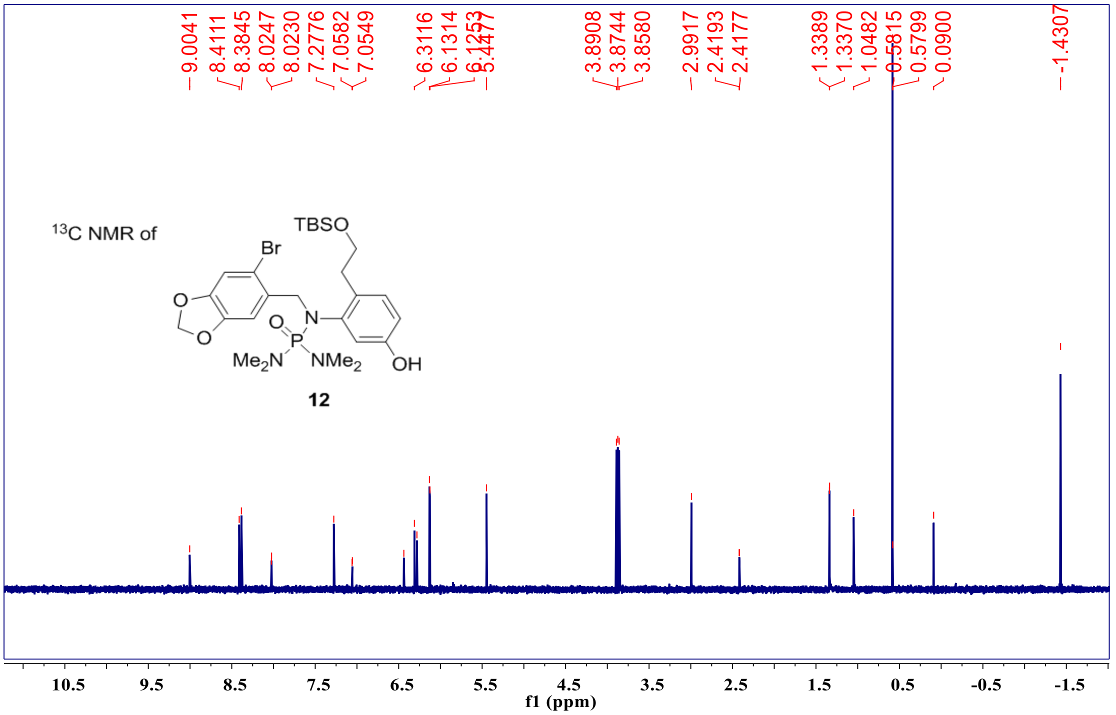

9.0041
8.4111
8.3845
8.0247
8.0230
7.2776
7.0582
7.0549
6.3116
6.1314
3.8908
3.8744
3.8580
2.9917
2.4193
2.4177
1.3389
1.3370
1.0482
0.5815
0.5799
0.0900
-1.4307
^{13}C NMR of
TBSO
Br
O
O
O
P
N
Me$_2$N
NMe$_2$
OH
12
f1 (ppm)

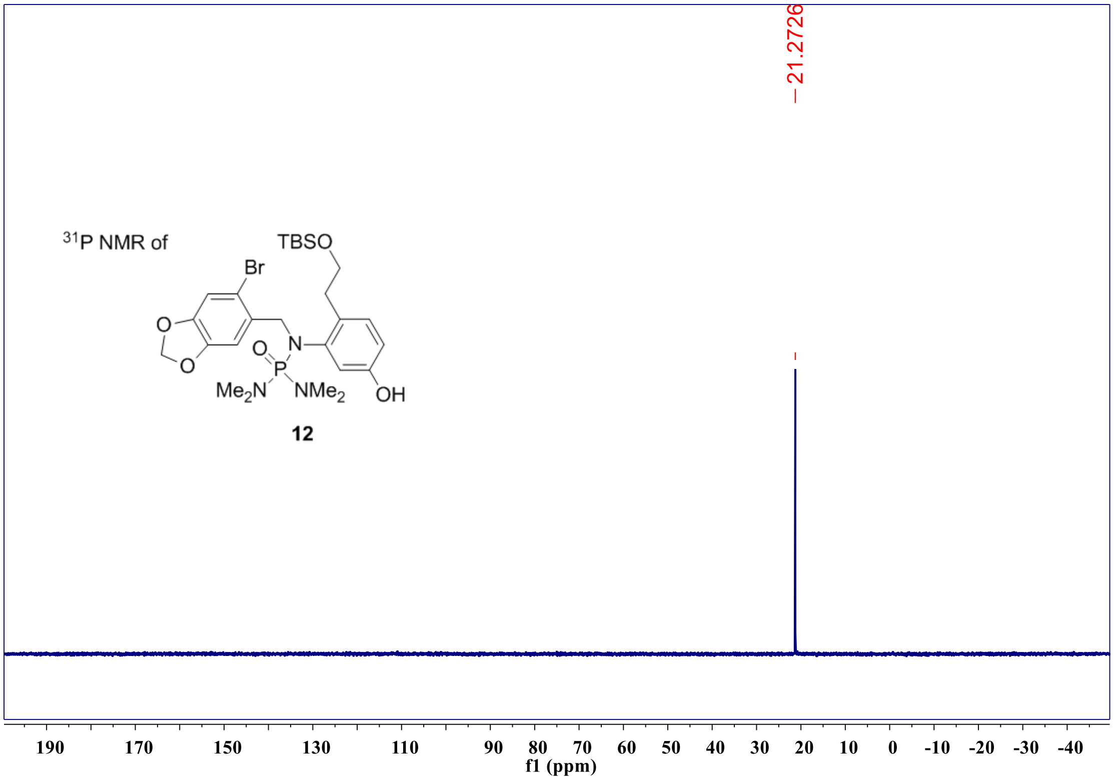

21.2726
31P NMR of
TBSO
Br
O
O
N
O
P
Me2N
NMe2
OH
12
190 170 150 130 110 90 80 70 60 50 40 30 20 10 0 -10 -20 -30 -40
f1 (ppm)

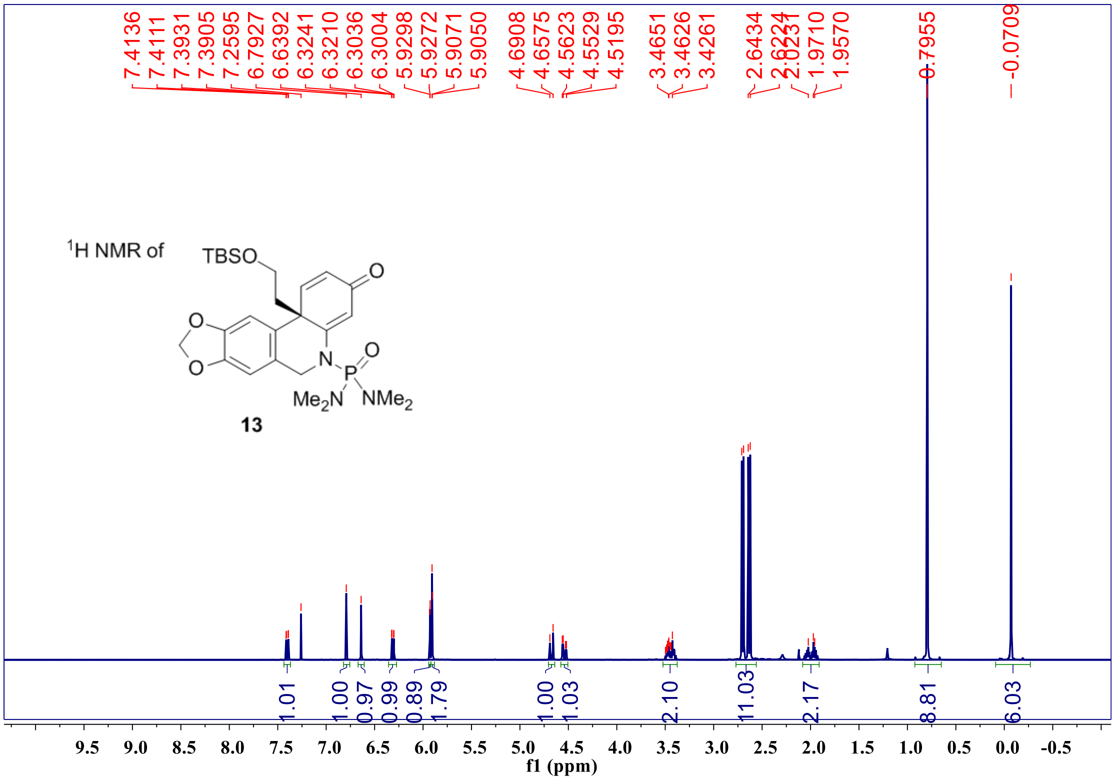

7.4136
7.4111
7.3931
7.3905
7.2595
6.7927
6.6392
6.3241
6.3210
6.3036
6.3004
5.9298
5.9272
5.9071
5.9050
4.6908
4.6575
4.5623
4.5529
4.5195
3.4651
3.4626
3.4261
2.6434
1.9710
1.9570
0.7955
-0.0709
1H NMR of
TBSO
O
O
O
N
P
O
Me2N
NMe2
13
1.01
1.00
0.97
0.99
0.89
1.79
1.00
1.03
2.10
11.03
2.17
8.81
6.03
9.5 9.0 8.5 8.0 7.5 7.0 6.5 6.0 5.5 5.0 4.5 4.0 3.5 3.0 2.5 2.0 1.5 1.0 0.5 0.0 -0.5
f1 (ppm)

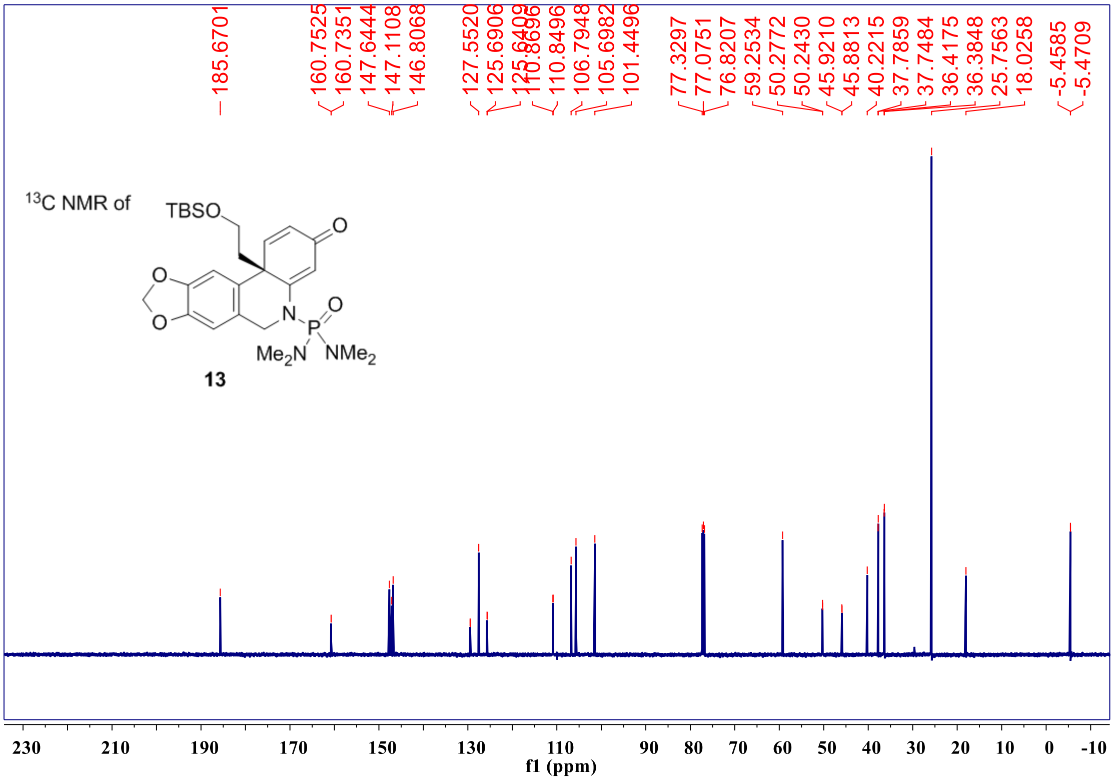
185.6701
160.7525
160.7351
147.6444
147.1108
146.8068
127.5520
125.6906
110.8496
106.7948
105.6982
101.4496
77.3297
77.0751
76.8207
59.2534
50.2772
50.2430
45.9210
45.8813
40.2215
37.7859
37.7484
36.4175
36.3848
25.7563
18.0258
-5.4585
-5.4709
^{13}C NMR of
TBSO
O
N
P
Me$_2$N
NMe$_2$
13
f1 (ppm)

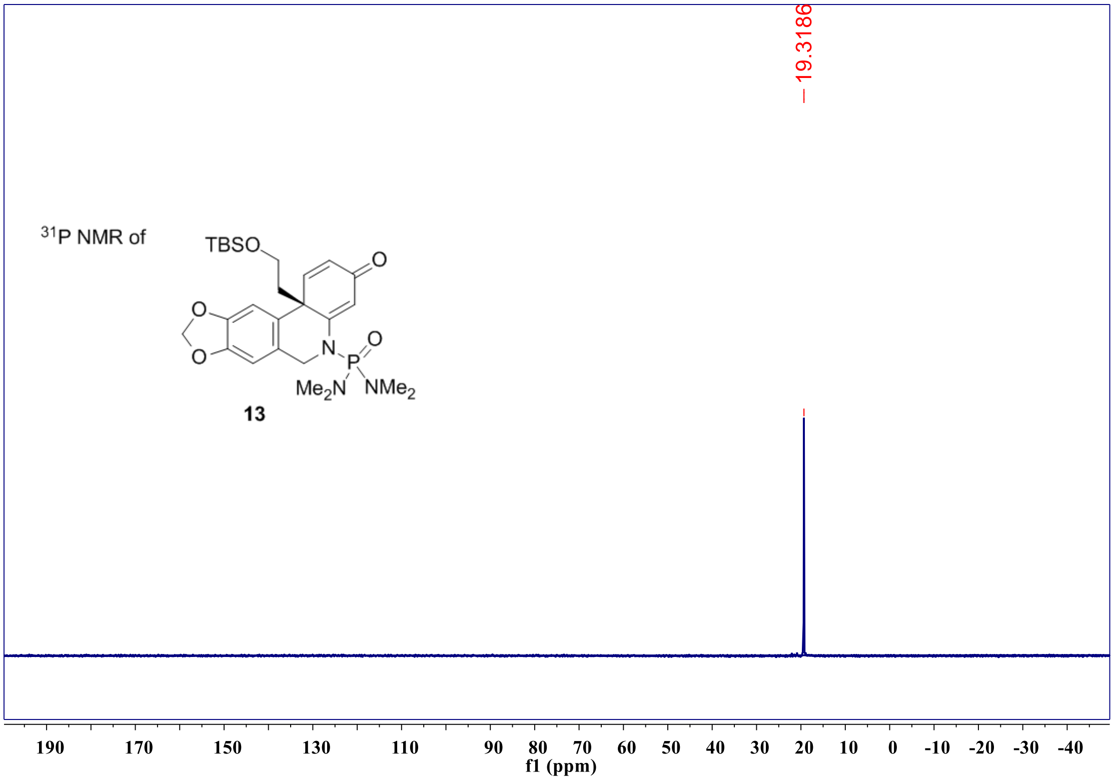
19.3186
^{31}P NMR of
TBSO
O
N
P
Me$_2$N
NMe$_2$
13
f1 (ppm)

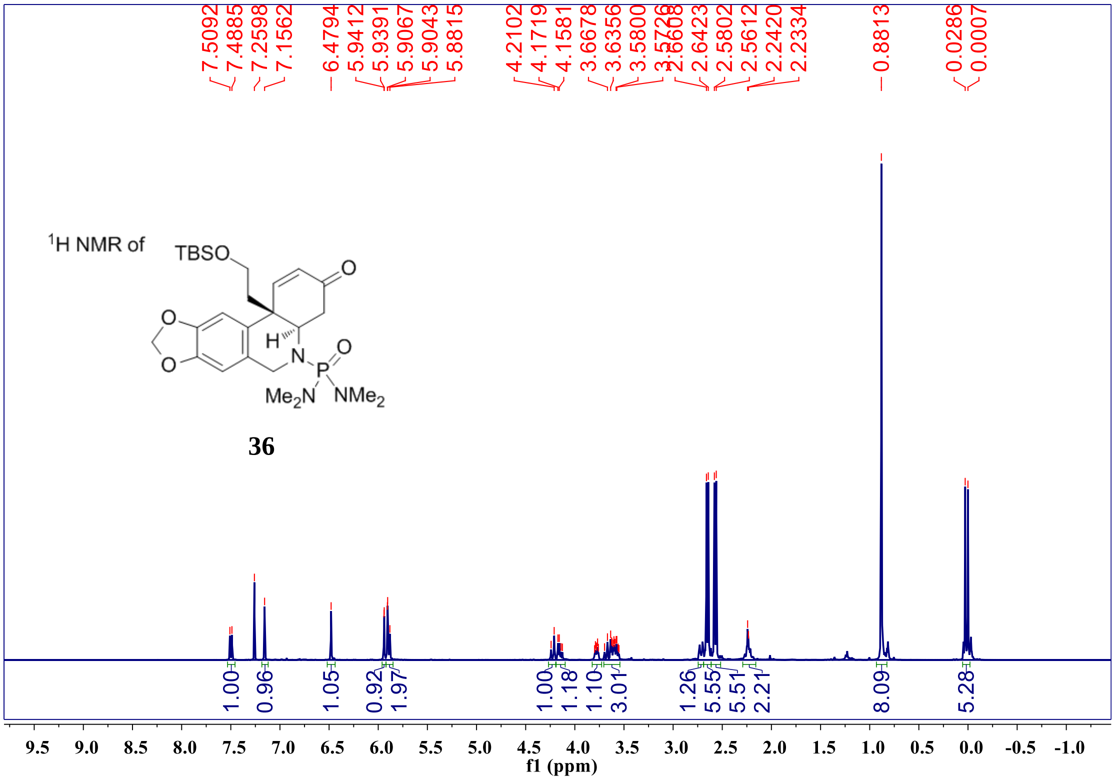
7.5092
7.4885
7.2598
7.1562
6.4794
5.9412
5.9391
5.9067
5.9043
5.8815
4.2102
4.1719
4.1581
3.6678
3.6356
3.5800
2.6423
2.5802
2.5612
2.2420
2.2334
0.8813
0.0286
0.0007
1H NMR of
TBSO
O
O
O
H
N
P
O
Me2N
NMe2
36
1.00
0.96
1.05
0.92
1.97
1.00
1.18
1.10
3.01
1.26
5.55
5.51
2.21
8.09
5.28
f1 (ppm)

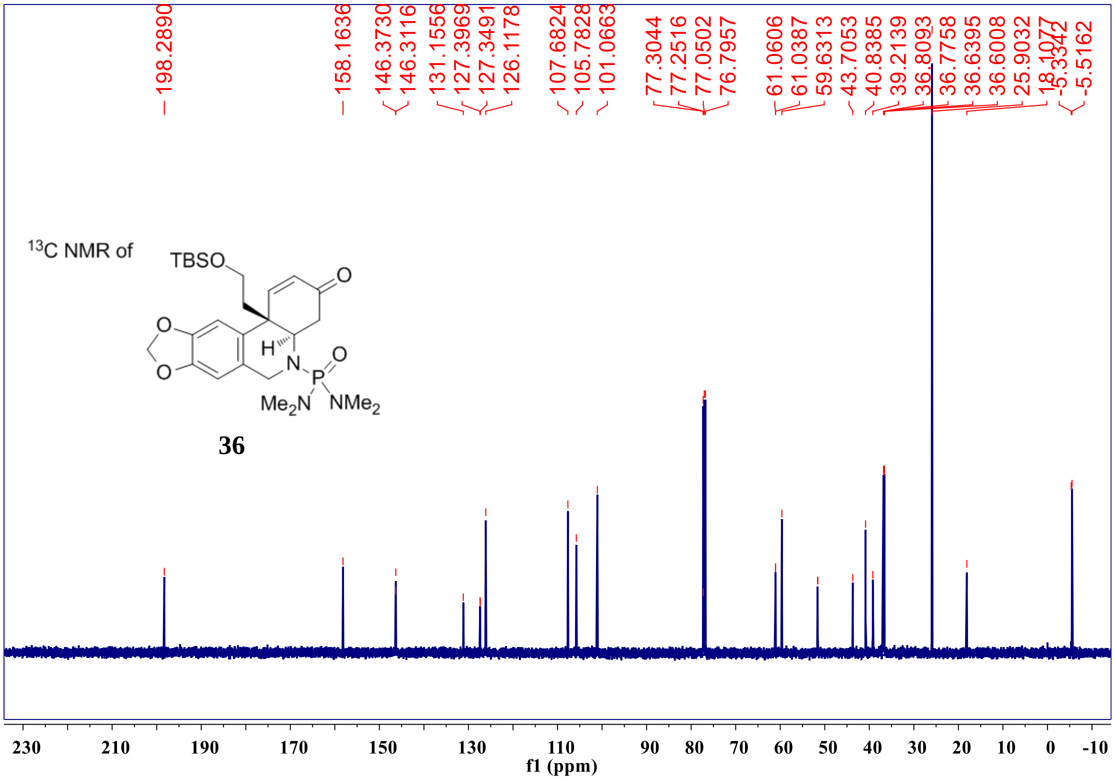
198.2890
158.1636
146.3730
146.3116
131.1556
127.3969
127.3491
126.1178
107.6824
105.7828
101.0663
77.3044
77.2516
77.0502
76.7957
61.0606
61.0387
59.6313
43.7053
40.8385
39.2139
36.8093
36.7758
36.6395
36.6008
25.9032
-5.5162
13C NMR of
TBSO
O
O
O
H
N
P
O
Me2N
NMe2
36
f1 (ppm)

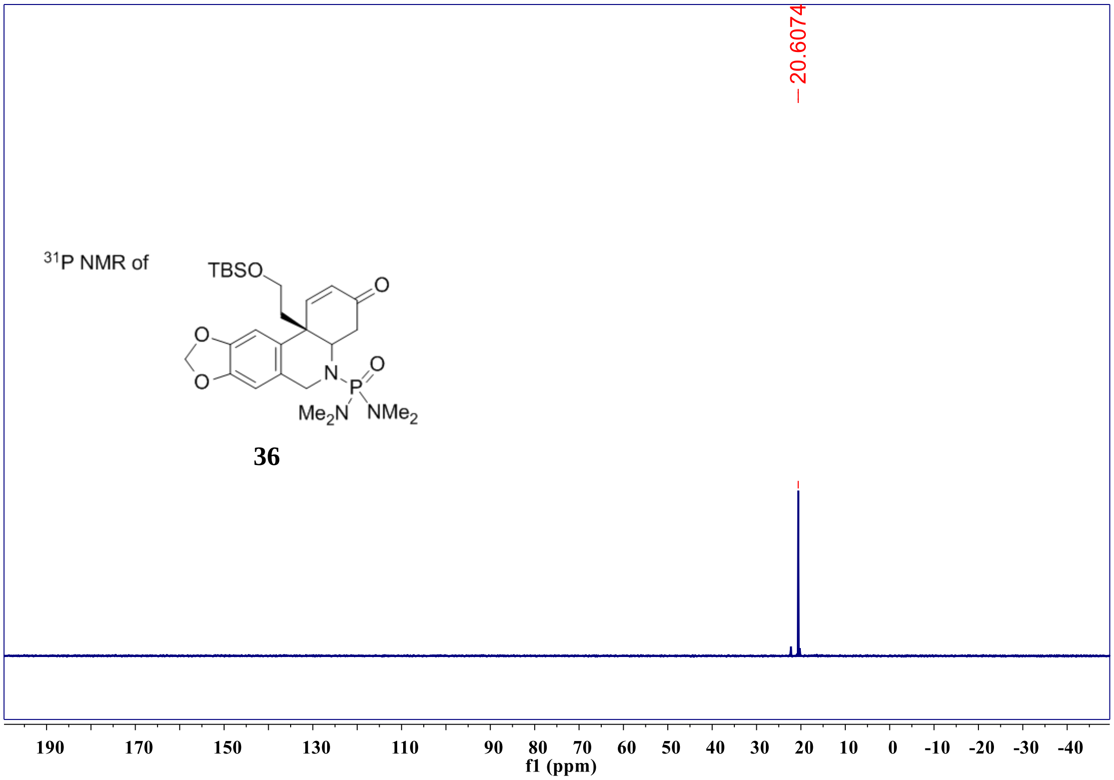

20.6074
31P NMR of
TBSO
O
O
O
N
P
O
Me2N
NMe2
36
190
170
150
130
110
90
80
70
60
50
40
30
20
10
0
-10
-20
-30
-40
f1 (ppm)

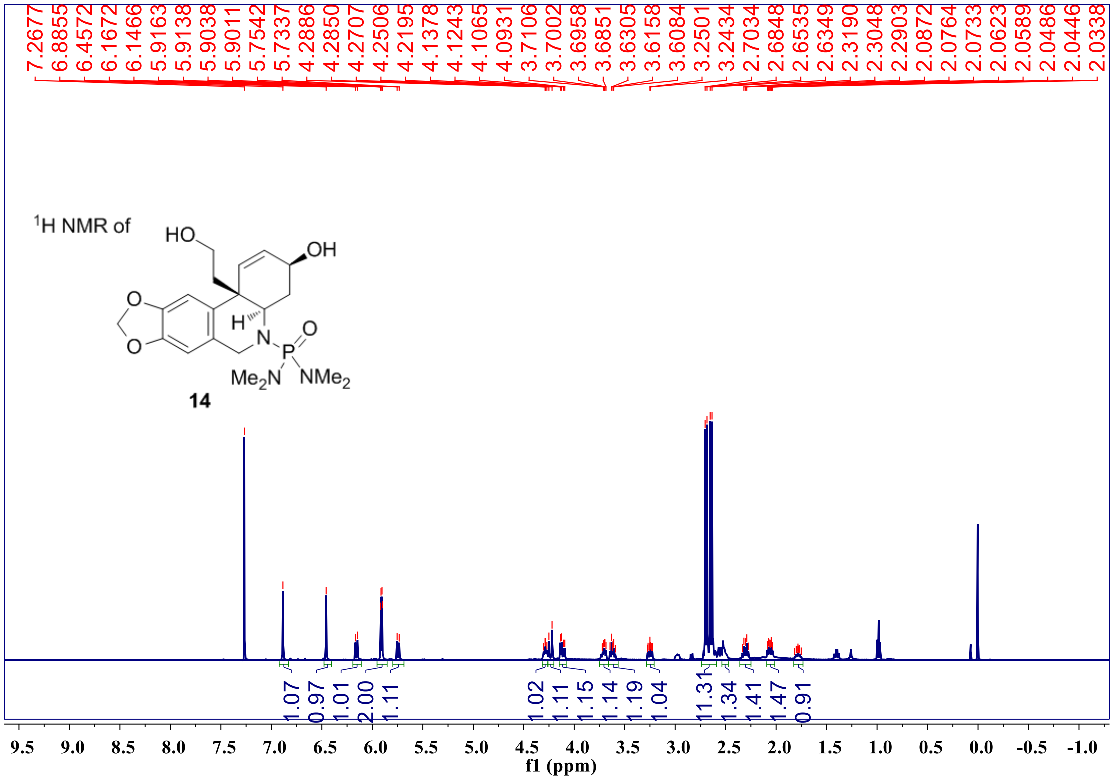

7.2677
6.8855
6.4572
6.1672
6.1466
5.9163
5.9138
5.9038
5.9011
5.7542
5.7337
4.2886
4.2850
4.2707
4.2506
4.2195
4.1378
4.1243
4.1065
4.0931
3.7106
3.7002
3.6958
3.6851
3.6305
3.6158
3.6084
3.2501
3.2434
2.7034
2.6848
2.6535
2.6349
2.3190
2.3048
2.2903
2.0872
2.0764
2.0733
2.0623
2.0589
2.0486
2.0446
2.0338
1H NMR of
HO
OH
O
O
H
N
P
O
Me2N
NMe2
14
1.07
0.97
1.01
2.00
1.11
1.02
1.11
1.15
1.14
1.19
1.04
11.31
1.34
1.41
1.47
0.91
9.5
9.0
8.5
8.0
7.5
7.0
6.5
6.0
5.5
5.0
4.5
4.0
3.5
3.0
2.5
2.0
1.5
1.0
0.5
0.0
-0.5
-1.0
f1 (ppm)

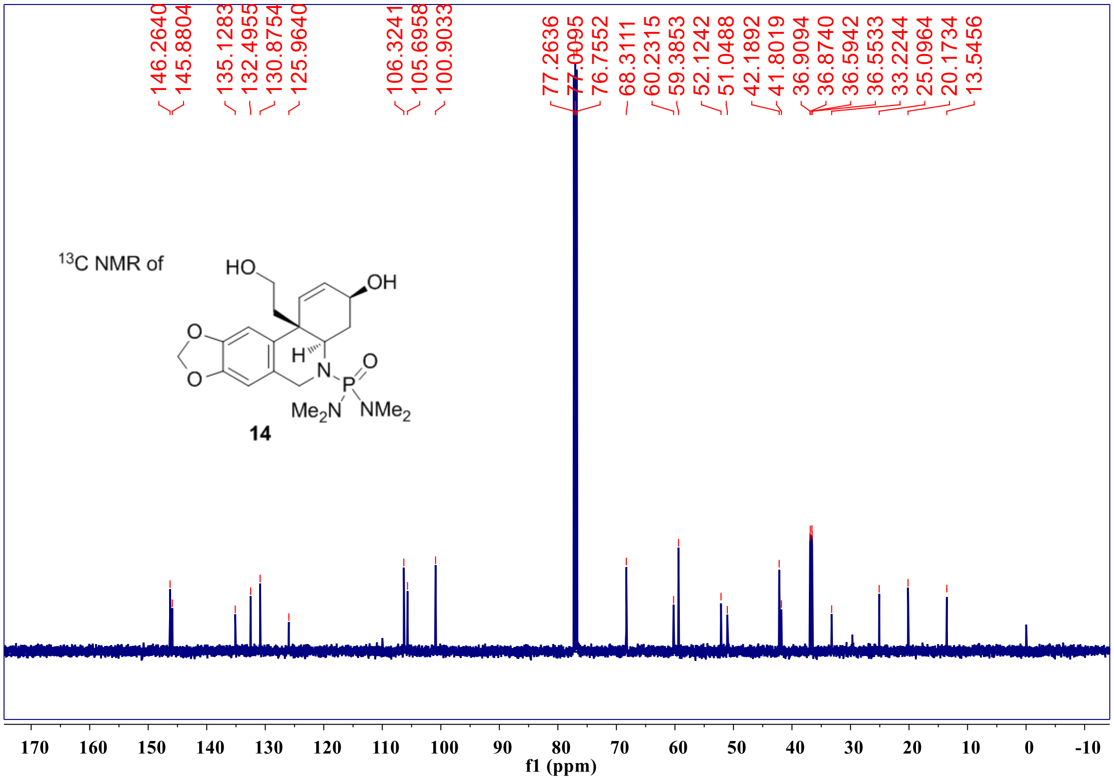
146.2640
145.8804
135.1283
132.4955
130.8754
125.9640
106.3241
105.6958
100.9033
77.2636
77.0095
76.7552
68.3111
60.2315
59.3853
52.1242
51.0488
42.1892
41.8019
36.9094
36.8740
36.5942
36.5533
33.2244
25.0964
20.1734
13.5456
13C NMR of
HO
OH
O
O
H
N
P
O
Me2N
NMe2
14
f1 (ppm)

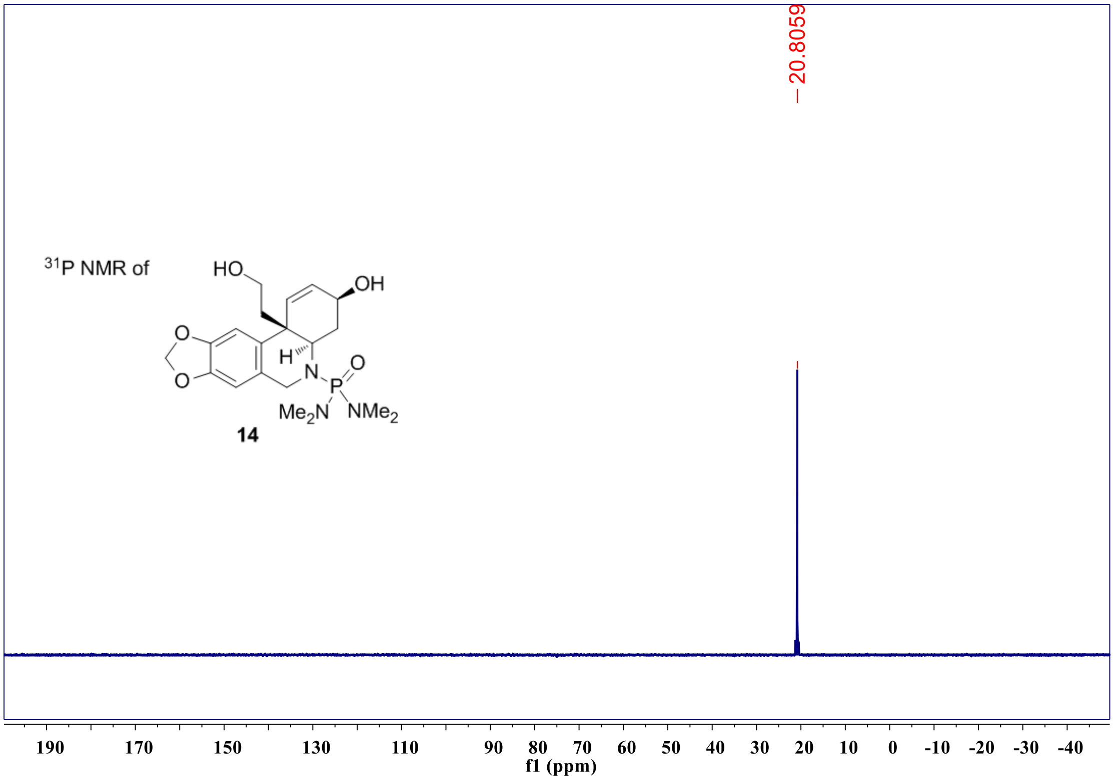
20.8059
31P NMR of
HO
OH
O
O
H
N
P
O
Me2N
NMe2
14
f1 (ppm)

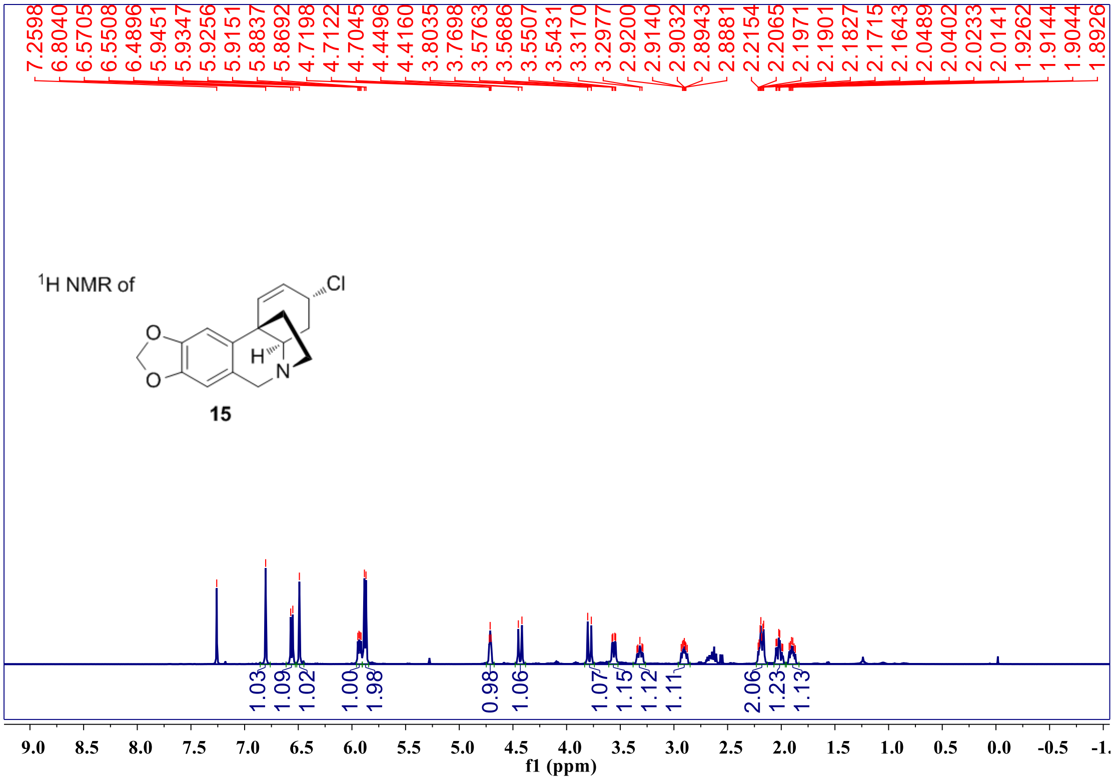
7.2598 6.8040 6.5705 6.5508 6.4896 5.9451 5.9347 5.9256 5.9151 5.8837 5.8692 4.7198 4.7122 4.7045 4.4496 4.4160 3.8035 3.7698 3.5763 3.5686 3.5507 3.5431 3.3170 3.2977 2.9200 2.9140 2.9032 2.8943 2.8881 2.2154 2.2065 2.1971 2.1901 2.1827 2.1715 2.1643 2.0489 2.0402 2.0233 2.0141 1.9262 1.9144 1.9044 1.8926
1H NMR of
Cl
O
O
H
N
15
1.03 1.09 1.02 1.00 1.98 0.98 1.06 1.07 1.15 1.12 1.11 2.06 1.23 1.13
9.0 8.5 8.0 7.5 7.0 6.5 6.0 5.5 5.0 4.5 4.0 3.5 3.0 2.5 2.0 1.5 1.0 0.5 0.0 -0.5
f1 (ppm)

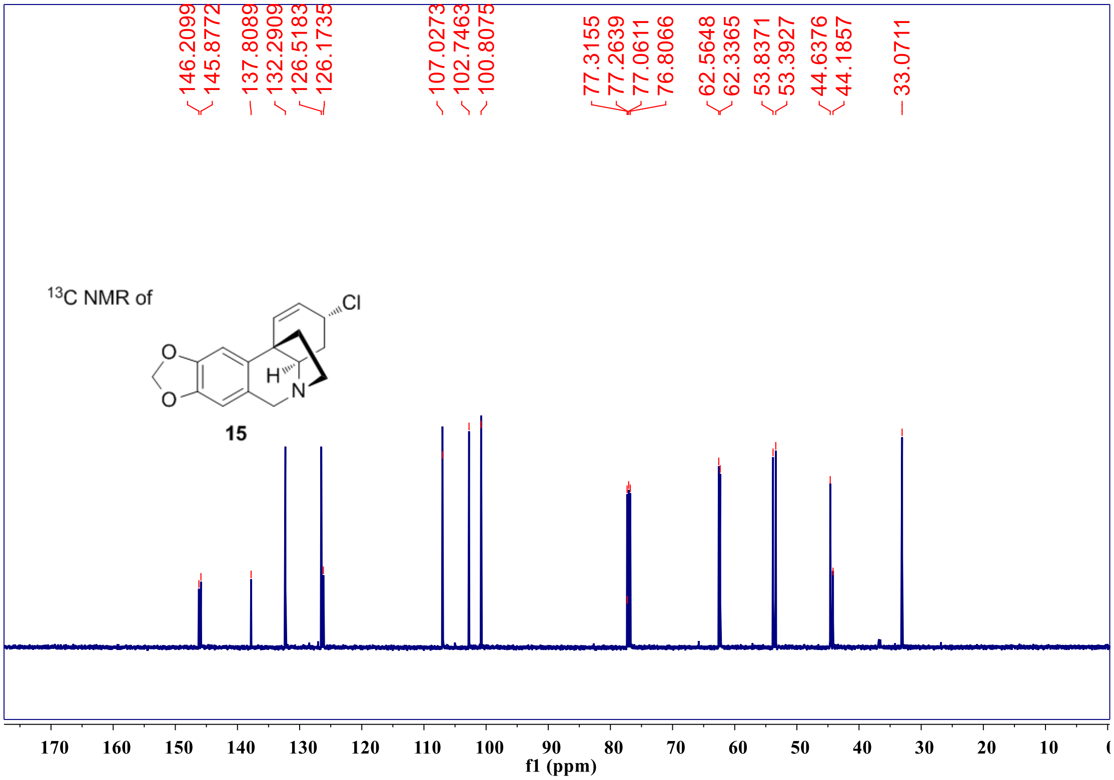
146.2099 145.8772 137.8089 132.2909 126.5183 126.1735 107.0273 102.7463 100.8075 77.3155 77.2639 77.0611 76.8066 62.5648 62.3365 53.8371 53.3927 44.6376 44.1857 33.0711
13C NMR of
Cl
O
O
H
N
15
170 160 150 140 130 120 110 100 90 80 70 60 50 40 30 20 10
f1 (ppm)

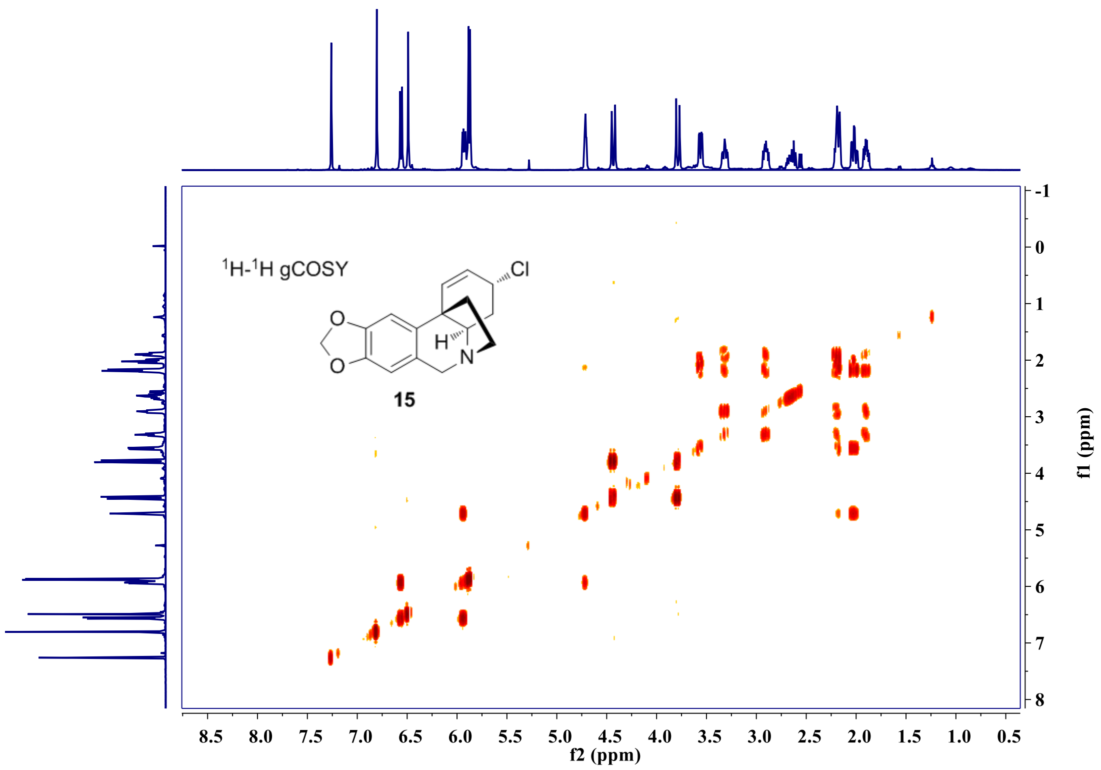

1H-1H gCOSY
15
f2 (ppm)
f1 (ppm)

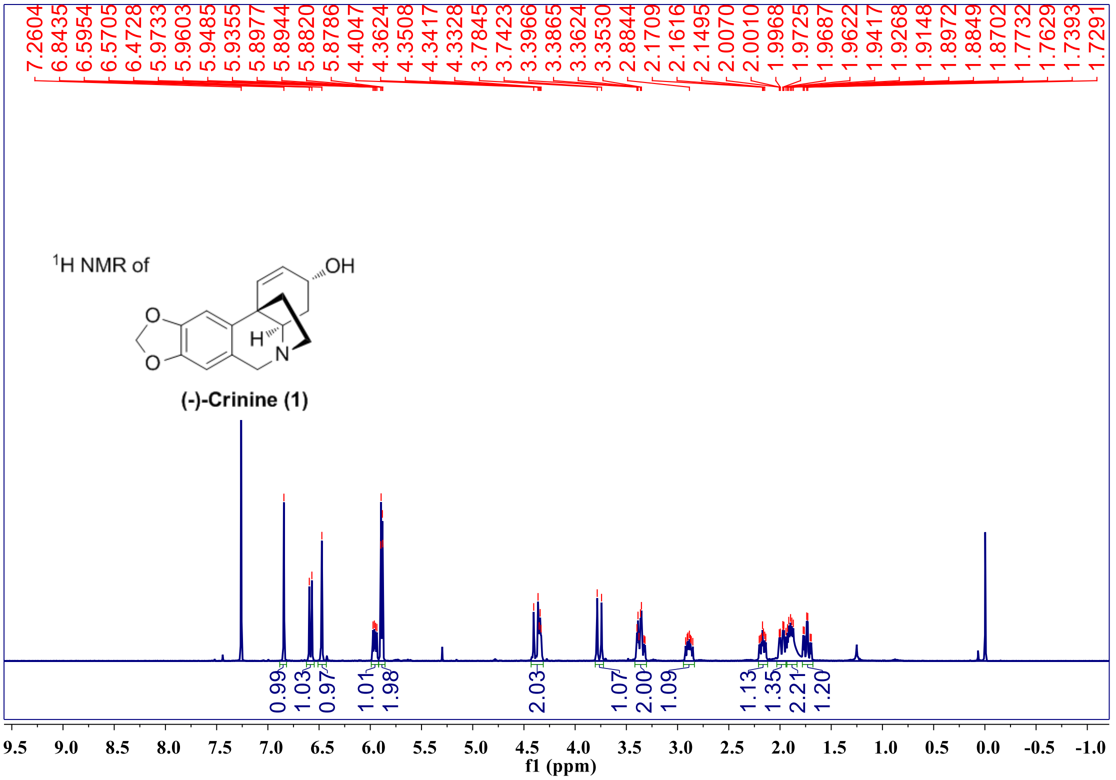

1H NMR of
(-)-Crinine (1)
f1 (ppm)

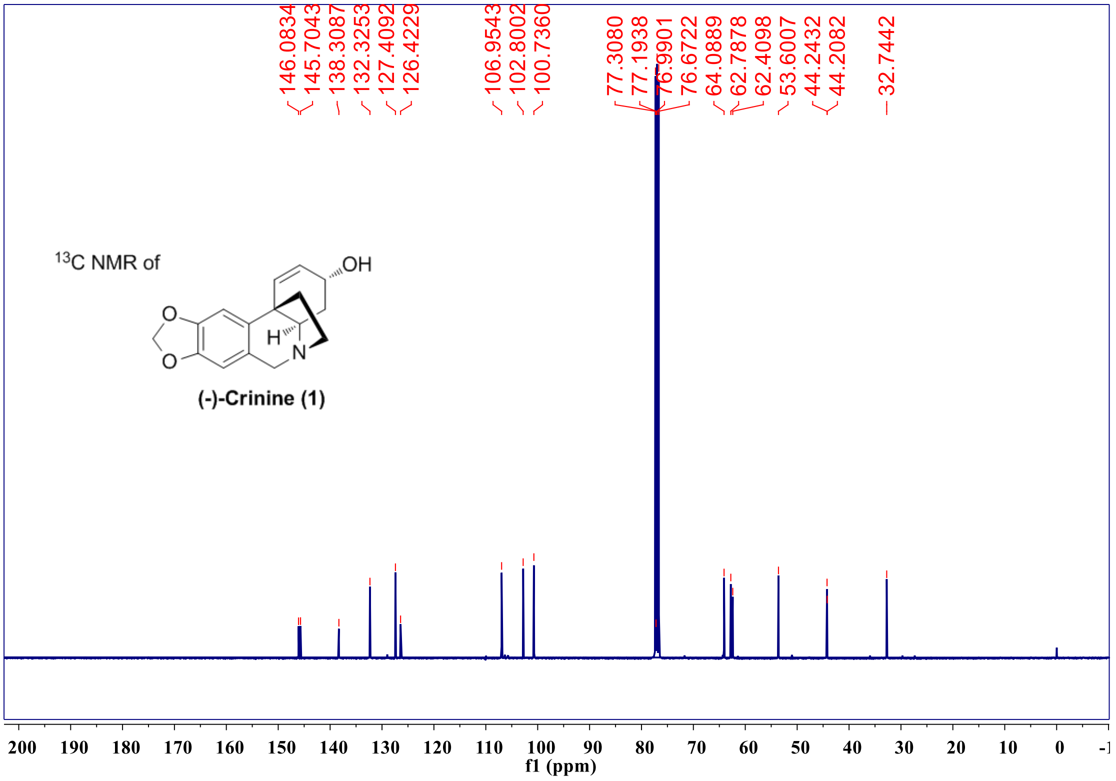
146.0834
145.7043
138.3087
132.3253
127.4092
126.4229
106.9543
102.8002
100.7360
77.3080
77.1938
76.9901
76.6722
64.0889
62.7878
62.4098
53.6007
44.2432
44.2082
32.7442
^{13}C NMR of
OH
O
O
H
N
(-)-Crinine (1)
f1 (ppm)

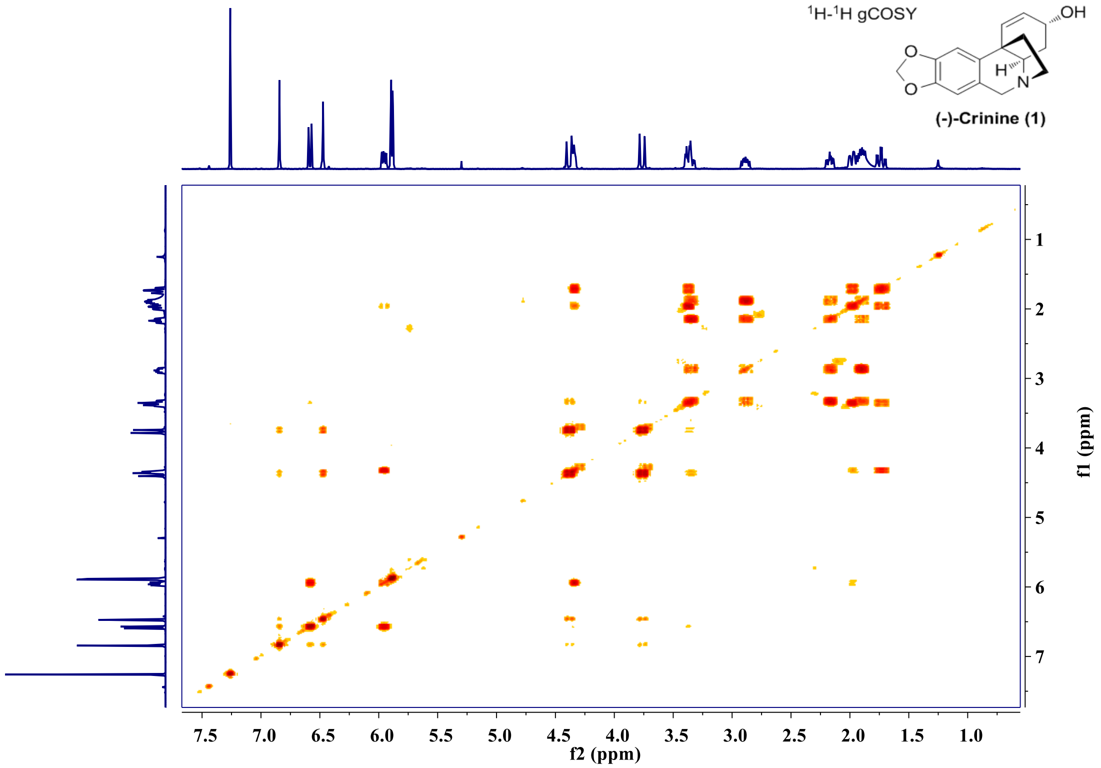
^{1}H-^{1}H gCOSY
OH
O
O
H
N
(-)-Crinine (1)
f2 (ppm)
f1 (ppm)

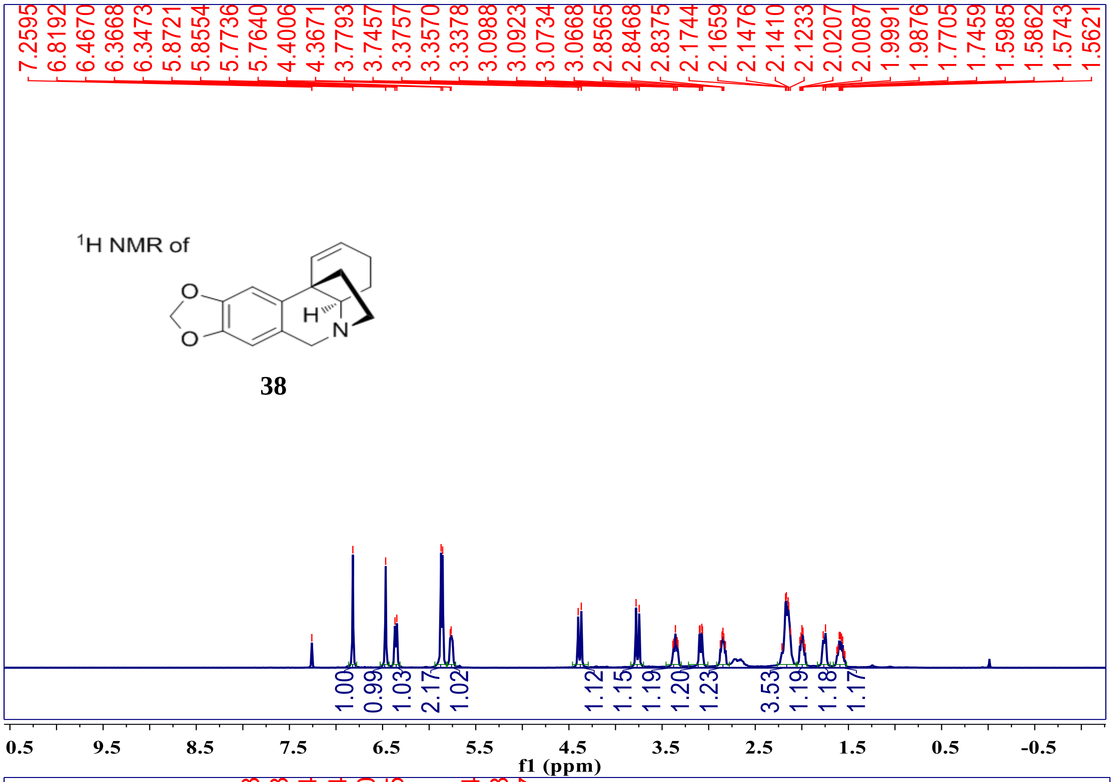
7.2595 6.8192 6.4670 6.3668 6.3473 5.8721 5.8554 5.7736 5.7640 4.4006 4.3671 3.7793 3.7457 3.3757 3.3570 3.3378 3.0988 3.0923 3.0734 3.0668 2.8565 2.8468 2.8375 2.1744 2.1659 2.1476 2.1410 2.1233 2.0207 2.0087 1.9991 1.9876 1.7705 1.7459 1.5985 1.5862 1.5743 1.5621
^{1}H NMR of
38
1.00 0.99 1.03 2.17 1.02 1.12 1.15 1.19 1.20 1.23 3.53 1.19 1.18 1.17
f1 (ppm)

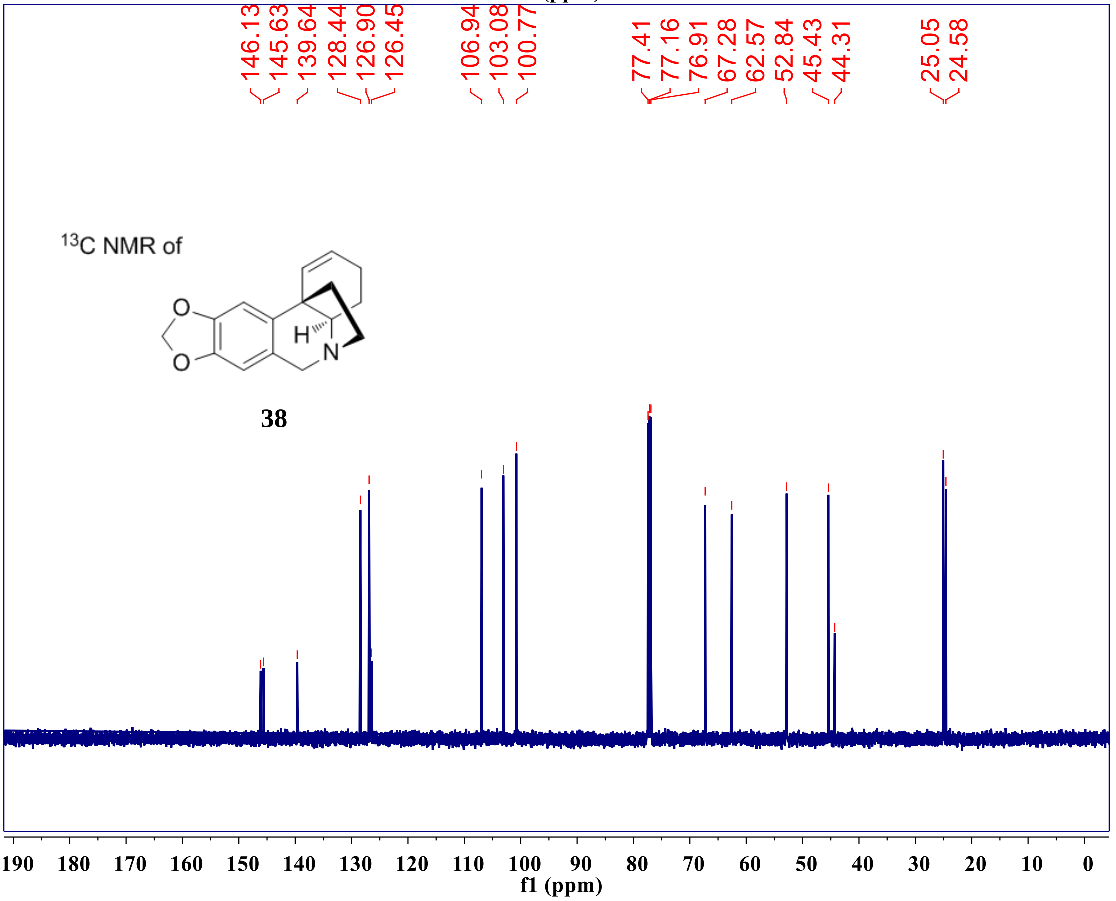
146.13 145.63 139.64 128.44 126.90 126.45 106.94 103.08 100.77 77.41 77.16 76.91 67.28 62.57 52.84 45.43 44.31 25.05 24.58
^{13}C NMR of
38
f1 (ppm)

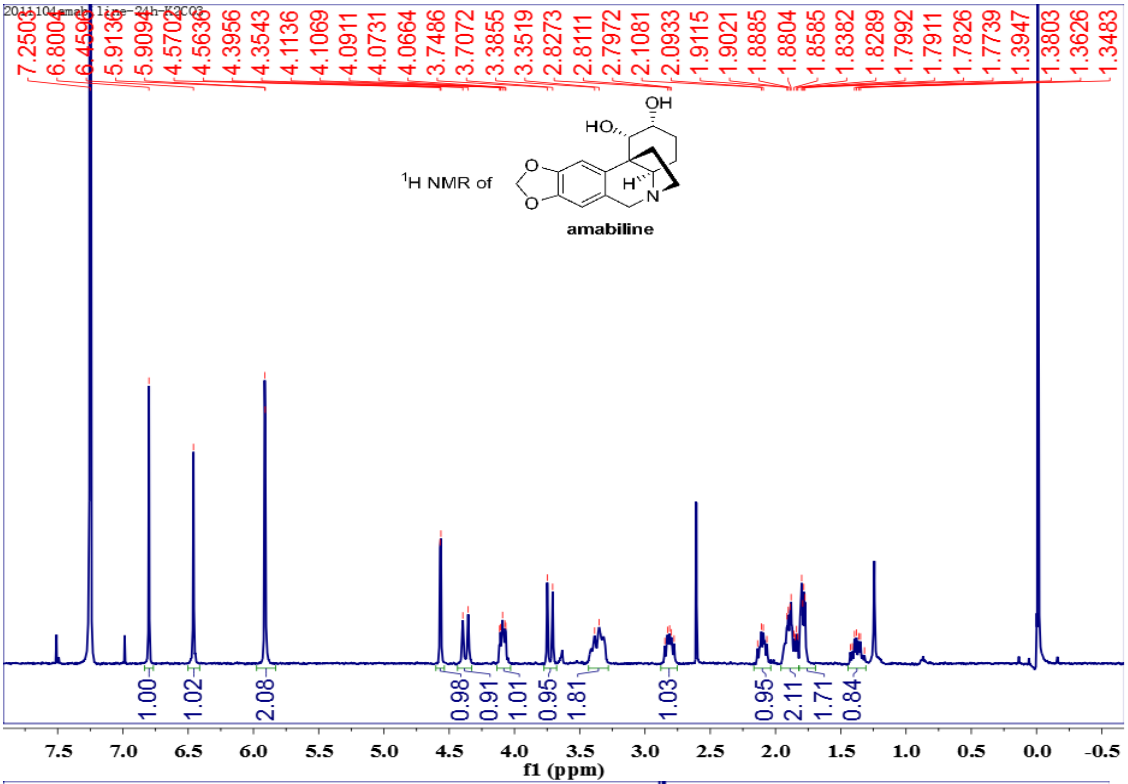

20111104amabiline-24h-K2CO3
7.2503
6.8004
6.4599
5.9136
5.9094
4.5702
4.5636
4.3956
4.3543
4.1136
4.1069
4.0911
4.0731
4.0664
3.7486
3.7072
3.3855
3.3519
2.8273
2.8111
2.7972
2.1081
2.0933
1.9115
1.9021
1.8885
1.8804
1.8585
1.8382
1.8289
1.7992
1.7911
1.7826
1.7739
1.3947
1.3803
1.3626
1.3483
OH
HO
O
O
H
N
¹H NMR of
amabiline
1.00
1.02
2.08
0.98
0.91
1.01
0.95
1.81
1.03
0.95
2.11
1.71
0.84
7.5
7.0
6.5
6.0
5.5
5.0
4.5
4.0
3.5
3.0
2.5
2.0
1.5
1.0
0.5
0.0
-0.5
f1 (ppm)

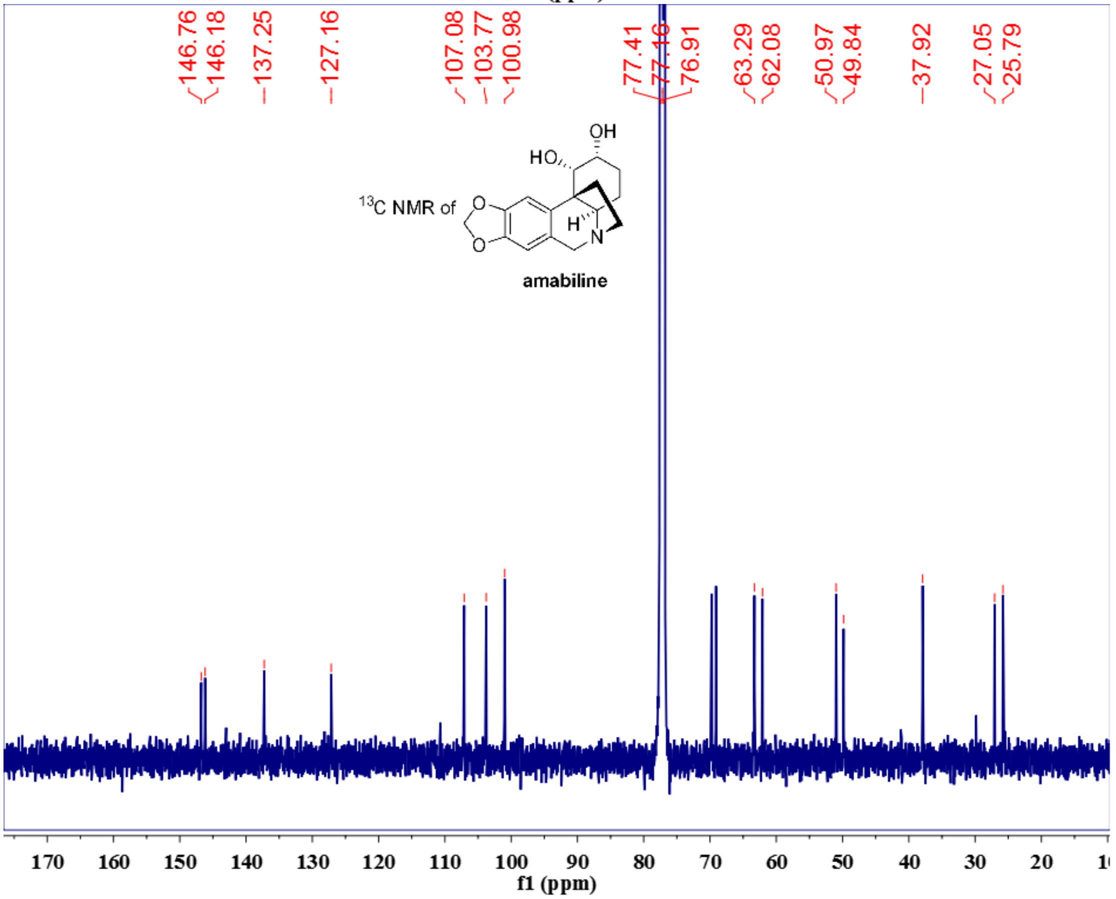

146.76
146.18
137.25
127.16
107.08
103.77
100.98
77.41
77.16
76.91
63.29
62.08
50.97
49.84
37.92
27.05
25.79
OH
HO
O
O
H
N
¹³C NMR of
amabiline
170
160
150
140
130
120
110
100
90
80
70
60
50
40
30
20
f1 (ppm)

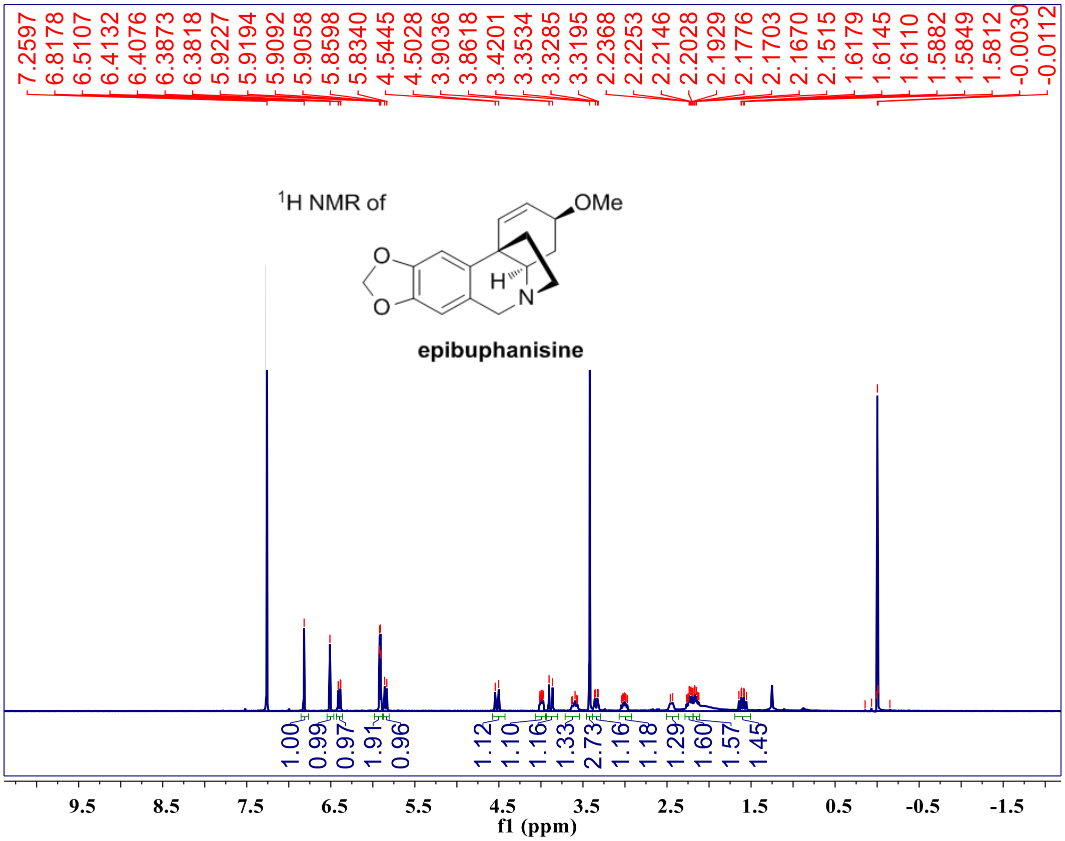

7.2597
6.8178
6.5107
6.4132
6.4076
6.3873
6.3818
5.9227
5.9194
5.9092
5.9058
5.8598
5.8340
4.5445
4.5028
3.9036
3.8618
3.4201
3.3534
3.3285
3.3195
2.2368
2.2253
2.2146
2.2028
2.1929
2.1776
2.1703
2.1670
2.1515
1.6179
1.6145
1.6110
1.5882
1.5849
1.5812
-0.0030
-0.0112
1H NMR of
OMe
O
O
H
N
epibuphanisine
1.00
0.99
0.97
1.91
0.96
1.12
1.10
1.16
1.33
2.73
1.16
1.18
1.29
1.60
1.57
1.45
9.5
8.5
7.5
6.5
5.5
4.5
3.5
2.5
1.5
0.5
-0.5
-1.5
f1 (ppm)

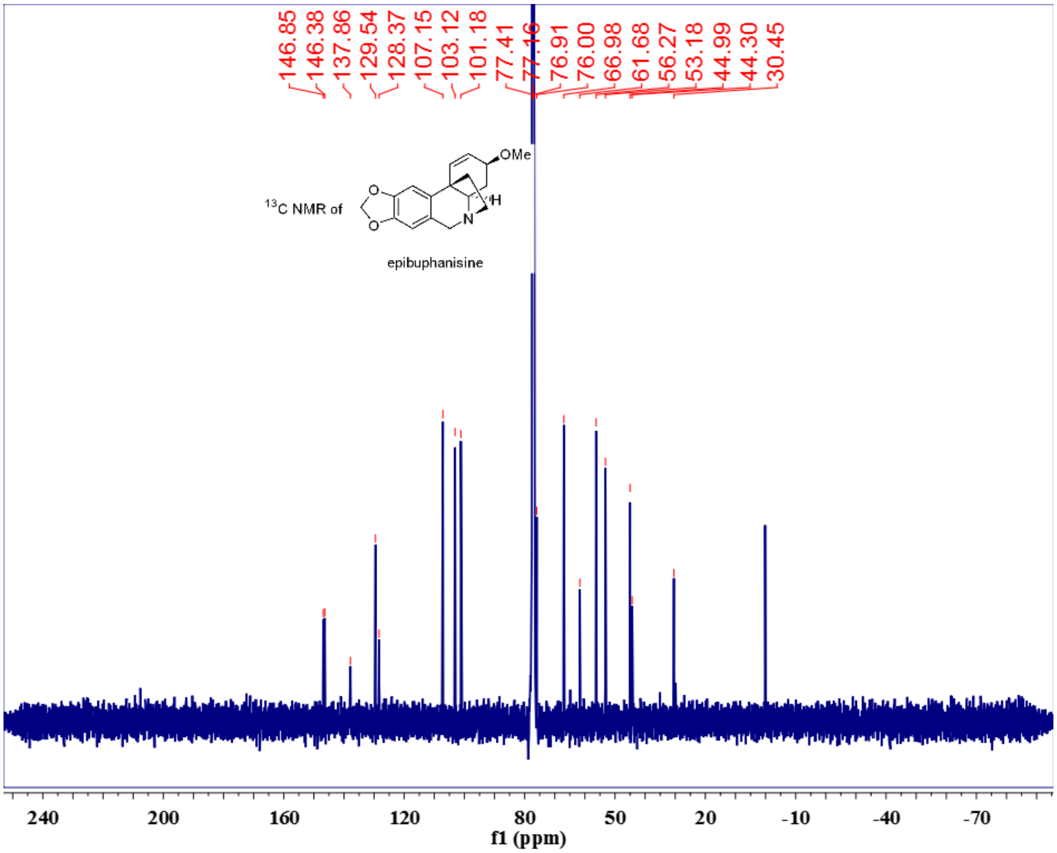
146.85
146.38
137.86
129.54
128.37
107.15
103.12
101.18
77.41
77.16
76.91
76.00
66.98
61.68
56.27
53.18
44.99
44.30
30.45
13C NMR of
OMe
H
N
O
O
epibuphanisine
240
200
160
120
80
50
20
-10
-40
-70
f1 (ppm)

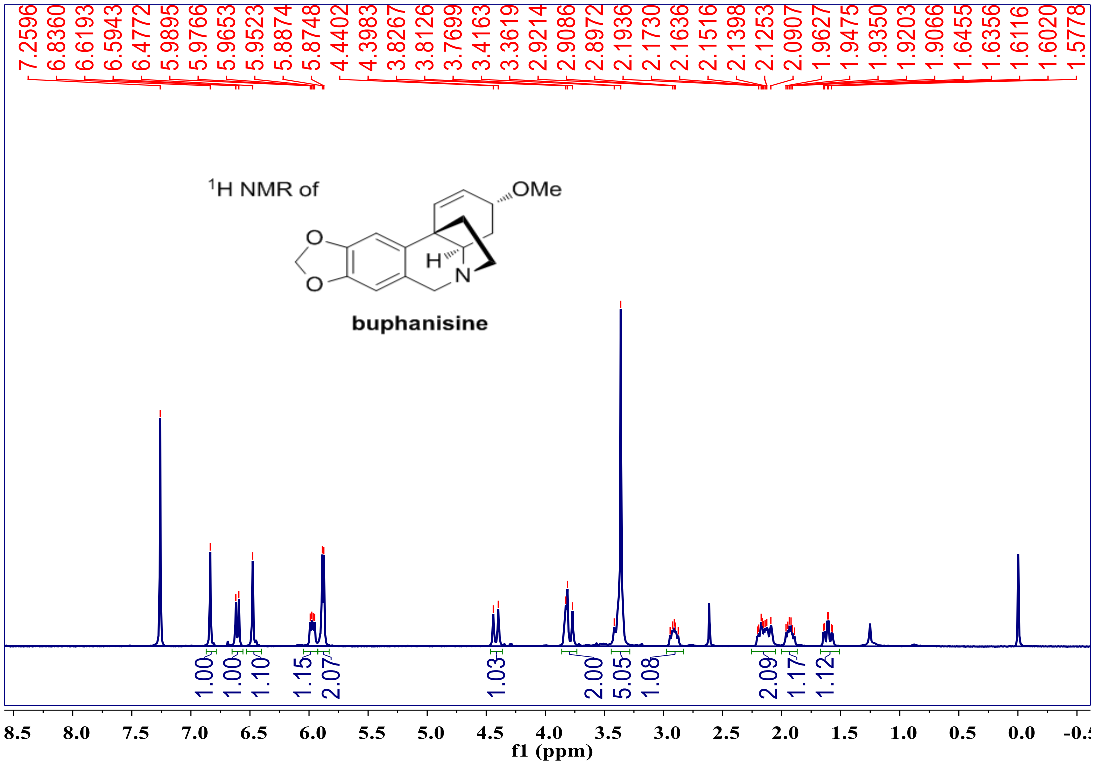
7.2596
6.8360
6.6193
6.5943
6.4772
5.9895
5.9766
5.9653
5.9523
5.8874
5.8748
4.4402
4.3983
3.8267
3.8126
3.7699
3.4163
3.3619
2.9214
2.9086
2.8972
2.1936
2.1730
2.1636
2.1516
2.1398
2.1253
2.0907
1.9627
1.9475
1.9350
1.9203
1.9066
1.6455
1.6356
1.6116
1.6020
1.5778
1H NMR of
OMe
H
N
O
O
buphanisine
1.00
1.00
1.10
1.15
2.07
1.03
2.00
5.05
1.08
2.09
1.17
1.12
8.5
8.0
7.5
7.0
6.5
6.0
5.5
5.0
4.5
4.0
3.5
3.0
2.5
2.0
1.5
1.0
0.5
0.0
f1 (ppm)

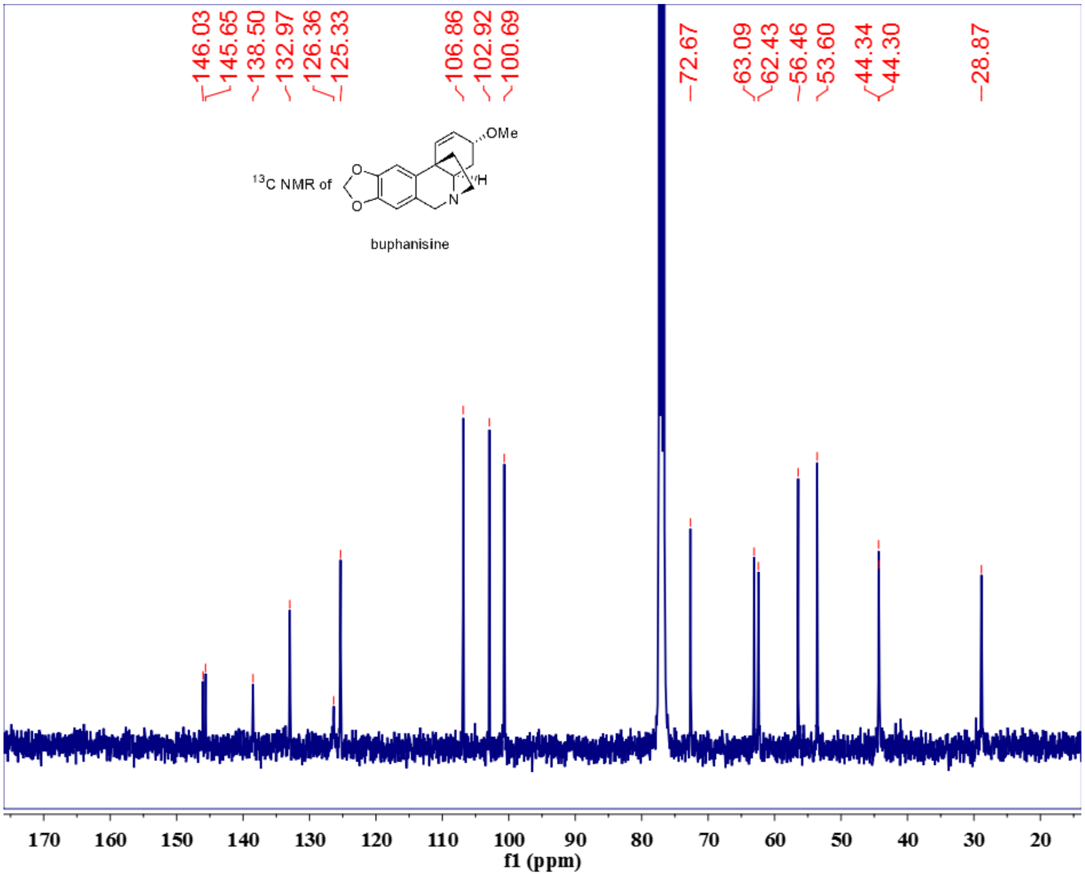
146.03
145.65
138.50
132.97
126.36
125.33
106.86
102.92
100.69
72.67
63.09
62.43
56.46
53.60
44.34
44.30
28.87
OMe
H
N
O
O
13C NMR of
buphanisine
170 160 150 140 130 120 110 100 90 80 70 60 50 40 30 20
f1 (ppm)

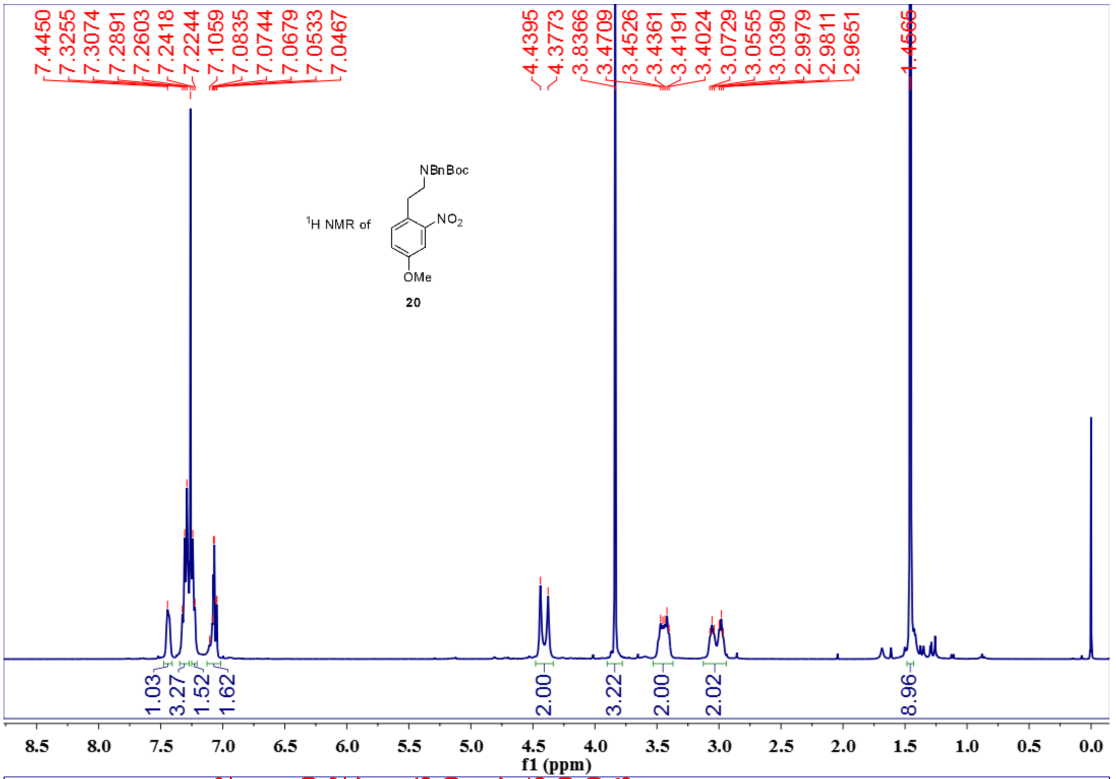

7.4450
7.3255
7.3074
7.2891
7.2603
7.2418
7.2244
7.1059
7.0835
7.0744
7.0679
7.0533
7.0467
4.4395
4.3773
3.8366
3.4709
3.4526
3.4361
3.4191
3.4024
3.0729
3.0555
3.0390
2.9979
2.9811
2.9651
1.4565
NBnBoc
^{1}H NMR of
NO_2
OMe
20
1.03
3.27
1.52
1.62
2.00
3.22
2.00
2.02
8.96
f1 (ppm)

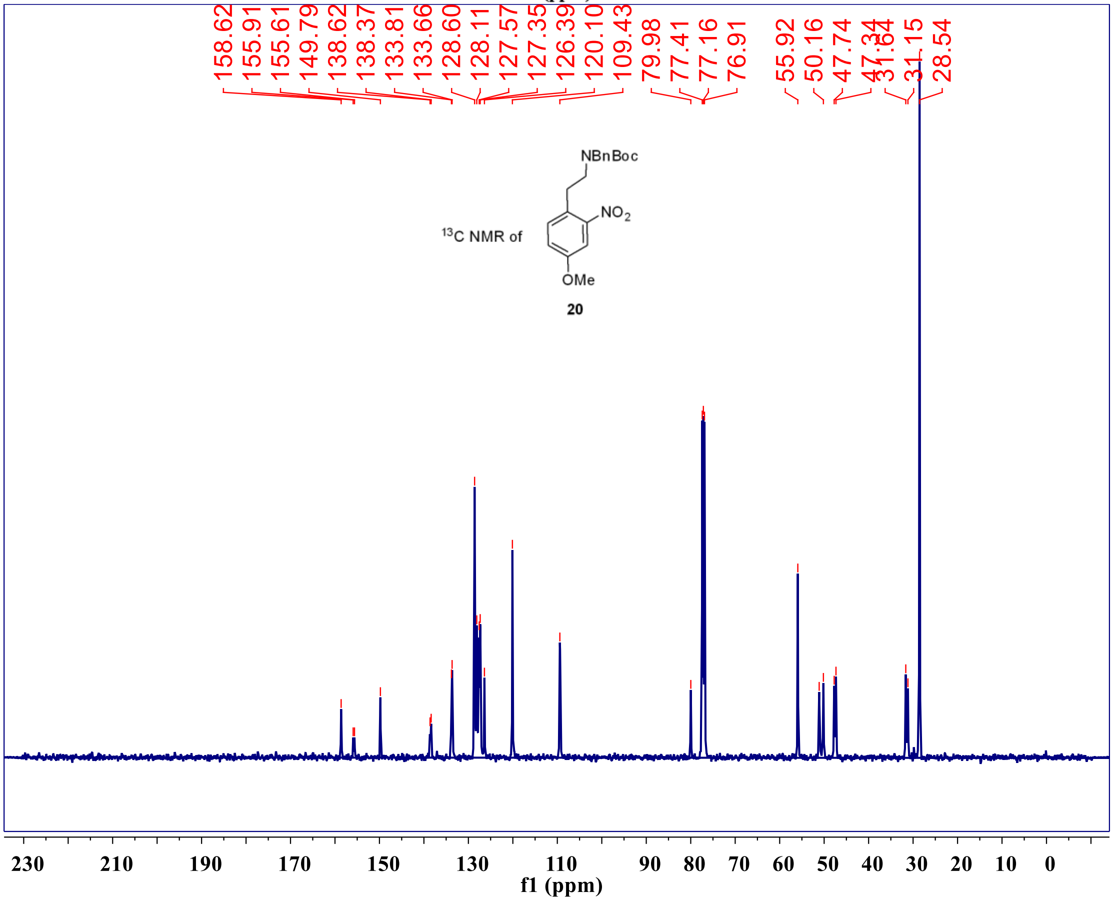

158.62
155.91
155.61
149.79
138.62
138.37
133.81
133.66
128.60
128.11
127.57
127.35
126.39
120.10
109.43
79.98
77.41
77.16
76.91
55.92
50.16
47.74
47.34
31.64
31.15
28.54
NBnBoc
^{13}C NMR of
NO_2
OMe
20
f1 (ppm)

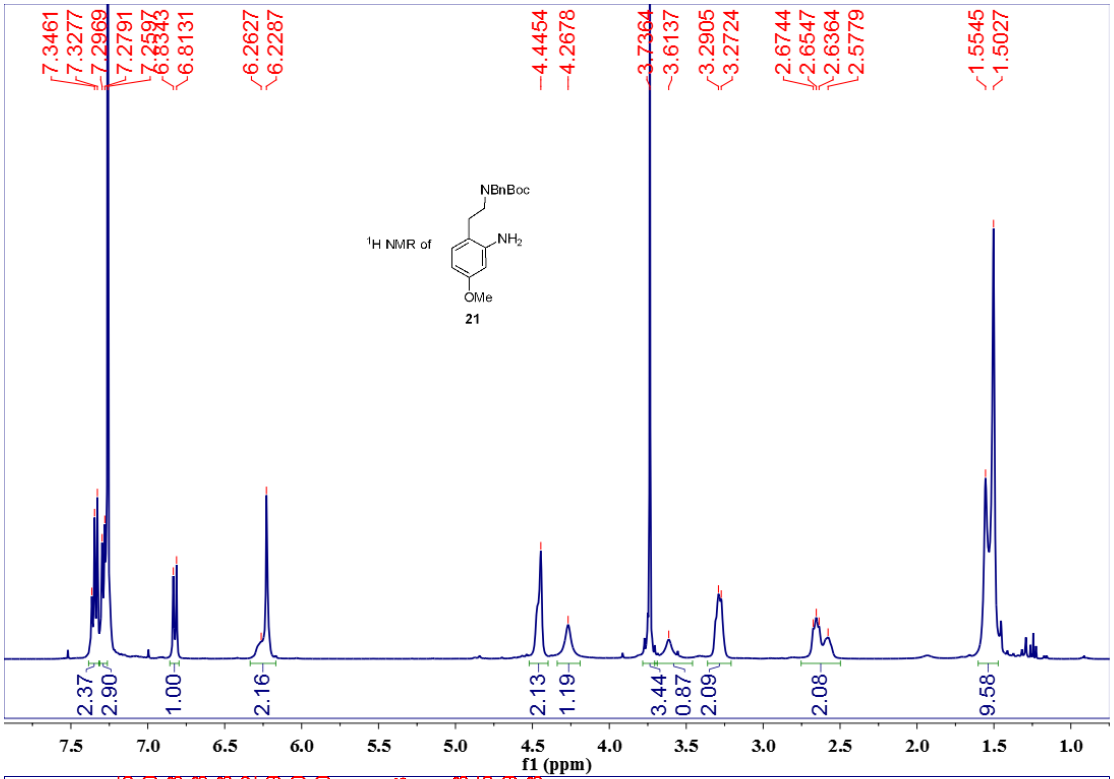

7.3461
7.3277
7.2969
7.2791
7.2597
6.8343
6.8131
6.2627
6.2287
4.4454
4.2678
3.7364
3.6137
3.2905
3.2724
2.6744
2.6547
2.6364
2.5779
1.5545
1.5027
^{1}H NMR of
NBnBoc
NH_2
OMe
21
2.37
2.90
1.00
2.16
2.13
1.19
3.44
0.87
2.09
2.08
9.58
7.5
7.0
6.5
6.0
5.5
5.0
4.5
4.0
3.5
3.0
2.5
2.0
1.5
1.0
f1 (ppm)

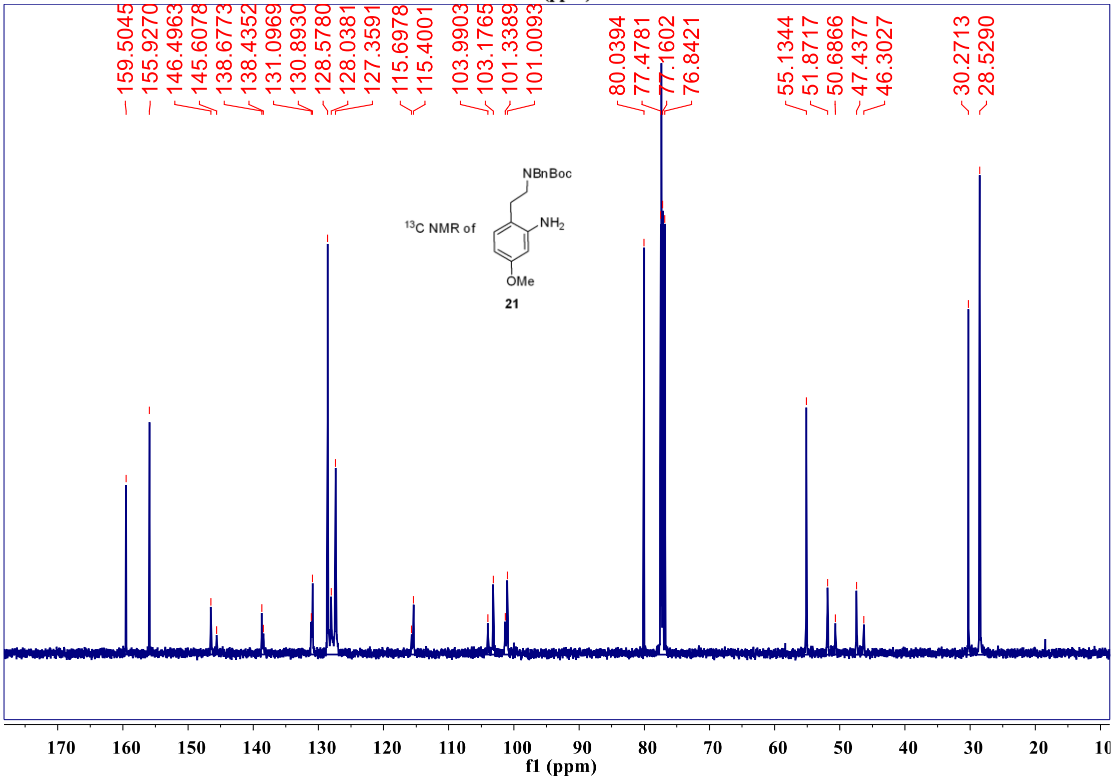

159.5045
155.9270
146.4963
145.6078
138.6773
138.4352
131.0969
130.8930
128.5780
128.0381
127.3591
115.6978
115.4001
103.9903
103.1765
101.3389
101.0093
80.0394
77.4781
77.1602
76.8421
55.1344
51.8717
50.6866
47.4377
46.3027
30.2713
28.5290
^{13}C NMR of
NBnBoc
NH_2
OMe
21
170
160
150
140
130
120
110
100
90
80
70
60
50
40
30
20
f1 (ppm)

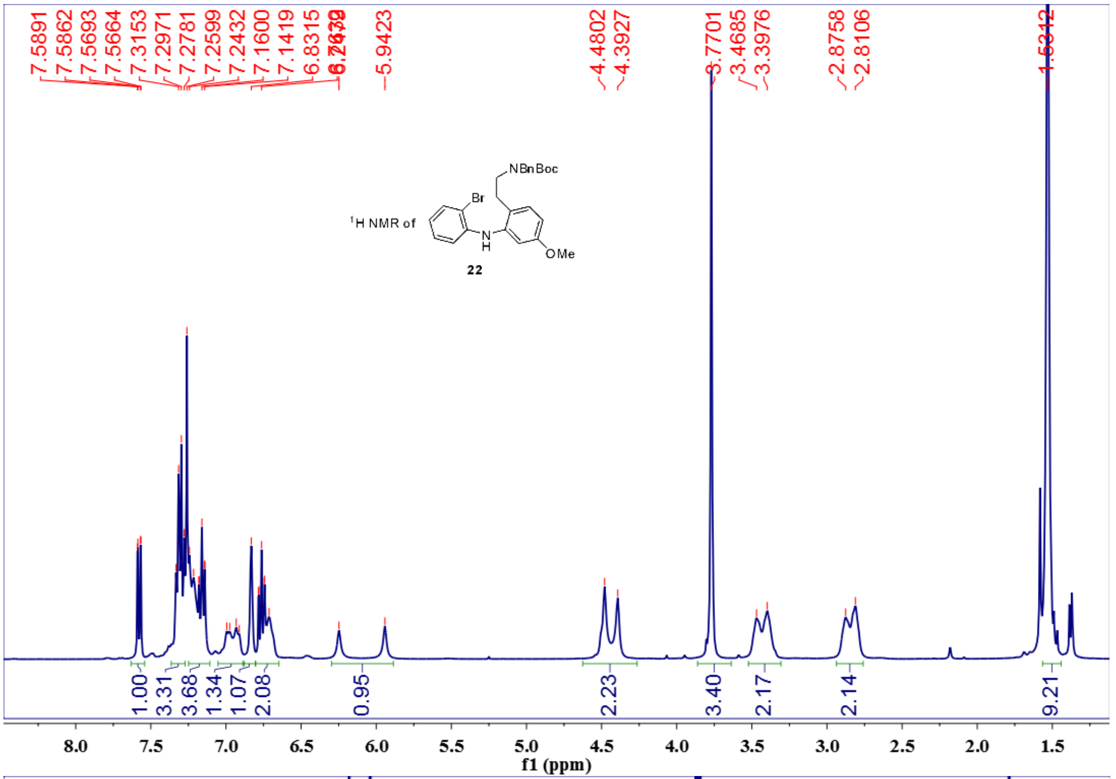

1H NMR of
NBnBoc
Br
N
H
OMe
22
f1 (ppm)

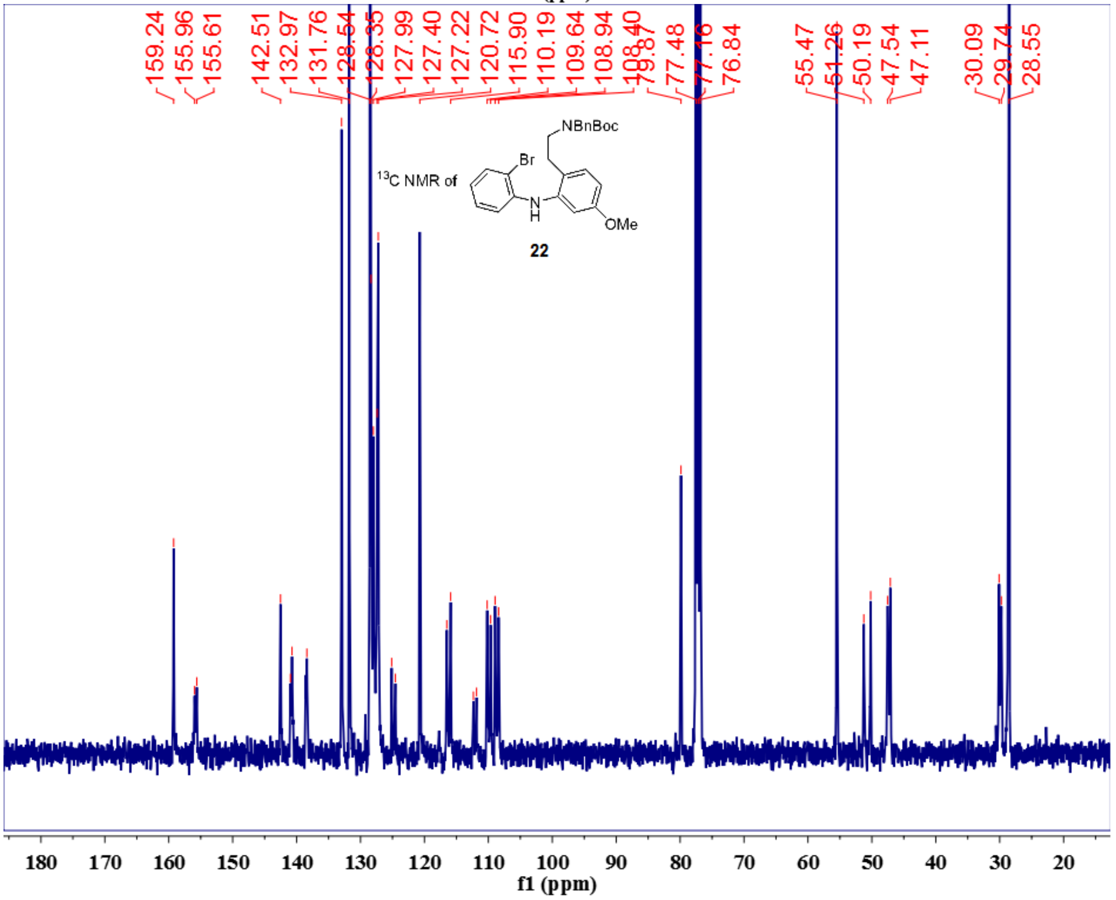

13C NMR of
NBnBoc
Br
N
H
OMe
22
f1 (ppm)

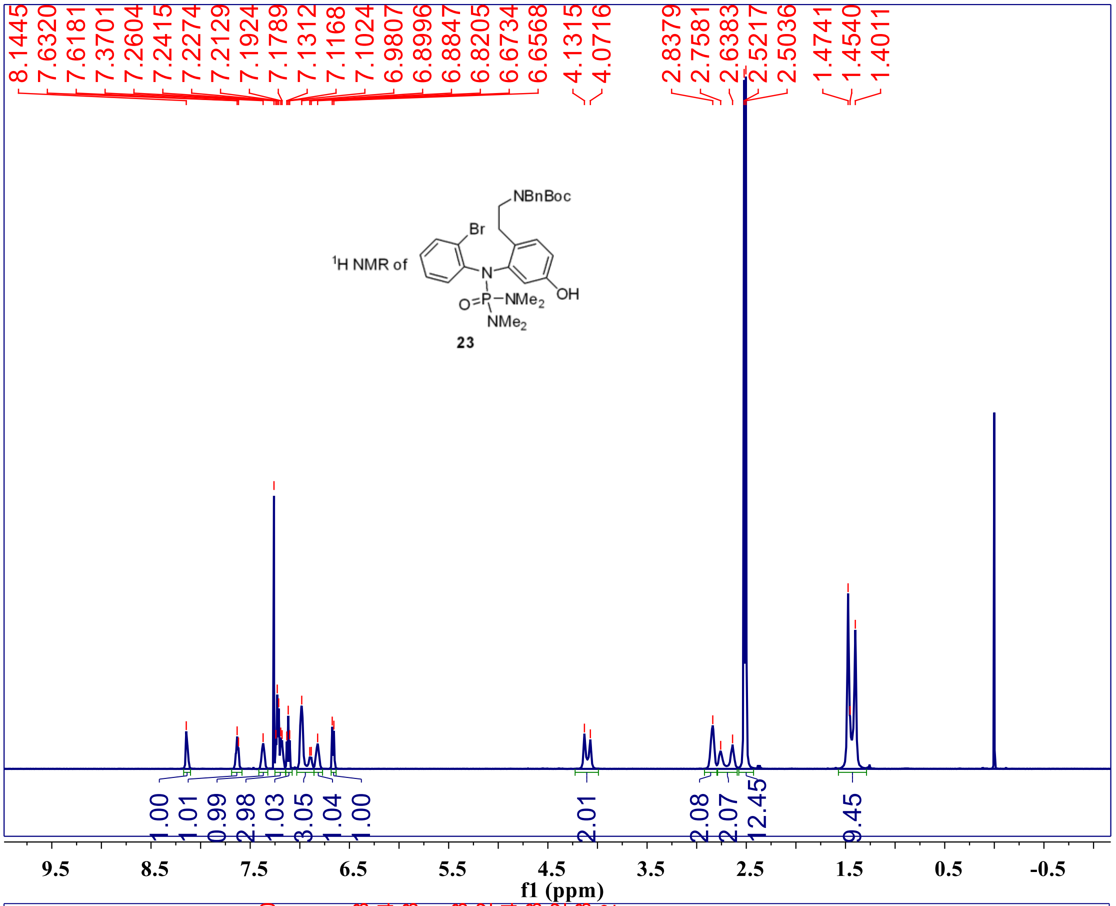

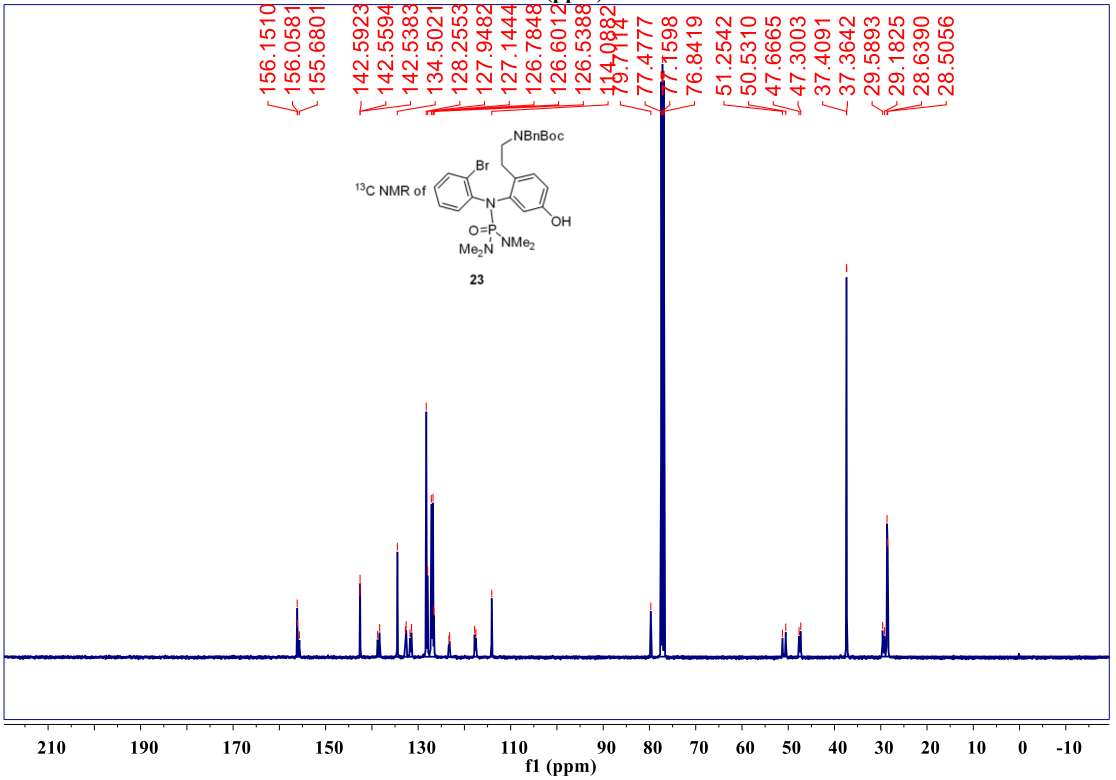

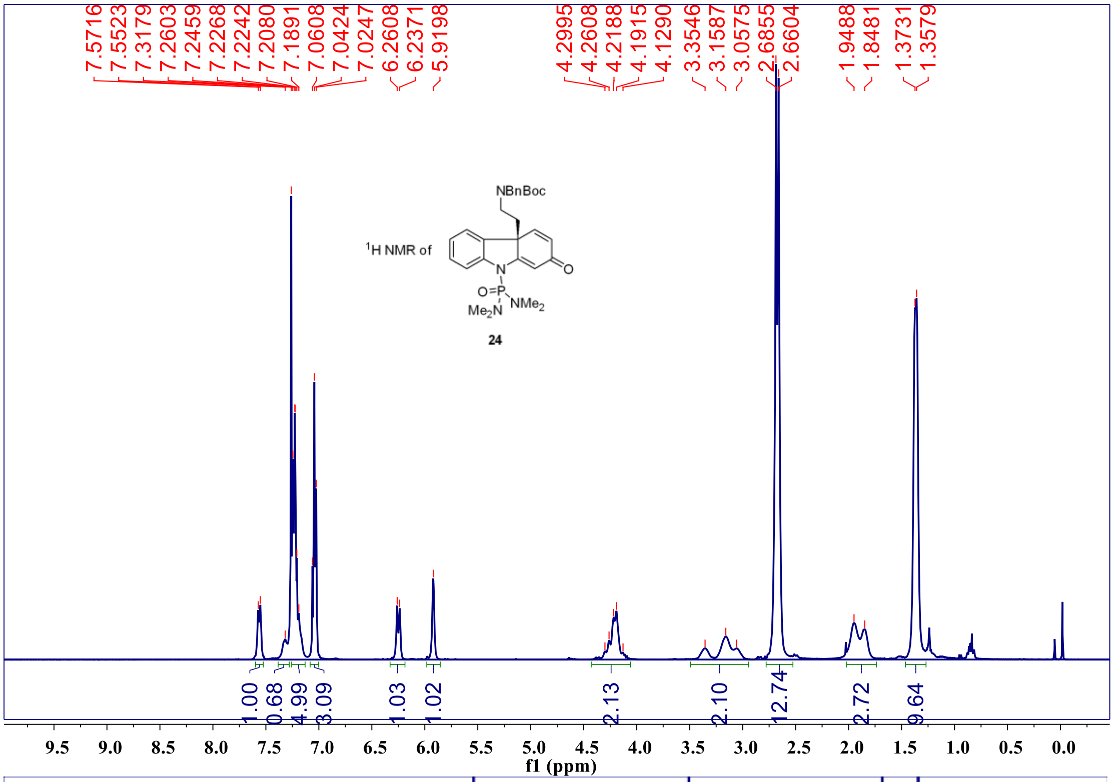

7.5716
7.5523
7.3179
7.2603
7.2459
7.2268
7.2242
7.2080
7.1891
7.0608
7.0424
7.0247
6.2608
6.2371
5.9198
4.2995
4.2608
4.2188
4.1915
4.1290
3.3546
3.1587
3.0575
2.6855
2.6604
1.9488
1.8481
1.3731
1.3579
NBnBoc
^{1}H NMR of
O
N
O=P
NMe$_2$
Me$_2$N
24
1.00
0.68
4.99
3.09
1.03
1.02
2.13
2.10
12.74
2.72
9.64
9.5 9.0 8.5 8.0 7.5 7.0 6.5 6.0 5.5 5.0 4.5 4.0 3.5 3.0 2.5 2.0 1.5 1.0 0.5 0.0
f1 (ppm)

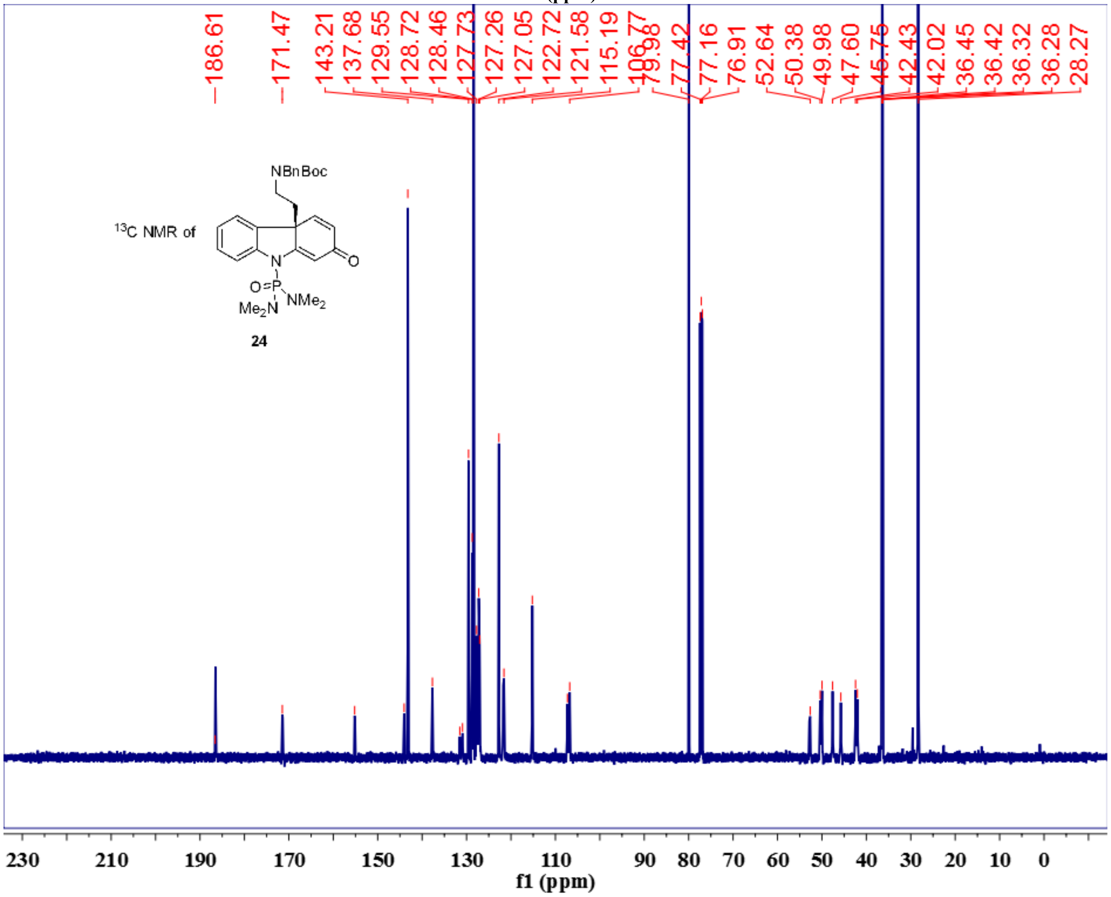

186.61
171.47
143.21
137.68
129.55
128.72
128.46
127.73
127.26
127.05
122.72
121.58
115.19
106.77
79.98
77.42
77.16
76.91
52.64
50.38
49.98
47.60
45.75
42.43
42.02
36.45
36.42
36.32
36.28
28.27
^{13}C NMR of
NBnBoc
O
N
O=P
NMe$_2$
Me$_2$N
24
230 210 190 170 150 130 110 90 80 70 60 50 40 30 20 10 0
f1 (ppm)

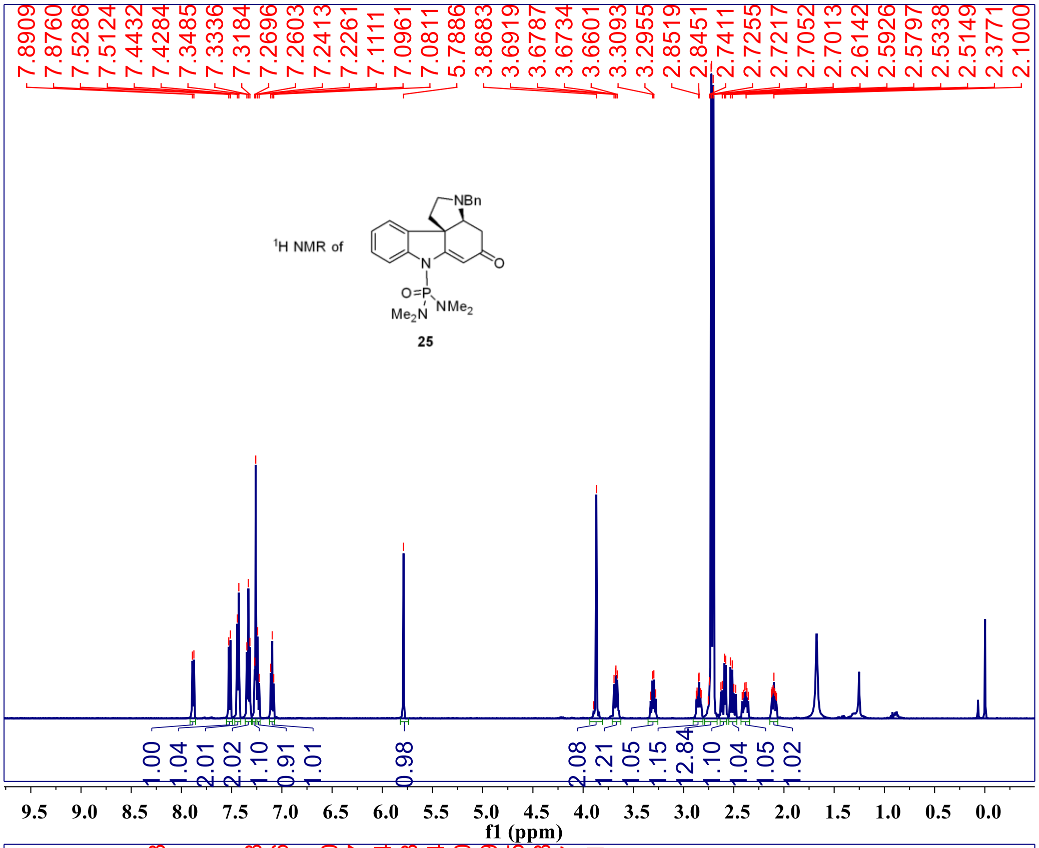

7.8909
7.8760
7.5286
7.5124
7.4432
7.4284
7.3485
7.3336
7.3184
7.2696
7.2603
7.2413
7.2261
7.1111
7.0961
7.0811
5.7886
3.8683
3.6919
3.6787
3.6734
3.6601
3.3093
3.2955
2.8519
2.8451
2.7411
2.7255
2.7217
2.7052
2.7013
2.6142
2.5926
2.5797
2.5338
2.5149
2.3771
2.1000
^{1}H NMR of
NBn
N
O=P
NMe$_2$
Me$_2$N
25
1.00
1.04
2.01
2.02
1.10
0.91
1.01
0.98
2.08
1.21
1.05
1.15
12.84
1.10
1.04
1.05
1.02
9.5 9.0 8.5 8.0 7.5 7.0 6.5 6.0 5.5 5.0 4.5 4.0 3.5 3.0 2.5 2.0 1.5 1.0 0.5 0.0
f1 (ppm)

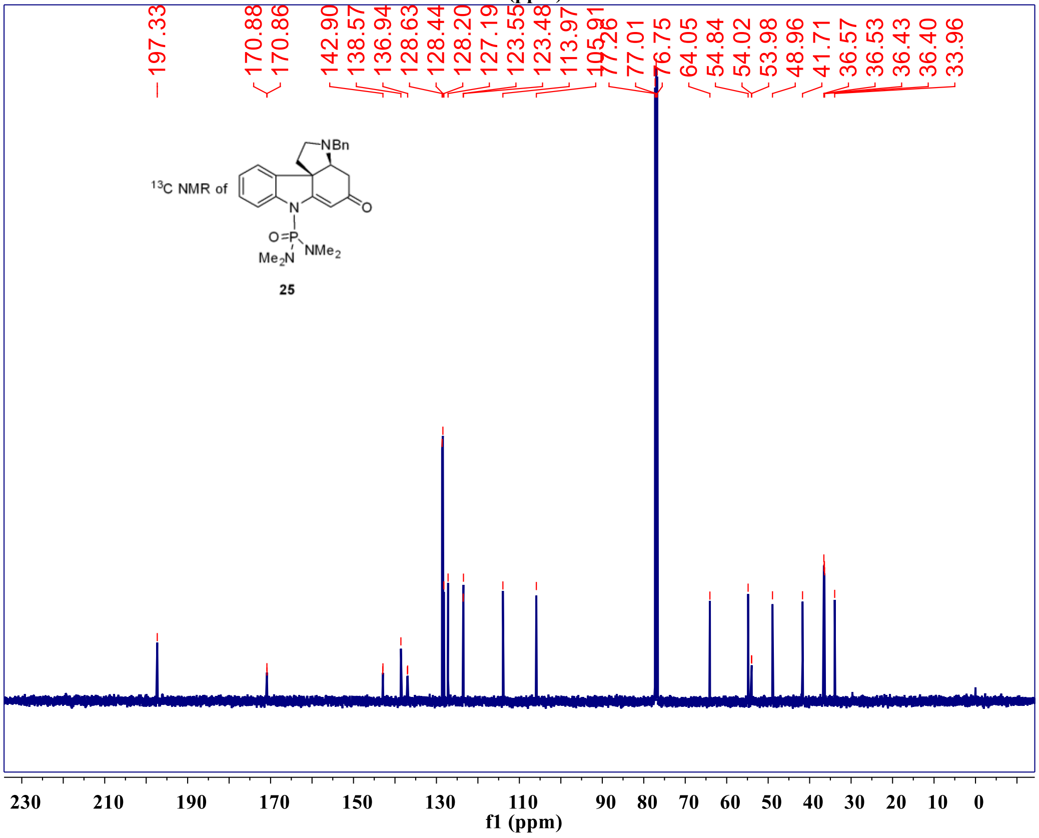

197.33
170.88
170.86
142.90
138.57
136.94
128.63
128.44
128.20
127.19
123.55
123.48
113.97
105.91
77.26
77.01
76.75
64.05
54.84
54.02
53.98
48.96
41.71
36.57
36.53
36.43
36.40
33.96
^{13}C NMR of
NBn
N
O=P
NMe$_2$
Me$_2$N
25
230 210 190 170 150 130 110 90 80 70 60 50 40 30 20 10 0
f1 (ppm)

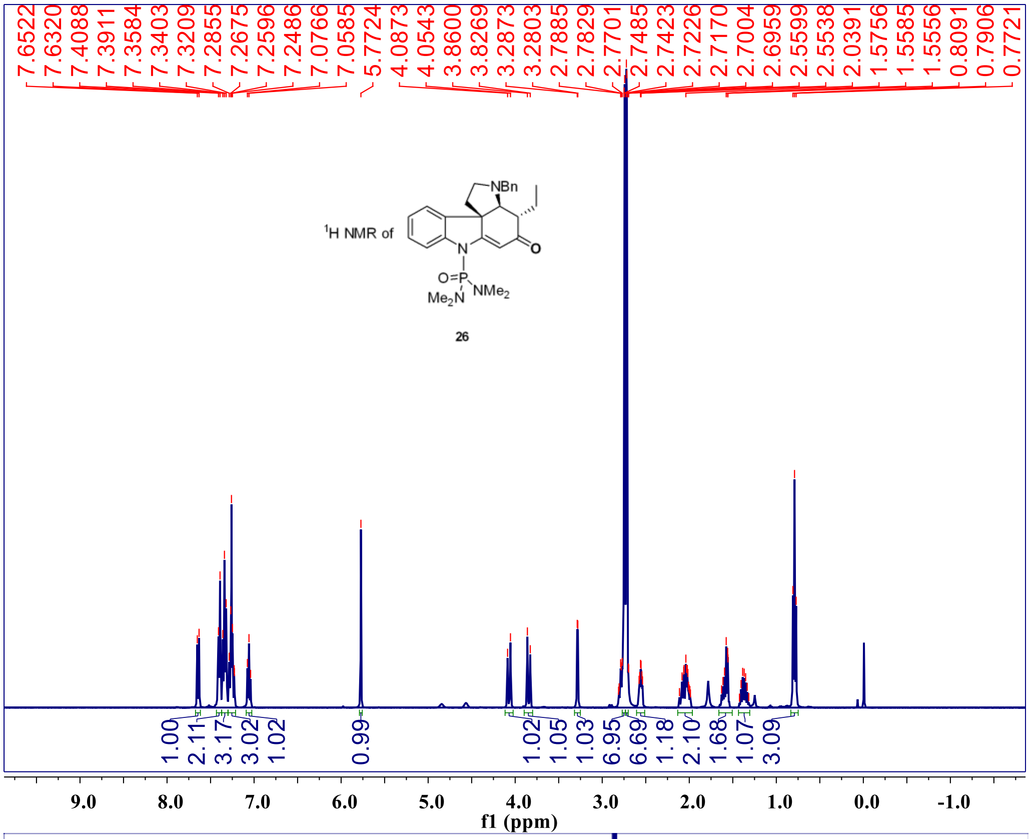
7.6522
7.6320
7.4088
7.3911
7.3584
7.3403
7.3209
7.2855
7.2675
7.2596
7.2486
7.0766
7.0585
5.7724
4.0873
4.0543
3.8600
3.8269
3.2873
3.2803
2.7885
2.7829
2.7701
2.7485
2.7423
2.7226
2.7170
2.7004
2.6959
2.5599
2.5538
2.0391
1.5756
1.5585
1.5556
0.8091
0.7906
0.7721
1H NMR of
NBn
N
O
O=P
NMe2
Me2N
26
1.00
2.11
3.17
3.02
1.02
0.99
1.02
1.05
1.03
6.95
6.69
1.18
2.10
1.68
1.07
3.09
9.0
8.0
7.0
6.0
5.0
4.0
3.0
2.0
1.0
0.0
-1.0
f1 (ppm)

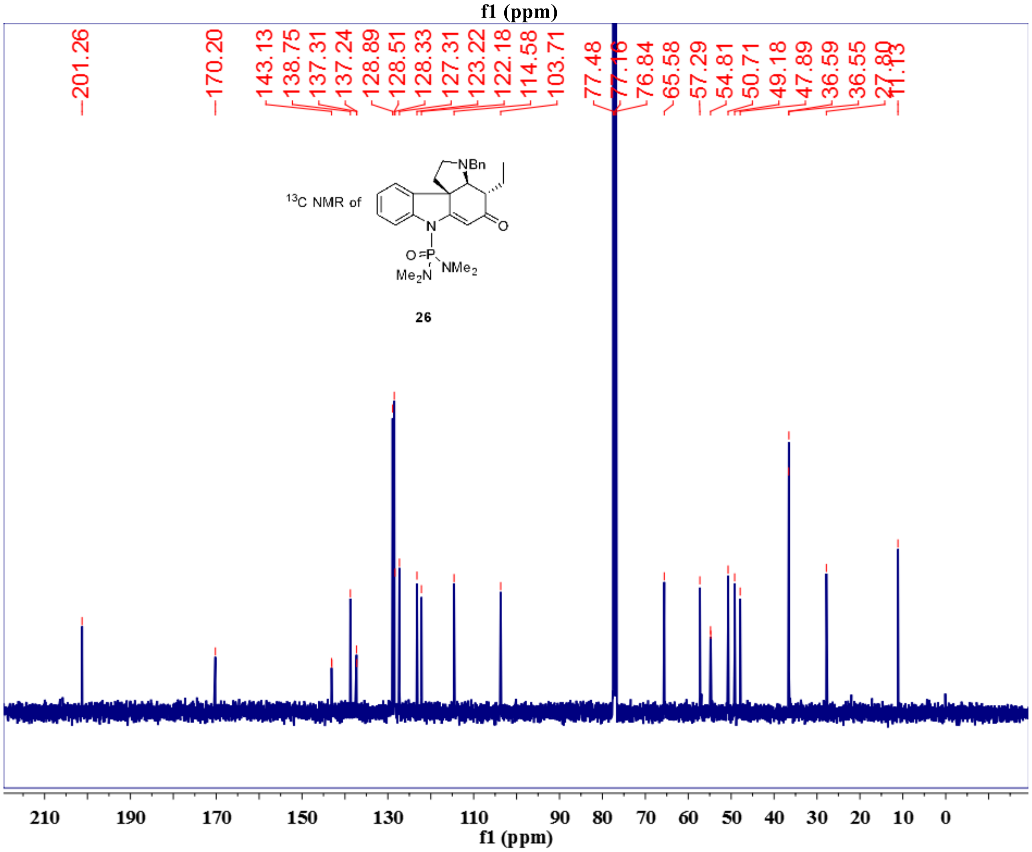
201.26
170.20
143.13
138.75
137.31
137.24
128.89
128.51
128.33
127.31
123.22
122.18
114.58
103.71
77.48
77.16
76.84
65.58
57.29
54.81
50.71
49.18
47.89
36.59
36.55
27.80
11.13
13C NMR of
NBn
N
O
O=P
NMe2
Me2N
26
210
190
170
150
130
110
90
80
70
60
50
40
30
20
10
0
f1 (ppm)

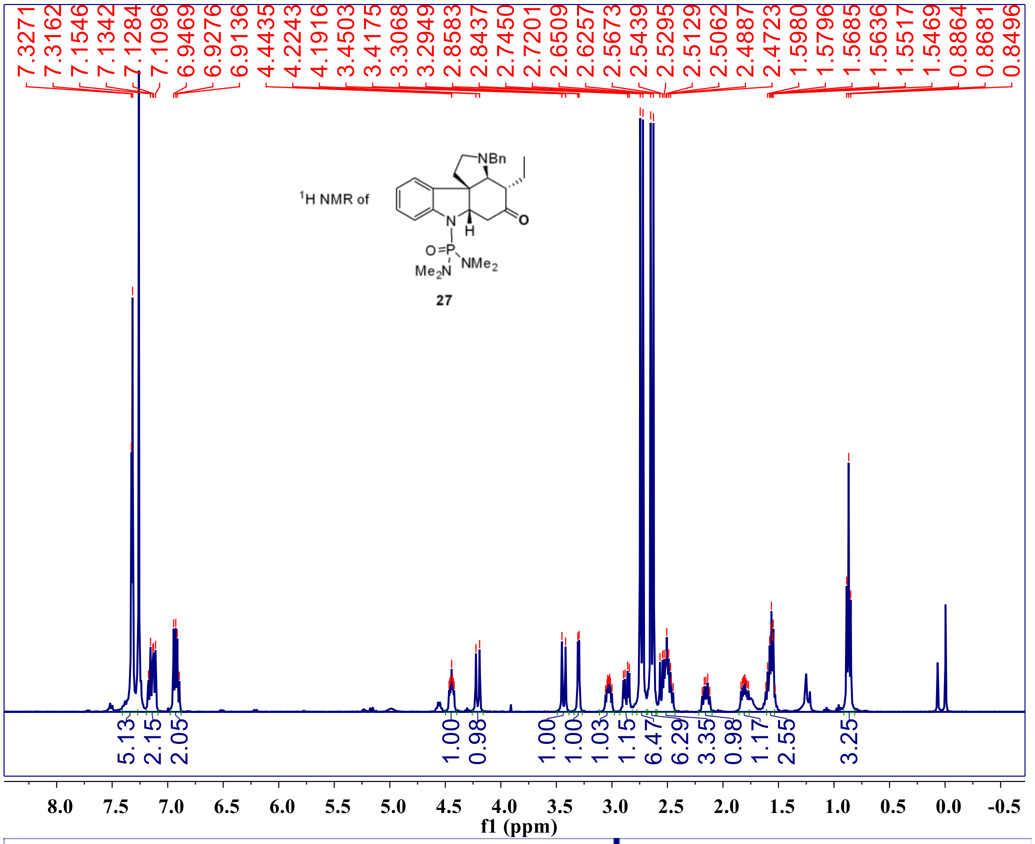
7.3271
7.3162
7.1546
7.1342
7.1284
7.1096
6.9469
6.9276
6.9136
4.4435
4.2243
4.1916
3.4503
3.4175
3.3068
3.2949
2.8583
2.8437
2.7450
2.7201
2.6509
2.6257
2.5673
2.5439
2.5295
2.5129
2.5062
2.4887
2.4723
1.5980
1.5796
1.5685
1.5636
1.5517
1.5469
0.8864
0.8681
0.8496
^{1}H NMR of
NBn
N
H
O
O=P
NMe$_2$
Me$_2$N
27
5.13
2.15
2.05
1.00
0.98
1.00
1.00
1.03
1.15
6.47
6.29
3.35
0.98
1.17
2.55
3.25
8.0
7.5
7.0
6.5
6.0
5.5
5.0
4.5
4.0
3.5
3.0
2.5
2.0
1.5
1.0
0.5
0.0
-0.5
f1 (ppm)

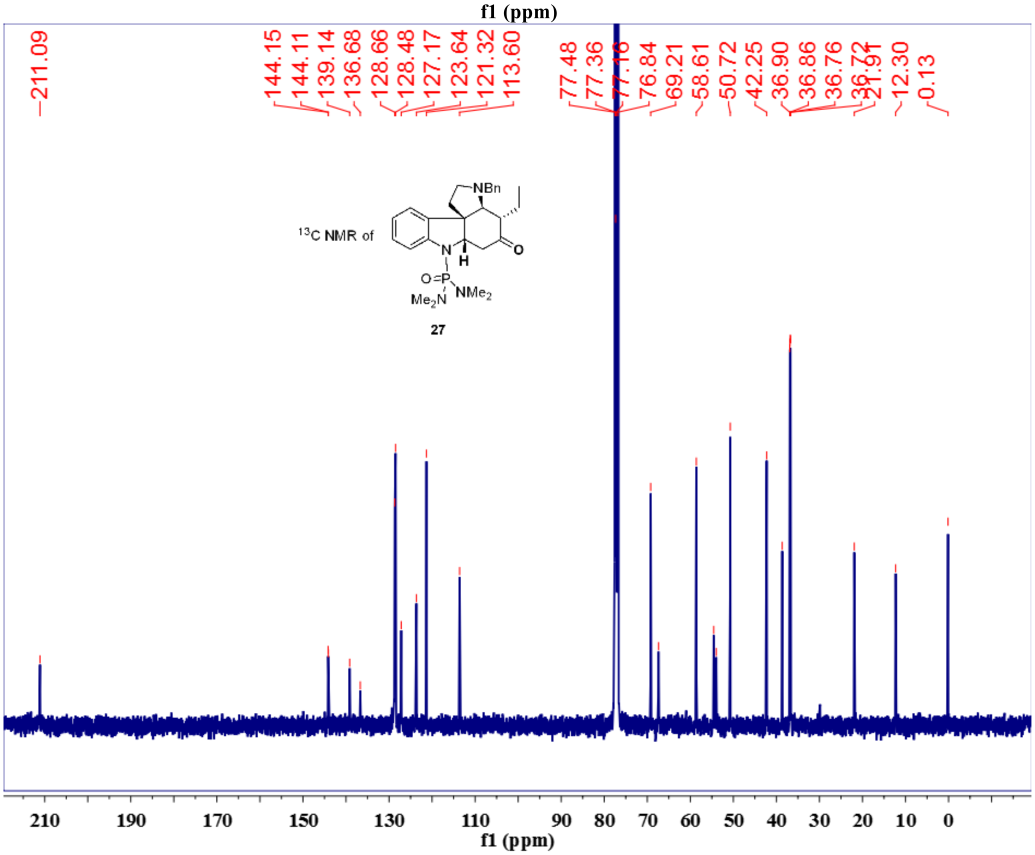
211.09
144.15
144.11
139.14
136.68
128.66
128.48
127.17
123.64
121.32
113.60
77.48
77.36
77.16
76.84
69.21
58.61
50.72
42.25
36.90
36.86
36.76
36.72
21.91
12.30
0.13
^{13}C NMR of
NBn
N
H
O
O=P
NMe$_2$
Me$_2$N
27
210
190
170
150
130
110
90
80
70
60
50
40
30
20
10
0
f1 (ppm)

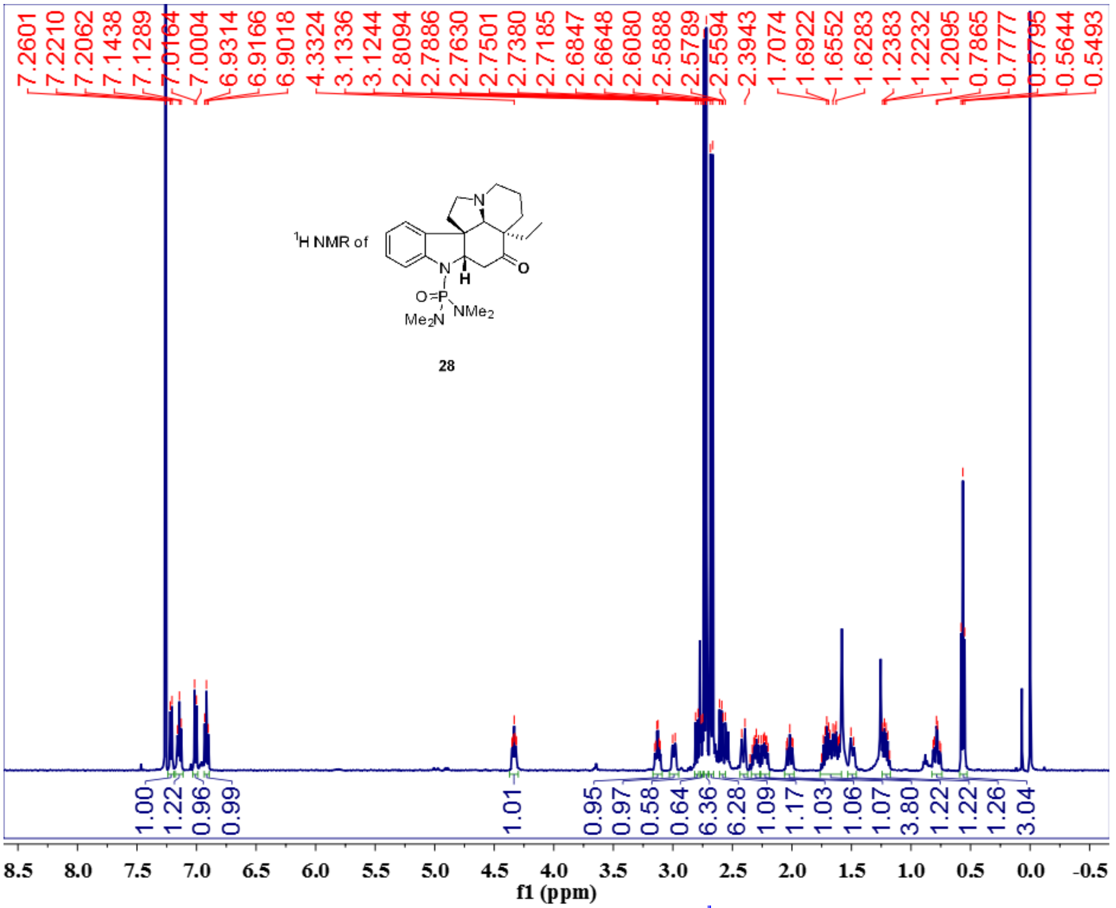
7.2601
7.2210
7.2062
7.1438
7.1289
7.0164
7.0004
6.9314
6.9166
6.9018
4.3324
3.1336
3.1244
2.8094
2.7886
2.7630
2.7501
2.7380
2.7185
2.6847
2.6648
2.6080
2.5888
2.5789
2.5594
2.3943
1.7074
1.6922
1.6552
1.6283
1.2383
1.2232
1.2095
0.7865
0.7777
0.5795
0.5644
0.5493
1H NMR of
28
1.00
1.22
0.96
0.99
1.01
0.95
0.97
0.58
0.64
6.36
6.28
1.09
1.17
1.03
1.06
1.07
3.80
1.22
1.22
1.26
3.04
f1 (ppm)

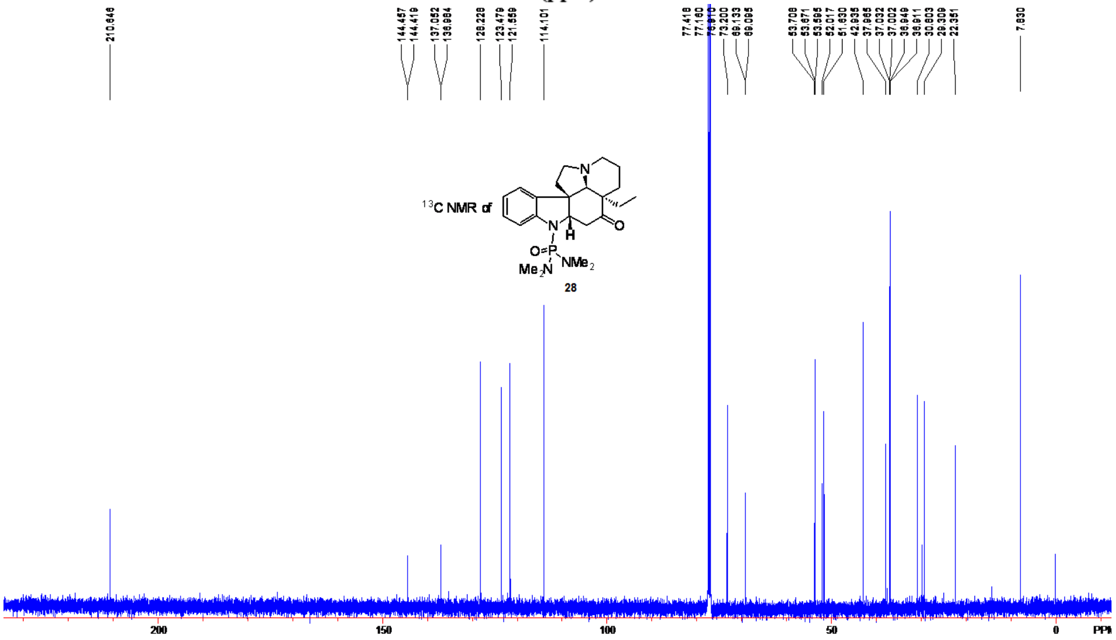
13C NMR of
28

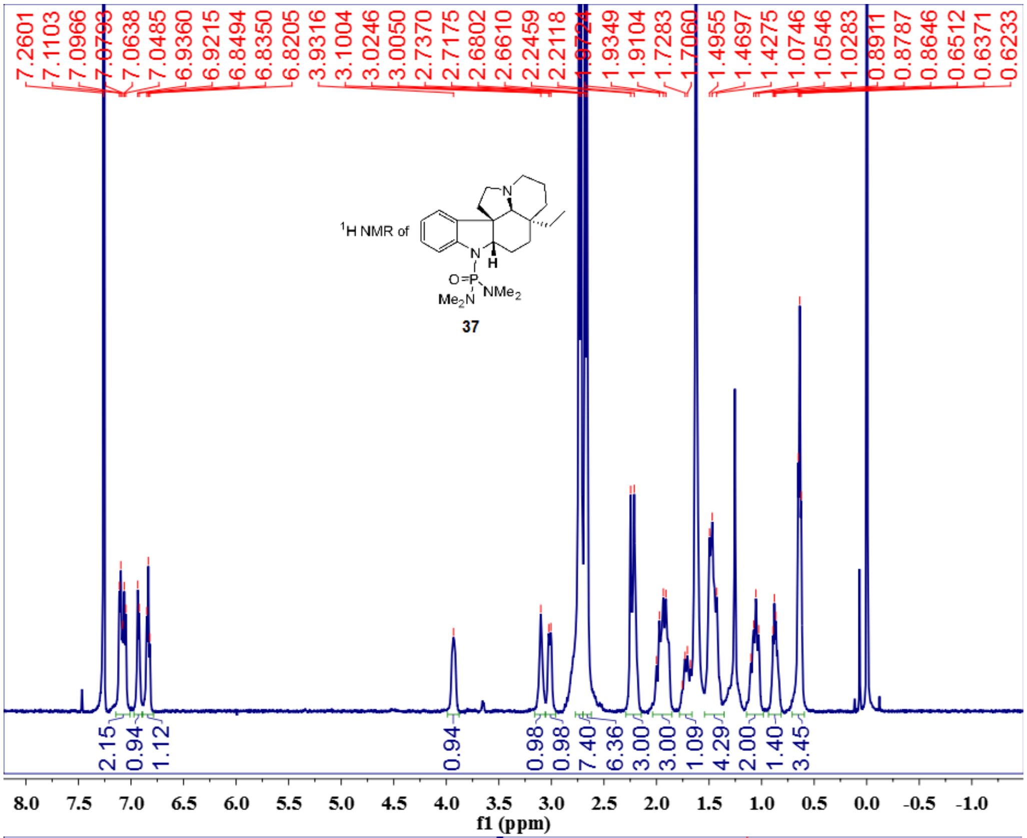
7.2601
7.1103
7.0966
7.0793
7.0638
7.0485
6.9360
6.9215
6.8494
6.8350
6.8205
3.9316
3.1004
3.0246
3.0050
2.7370
2.7175
2.6802
2.6610
2.2459
2.2118
1.9724
1.9349
1.9104
1.7283
1.7060
1.4955
1.4697
1.4275
1.0746
1.0546
1.0283
0.8911
0.8787
0.8646
0.6512
0.6371
0.6233
¹H NMR of
NMe₂
Me₂N
37
2.15
0.94
1.12
0.94
0.98
0.98
7.40
6.36
3.00
3.00
1.09
4.29
2.00
1.40
3.45
f1 (ppm)

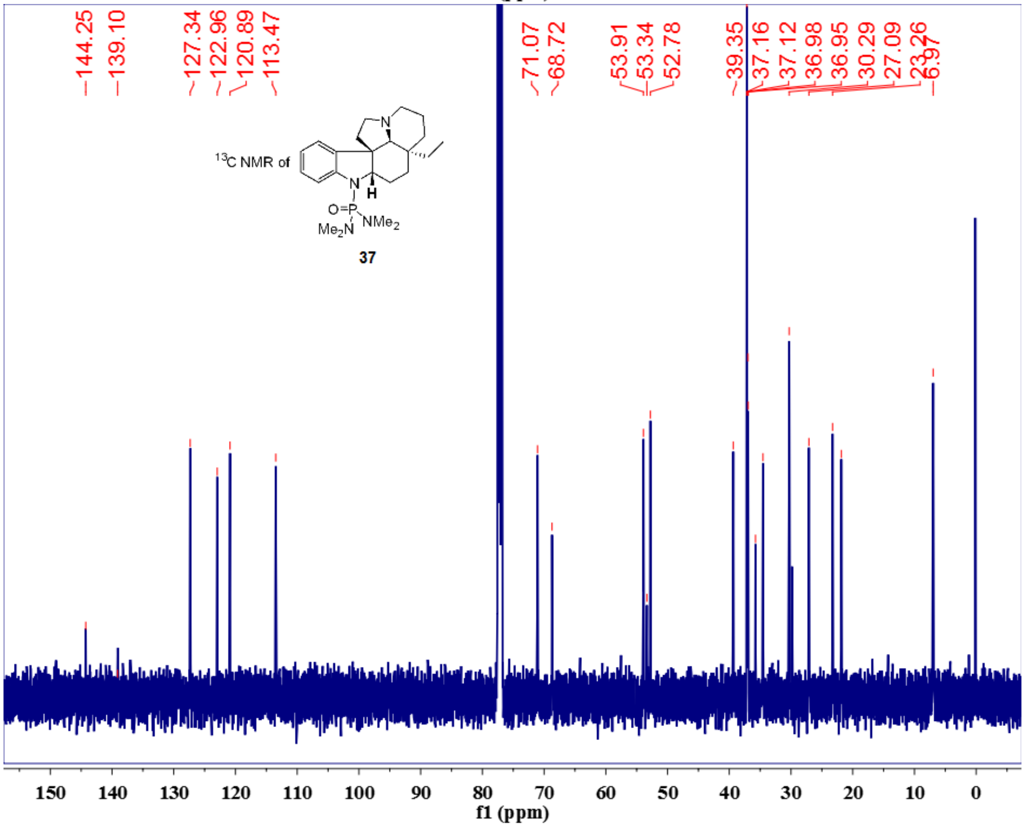
144.25
139.10
127.34
122.96
120.89
113.47
71.07
68.72
53.91
53.34
52.78
39.35
37.16
37.12
36.98
36.95
30.29
27.09
23.26
6.97
¹³C NMR of
NMe₂
Me₂N
37
f1 (ppm)

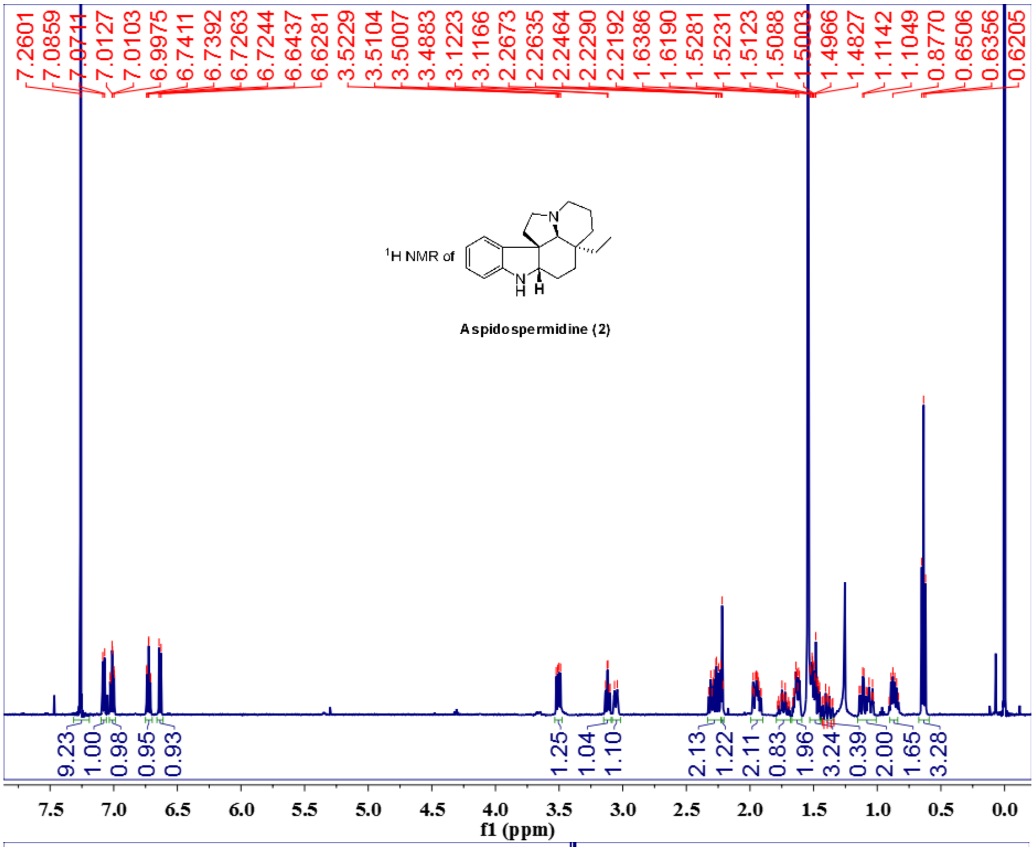

^{1}H NMR of
Aspidospermidine (2)
f1 (ppm)

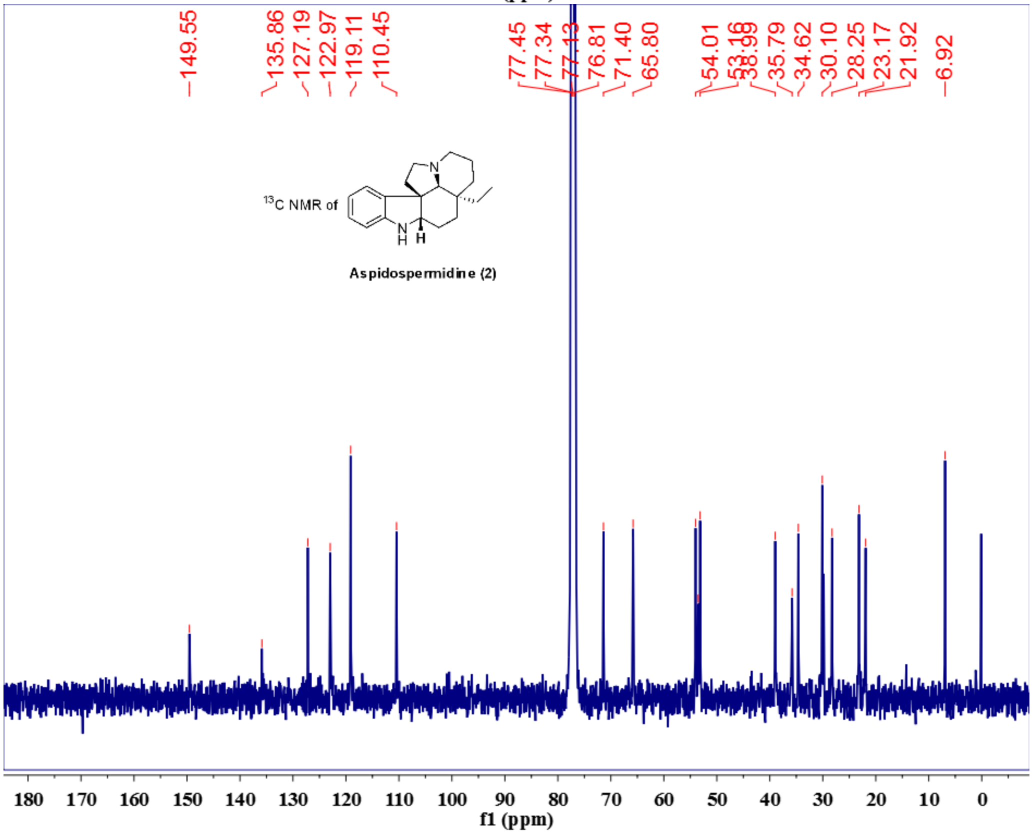

^{13}C NMR of
Aspidospermidine (2)
f1 (ppm)

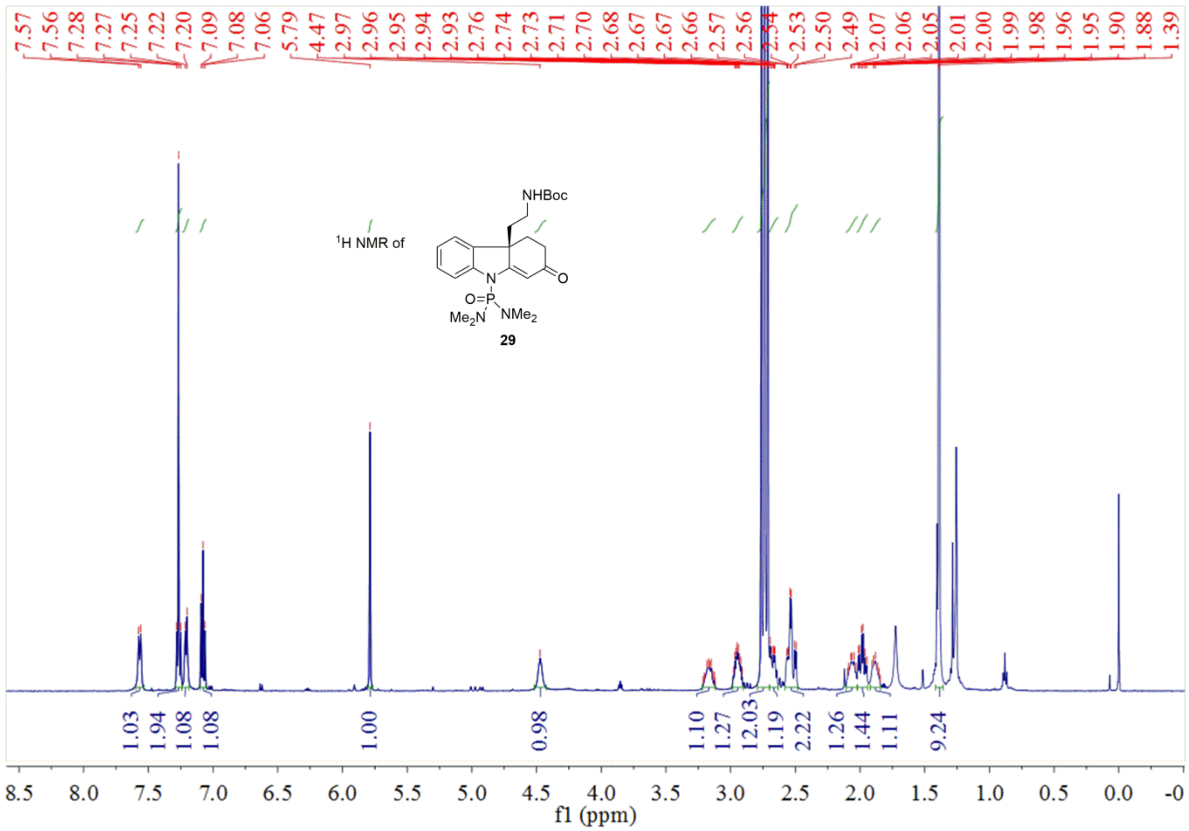
1H NMR of
NHBoc
O=P
Me2N
NMe2
29
f1 (ppm)

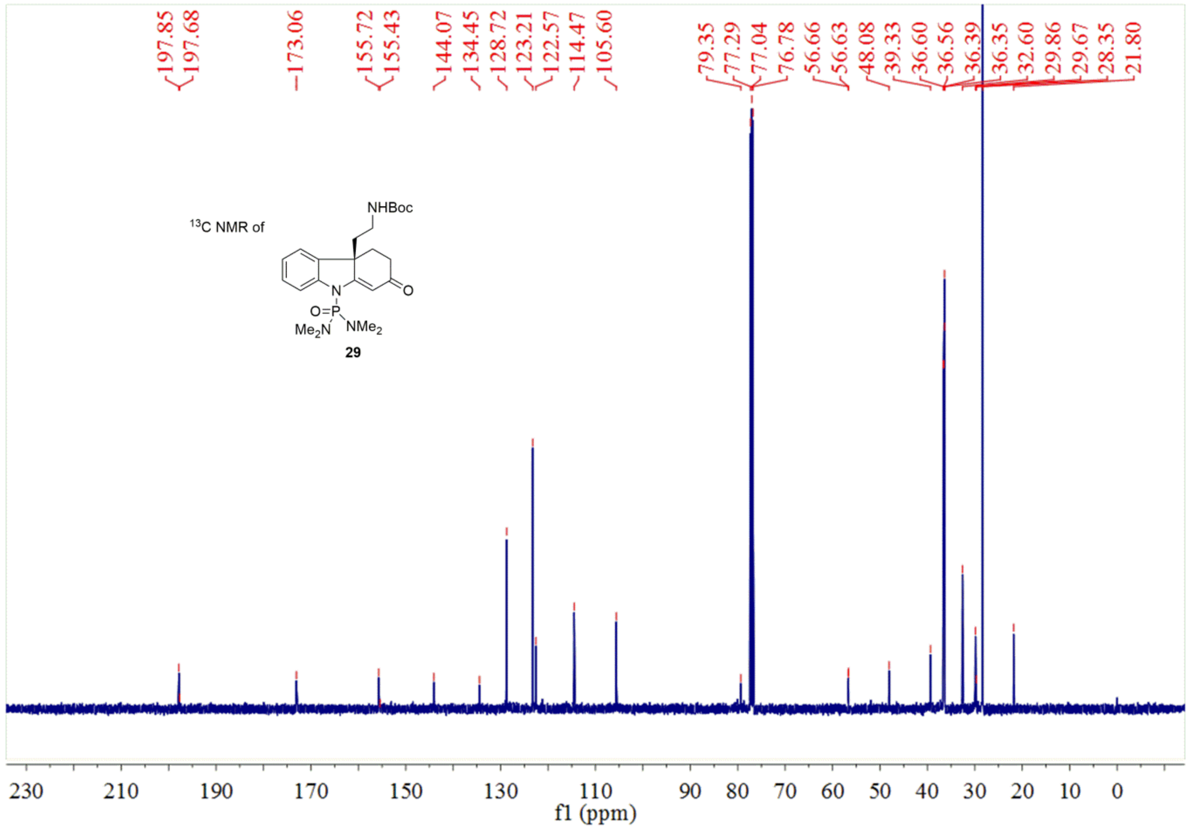
13C NMR of
NHBoc
O=P
Me2N
NMe2
29
f1 (ppm)

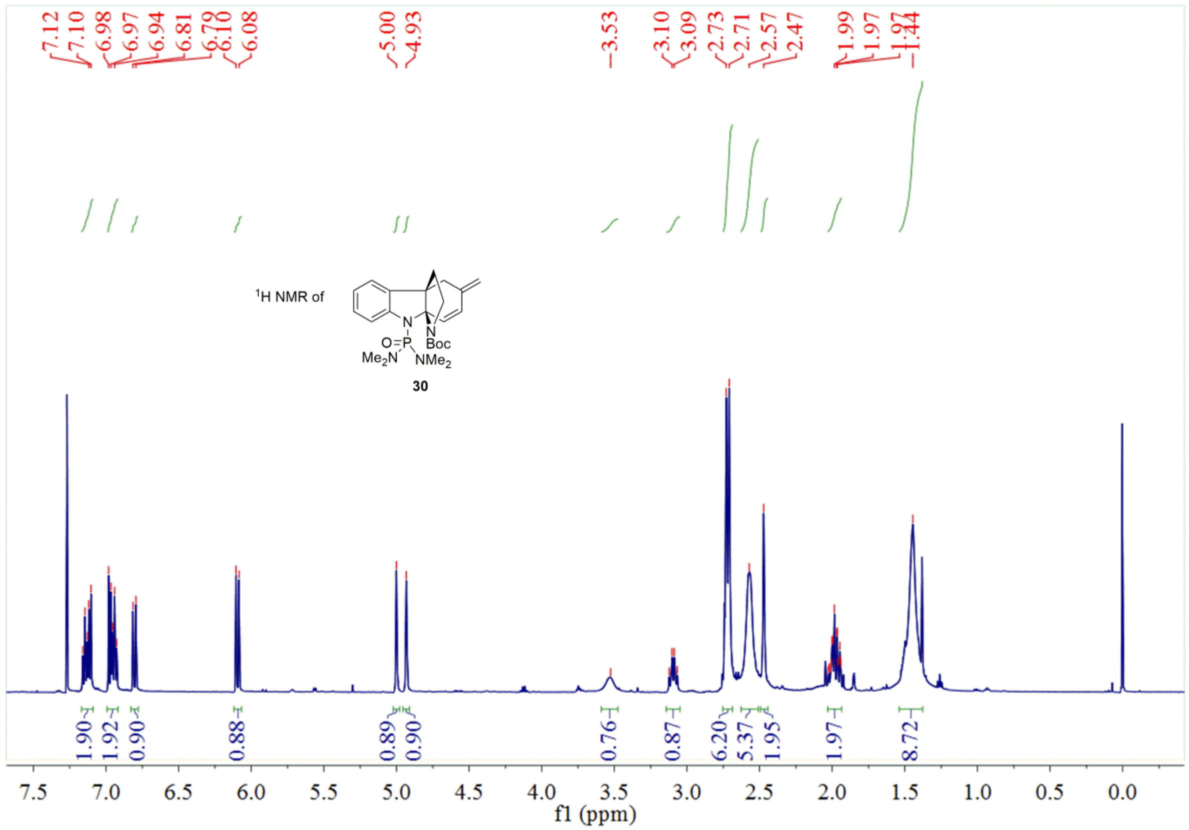

7.12
7.10
6.98
6.97
6.94
6.81
6.79
6.10
6.08
5.00
4.93
3.53
3.10
3.09
2.73
2.71
2.57
2.47
1.99
1.97
1.97
1.44
^{1}H NMR of
O=P
N
Boc
Me_2N
NMe_2
30
1.90
1.92
0.90
0.88
0.89
0.90
0.76
0.87
6.20
5.37
1.95
1.97
8.72
f1 (ppm)

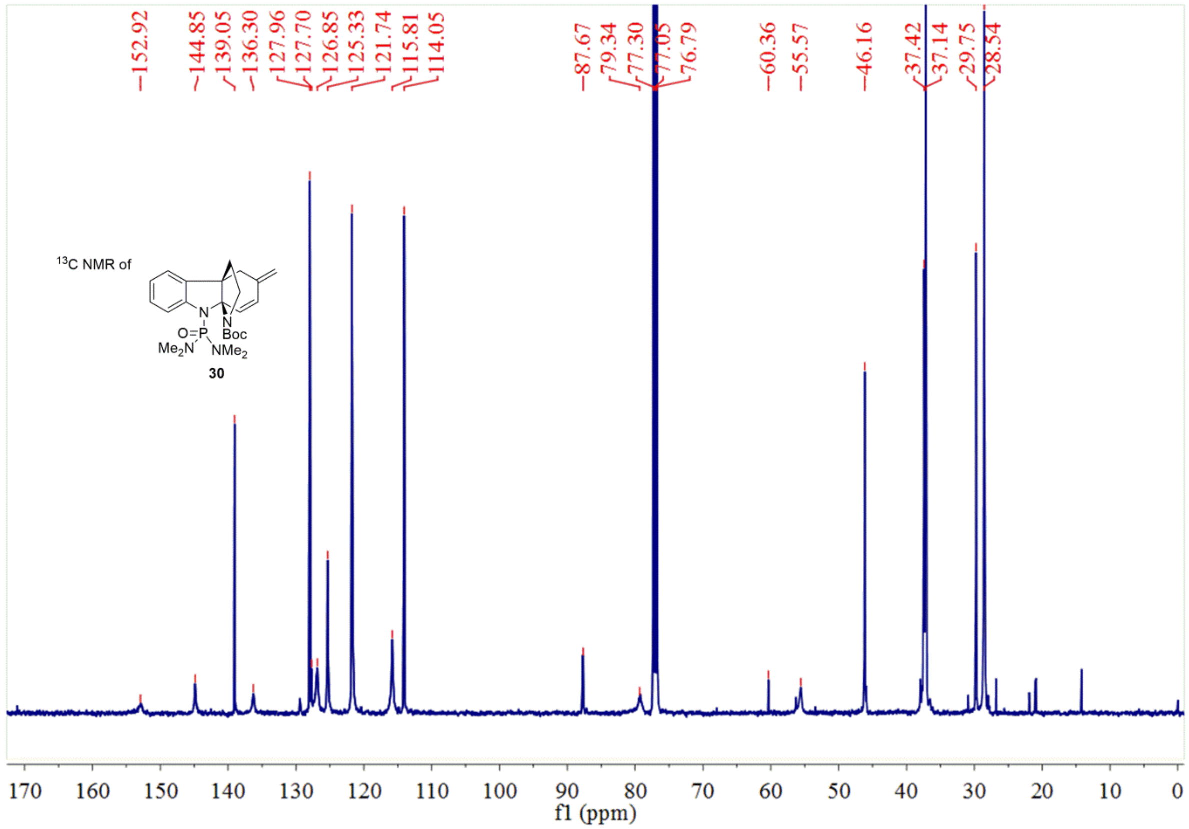

152.92
144.85
139.05
136.30
127.96
127.70
126.85
125.33
121.74
115.81
114.05
87.67
79.34
77.30
77.05
76.79
60.36
55.57
46.16
37.42
37.14
29.75
28.54
^{13}C NMR of
O=P
N
Boc
Me_2N
NMe_2
30
f1 (ppm)

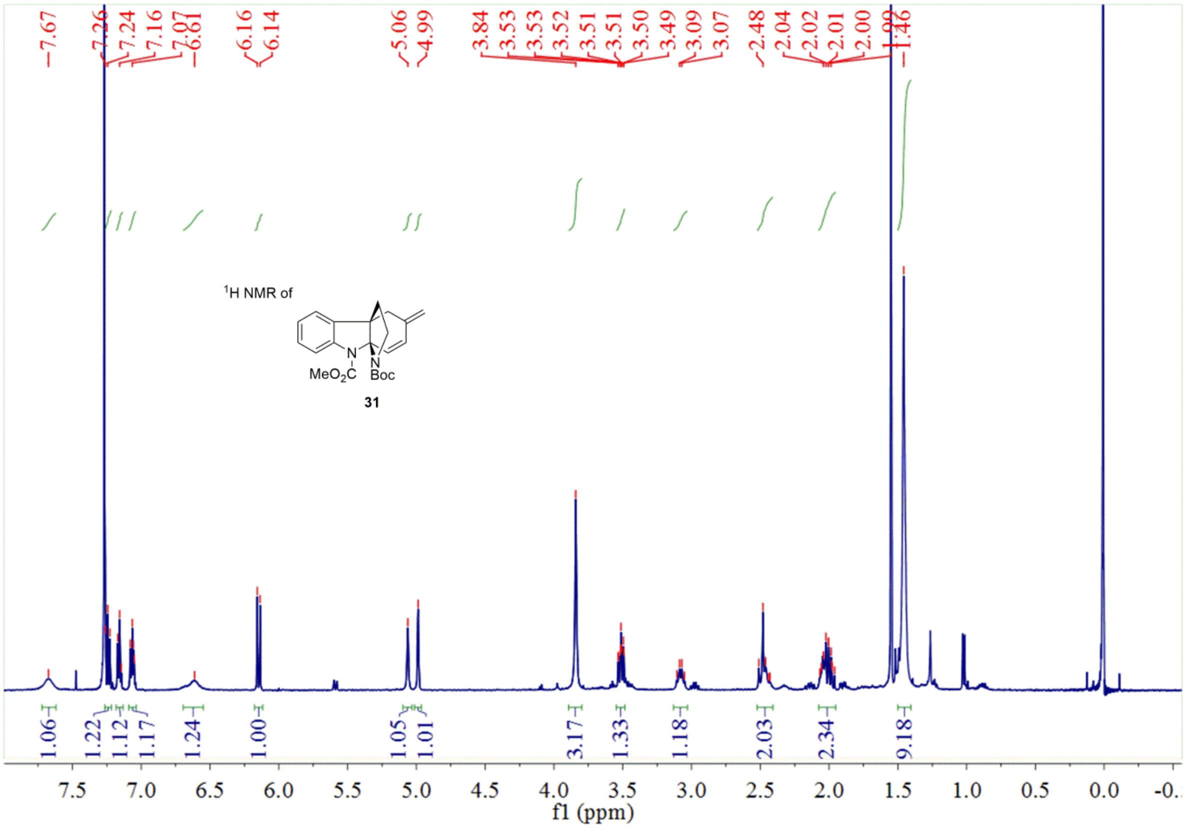
-7.67
7.26
7.24
7.16
7.07
6.61
6.16
6.14
5.06
4.99
3.84
3.53
3.53
3.52
3.51
3.51
3.50
3.49
3.09
3.07
2.48
2.04
2.02
2.01
2.00
1.99
1.46
1H NMR of
MeO2C
N
N
Boc
31
1.06
1.22
1.12
1.17
1.24
1.00
1.05
1.01
3.17
1.33
1.18
2.03
2.34
9.18
f1 (ppm)

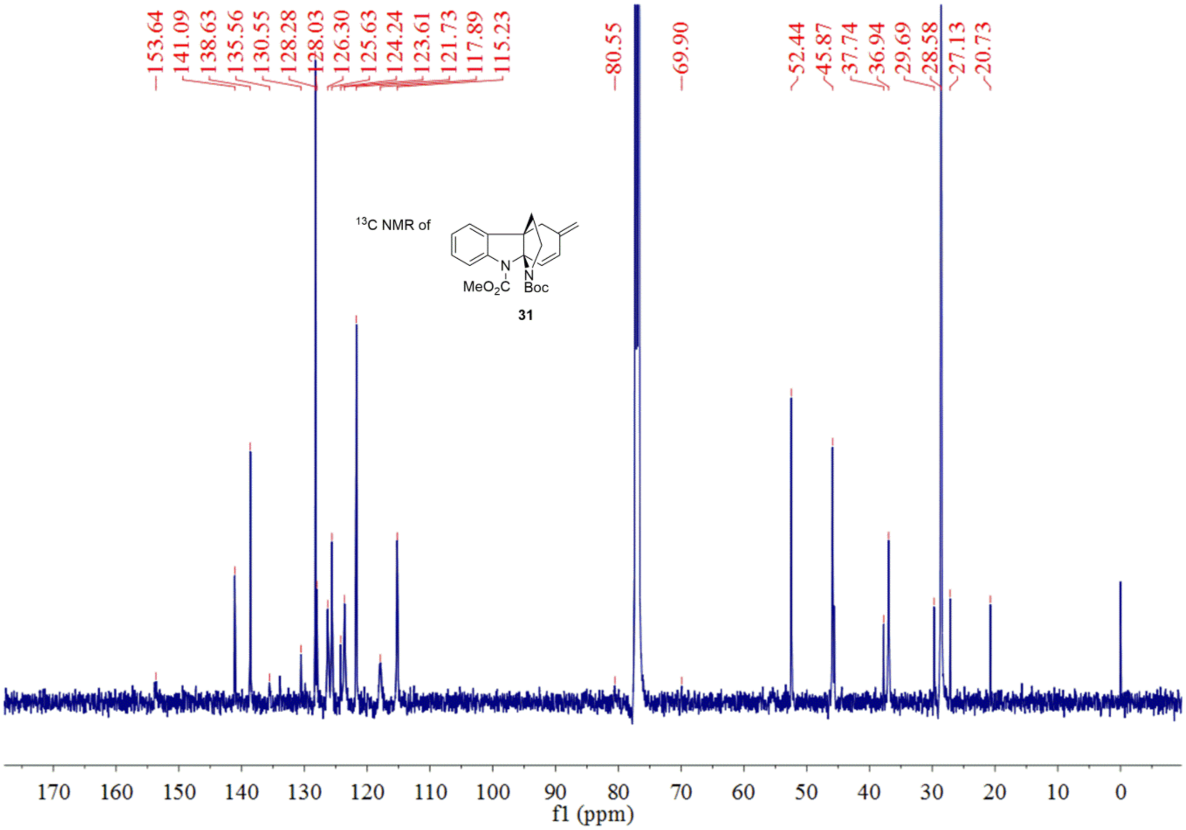
153.64
141.09
138.63
135.56
130.55
128.28
128.03
126.30
125.63
124.24
123.61
121.73
117.89
115.23
80.55
69.90
52.44
45.87
37.74
36.94
29.69
28.58
27.13
20.73
13C NMR of
MeO2C
N
N
Boc
31
f1 (ppm)

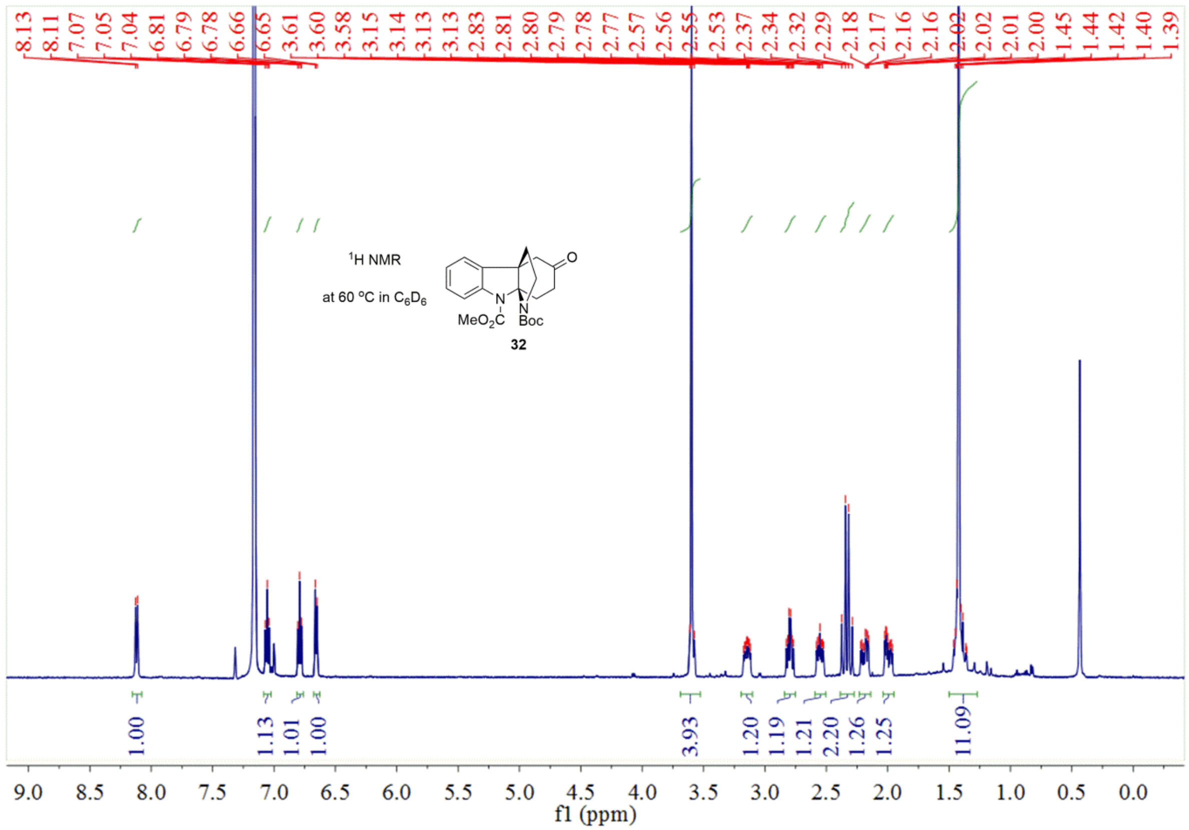
1H NMR
at 60 °C in C6D6
MeO2C
Boc
32
f1 (ppm)

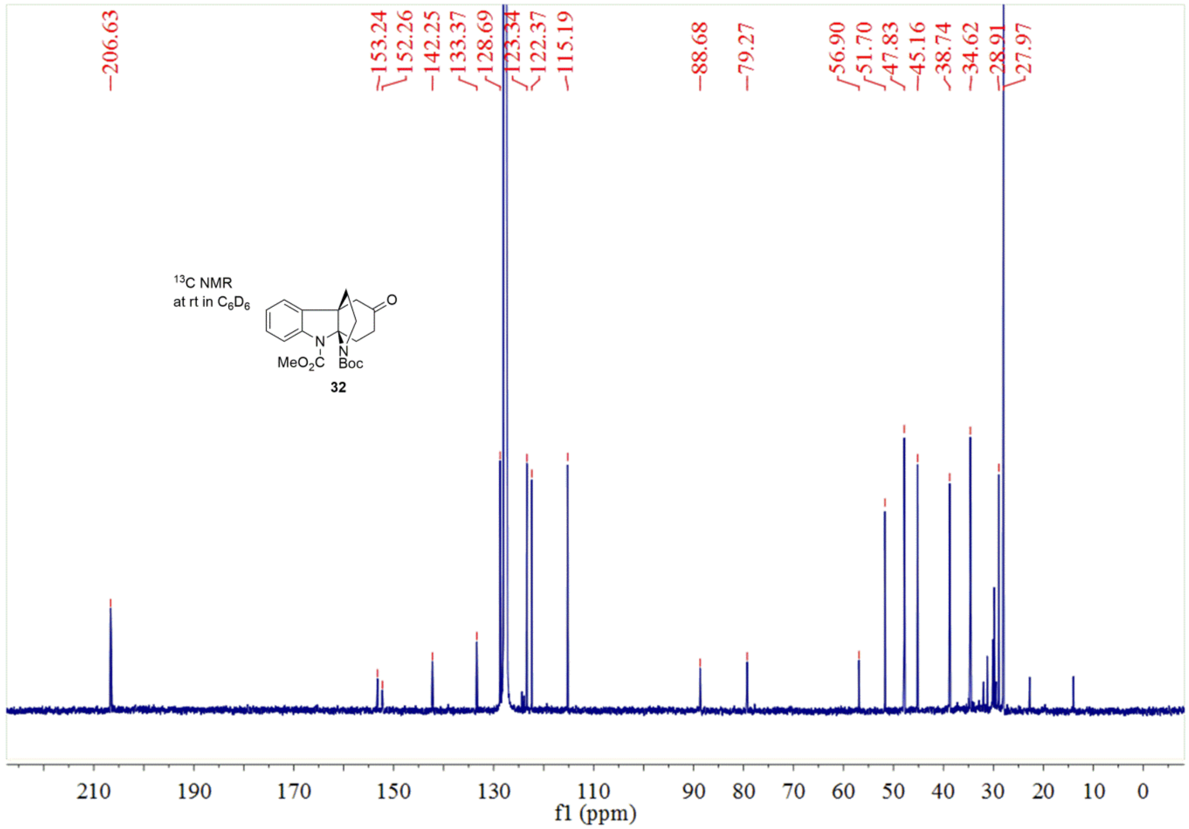
13C NMR
at rt in C6D6
MeO2C
Boc
32
f1 (ppm)
